# *trans*-Cyclooctene- and Bicyclononyne-Linked Nucleotides for Click Modification of DNA with Fluorogenic Tetrazines and Live Cell Metabolic Labeling and Imaging

**DOI:** 10.1021/acs.bioconjchem.3c00064

**Published:** 2023-03-27

**Authors:** Ambra Spampinato, Erika Kužmová, Radek Pohl, Veronika Sýkorová, Milan Vrábel, Tomáš Kraus, Michal Hocek

**Affiliations:** † Institute of Organic Chemistry and Biochemistry, Czech Academy of Sciences, Flemingovo Namesti 2, Prague 6 CZ-16610, Czech Republic; ‡ Department of Organic Chemistry, Faculty of Science, Charles University in Prague, Hlavova 8, Prague 2 12843, Czech Republic

## Abstract

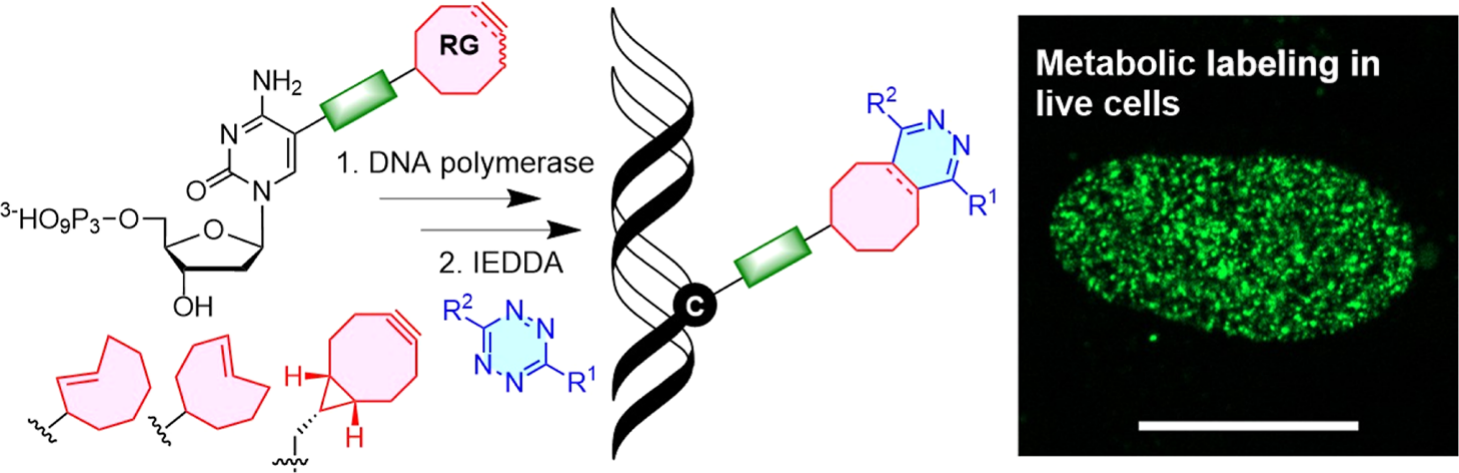

A series of 2′-deoxyribonucleoside triphosphates (dNTPs) bearing 2- or 4-linked *trans*-cyclooctene (TCO) or bicyclononyne (BCN) tethered through a shorter propargylcarbamate or longer triethyleneglycol-based spacer were designed and synthesized. They were found to be good substrates for KOD XL DNA polymerase for primer extension enzymatic synthesis of modified oligonucleotides. We systematically tested and compared the reactivity of TCO- and BCN-modified nucleotides and DNA with several fluorophore-containing tetrazines in inverse electron-demand Diels–Alder (IEDDA) click reactions to show that the longer linker is crucial for efficient labeling. The modified dNTPs were transported into live cells using the synthetic transporter **SNTT1**, incubated for 1 h, and then treated with tetrazine conjugates. The PEG3-linked 4TCO and BCN nucleotides showed efficient incorporation into genomic DNA and good reactivity in the IEDDA click reaction with tetrazines to allow staining of DNA and imaging of DNA synthesis in live cells within time periods as short as 15 min. The BCN-linked nucleotide in combination with TAMRA-linked (TAMRA = carboxytetramethylrhodamine) tetrazine was also efficiently used for staining of DNA for flow cytometry. This methodology is a new approach for *in cellulo* metabolic labeling and imaging of DNA synthesis which is shorter, operationally simple, and overcomes several problems of previously used methods.

## Introduction

Metabolic labeling of replicating DNA is one of the most efficient approaches to monitor cell cycle progression used in experimental cell biology, cancer research, and clinical medicine.^1^ Traditional methods for the detection and quantification of DNA replication rely on the incorporation of modified nucleotide analogues bearing halogen (BrdU, IdU)^2^ or alkyne (EdU)^3^ substituents into the replicating DNA during the S phase of the cell cycle and the subsequent detection of incorporated nucleotides by autoradiography or fluorescence detection by either immunocytochemical staining (BrdU, IdU) or chemical reaction (EdU). Methods employing fluorescent probes require permeabilization of the cell membrane, i.e., fixation of cells, to allow the washout of unreacted nucleosides and entry of fluorescent labeling reagents. Insufficient removal of unreacted nucleosides and fluorescent labeling reagents prior to flow cytometry analysis or fluorescence microscopy imaging would result in the emission of a significant amount of false-positive fluorescence signal, which would prevent subsequent deconvolution of the data.

Recent efforts have been aimed at labeling nascent DNA in *live* cells or even in whole organisms. A novel one-step labeling protocol was reported^4^ that relies on the incorporation of metabolically active fluorescently labeled deoxynucleoside triphosphates (FL-dNTPs) into genomic DNA in live cells. This method^4−6^ is rapid and operationally simple, and it does not require permeabilization of the cell membrane; polar and negatively charged FL-dNTPs are delivered into the cells by the synthetic nucleoside triphosphate transporter (**SNTT1**),^4^ and the unreacted residual material is exported by the cells themselves—presumably by efflux pumps. This natural clearance mechanism allows us to acquire images of labeled DNA with a high signal-to-noise ratio in live cells within less than 1 h after their treatment, without the need for fixation and washing of the cells. Recently, Luedtke’s group has reported metabolic labeling in live organisms through injection^7^ or even through spontaneous uptake^8^ of TAMRA-linked (TAMRA = carboxytetramethylrhodamine) dATP. Alternatively, a two-step approach of DNA labeling in live cells employing 5-vinyl-2′-deoxyuridine (VdU) was recently described.^9−11^ The first step involves *in situ* (*in cellulo*) triphosphorylation of VdU followed by its incorporation into DNA. The post-labeling step is performed either through a reaction with cell membrane-permeable tetrazole–coumarin conjugates^11^ under UV (350 nm) irradiation in cellulo or with the acridine orange-tetrazine reagent^10^ by means of inverse electron-demand Diels–Alder (IEDDA) reaction. Both methods require a relatively long period (16 h) of incubation of cells with VdU, followed by 4 h of incubation with the reagents. Furthermore, the tetrazole method^11^ requires irradiation of live cells with potentially harmful UV light (350 nm).

Although two-step labeling protocols are more complex compared to direct labeling using **SNTT1** with FL-dNTP, there are cases where they can serve as first choice methods: when the fluorophore moiety prevents efficient incorporation of FL-dNTP into DNA by cellular DNA polymerases. In the course of testing a wide range of commercially available FL-dNTPs (data not shown) as well as new FL-dNTPs developed in our lab, we observed that while all tested FL-dNTPs were transported into cells by **SNTT1**, only some of them were efficiently incorporated into genomic DNA,^4−6^ and others were incorporated either inefficiently^12^ or not at all.^13−15^ In such cases, fluorescent labeling could, in principle, be achieved by **SNTT1**-assisted delivery of dNTPs equipped with a “clickable handle”. These dNTPs need to be good substrates for intracellular DNA polymerases, and after their incorporation into DNA, the clickable handles of the nucleotides must be able to undergo a chemical post-labeling performed with suitably modified fluorophores in live cells. There have been many examples^16,17^ of clickable nucleosides and nucleotides used in the chemical^18,19^ or enzymatic^20−26^ synthesis of clickable nucleic acid probes, including several dNTPs (mostly 2′-deoxyuridine nucleotides) bearing *trans*-cyclooctene (TCO),^21−23^ bicyclononyne (BCN),^24^ or cyclopropene^25,26^ moiety that undergo strain-promoted azide–alkyne cycloadditions with azides or IEDDA reactions with various tetrazines. However, these studies were so far limited to *in vitro* DNA labeling. The ability to perform the labeling reactions in the complex environment of the nuclei in living cells would significantly increase the impact and applicability of this approach.

**Figure 1:**
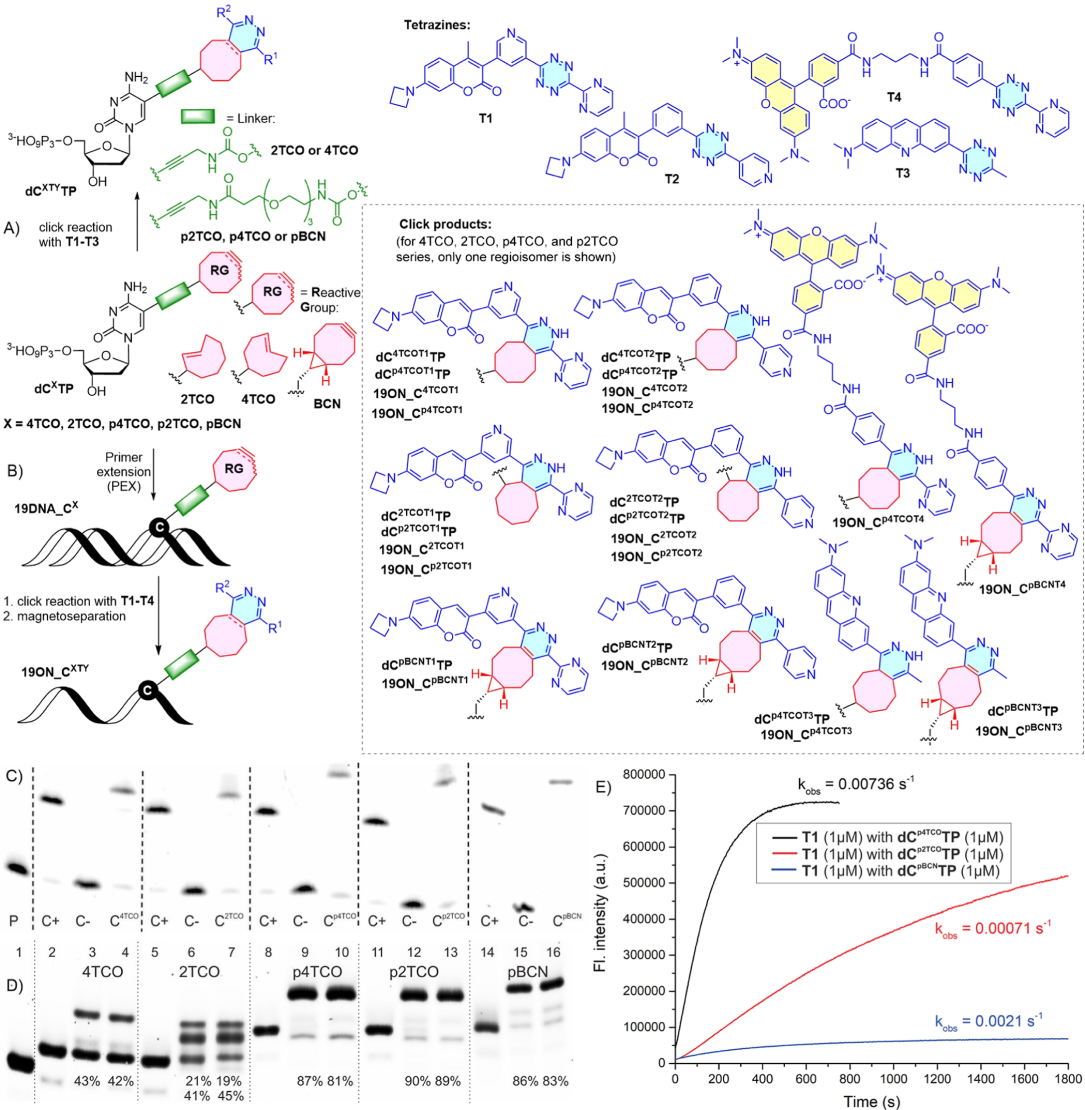
(A) Click reaction of **dC**^**X**^**TP** with tetrazines **T1**-**T3**; (B) enzymatic synthesis of **19DNA_C**^**X**^ (**X = 4TCO**, **2TCO**, **p4TCO**, **p2TCO**, **pBCN**) and the consequent click reaction of **19DNA_C**^**X**^ with tetrazines **T1**–**T4** and magnetoseparation to obtain **19ON_C**^**XTY**^; (C) PAGE analysis of the PEX reactions with modified **dC**^**X**^ (**X = 4TCO**, **2TCO**, **p4TCO**, **p2TCO**, **pBCN**) using KOD XL DNA polymerase, primer prim^A^, and template Temp^19_1C^. P: primer, (C+): natural dGTP, (C−): natural dGTP without dCTP, (**C**^**TCO/BCN**^): **dC**^**4TCO**^ or **dC**^**2TCO**^**dC**^**p4TCO**^ or **dC**^**p2TCO**^ or **dC**^**pBCN**^, dGTP; (D) denaturing PAGE analysis of the DNA-tetrazine reaction of **19DNA_C**^**4TCO**^ with **T1** (5 μM) lane 3 or **T1** (50 μM) lane 4; of **19DNA_C**^**2TCO**^ with **T1** (5 μM) lane 6 or **T1** (50 μM) lane 7; of **19DNA_C**^**p4TCO**^ with **T1** (5 μM) lane 9 or **T1** (50 μM) lane 10; of **19DNA_C**^**p2TCO**^ with **T1** (5 μM) lane 12 or **T1** (50 μM) lane 13; of **19DNA_C**^**pBCN**^ with **T1** (5 μM) lane 15 or **T1** (50 μM) lane 16. Negative controls (−): **19DNA**^**natural**^ lane 1, **19DNA_C**^**4TCO**^ lane 2, **19DNA_C**^**2TCO**^ lane 5, **19DNA_C**^**p4TCO**^ lane 8, **19DNA_C**^**p2TCO**^ lane 11, **19DNA_C**^**pBCN**^ lane 14. (E) Fluorescence time-lapse measurements of **dC**^**p4TCO**^**TP** or **dC**^**p2TCO**^**TP** or **dC**^**pBCN**^**TP** with **T1** tetrazine showing changes in the fluorescence signal of the click products in time (Supporting Information, Section S3.1, Figure S10C).

In this paper, we report on a systematic study of several new dCTPs bearing TCO or BCN moieties linked through different tethers and compare their substrate activity for polymerase incorporation, reactivity with different tetrazines, efficiency of transport across the cell membranes using **SNTT1**, and capacity for live cell metabolic labeling through incorporation of the reactive nucleotides into genomic DNA followed by the IEDDA click reactions. As reactive counterparts, several fluorophore-linked tetrazine conjugates were tested. In particular, we turned our attention to recently described^27^*fluorogenic* coumarin–tetrazine conjugates **T1** and **T2** (Figure 1) because (i) they were reported to be compatible with live cells and (ii) the rates of the reactions of these conjugates with TCO and BCN moieties were very high, requiring reaction times on the order of minutes. In addition, we included in this study a recently described^10^ fluorogenic acridine orange–tetrazine conjugate **T3** “PINK” as well as a commercially available 5-TAMRA-pyrimidyl-tetrazine conjugate **T4**. The use of fluorogenic probes is beneficial especially in the context of living cells, where high signal-to-noise ratio is crucial for achieving good quality data without the need for extensive washing steps.^28^

## Synthesis

**Scheme 1:**
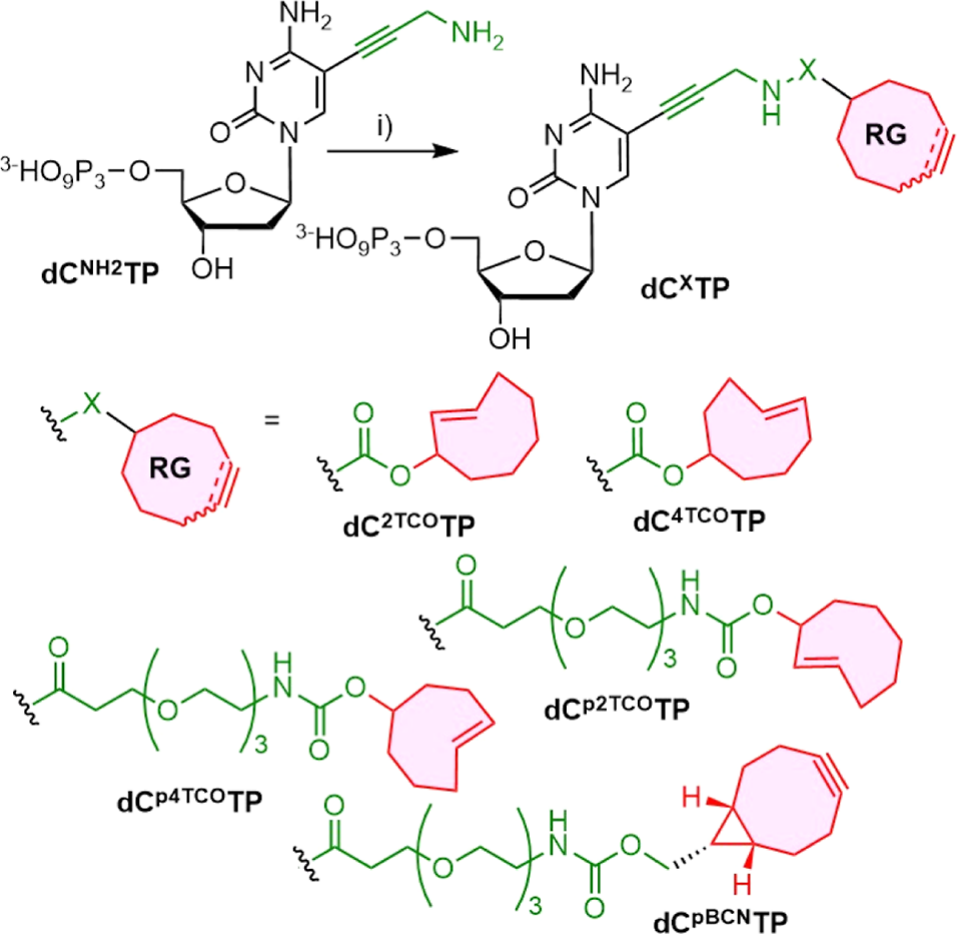
Synthesis of 4TCO-, 2TCO-, p4TCO-, p2TCO-, and pBCN-Modified 2′-Deoxycitidine Triphosphates Conditions: (i) 2-TCO–NHS–carbonate, or 4-TCO–NHS–carbonate, or 2-TCO-PEG3-NHS-carbonate, or 4-TCO-PEG3-NHS-carbonate, or endo-BCN-PEG3-NHS-ester in H_2_O/TEAB 1 M, dimethylformamide, 55 °C, 4 h.

The design of the dNTP conjugates bearing reactive TCO and BCN groups was partly inspired by the above-mentioned prior studies,^21−24^ but the synthetic routes were adjusted according to synthetic approaches used in our lab. Thus, we prepared four dCTP derivatives with appended *trans*-cyclooct-2-en-1-oxy- (2-TCO) and *trans*-cyclooct-4-en-1-oxy- (4-TCO) reactive groups (Scheme 1) tethered to the nucleobase either via a short linker (**dC**^**2TCO**^**TP**, **dC**^**4TCO**^**TP**) or via a triethyleneglycol (PEG3) spacer (**dC**^**p2TCO**^**TP** and **dC**^**p4TCO**^**TP**). In addition, we synthesized a new BCN derivative **dC**^**pBCN**^**TP**. We chose 5-propargylamino-2′-deoxycitidine triphosphate **dC**^**NH2**^**TP**(29,30) and its corresponding 5′-monophosphate **dC**^**NH2**^**MP** as the key intermediates for the synthesis of all functionalized nucleotides. The reactive TCO dienophile groups were installed to **dC**^**NH2**^**TP** or **dC**^**NH2**^**MP** through carbamate moieties. Thus, the reactions of **dC**^**NH2**^**TP** with 2-TCO-*N*-succinimidyl or 4-TCO-*N*-succinimidyl esters gave the corresponding **dC**^**2TCO**^**TP** and **dC**^**4TCO**^**TP** in 29 and 35% yields, respectively. We also prepared the corresponding model **dC**^**4TCO**^**MP** by the analogous reaction of **dC**^**NH2**^**MP** with 4-TCO-*N*-succinimidyl ester in 48% yield (see Scheme S1 in the Supporting Information). Similarly, the reactions of **dC**^**NH2**^**TP** with 2-TCO-PEG3 or 4-TCO-PEG3 succinimidyl esters gave PEG3 spacer-containing **dC**^**p2TCO**^**TP** and **dC**^**p4TCO**^**TP** nucleotides in 35 and 37% yields, respectively. The BCN derivative **dC**^**pBCN**^**TP** was prepared in 20% yield by the reaction of the nucleotide **dC**^**NH2**^**TP** with *endo*-BCN-PEG3-succinimidyl ester. All final nucleotide products were thoroughly purified by high-performance liquid chromatography (HPLC), and their concentration in aqueous stock solutions was determined by the ERETIC (Electronic REference To access *In vivo* Concentrations) method^31^ prior to their use.

### Enzymatic Incorporation

All five modified dNTPs (**dC**^**2TCO**^**TP**, **dC**^**4TCO**^**TP**, **dC**^**p2TCO**^**TP**, **dC**^**p4TCO**^**TP**, and **dC**^**pBCN**^**TP**) were tested as substrates for KOD XL DNA polymerase in a primer extension (PEX) reaction (Figure 1C) using a FAM-labeled 15 nt primer along with 19 nt template encoding for the incorporation of one modified **dC**^**X**^ nucleotide followed by three guanines (Table S1 in the Supporting Information). Monitoring of the reactions by means of denaturing PAGE analyses showed (Figures 1C, S3, and S4) an efficient incorporation of each of the modified nucleotides into full-length 19-mer double-stranded oligonucleotide products containing one modification (Table S2 in the Supporting Information). Corresponding modified single-stranded oligonucleotides (ssON) were prepared by semipreparative scale PEX using a single- or double-biotinylated template followed by magnetoseparation on streptavidin-coated magnetic beads and were characterized by matrix-assisted laser desorption ionization time-of-flight mass spectrometry (MALDI–TOF MS) (Table S3 in the Supporting Information), confirming the identity of all the corresponding modified ON products.

### Reactivity of Modified Nucleotides with Tetrazine–Fluorophore Conjugates

In order to characterize the product of the IEDDA reaction, a model reaction of **dC**^**4TCO**^**MP** with **T5** (3,6-di-2-pyridyl-1,2,4,5-tetrazine) was performed, which provided 1,4-dihydropyridazine derivative **dC**^**4TCOT5**^**MP** (see the Supporting Information, Section S1.4). MS and nuclear magnetic resonance (NMR) confirmed the formation of the desired conjugate; however, the complete assignment of ^1^H and ^13^C NMR spectra was impossible because the product is a mixture of several regio- and stereoisomers^32^ (for details, see the Supporting Information). Therefore, all other products of IEDDA click reactions were characterized only by LC–MS.

Then, the reactivity of all functionalized nucleoside triphosphates (**dC**^**2TCO**^**TP**, **dC**^**4TCO**^**TP**, **dC**^**p2TCO**^**TP**, **dC**^**p4TCO**^**TP**, and **dC**^**pBCN**^**TP**) with an excess of tetrazine conjugates **T1**–**T3** (Figures 1A and S5–S9 in the Supporting Information) was tested in an aqueous solution. The reactions with **T1** or **T2** proceeded at 37 °C for 30 min, while the reaction with **T3** was for 18 h. The outcome was followed by LC–MS, and in all cases, the expected click conjugates **dC**^**XTY**^**TP** were formed and characterized by MS. The conversions (see Table S5 in the Supporting Information) were lower for dNTPs containing the shorter propargyl-carbamate linker (27–78%) and better for the compounds bearing the longer PEG3-based linker (55–90%), and the reactivity of tetrazine **T2** was lower than that of tetrazines **T1** and **T3**. In parallel, we studied the outcome and kinetics of the IEDDA click reactions by fluorescence spectroscopy. The reactions of **T1** with TCO- and BCN-linked nucleotides were performed by mixing equimolar amounts of reactants in quartz cuvettes to achieve their final 1 μM concentration in phosphate-buffered saline. To calculate “turn on” values, i.e., increase of fluorescence intensity resulting from the difference of the fluorescence intensities exhibited by the products and the starting materials, the fluorescence of individual reactants was measured and subtracted from that of the reaction mixture recorded after 30 min. The largest turn-on effects, 210- and 220-fold, were observed with **dC**^**4TCO**^**TP** and **dC**^**p4TCO**^**TP**, respectively. The analogous 2-TCO isomers, **dC**^**2TCO**^**TP** and **dC**^**p2TCO**^**TP**, showed somewhat diminished light-up effect: 130- and 125-fold, respectively. These results indicate that the apparent light-up effect measured at 30 min arbitrary time point was significantly higher for the 4-TCO nucleotides and that the length of the linker did not affect the observed light-up effect. Kinetic analysis of the reactions of **dC**^**p2TCO**^**TP**, **dC**^**p4TCO**^**TP**, and **dC**^**pBCN**^**TP**, respectively, with **T1** (Figure 1E) revealed significant differences in rate constants for reactions of 4-TCO versus 2-TCO derivatives; **dC**^**p4TCO**^**TP** displays a reaction rate (*k*_obs_ = 0.00736 s^–1^) one order of magnitude higher than that of **dC**^**p2TCO**^**TP** (0.00071 s^–1^). In order to have a comparison of the fluorescence intensity increase of the products at the same concentration, fluorescence intensities at half-life of reactions were calculated (Table S6 in the Supporting Information).

Their comparison revealed that both products of reactions of **T1** with **dC**^**p2TCO**^**TP** and **dC**^**p4TCO**^**TP** have comparable fluorescence intensities, indicating that the lower turn-on value observed for **dC**^**p2TCO**^**TP** at the 30 min time point is entirely due to the lower reaction rate. The reaction of **dC**^**pBCN**^**TP** with **T1** exhibits a lower reaction rate (0.0021 s^–1^), and the product displays a significantly lower brightness than the TCO products at half-life. The analogous reactions with tetrazine **T2** gave only a 3–5-fold increase of fluorescence. The reactions were performed by mixing of 1 mM solutions, which were diluted to their final 1 μM concentration after a 60 min reaction period; a 5-fold light-up effect (Figure S8 in the Supporting Information) was obtained for the reaction of **dC**^**4TCO**^**TP**, whereas a 3-fold increase of fluorescence was observed for **dC**^**2TCO**^**TP** and **dC**^**pBCN**^**TP**. The rate constant (*k*_obs_ = 0.00402 s^–1^) for the reaction of **dC**^**p4TCO**^**TP** and **T2** was about half of that observed with **T1**. The relatively small observed light-up effect may also be a consequence of a relatively larger fluorescence of the **T2** itself or decomposition impurities arising from **T2** also observed in an earlier study.^27^ Acridine orange-tetrazine^10^ “PINK” conjugate **T3** showed measurable light-up with **dC**^**p4TCO**^**TP** and **dC**^**pBCN**^**TP**; due to the lower reactivity of **T3**, the reaction at a concentration of 1 mM was run for 2 h at 37 °C, yielding light-up factors of 15 and 50 (Figure S9A,B in the Supporting Information) for **dC**^**p4TCO**^**TP** and **dC**^**pBCN**^**TP**, respectively.

### Fluorescent Labeling of Modified DNA *In Vitro*

Next, we tested the reactivity of the reactive functionalized dsDNA (19-bp; Table S3 in the Supporting Information). Thus, the PEX products (0.2 μM) were incubated with excess (5 or 50 μM) of tetrazines **T1**–**T4** in the H_2_O–DMSO mixture for 30 min at 37 °C, and the reaction mixtures were analyzed by means of PAGE (Figure 1D and S12–S15 in the Supporting Information). All TCO-modified DNAs reacted with **T1**, forming the isomeric mixtures of dihydropyridazine conjugates (Figure 1B,D). The PEG3-linked TCO-derivatives **19DNA_C**^**p4TCO**^ and **19DNA_C**^**p2TCO**^ gave higher conversions (81–90%, Figure S12 in the Supporting Information) than the **19DNA_C**^**4TCO**^ and **19DNA_C**^**2TCO**^ ONs with short linkers (42–64%), indicating the positive role of a longer linker. The multiple bands of the click products of reactions of **19DNA_C**^**2TCO**^ with **T1** correspond to isomers of the products as proved by HPLC–MS analysis (for details, see Figures S11 and S12 in the Supporting Information). Also, the BCN-linked **19DNA_C**^**pBCN**^ was efficiently labeled with **T1**. On the other hand, tetrazine **T2** reacted readily and completely only with BCN-linked **19DNA_C**^**pBCN**^ (Figure S13 in the Supporting Information), whereas the TCO-linked DNA reacted with **T2** poorly with only incomplete conversions. The reaction of **19DNA_C**^**p4TCO**^ and **19DNA_C**^**pBCN**^ with tetrazine **T3** required a higher concentration of **T3** (500 μM) and 18 h of incubation at 37 °C. Under these conditions, the reactions reached completion, but some additional bands appeared in PAGE (Figure S15B in the Supporting Information), indicating the presence of products of decomposition. Tetrazine **T4** showed nearly complete conversion in reaction with **19DNA_C**^**p4TCO**^ (50 μM of **T4**; 30 min at 37 °C), while **19DNA_C**^**p2TCO**^ and **19DNA_C**^**pBCN**^ allowed only partial conversion under these conditions. It is worth noting that we cannot completely rule out the possibility that at least part of the dienophile moiety on the ON degraded during the labeling experiment because the high reactivity of the strained dienophiles is inherently linked to lower stability.^33,34^

### Post-labeling *In Cellulo*

**Figure 2:**
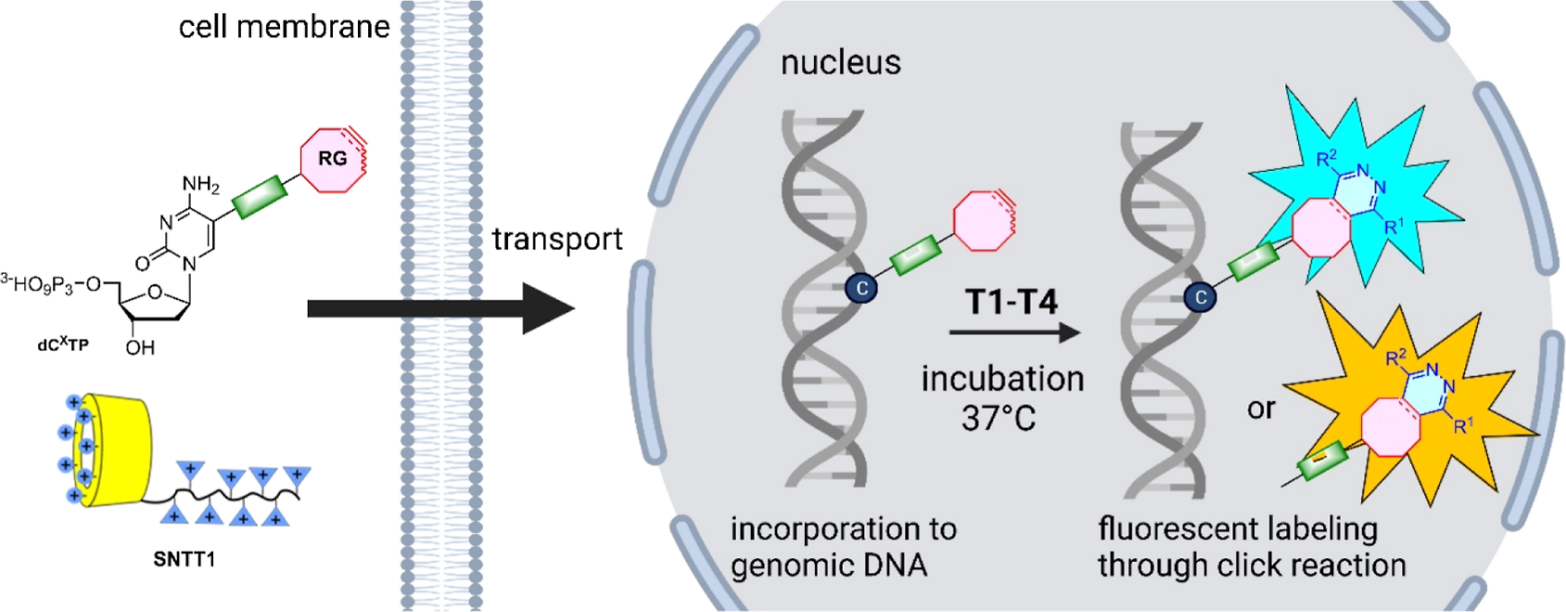
Modified dNTPs (**dC**^**2TCO**^**TP**, **dC**^**4TCO**^**TP**, **dC**^**p2TCO**^**TP**, **dC**^**p4TCO**^**TP**, or **dC**^**pBCN**^**TP**) were delivered to U-2 OS cells with the **SNNT1** transporter and further incubated in the cultivation medium for 60 min. Then, the cells were incubated with tetrazines **T1**–**T4** and imaged by confocal microscopy.

We tested various combinations of reactive group–modified nucleotides and tetrazines **T1**–**T4** for the labeling of DNA in live U-2 OS cells. In a typical experiment, the cells were treated with an equimolar mixture (10 μM) of a particular **dN**^**X**^**TP** (**dC**^**2TCO**^**TP**, **dC**^**4TCO**^**TP**, **dC**^**p2TCO**^**TP**, **dC**^**p4TCO**^**TP**, or **dC**^**pBCN**^**TP**) and the **SNTT1** transporter in tricine buffer for 5 min at 37 °C according to a protocol described earlier^6^ (Figure 2).

**Figure 3:**
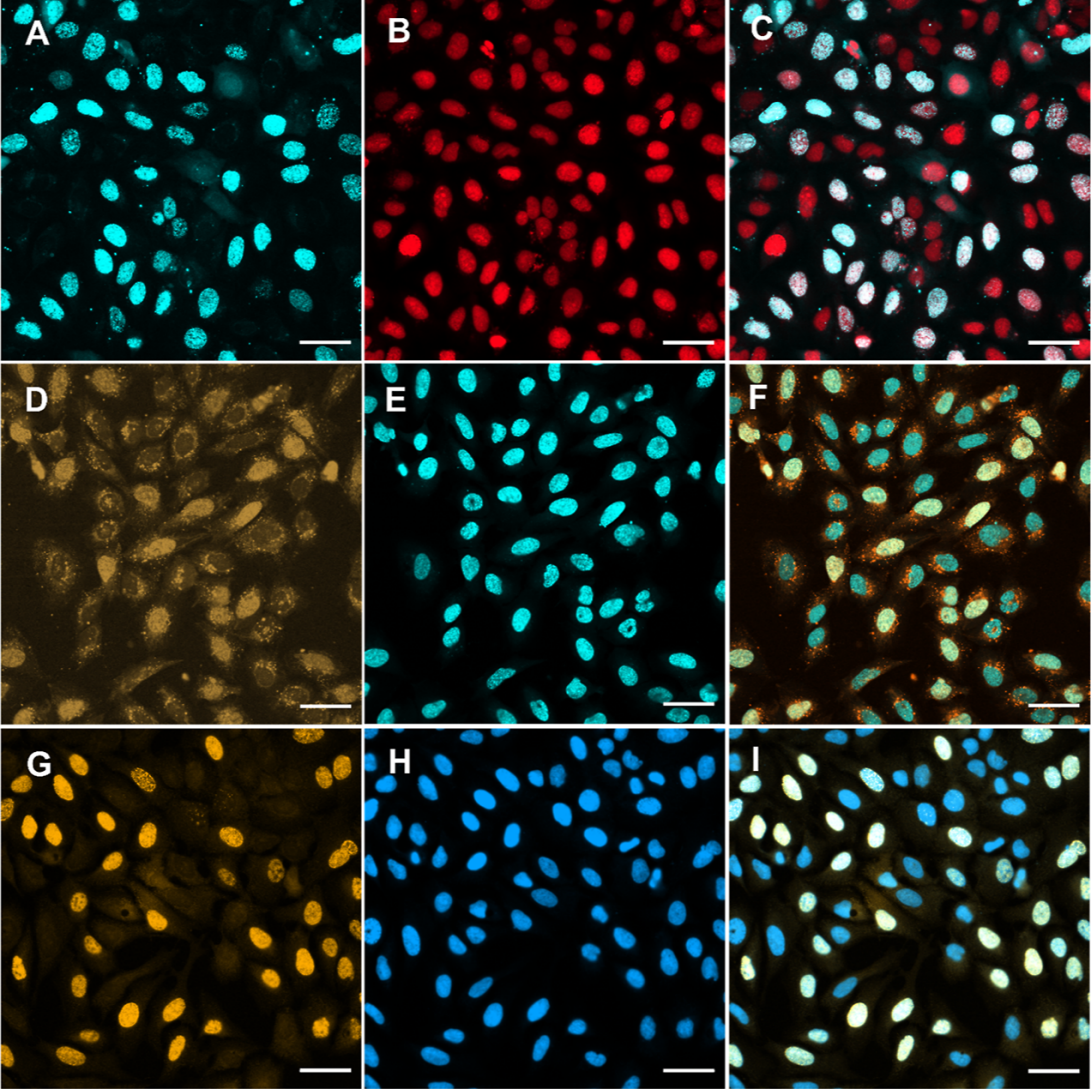
Confocal microscopy imaging of U-2 OS cells with labeled DNA. Cells were treated with **dC**^**p4TCO**^**TP**/**SNTT1** (A–F; 10 μM) or **dC**^**pBCN**^**TP**/**SNTT1** (G–I; 10 μM) for 5 min, then incubated in a medium for 60 min. Subsequently, tetrazine **T1** (A–C; 1 μM; 15 min) or **T3** (D–F; 10 μM; 4 h) was added. Cells incubated with **dC**^**pBCN**^**TP** (G–I) were fixed with methanol before tetrazine **T4** (G–I; 2 μM; 30 min) was added. Prior to microscopy, DRAQ5 (B–C; 2.5 μM), or HOECHST 33342 (E–F; 3 μM), or DAPI (H–I; 0.5 μM) was added. **T1** channel (A), DRAQ5 channel (B), and merged (C); **T3** channel (D), HOECHST 33342 (E), and merged (F); **T4** channel (G), DAPI (H), and merged (I). Bar size 50 μm.

Then, the cells were incubated in a cultivation medium for an additional 60 min, and after this period a working solution of a tetrazine (**T1**–**T4**) in the cultivation medium was added to achieve the desired final concentration. The cells were then incubated at 37 °C on a confocal microscope stage and inspected at regular intervals to observe the progress of DNA labeling, which revealed itself as a punctuate staining of *some* nuclei (only less than a half of U-2 OS cells synthesize DNA at a particular moment; i.e., they are in the S phase). Results are summarized in Table S8 in the Supporting Information. Cells treated with either **dC**^**2TCO**^**TP** or **dC**^**4TCO**^**TP** did not show DNA labeling when exposed to tetrazines **T1** or **T2** (1–10 μM; Figures S19, S20, and S22–S24 in the Supporting Information). Instead, the unspecific staining of mostly cytosolic structures, presumably mitochondria, was observed, while nuclei remained relatively dark. Longer treatment of cells with tetrazines further increased cytosolic fluorescence. Control experiments, in which live cells (Figure S23 in the Supporting Information) were only exposed to tetrazines **T1** or **T2**, gave a similar staining pattern. These experiments indicate that the background (cytosolic) fluorescence stems from the tetrazines, which either bind to cytosolic structures or undergo decomposition^35,36^ in the intracellular space, which results in an increase of the fluorescence of the conjugated coumarines. *In vitro* experiments, which showed inferior reactivity of **19DNA_C**^**4TCO**^ and **19DNA_C**^**2TCO**^ ONs with short linkers, would suggest that the failure of these reagents to label DNA *in cellulo* can be ascribed to low reactivity of the TCO groups appended to the DNA in close proximity. In contrast, when the cells were incubated first with **dC**^**p2TCO**^**TP** or **dC**^**p4TCO**^**TP**, in which the TCO groups were spaced from the nucleobase with the PEG3 linker, and subsequently treated with **T1**, their nuclei showed a clear punctuate pattern, typical for DNA foci (Figures 3A–C and S29 in the Supporting Information). Tetrazine **T2**, however, allowed clear DNA labeling with **dC**^**p4TCO**^**TP**; only **dC**^**p2TCO**^**TP**—treated cells gave unspecific staining (Figure S27 in the Supporting Information).

A comparison of images of cells treated with **dC**^**p2TCO**^**TP** or **dC**^**p4TCO**^**TP** and **T1** acquired under the same conditions showed that the contrast between the background residual fluorescence and DNA signal was about twice as high for **dC**^**p4TCO**^**TP** compared to that achieved with **dC**^**p2TCO**^**TP** (cf. Figures S29 and S31). Tetrazine **T1** a provided higher contrast than **T2** regardless of the nucleotide used (**dC**^**p2TCO**^**TP** or **dC**^**p4TCO**^**TP**). Both tetrazines labeled DNA *in cellulo* in very short times—15 min was sufficient to obtain clear images. Longer reaction times did not improve the contrast; instead an increase of background fluorescence was observed. Next, we tested the labeling of the DNA of cells, which had been incubated with **dC**^**pBCN**^**TP**, with tetrazines **T1** or **T2**. Both tetrazines reacted rapidly and provided visible DNA foci. Nevertheless, the contrast between the labeled DNA and the background was lower than that observed for **dC**^**p4TCO**^**TP**.

We tested the tetrazine conjugate **T3** in live cells, which had been incubated with **dC**^**p2TCO**^**TP**, **dC**^**p4TCO**^**TP**, or **dC**^**pBCN**^**TP**, delivered by **SNTT1**. Clear labeling of DNA was observed with **dC**^**pBCN**^**TP** after 4 h of incubation (Figures 3D–F and S37 in the Supporting Information). However, aside from the fluorescence arising from synthesizing nuclei, a strong signal corresponding to non-specific staining was observed in the cytosol of nearly all cells (Figures 3D and S37). Cells that had been incubated with **dC**^**p2TCO**^**TP** or **dC**^**p4TCO**^**TP** revealed only non-specific fluorescence in various cell compartments (Figures S28 and S32 in the Supporting Information). Prolongation of the treatment of cells with **T3** led to stronger non-specific fluorescence.

Next, we tested the reactivity of a commercially available 5-TAMRA-pyrimidyl-tetrazine **T4** of cells incubated with **dC**^**p4TCO**^**TP** or **dC**^**pBCN**^**TP**. This labeling reagent only sluggishly permeated the cell membrane and required a longer incubation time and higher concentration (20 μM) of **T4** to allow visible DNA foci (Figures S33 and S38 in the Supporting Information). In addition, strong background fluorescence was present in the intracellular space as well as on the plasma membrane. We tested this reagent also in fixed cells; the cells that had been treated with either **dC**^**p4TCO**^**TP** or **dC**^**pBCN**^**TP** delivered by **SNTT1** were then incubated for 60 min in the medium and then fixed subsequently with methanol. These cells were then treated with 2 μM solutions of **T4**, which allowed very clear labeling of DNA (Figure 3, and Figures S34 and S39 in the Supporting Information. A comparison of images of cells, whose DNA was modified by p4TCO versus pBCN, acquired under the same conditions revealed a higher contrast in the case of the latter (Figure S40).

Collectively, these experiments showed that PEG3-2-TCO, PEG3-4-TCO, and PEG3-BCN reactive groups are suitable for DNA labeling in live cells; especially PEG3-4-TCO and PEG3-BCN nucleotides incorporated into DNA show high reactivity with coumarin–tetrazine **T1** and 5-TAMRA-pyrimidyl-tetrazine **T4** conjugates; PINK tetrazine **T3** reacted with the BCN-labeled DNA in live cells with limited efficacy, producing a high unspecific background fluorescence signal. Our data highlight the importance of finding the right combination of dienophile and tetrazine. This appears to be particularly crucial for achieving efficient labeling in living cells.

**Figure 4:**
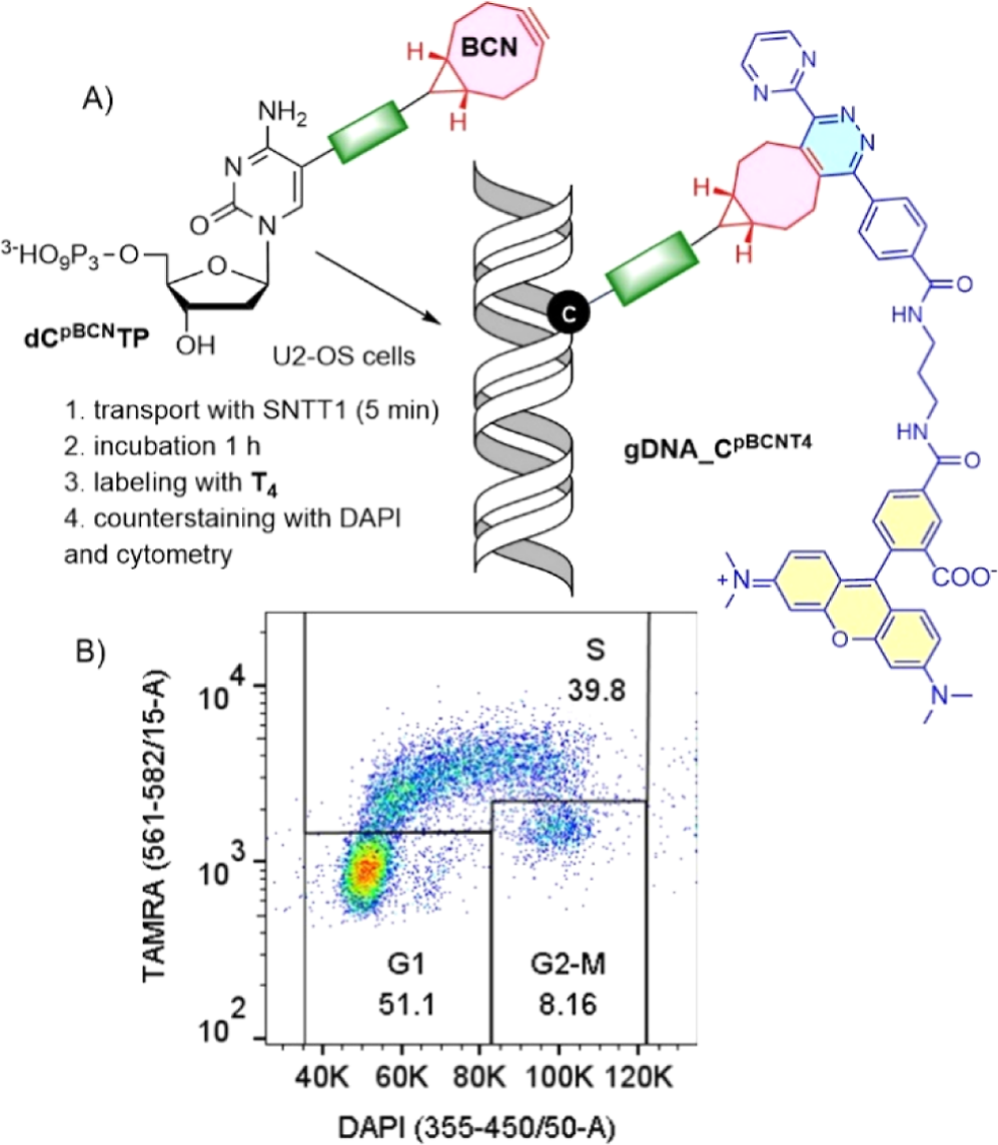
DNA incorporation-based cell cycle analysis of U-2 OS cells. (A) The cells were pulse-treated with a mixture of **dC**^**pBCN**^**TP/SNTT 1** (10 μM) in tricine buffer for 4 min and further incubated in a conditioned medium for 1 h. After this period, the cells were fixed with methanol, incubated with 2 μM TAMRA-tetrazine (**T4**) for 30 min at 37 °C, counterstained with DAPI, and analyzed by flow cytometry (B).

Finally, we tested whether these *in cellulo* labeling methods could be exploited in the DNA incorporation-based cell cycle progression assay using protocols, which we developed recently.^6^ The coumarin–tetrazine conjugate **T1** was tested first. In short, the cells were treated with either **dC**^**p4TCO**^**TP** or **dC**^**pBCN**^**TP** and **SNTT1** in tricine buffer and then incubated in a complete cultivation medium for 60 min. Then, the cells were treated with tetrazine **T1** for 15 min. The cells were subsequently divided into two fractions; the first one was submitted to cell cytometry to check the labeling efficiency (i.e., the difference between fluorescence intensities of the labeled and non-labeled cell populations). To the second fraction, DRAQ5, a widely used cell-permeable far-red fluorescent DNA-staining reagent, was added as a cell-permeable quantitative DNA staining reagent, and the cells were analyzed by cell cytometry as well. The analyses of the first fraction revealed that the two expected populations of cells (G1 and G2/M phase non-labeled vs S phase labeled) are indeed differentiated, but the peaks of fluorescence intensities were separated by one order of magnitude, which is lower than what we observed in our recent work.^6^ The control experiments revealed that the background fluorescence in non-synthesizing cell populations (G1 and G2/M) was relatively high, thus decreasing the separation of the cell populations. Moreover, measurements of the second fraction of cells revealed that the addition of DRAQ5 caused lowering of fluorescence intensity of the S-phase cells. As a result, the separation of the two populations was lost, precluding the bivariate analysis of the cell cycle. Presumably, DRAQ5 that intercalated into DNA quenched the fluorescence of the nearby located coumarin fluorophores; there is, indeed, a partial overlap of DRAQ5 absorption and coumarin emission bands between 400 and 500 nm. In contrast, labeling of DNA with **dC**^**pBCN**^**TP**/**T4** reagents in fixed cells followed with the addition of DAPI resulted in an acceptable separation of all cell cycle phases (Figures 4 and S41).

## Conclusions

We have developed a new approach for a two-step DNA metabolic labeling that proceeds both *in vitro* and in live cells. We have prepared a series of modified nucleoside triphosphates with appended reactive groups (2-TCO, 4-TCO, or BCN) via a short (**dC**^**2TCO**^**TP**, **dC**^**4TCO**^**TP**) or a long PEG3 linker (**dC**^**p2TCO**^**TP**, **dC**^**p4TCO**^**TP**, and **dC**^**pBCN**^**TP**). All prepared modified dCTPs showed various fluorescence “turn-on” values (3–220-fold) upon reaction with fluorogenic tetrazines **T1**–**T3** in the solution. *In vitro*, these dCTPs were efficiently incorporated into 19-mer dsONs in PEX reactions, and the modified ONs were then labeled with four fluorophore–tetrazine conjugates **T1**–**T4** by means of IEDDA reaction. ONs with incorporated nucleotides that had reactive groups attached over the PEG3 linker (**dC**^**p2TCO**^, **dC**^**p4TCO**^, and **dC**^**pBCN**^) underwent smooth labeling reactions with tetrazines, while ONs modified with **dC**^**2TCO**^ or **dC**^**4TCO**^ showed lower reactivity. Similar trends were also observed when the system was applied for the labeling of the nascent DNA in live cells. The modified **dC**^**X**^**TP**s were delivered to cells by means of nucleoside triphosphate transporter **SNTT1** and allowed to incorporate for 60 min; subsequent treatment of cells with one of the tetrazines in question (**T1**–**T4**) revealed that the DNA with incorporated **dC**^**p2TCO**^, **dC**^**p2TCO**^, **dC**^**p4TCO**^, and **dC**^**pBCN**^ was labeled efficiently with **T1**, whereas **T2** could label only the PEG-linked modified DNA. Cells treated with **dC**^**2TCO**^**TP** or **dC**^**4TCO**^**TP** did not show labeling with **T1** or with **T2**. Tetrazine **T3** labeled solely the DNA with incorporated **dC**^**pBCN**^ but required a longer reaction time (4 h). Commercially available **T4** was able to label the DNA with incorporated **dC**^**p4TCO**^ or **dC**^**pBCN**^ in live cells but less efficiently due to low cell membrane permeability. When the cells were fixed after the incorporation of **dC**^**p4TCO**^**TP** or **dC**^**pBCN**^**TP**, labeling with **T4** allowed clear images of nuclei with DNA foci. Notably, the **dC**^**pBCN**^**TP**/**T4** system revealed the best contrast in imaging experiments and was also successfully employed in a cell cycle cytometric assay, which allowed the quantification of cells at different stages of the cell cycle. The two-step procedure developed in this paper enables pulse labeling of DNA in live cells within 75 min. We anticipate that this system can be particularly useful for *in cellulo* labeling of the nascent DNA, especially in cases where the direct incorporation of FL-dNTPs is not possible due to the incompatibility of the fluorophore with cellular polymerases.

## Supporting Information Available

Full experimental section with synthetic procedures and characterization of all compounds, biochemical methods and procedures, fluorescence turn-on measurements and LC–MS of click reactions of modified nucleotides or DNA, additional fluorescence microscopy images, and copies of NMR, MALDI, and LC–MS spectra (PDF)
