# Supplementary material for: trans-Cyclooctene- and Bicyclononyne-Linked Nucleotides for Click Modification of DNA with Fluorogenic Tetrazines and Live Cell Metabolic Labeling and Imaging
Source: Bioconjug Chem. 2023 Mar 27;34(4):772–80. doi: 10.1021/acs.bioconjchem.3c00064 (PMC10119924; doi:10.1021/acs.bioconjchem.3c00064)
Supplement: Supplementary file 1 — bc3c00064_si_001.pdf [file bc3c00064_si_001.pdf]

Supporting Information

Trans-cyclooctene- and Bicyclononyne-Linked Nucleotides for Click Modification of DNA with Fluorogenic Tetrazines and Live Cell Metabolic Labelling and Imaging

Ambra Spampinato,[a,b] Erika Kužmová,[a] Radek Pohl,[a] Veronika Sýkorová,[a] Milan Vrábel,*[a] Tomáš Kraus,*[a] and Michal Hocek*[a,b]

[a] *Institute of Organic Chemistry and Biochemistry, Academy of Sciences of the Czech Republic, Flemingovo namesti 2, CZ-16610 Prague 6, Czech Republic;*

[b] *Department of Organic Chemistry, Faculty of Science, Charles University in Prague, Hlavova 8, Prague-2 12843, Czech Republic.*

Table of contents

1. **Experimental section – organic chemistry part**
    - 1.1. Synthetic scheme
    - 1.2. Synthesis of modified monophosphate and triphosphates
        - 1.2.1. Synthesis of **dC^{4TCO}TP**
        - 1.2.2. Synthesis of **dC^{NH2}MP**
        - 1.2.3. Synthesis of **dC^{PAF3}MP**
        - 1.2.4. Synthesis of **dC^{4TCO}MP**
        - 1.2.5. Synthesis of **dC^{2TCO}TP**
        - 1.2.6. Synthesis of **dC^{p4TCO}TP**
        - 1.2.7. Synthesis of **dC^{p2TCO}TP**
        - 1.2.8. Synthesis of **dC^{pBCN}TP**
    - 1.3. Structures of tetrazines used in this study – Inverse electron demands Diels-Alder reactions **(IEDDA)**
    - 1.4. IEDDA model reaction of **dC^{4TCO}MP** with 3,6-di-2-pyridyl-1,2,4,5-tetrazine **T5**
2. **Experimental section - biochemistry**
    - 2.1. List of sequences of primers, templates and oligonucleotides used in this study
    - 2.2. Enzymatic synthesis of TCO-, BCN-modified DNA
        - 2.2.1 Single incorporation of **dC^{4TCO}TP** or **dC^{2TCO}TP** or **dC^{p4TCO}TP** or **dC^{p2TCO}TP** or **dC^{pBCN}TP** using 19-mer template-Analytical scale
        - 2.2.2 Single incorporation of **dC^{4TCO}TP** or **dC^{2TCO}TP** or **dC^{p4TCO}TP** or **dC^{p2TCO}TP** or **dC^{pBCN}TP** using 19-mer template-Semi-preparative scale
    - 2.3. DB Streptavidin magneto-separation procedure
        - 2.3.1. MALDI data of **dC^{4TCO}-, dC^{2TCO}-**, **dC^{p4TCO}-**, **dC^{p2TCO}-**, **dC^{pBCN}**-modified oligonucleotides
3. **Fluorescence turn-on measurements and LC/MS characterizations of dC^{XTY}TP click products**

3.1. Fluorescence turn-on measurements and LC-MS characterizations of reactions between **dC^{4TCO}TP** or **dC^{2TCO}TP** with **T1** or **T2** and between **dC^{p4TCO}TP** or **dC^{p2TCO}TP** or **dC^{pBCN}TP** with **T1**, **T2** or **T3** tetrazines (**dC^{XTY}TP**)

4. Reaction of 19DNA_C^{4TCO} or 19DNA_C^{2TCO} or 19DNA_C^{p4TCO} or 19DNA_C^{p2TCO} or 19DNA_C^{pBCN} with tetrazines

4.1. Reaction of **19DNA_C^{X}** (**X** = **4TCO** or **2TCO** or **p4TCO** or **p2TCO** or **pBCN**) with **T1** or **T2** tetrazines and of **19DNA_C^{X}** (**X** = **p4TCO** or **pBCN**) with T4 tetrazine

4.2. Reaction of **19DNA_C^{X}** (**X** = **p4TCO** or **pBCN**) with **T3** tetrazine

4.3. Formation of tetrazine-ONs click products (**19ON_C^{XTY}**) - MALDI-TOF analysis

5. Oligonucleotide fluorescence turn-on measurement of 19ON_C^{p4TCO} or 19ON_C^{pBCN} with T1 or T3 tetrazines (**19ON_C^{XTY}**)

6. Labelling of DNA using dC^{p4TCO}TP or dC^{p2TCO}TP or dC^{pBCN}TP derivatives delivered to U-2 OS cells by SNTT1[6]

7. Copies of NMR spectra

8. Copies of MALDI-TOF mass spectra

9. References

1. Experimental section-organic chemistry part

General remarks for the synthetic part

^{1}H, ^{13}C and ^{31}P NMR spectra were acquired on a Bruker AVANCE IIIHD 600 (1H at 600.1 MHz, ^{13}C at 150.9 MHz), Bruker AVANCE IIIHD 500 (^{1}H at 500.0 MHz, ^{13}C at 125.7 MHz, ^{31}P at 202.4 MHz) and JEOL ECZR 500 (^{1}H at 500.2 MHz, ^{13}C at 125.8 MHz, ^{31}P at 202.5) spectrometers, as indicated. ^{1}H and ^{13}C resonances were fully assigned using H,H-COSY, H,H-ROESY, H,C-HSQC and H,C-HMBC techniques. All chemical shifts are quoted on the δ scale in ppm and referenced using residual ^{1}H solvent signal in ^{1}H NMR spectra ($\delta(CHCl_3)$ = 7.26 ppm; $\delta(CHD_2OD)$ = 3.31 ppm; and ^{13}C solvent signal in ^{13}C NMR spectra $\delta(CDCl_3)$ = 77.0 ppm; $\delta(CD_3OD)$ = 49.0 ppm). NMR spectra measured in D_2O were referenced to the signal of *t*-BuOH (10% v/v solution in D_2O, 1 drop) as the internal standard (1.24 ppm in ^{1}H, 32.43 ppm in ^{13}C). ^{31}P NMR spectra were referenced to H_3PO_4 signal (0 ppm) as the external standard. Coupling constants (*J*) are reported in Hz with the following splitting abbreviations: s = singlet, d= doublet, t = triplet, q = quartet, m = multiplet. High resolution mass spectra were measured on LTQ Orbitrap XL (Thermo Fisher Scientific) using Electrospray Ionization technique (ESI). Reactions were monitored by thin layer chromatography (TLC) on TLC Silica gel 60 F254 (Merck) and detected by UV (254 nm) or by solution of 4-anisaldehyde 3.6% v/v in ethanol and 10% v/v of sulphuric acid. Reactions were monitored by Advion Expression Compact Mass Spectrometer connected with Plate Express® TLC Plate Reader using ESI. High performance flash chromatography (HPFC) was performed with Biotage SP1 apparatus on DEAE SEPHADEX A-25 sodium form columns or with ISCO Combiflash Rf system on RediSep Rf Gold Silica Gel Disposable columns. Purification of nucleoside triphosphates was performed using HPLC (Waters modular HPLC system) on a column packed with 10 µm C18 reversed phase (Phenomenex, Luna C18 100 Å). Reactions were analysed by TLC using IPAV (isopropyl alcohol /NH_4OH /H_2O, ratio 11/7/2) as mobile phase. All materials were purchased from commercial suppliers and used without further purification unless otherwise stated. $POCl_3$ and $PO(OMe)_3$ were distilled prior to use. The water used in synthetic part was of HPLC quality. (*E*)-cyclooct-4-en-1-yl-2,5-dioxo-1-pyrrolidinyl ester carbonic acid (**4TCO-NHS-carbonate**), (*E*)-cyclooct-2-en-1-yl-2,5-dioxopyrrolidin-1-yl ester carbonic acid (**2TCO-NHS-carbonate**), (E)-1-(cyclooct-4-en-1-yloxy)-2,5-dioxopyrrolidin-1-yl-1-(oxo-5,8,11-trioxa-2-

azatetradecan)-14-oate (**4TCO-PEG3-NHS-ester**), (E)-1-(cyclooct-2-en-1-yloxy)-2,5-dioxopyrrolidin-1-yl-1-(oxo-5,8,11-trioxa-2-aza)-tetradecan-14-oate (**2TCO-PEG3-NHS-ester**) were purchased from Sirius Fine Chemicals SiChem GmbH. 2,5-dioxopyrrolidin-1-yl 1-(bicyclo[6.1.0]non-4-yn-9-yl)-3-oxo-2,7,10,13-tetraoxa-4-azahexadecan-16-oate (**endo-BCN-PEG3-NHS-ester**) was purchased from BroadPharm. Other chemicals were of analytical grade.

1.1 Synthetic scheme

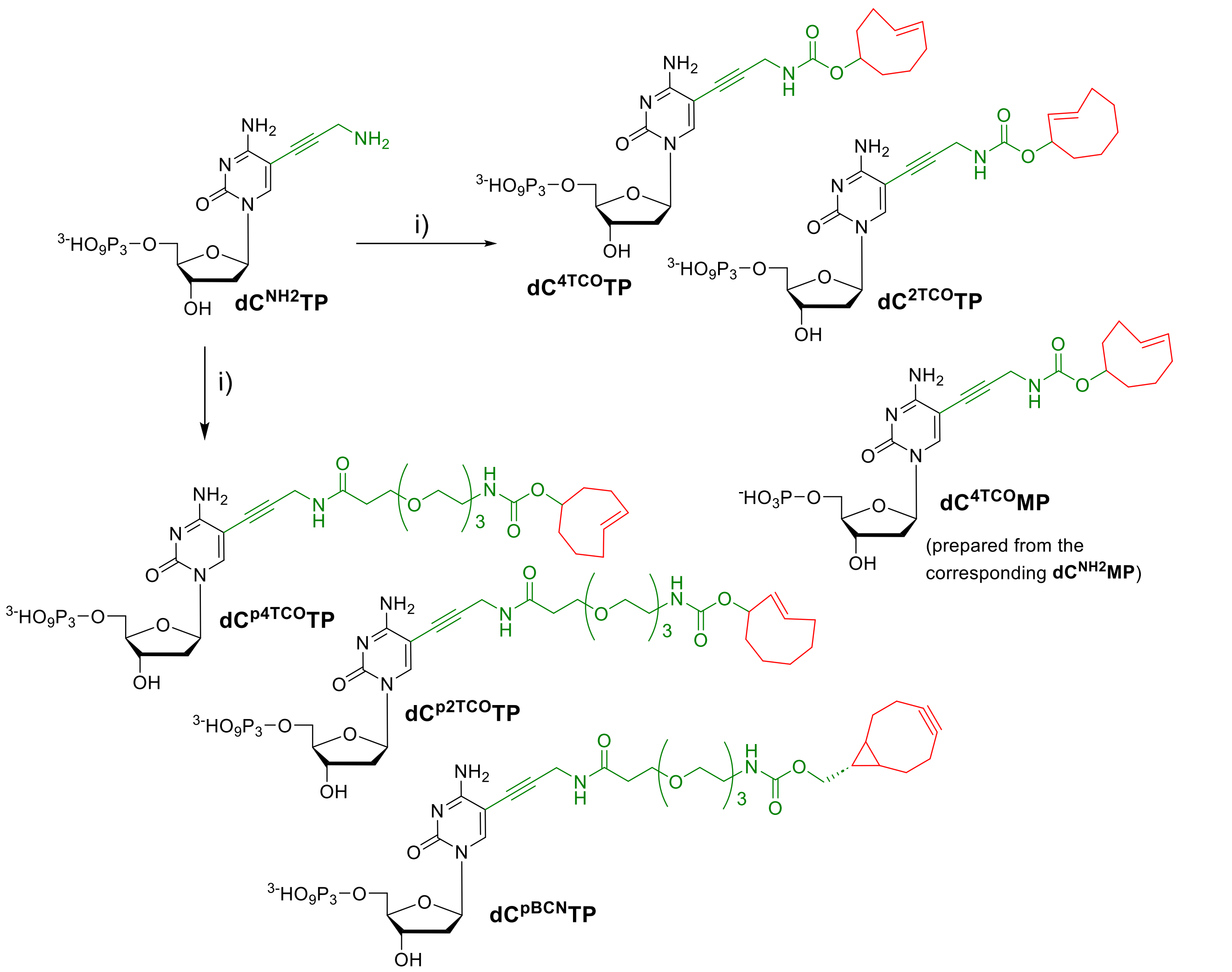

Scheme S1. Chemical synthesis of 4TCO-, 2TCO-, p4TCO-, p2TCO- and pBCN- modified-2'-deoxycitidine triphosphates and 4TCO- modified-2'-deoxycitidine monophosphate. Conditions i) 4TCO-NHS-carbonate or 2TCO-NHS-carbonate or 4TCO-PEG3-NHS-carbonate or 2TCO-PEG3-NHS-carbonate or endo-BCN-PEG3-NHS-ester in H_2O/TEAB 1 M, DMF, 55 °C, 4 h.

1.2. Synthesis of modified triphosphates and monophosphate (dC^{4TCO}MP)

1.2.1. 5-{[(*E*)-cyclooct-2-en-1-yl)oxy)carbonylamino]prop-1-yl}-2’-deoxycytidine-5-*O*-triphosphate triethylammonium salt (dC^{2TCO}TP)

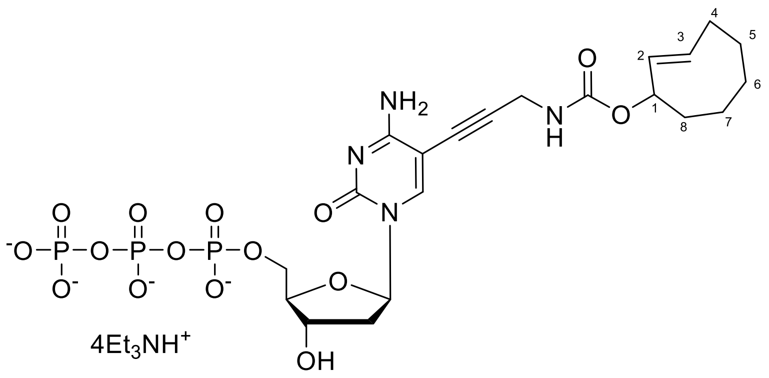

The freeze-dried **dC^{NH2}TP**[1,2] (5 mg, 7.44 µmol) was dissolved in 1 M TEAB buffer (pH 8, 500 µL), 2TCO-NHS-carbonate (6.25 g, 24.9 µmol) in DMF (416 µL) was added to the reaction mixture. The mixture was stirred at 55 ºC for 4 h and the solvent was removed *in vacuo*. The product was purified by RP-HPLC using a Phenomenex HPLC column (Luna 10 C8 100Å, 10×250 mm). Eluent A: 0.1 M TEAB buffer (pH 7.5), B: 60% acetonitrile in 0.1 M TEAB (pH 7.5). Gradient: 30% A - 70% buffer B in 19 min. Several co-distillations with water followed by freeze-drying gave the pure product **dC^{2TCO}TP** as yellowish powder (1.9 mg, 29% yield), Rf = 0.74 (IPAV).

Mixture of diastereoisomers ~3:2 (observable on signal of H-1′, C-1′,2′,3′)

^{1}H NMR (500.0 MHz, D_2O, ref(*t*BuOH) = 1.24 ppm): 0.81 (m, 1H, H-6b-cyclooct); 1.09 (bm, 1H, H-7b-cyclooct); 1.28 (t, 36H, J_{vic} = 7.3, C**H$_3$**CH$_2$N); 1.49 (bm, 1H, H-5b-cyclooct); 1.65 (bm, 1H, H-7a-cyclooct); 1.73 – 1.87 (m, 2H, H-6a,8b-cyclooct); 1.92 (bm, 1H, H-5a-cyclooct); 1.96 – 2.10 (m, 2H, H-4b,8a-cyclooct); 2.31 (dt, 1H, J_{gem} = 14.3, $J_{2'b,1'}$ = $J_{2'b,3'}$ = 6.6, H-2′b); 2.41 – 2.49 (m, 2H, H-2′a, H-4a-cyclooct); 3.21 (q, 24H, J_{vic} = 7.3, CH$_3$C**H$_2$**N); 4.15 (bs, 2H, CH$_2$N); 4.18 – 4.26 (m, 3H, H-4′,5′); 4.61 (m, 1H, H-3′); 5.21 (bs, 1H, H-1-cyclooct); 5.64 (dd, 1H, $J_{2,3}$ = 16.6, $J_{2,1}$ = 1,7, H-2-cyclooct); 5.89 (bm, 1H, H-3-cyclooct); 6.27, 6.28 (t, 1H, $J_{1',2'}$ = 6.6, H-1′); 8.17 (s, 1H, H-6).
^{13}C NMR (125.7 MHz, D_2O, ref(*t*BuOH) = 32.43 ppm): 11.06 (**C**H$_3$CH$_2$N); 26.38 (CH$_2$-7-cyclooct); 31.17 (CH$_2$-6-cyclooct); 33.68 (b, CH$_2$N); 38.21 (CH$_2$-4,5-cyclooct); 42.17, 42.22 (CH$_2$-2′); 42.64 (CH$_2$-8-cyclooct); 49.51 (CH$_3$**C**H$_2$N); 68.01 (b, CH$_2$-5′); 73.17, 73.22 (CH-3′); 75.81 (b, **C**≡CCH$_2$);

78.50 (b, CH-1-cyclooct); 88.36 (d, $J_{C,P}$ = 8.8, CH-4′); 89.09, 89.12 (CH-1′); 95.02 (b, C-5); 95.42 (b, C≡**C**CH_2); 133.98 (CH-2-cyclooct); 135.36 (b, CH-3-cyclooct); 147.61 (CH-6); 158.90 (C-2); 160.14 (b, NHCOO); 167.94 (C-4).

^{31}P NMR (202.4 MHz, D_2O): -22.55 (bdd, J = 21.2, 19.9, P_β); -11.31 (d, J = 19.9, P_α); -6.37 (d, J = 21.2, P_γ).

HR/MS (ESI-) for $C_{21}H_{30}O_{15}N_4P_3$: calculated 671.09260 $[M-H]^-$, found 671.09230 $[M-H]^-$.

1.2.2. 5-{[(*E*)-cyclooct-4-en-1-yl)oxy)carbonylamino]prop-1-yl}-2'-deoxycytidine-5-*O*-triphosphate triethylammonium salt (dC^{4TCO}TP)

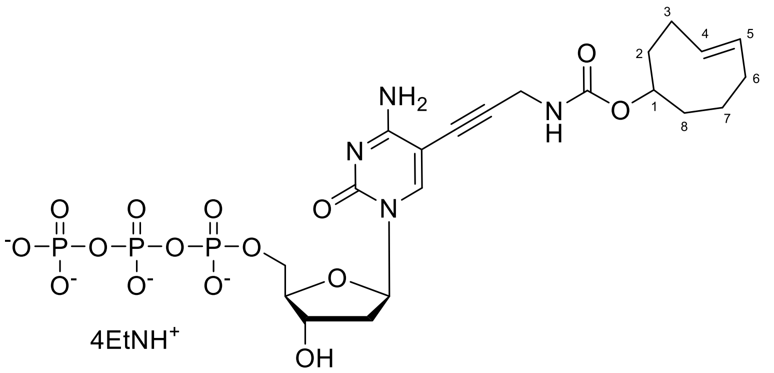

The freeze-dried **dC^{NH2}TP**[1,2] (3.7 mg, 7.44 µmol) was dissolved in 1 M TEAB buffer (pH 8, 370 µL), 4TCO-NHS-carbonate (4.62 mg, 18.40 µmol) in DMF (307 µL) was added to the reaction mixture. The mixture was stirred at 55 ºC for 4 h and the solvent was removed *in vacuo.* The product was purified by RP-HPLC using a Phenomenex HPLC column (Luna 10 C8 100Å, 10×250 mm). Eluent A: 0.1 M TEAB buffer (pH 7.5), B: 60% acetonitrile in 0.1 M TEAB (pH 7.5). Gradient: 30% A - 70% B in 20 min. Several co-distillations with water followed by freeze-drying gave the pure product **dC^{4TCO}TP** as yellowish powder (1.7 mg, 35% yield) Rf = 0.72 (IPAV).

^{1}H NMR (500.0 MHz, D_2O, ref(*t*BuOH) = 1.24 ppm): 1.28 (t, 18H, J_{vic} = 7.3, C**H**$_3$CH$_2$N); 1.51 (bm, 1H, H-7b-cyclooct); 1.58 – 1.70 (bm, 2H, H-2b,7a-cyclooct); 1.74 (bm, 1H, H-8b-cyclooct); 1.84 – 1.95 (bm, 2H, H-2a,8a-cyclooct); 2.09 (bm, 1H, H-3b-cyclooct); 2.13 – 2.23 (bm, 2H, H-6-cyclooct); 2.31 (dt, 1H, J_{gem} = 14.1, $J_{2'b,1'}$ = $J_{2'b,3'}$ = 6.5, H-2′b); 2.37 (bm, 1H, H-3a-cyclooct); 2.45 (ddd, 1H, J_{gem} = 14.1, $J_{2'a,1'}$ = 6.5, $J_{2'a,3'}$ = 4.0, H-2′a); 3.20 (q, 12H, J_{vic} = 7.3, CH$_3$C**H**$_2$N); 4.13 (bs, 2H, CH$_2$N); 4.19 – 4.23 (m, 3H, H-4′,5′); 4.60 (m, 1H, H-3′); 4.71 (m, 1H, H-1-cyclooct, partially overlapped with water signal); 5.66 – 5.78 (bm, 2H, H-4,5-cyclooct); 6.25 (t, 1H, $J_{1',2'}$ = 6.5, H-1′); 8.16 (s, 1H, H-6).

^{13}C NMR (125.7 MHz, D_2O, ref(*t*BuOH) = 32.43 ppm): 11.05 (**C**H_3CH_2N); 24.41 (CH_2-3-cyclooct); 27.22 (CH_2-7-cyclooct); 27.90 (CH_2-6-cyclooct); 33.69 (b, CH_2N); 36.17 (CH_2-2,8-cyclooct); 42.22 (CH_2-2′); 49.49 (CH_3**C**H_2N); 68.04 (d, $J_{C,P}$ = 5.5, CH_2-5′); 73.25 (CH-3′); 75.55 (**C**≡CCH_2); 80.67 (CH-1-cyclooct); 88.41 (d, $J_{C,P}$ = 8.9, CH-4′); 89.26 (CH-1′); 95.01 (C-5); 95.63 (C≡**C**CH_2); 132.86, 133.12 (CH-4,5-cyclooct); 147.83 (CH-6); 158.16 (C-2); 161.04 (NHCOO); 167.46(C-4).

^{31}P NMR (202.4 MHz, D_2O): -23.15 (bdd, J = 18.9, 16.0, P_β); -11.47 (bd, J = 18.9, P_α); -10.84 (d, J = 16.0 P_γ).

HR/MS (ESI-) for $C_{21}H_{30}O_{15}N_4P_3$: calculated 671.09260 $[M-H]^-$, found 671.09163 $[M-H]^-$.

1.2.3 5-(4-amino-2-oxo-5-(3-(2,2,2-trifluoroacetamido)prop-1-yn-1-yl)-2’-deoxycytidine-5-*O*-monophosphate triethylammonium salt (dC^{PAF3}MP)

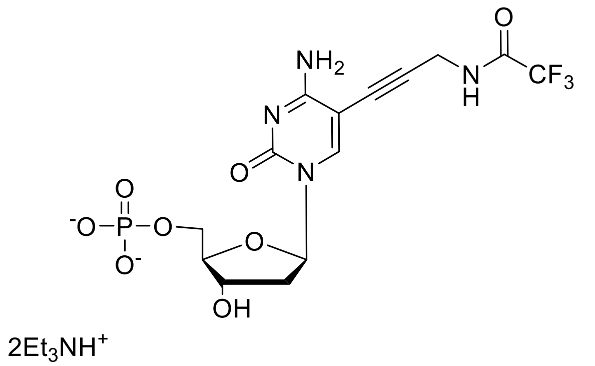

Compound **dC^{PACF3}** [2] (250 mg, 687.5 µmol) was dried at 25 °C overnight in vacuo. After cooling on ice, $PO(OMe)_3$ (2.5 mL) and $POCl_3$ (200 µL) were added under argon atmosphere. The reaction mixture was stirred for 2 h at 0 °C. The product was purified by SEPHADEX Na+ using H_2O/TEAB 2 M buffer as eluent and subsequentely by RP-HPLC using a Phenomenex HPLC column (Luna 10 C8 100Å, 10×250 mm). Several co-distillations with water followed by freeze-drying gave 80% pure product **dC^{PAF3}MP** as yellowish powder (yield 30%) and 20% of hydrolyzed product which is the desired product for the next step.

Contains ~ 20% of hydrolyzed product (without trifluoroacetyl)

^{1}H NMR (500.0 MHz, D_2O, ref(*t*BuOH) = 1.24 ppm): 1.27 (t, 18H, J_{vic} = 7.3, C**H$_3$**CH_2N); 2.30 (dt, 1H, J_{gem} = 13.9, $J_{2'b,1'}$ = $J_{2'b,3'}$ = 6.6, H-2′b); 2.43 (ddd, 1H, J_{gem} = 13.9, $J_{2'a,1'}$ = 6.2, $J_{2'a,3'}$ = 3.9, H-2′a); 3.18

(q, 12H, J_{vic} = 7.3, $CH_3C\mathbf{H_2}N$); 3.90 – 3.96 (m, 2H, H-5′); 4.16 (q, 1H, $J_{4',5'}$ = 4.9, $J_{4',5'}$ = 3.9, H-4′); 4.36(s, 2H, CH_2N); 4.52 (dt, 1H, $J_{3',2'}$ = 6.6, 3.9, $J_{3',4'}$ = 3.9, H-3′); 6.25 (dd, 1H, $J_{1',2'}$ = 6.6, 6.2, H-1′); 8.20 (s, 1H, H-6).

^{13}C NMR (125.7 MHz, D_2O, ref(*t*BuOH) = 32.43 ppm): 8.93 ($\mathbf{C}H_3CH_2N$); 30.81 (CH_2N); 39.90 (CH_2-2′); 47.32 ($CH_3\mathbf{C}H_2N$); 64.18 (d, $J_{C,P}$ = 4.5, CH_2-5′); 71.56 (CH-3′); 74.88 ($\mathbf{C}{\equiv}CCH_2$); 86.87 (d, $J_{C,P}$ = 8.3, CH-4′); 86.96 (CH-1′); 90.67 ($C{\equiv}\mathbf{C}CH_2$); 92.42 (C-5); 116.44 (q, $J_{C,F}$ = 286.1, $\mathbf{C}F_3CO$); 146.02 (CH-6); 156.75 (C-2); 159.46 (q, $J_{C,F}$ = 38.6, $CF_3\mathbf{C}O$); 165.78 (C-4).

^{31}P{^{1}H} NMR (202.4 MHz, D_2O): 4.67.

^{19}F NMR (470.4 MHz, D_2O): -72.06.

HR/MS (ESI-) for $C_{14}H_{15}O_8N_4F_3P$: calculated 455.05851 $[M-H]^-$, found 455.05884 $[M-H]^-$

1.2.4 5-[3-(Aminoprop-1-yn-1-yl)]-2’-deoxycytidine 5-*O*-monophosphate triethylammonium salt (dC^{NH2}MP)

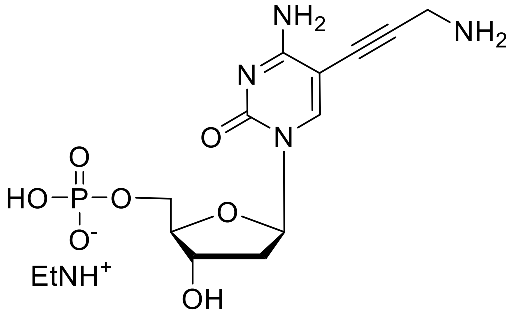

dC^{PAF3}MP (116 mg, 255 μmol) was dissolved NH_4OH aq. (10 mL) and stirred at room temperature for 24 h. The product was purified by RP-HPLC using a Phenomenex HPLC column (Luna 10 C8 100Å, 10×250 mm). Several co-distillations with water followed by freeze-drying gave the pure product **dC^{NH2}MP** as white powder (74 mg, 81%).

^{1}H NMR (500.0 MHz, D_2O, ref(*t*BuOH) = 1.24 ppm): 1.28 (t, 9H, J_{vic} = 7.3, $C\mathbf{H_3}CH_2N$); 2.32 (dt, 1H, J_{gem} = 14.0, $J_{2'b,1'}$ = $J_{2'b,3'}$ = 6.2, H-2′b); 2.48 (ddd, 1H, J_{gem} = 14.0, $J_{2'a,1'}$ = 6.5, $J_{2'a,3'}$ = 4.7, H-2′a); 3.20 (q, 6H, J_{vic} = 7.3, $CH_3C\mathbf{H_2}N$); 4.03 (d, 1H, J_{gem} = 17.0, $CH_a\mathbf{H_b}NH_2$); 4.04 (ddd, 1H, J_{gem} = 11.7, $J_{H,P}$ = 4.8, $J_{5'b,4'}$ = 3.1, H-5′b); 4.06 (d, 1H, J_{gem} = 17.0, $C\mathbf{H_a}H_bNH_2$); 4.14 (ddd, 1H, J_{gem} = 11.7, $J_{H,P}$ = 4.1, $J_{5'a,4'}$ = 2.6, H-5′a); 4.19 (dddd, 1H, $J_{4',3'}$ = 4.3, $J_{4',5'}$ = 3.1, 2.6, $J_{H,P}$ = 2.1, H-4′); 4.55 (ddd, 1H, $J_{3',2'}$ = 6.2, 4.7, $J_{3',4'}$ = 4.3, H-4′); 6.25 (dd, 1H, $J_{1',2'}$ = 6.5, 6.2, H-1′); 8.44 (s, 1H, H-6).

^{13}C NMR (125.7 MHz, D_2O, ref(*t*BuOH) = 32.43 ppm): 11.06 (**C**H_3CH_2N); 32.66 (CH_2NH_2); 42.89 (CH_2-2′); 49.51 (CH_3**C**H_2N); 66,62 (d, $J_{C,P}$ = 4.9, CH_2-5′); 72.71 (CH-3′); 80.71 (**C**≡CCH_2); 88.71 (d, $J_{C,P}$ = 9.0, CH-4′); 89.34 (CH-1′); 89.43 (C≡**C**CH_2); 93.84 (C-5); 149.30 (CH-6); 158.79 (C-2); 167.53 (C-4).

^{31}P NMR (202.4 MHz, D_2O): 1.57.

HR/MS (ESI-) for $C_{12}H_{16}N_4O_7P^-$: calculated 359.07621 [M-H]$^-$; found 359.07565 [M-H]$^-$.

1.2.5. 5-{[(*E*)-Cyclooct-4-en-1-yl)oxy)carbonylamino]prop-1-yl}-2'-deoxycytidine-5-*O*-triphosphate triethylammonium salt (dC^{4TCO}MP)

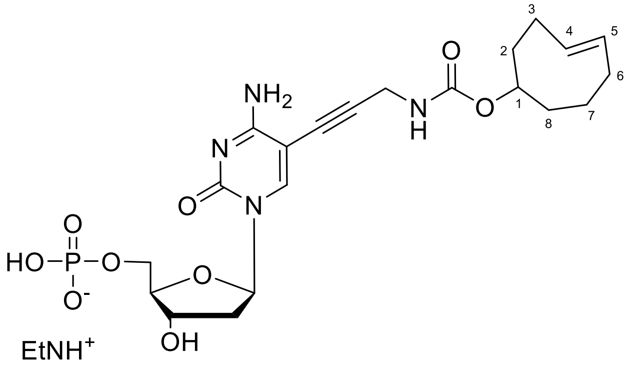

The freeze-dried **dC^{NH2}MP** (29.2 mg, 80.7 µmol) was dissolved in 1 M TEAB buffer (pH 8, 3 mL), 4TCO-NHS-carbonate (30 mg, 112.3 µmol) in DMF (3 mL) was added to the reaction mixture. The mixture was stirred at 55 ºC for 4 h and the solvent was removed *in vacuo*. The product was purified by RP-HPLC using a Phenomenex HPLC column (Luna 10 C8 100Å, 10×250 mm). Eluent A: 0.1 M TEAB buffer (pH 7.5), B: 60% acetonitrile in 0.1 M TEAB (pH 7.5). Several co-distillations with water followed by freeze-drying gave the pure product **dC^{4TCO}MP** as yellowish powder (20 mg, 48% yield) Rf = 0.60 (IPAV).

^1H NMR (500.0 MHz, D_2O, ref(*t*BuOH ext) = 1.24 ppm): 1.28 (t, 9H, J_{vic} = 7.3, C**H₃**CH_2N); 1.51 (m, 1H, H-7b-cyclooct); 1.59 – 1.70 (bm, 2H, H-2b,7a-cyclooct); 1.73 (bm, 1H, H-8b-cyclooct); 1.84 – 1.93 (bm, 2H, H-2a,8a-cyclooct); 2.09 (m, 1H, H-3b-cyclooct); 2.13 – 2.22 (m, 2H, H-6-cyclooct); 2.29 (ddd, 1H, J_{gem} = 14.2, $J_{2'b,1'}$ = 6.8, $J_{2'b,3'}$ = 6.3, H-2′b); 2.37 (bm, 1H, H-3a-cyclooct); 2.46 (ddd, 1H, J_{gem} = 14.2, $J_{2'a,1'}$ = 6.4, $J_{2'a,3'}$ = 3.9, H-2′a); 3.20 (q, 6H, J_{vic} = 7.3, CH_3C**H_2**N); 4.05 (ddd, 1H, J_{gem} = 11.6, $J_{H,P}$ = 5.7, $J_{5'b,4'}$ = 4.1, H-5′b); 4.09 (ddd, 1H, J_{gem} = 11.6, $J_{H,P}$ = 4.9, $J_{5'a,4'}$ = 3.5, H-5′a); 4.12 (bs, 2H, CH_2N); 4.20 (dtd, 1H, $J_{4',5'}$ = 4.1, 3.5, $J_{4',3'}$ = 3.5, $J_{H,P}$ = 1.7, H-4′); 4.52 (ddd, 1H, $J_{3',2'}$ = 6.3, 3.9,

$J_{3',4'}$ = 3.5, H-3′); 4.71 (m, 1H, H-1-cyclooct, overlapped with water signal); 5.65 – 5.78 (bm, 2H, H-4,5-cyclooct); 6.25 (dd, 1H, $J_{1',2'}$ = 6.8, 6.4, H-1′); 8.15 (s, 1H, H-6).

^{13}C NMR (125.7 MHz, D_2O, ref(*t*BuOH ext) = 32.43 ppm): 11.06 (**C**H_3CH_2N); 24.41 (CH_2-3-cyclooct); 27.21 (CH_2-7-cyclooct); 27.89 (CH_2-6-cyclooct); 33.64 (b, CH_2N); 36.05 (b, CH_2-8-cyclooct); 36.19 (CH_2-2-cyclooct); 42.46 (CH_2-2′); 49.51 (CH_3**C**H_2N); 67.29 (d, $J_{C,P}$ = 4.9, CH_2-5′); 73.50 (CH-3′); 75.75 (**C**≡CCH_2); 80.68 (CH-1-cyclooct); 88.53 (d, $J_{C,P}$ = 8.6, CH-4′); 89.39 (CH-1′); 94.96 (C-5); 95.41 (b, C≡**C**CH_2); 132.86 (CH-4-cyclooct); 133.12 (CH-5-cyclooct); 147.66 (CH-6); 158.62 (C-2); 161.03 (b, NHCOO); 167.78 (C-4).

^{31}P NMR (202.4 MHz, D_2O): 0.32.

HR/MS (ESI-) for $C_{21}H_{28}N_4O_9P^-$: calculated 511.17 [M-H]$^-$; found 511.2 [M-H]$^-$.

1.2.6. 5-{[1-((*E*)-Cyclooct-4-en-1-yl)oxy]-1,14-dioxo-5,8,11-trioxa-2,15-diazaoctadec-17-yn-18-yl}-2’-deoxycytidine-5-*O*-triphosphate triethylammonium salt (dC^{p4TCO}TP)

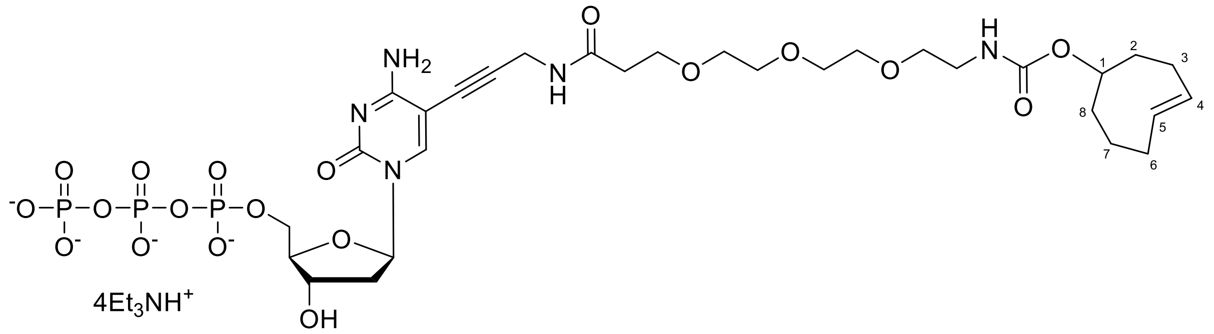

The freeze-dried **dC^{NH2}TP**[1,2] (4.4 mg, 8.46 µmol) was dissolved in 1 M TEAB buffer (pH 8, 352 µL), 4TCO-PEG3-NHS ester (5.71 mg, 12.13 µmol) in DMF (285 µL) was added to the reaction mixture. The mixture was stirred at 55 ºC for 4 h and the solvent was removed *in vacuo*. The product was purified by RP-HPLC using a Phenomenex HPLC column (Luna 10 C8 100Å, 10×250 mm). Eluent A: 0.1 M TEAB buffer (pH 7.5), B: 60% acetonitrile in 0.1 M TEAB (pH 7.5). Gradient: 30% A-70% B in

32 min. Several co-distillations with water followed by freeze-drying gave the pure product **dC^{p4TCO}TP** as yellowish powder (2.70 mg 37% yield), Rf = 0.70 (IPAV).

^{1}H NMR (600.1 MHz, D_2O, ref(*t*BuOH) = 1.24 ppm): 1.24 (t, 36H, J_{vic} = 7.3, C**H$_3$**CH$_2$N); 1.29 (bm, 1H, H-8b-cyclooct); 1.43 (bm, 1H, H-7b-cyclooct); 1.73 (bm, 1H, H-2b-cyclooct); 1.78 (bm, 1H, H-7a-cyclooct); 1.86 (bm, 1H, H-6b-cyclooct); 2.10 (bm, 1H, H-3b-cyclooct); 2.17 – 2.26 (m, 3H, H-2a,8a,6a-cyclooct); 2.30 (dt, 1H, J_{gem} = 14.0, $J_{2'b,1'}$ = $J_{2'b,3'}$ = 6.5, H-2′b); 2.31 (m, 1H, H-3a-cyclooct); 2.44 (ddd, 1H, J_{gem} = 14.0, $J_{2'a,1'}$ = 6.5, $J_{2'a,3'}$ = 4.7, H-2′a); 2.54 – 2.63 (m, 2H, COC**H$_2$**CH$_2$O); 3.09 (q, 24H, J_{vic} = 7.3, CH$_3$C**H$_2$**N); 3.27 – 3.36 (bm, 2H, NHCOC**H$_2$**CH$_2$O); 3.57 – 3.62 (bm, 2H, NHCOCH$_2$C**H$_2$**O); 3.63 – 3.70 (m, 8H, OC**H$_2$**C**H$_2$**O); 3.77 – 3.87 (m, 2H, COCH$_2$C**H$_2$**O); 4.17 – 4.30 (m, 5H, H-4′,5′, CH$_2$N); 4.64 (dt, 1H, $J_{3',2'}$ = 6.5, 4.7, $J_{3',4'}$ = 4.7, H-3′); 4.76 (m, 1H, H-1-cyclooct); 5.60 – 5.69 (bm, 2H, H-4,5-cyclooct); 6.25 (t, 1H, $J_{1',2'}$ = 6.5, H-1′); 8.21 (s, 1H, H-6).

^{13}C NMR (150.9 MHz, D_2O, ref(*t*BuOH) = 30.29 ppm): 8.92 (**C**H$_3$CH$_2$N); 27.95 (CH$_2$-7-cyclooct); 29.93 (CH$_2$-3-cyclooct); 30.11 (CH$_2$N); 32.46 (CH$_2$-8-cyclooct); 34.18 (CH$_2$-6-cyclooct); 36.35 (CO**C**H$_2$CH$_2$O); 39.71 (CH$_2$-2′); 40.38 (N**C**H$_2$CH$_2$O); 40.67 (CH$_2$-2-cyclooct); 46.85 (CH$_3$**C**H$_2$N); 65.18 (d, $J_{C,P}$ = 5.6, CH$_2$-5′); 67.10 (COCH$_2$**C**H$_2$O); 69.80 (NCH$_2$**C**H$_2$O); 69.87, 69.98 (O**C**H$_2$**C**H$_2$O); 70.02 (CH-1-cyclooct); 70.04, 70.09 (O**C**H$_2$**C**H$_2$O); 71.74 (CH-3′); 73.54 (**C**≡CCH$_2$); 86.01 (d, $J_{C,P}$ = 9.0, CH-4′); 86.48 (CH-1′); 92.29 (C≡**C**CH$_2$); 92.55 (C-5); 132.85, 136.23 (CH-4,5-cyclooct); 145.42 (CH-6); 156.39 (C-2); 158.924 (NHCOO); 165.49 (C-4); 174.53 (CONH).

^{31}P NMR (202.4 MHz, D_2O): -22.59 (bdd, *J* = 21.2, 20.0, P$_\beta$); -11.40 (d, *J* = 20.0, P$_\alpha$); -6.37 (d, *J* = 21.2, P$_\gamma$).

HR/MS (ESI-) for $C_{30}H_{48}N_5O19P_3$: calculated 874.20836 [M-H]$^-$, found 874.20807 [M-H]$^-$.

1.2.7. 5-{[1-((*E*)-Cyclooct-2-en-1-yl)oxy]-1,14-dioxo-5,8,11-trioxa-2,15-diazaoctadec-17-yn-18-yl}-2’-deoxycytidine-5-*O*-triphosphate triethylammonium salt (dC^{p2TCO}TP)

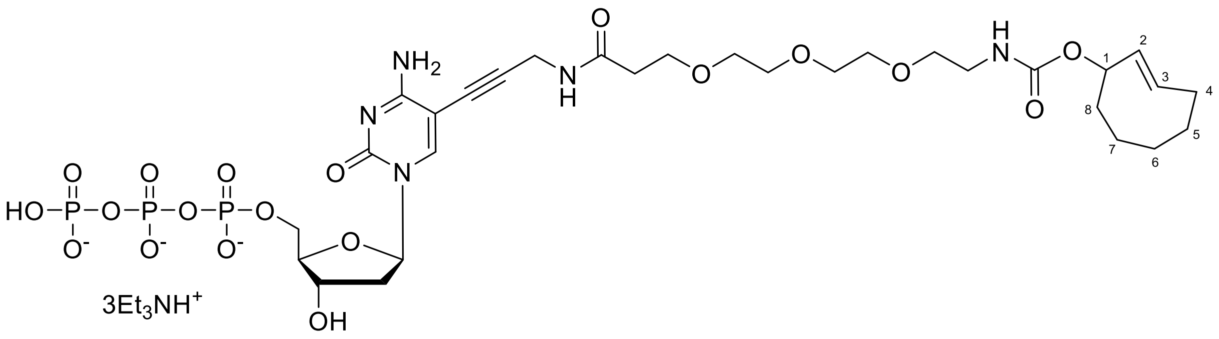

The freeze-dried **dC^{NH2}TP**[1,2] (7 mg, 13.46 µmol) was dissolved in 1 M TEAB buffer (pH 8, 560 µL), 2TCO-PEG3-NHS ester (9.07 mg, 21.59 µmol) in DMF (520 µL) was added to the reaction mixture. The mixture was stirred at 55 ºC for 4 h and the solvent was removed *in vacuo*. The products were purified by RP-HPLC using a Phenomenex HPLC column (Luna 10 C8 100Å, 10×250 mm). Eluent A: 0.1 M TEAB buffer (pH 7.5), B: 60% acetonitrile in 0.1 M TEAB (pH 7.5). 30% A-70% B in 32 min. Several co-distillations with water followed by freeze-drying gave the pure product **dC^{P2TCO}TP** as yellowish powder (4.15 mg, 35%), Rf = 0.68 (IPAV).

^{1}H NMR (500.0 MHz, D_2O, ref(*t*BuOH) = 1.24 ppm): 0.78 (bm, 1H, H-6b-cyclooct); 1.04 (bm, 1H, H-7b-cyclooct); 1.28 (t, 27H, J_{vic} = 7.3, C**H3**CH2N); 1.46 (bm, 1H, H-5b-cyclooct); 1.63 (bm, 1H, H-7a-cyclooct); 1.75 (bm, 1H, H-8b-cyclooct); 1.82 (bm, 1H, H-6a-cyclooct); 1.93 (bm, 1H, H-5a-cyclooct); 1.96 – 2.06 (m, 2H, H-4b,8a-cyclooct); 2.30 (dt, 1H, J_{gem} = 14.1, $J_{2'b,1'}$ = $J_{2'b,3'}$ = 6.5, H-2′b); 2.41 (m, 1H, H-4a-cyclooct); 2.45 (ddd, 1H, J_{gem} = 14.1, $J_{2'a,1'}$ = 6.5, $J_{2'a,3'}$ = 4.1, H-2′a); 2.53 – 2.63 (m, 2H, COC**H2**CH2O); 3.20 (q, 18H, J_{vic} = 7.3, CH3C**H2**N); 3.29 – 3.34 (bm, 2H, NHCOC**H2**CH2O); 3.59 (bt, 2H, J_{vic} = 5.3, NHCOCH2C**H2**O); 3.64 – 3.70 (m, 8H, OC**H2**C**H2**O); 3.78 – 3.85 (m, 2H, COCH2C**H2**O); 4.16 – 4.27 (m, 5H, H-4′,5′, CH2N); 4.63 (dd, 1H, $J_{3',2'}$ = 6.5, 4.1, $J_{3',4'}$ = 3.1, H-3′); 5.15 (bm, 1H, H-1-cyclooct); 5.62 (dd, 1H, $J_{2,3}$ = 16.5, $J_{2,1}$ = 2.0, H-2-cyclooct); 5.87 (m, 1H, H-3-cyclooct); 6.26 (t, 1H, $J_{1',2'}$ = 6.5, H-1′); 8.19 (s, 1H, H-6).

^{13}C NMR (125.7 MHz, D_2O, ref(*t*BuOH) = 30.29 ppm): 8.92 (**C**H3CH2N); 24.27 (CH2-7-cyclooct); 29.04 (CH2-6-cyclooct); 30.41 (b, CH2N); 36.09, 36.21 (CH2-4,5-cyclooct); 36.64 (CO**C**H2CH2O); 40.15 (CH2-2′); 40.54 (CH2-8-cyclooct); 40.67 (N**C**H2CH2O); 47.35 (CH3**C**H2N); 65.84 (d, $J_{C,P}$ = 5.6, CH2-5′); 67.39 (COCH2**C**H2O); 69.99, 70.13, 70.26, 70.32, 70.35 (O**C**H2**C**H2O, NCH2**C**H2O); 70.99 (CH-3′); 73.77 (**C**≡CCH2); 75.83 (CH-1-cyclooct); 86.24 (d, $J_{C,P}$ = 9.0, CH-4′); 86.98 (CH-1′); 92.57

(C≡**C**CH_2); 92.88 (C-5); 132.06 (CH-2-cyclooct); 132.98 (CH-3-cyclooct); 145.63 (CH-6); 156.62 (C-2); 158.92 (NHCOO); 165.72 (C-4); 174.83 (CONH).

^{31}P NMR (202.4 MHz, D_2O): -23.25 (bdd, J = 20.1, 19.5, P_β); -11.56 (d, J = 20.1, P_α); -10.75 (d, J = 19.5, P_γ).

HR/MS (ESI-) for $C_{30}H_{48}N_5O_{19}P_3$: calculated 874.20836 $[M-H]^-$, found 874.20715 $[M-H]^-$.

1.2.8. Synthesis of 5-{[(bicyclo[6.1.0]non-4-yn-9-yl)-3,16-dioxo-2,7,10,13-tetraoxa-4,17-diazaicos-19-yn-20-yl)-2'-deoxycytidine-5-*O*-triphosphate triethylammonium salt (dC^{pBCN}TP)

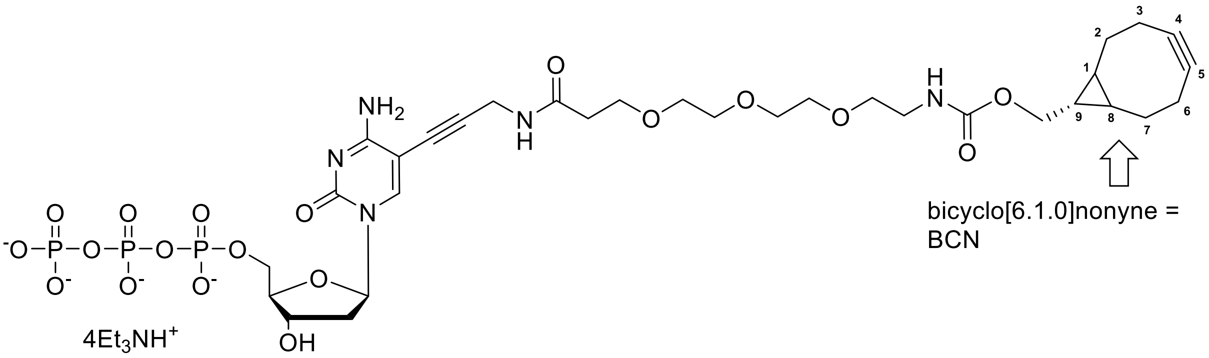

The freeze-dried **dC^{NH2}TP**[1,2] (4.6 mg, 2.29 µmol) was dissolved in 0.5 M TEAB buffer (pH 7.5, 319 µL), endo-BCN-PEG3-NHS ester (6 mg, 3.81 µmol) in DMF (258 µL) was added to the reaction mixture. The mixture was stirred at 55 ºC for 4 h and the solvent was removed *in vacuo*. The products were purified by RP-HPLC using a Phenomenex HPLC column (Luna 10 C8 100Å, 10×250 mm). Eluent A: 0.1 M TEAB buffer (pH 7.5), B: 60% acetonitrile in 0.1 M TEAB (pH 7.5). 30% A-70% B in 32 min. Several co-distillations with water followed by freeze-drying gave the pure product **dC^{pBCN}TP** as yellowish powder (1.36 mg, 20%), Rf = 0.68 (IPAV).

^{1}H NMR (600.1 MHz, D_2O, ref(*t*BuOH) = 1.24 ppm): 0.92 – 0.98 (m, 2H, H-1,8-BCN); 1.25 (t, 36H, J_{vic} = 7.3, C**H$_3$**CH_2N); 1.37 (m, 1H, H-9-BCN); 1.49 – 1.60 (bm, 2H, H-2b,7b-BCN); 2.15 – 2.29 (bm, 6H, H2a,3,6,7a-BCN); 2.31 (dt, 1H, J_{gem} = 14.0, $J_{2'b,1'}$ = $J_{2'b,3'}$ = 6.5, H-2′b); 2.44 (ddd, 1H, J_{gem} = 14.0, $J_{2'a,1'}$ = 6.5, $J_{2'a,3'}$ = 4.6, H-2′a); 2.54 – 2.63 (m, 2H, COC**H$_2$**CH_2O); 3.12 (q, 24H, J_{vic} = 7.3, CH_3C**H$_2$**N); 3.29 – 3.33 (bm, 2H, NHCOC**H$_2$**CH_2O); 3.57 – 3.60 (bm, 2H, NHCOCH_2C**H$_2$**O); 3.64 – 3.69 (m, 8H,

OCH_2CH_2O); 3.78 – 3.85 (m, 2H, COCH_2CH_2O); 4.14 – 4.24 (m, 6H, H-4′,5′b, CH_2N, CH_2O); 4.27 (ddd, 1H, J_{gem} = 11.5, $J_{H,P}$ = 6.3, $J_{5'a,4'}$ = 3.8, H-5′a); 4.63 (dt, 1H, $J_{3',2'}$ = 6.5, 4.6, $J_{3',4'}$ = 4.6, H-3′); 6.24 (t, 1H, $J_{1',2'}$ = 6.5, H-1′); 8.21 (s, 1H, H-6).

^{13}C NMR (150.9 MHz, D_2O, ref(*t*BuOH) = 30.29 ppm): 9.11 (**C**H_3CH_2N); 17.88 (CH-9-BCN); 20.41 (CH-1,8-BCN); 21.43 (CH_2-3,6-BCN); 29.24 (CH_2-2,7-BCN); 30.38 (CH_2N); 36.65 (CO**C**H_2CH_2O); 40.03 (CH_2-2′); 40.80 (N**C**H_2CH_2O); 47.22 (CH_3**C**H_2N); 64.46 (CH_2O); 66.47 (d, $J_{C,P}$ = 5.4, CH_2-5′); 67.39 (COCH_2**C**H_2O); 69.99 (NCH_2**C**H_2O); 70.12, 70.24 (O**C**H_2**C**H_2O); 70.31 (CH-3′); 70.33 (O**C**H_2CH_2O); 73.85 (**C**≡CCH_2); 86.28 (d, $J_{C,P}$ = 9.0, CH-4′); 86.81 (CH-1′); 92.59 (C≡**C**CH_2); 92.81 (C-5); 100.92 (C-4,5-BCN); 145.73 (CH-6); 156.68 (C-2); 159.51 (NHCOO); 165.78 (C-4); 174.77 (CONH).

^{31}P NMR (202.4 MHz, D_2O): -22.42 (dd, *J* = 21.0, 19,9, P_β); -11.38 (d, *J* = 19,9, P_α); -6.33 (d, *J* = 21.0, P_γ).

HR/MS (ESI-) for $C_{32}H_{48}N5O_{19}P_3$: calculated 898.20836 [M-H]$^-$, found 898.20729 [M-H]$^-$.

1.3. Structures of tetrazines used in this study – Inverse electron demands Diels-Alder reactions (IEDDA)

7-(Azetidin-1-yl)-4-methyl-3-(5-(6-pyrimidin-2-yl)-1,2,4,5-tetrazin-3-yl)pyridin-3-yl)-2*H*-chromen-2-one (**T1**)[3,4], 7-(Azetidin-1-yl)-4-methyl-3-(3-(6-(pyridin-4-yl)-1,2,4,5-tetrazin-3-yl)phenyl)-2*H*-chromen-2-one (**T2**)[3,4], N,N-dimethyl-6-(6-methyl-1,2,4,5-tetrazin-3-yl)acridin-3-amine (**T3**)[6], 2-(6-(dimethylamino)-3-(dimethyliminio)-3H-xanthen-9-yl)-5-((3-(4-(6-(pyrimidin-2-yl)-1,2,4,5-tetrazin-3-yl)benzamido)propyl)carbamoyl)benzoate or pyrimidyl-tetrazine-Cy3 (**T4**) and 3,6-di-2-pyridyl-1,2,4,5-tetrazine (**T5**).

T1 and **T2** (ca. 5 mg) were freshly purified by RP HPLC on Arion C18 semipreparative column using a gradient of CH_3CN in H_2O (30 to 95% over 25 min) containing 0.1% TFA. Fractions containing the product (LC-MS analysis) were pooled and lyophilized. The products were analyzed using PDA, MS and fluorescence detector (Ex = 380 nm, Em = 475 nm) (Figure S1B). The analysis revealed that **T2** contains fluorescent impurities, which were not removed during the purification step. In contrast, **T1** can be purified to such an extent that no additional fluorescent impurities are present in the final product. Accordingly, **T1** has much lower background than has **T2**.

A)

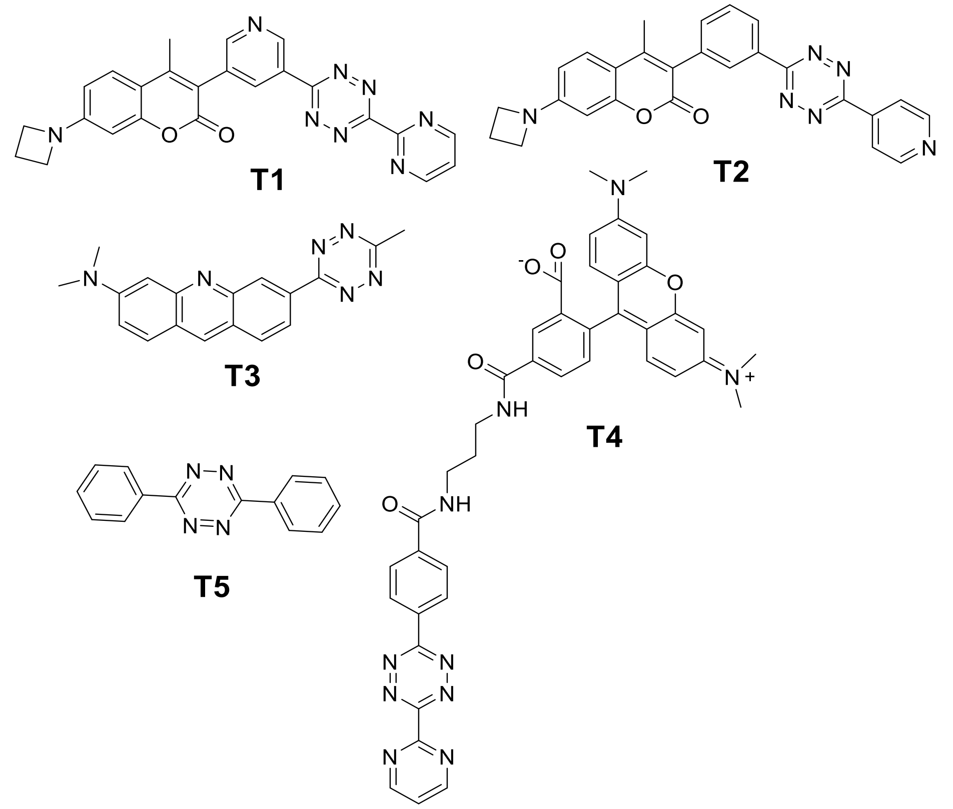

B)

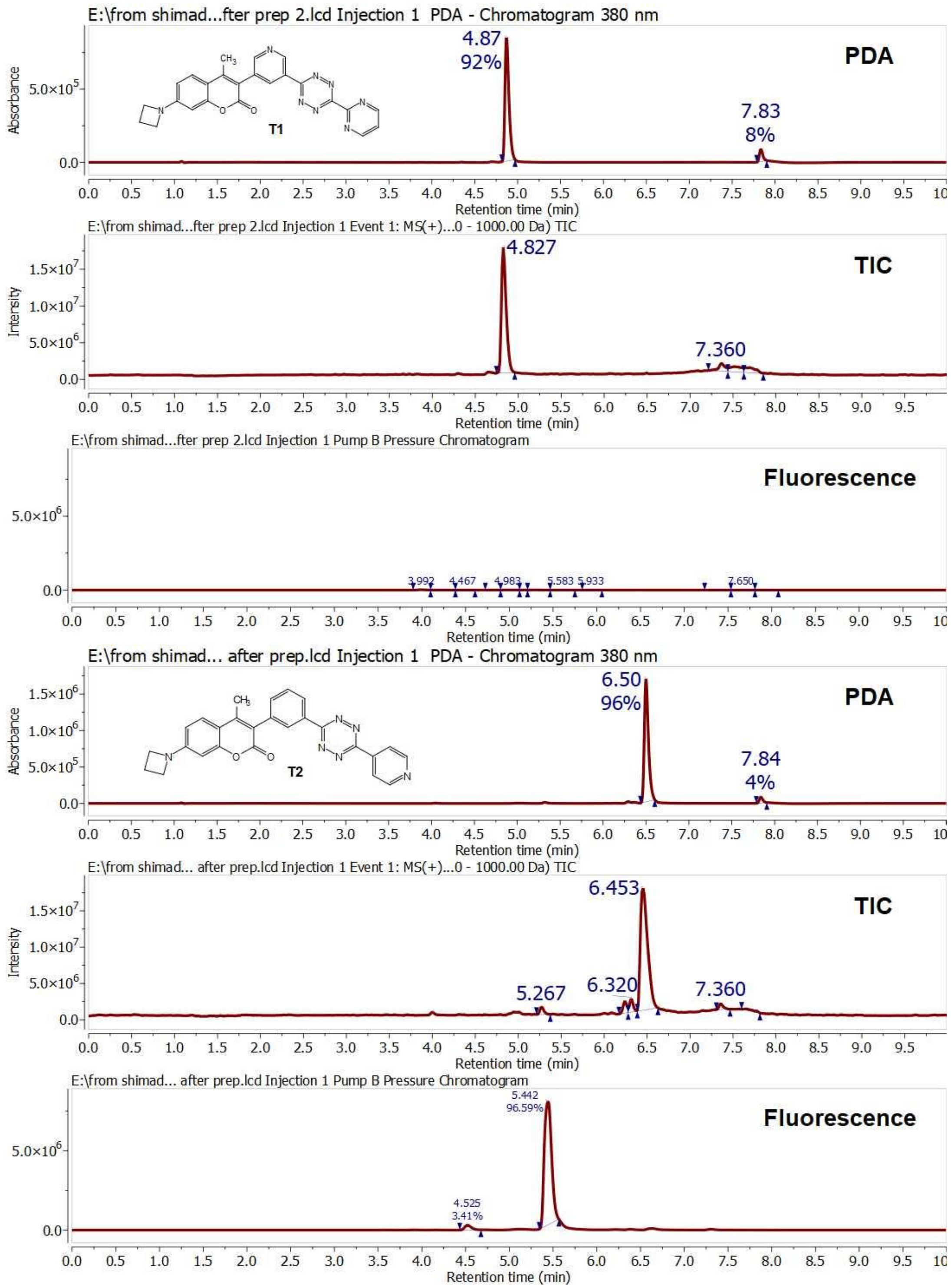

Figure S1. A) Structures of tetrazines used in this study[3,4,6], B) LC-MS analysis of freshly purified (semipreparative HPLC) **T1** and **T2**, which shows that residual impurities are present in **T2** even after purification. These are visible especially in the chromatogram that shows the fluorescence signal.

1.4. IEDDA model reaction of dC^{4TCO}MP with 3,6-di-2-pyridyl-1,2,4,5-tetrazine T5

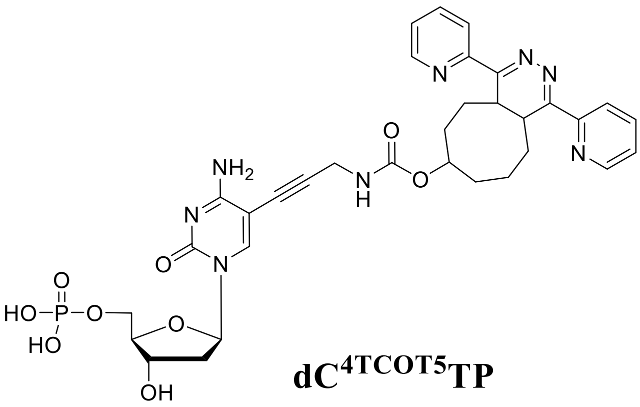

dC^{4TCOT5}TP

The freeze-dried **dC^{4TCO}MP** (20 mg, 39 µmol) was dissolved in water (3 mL).
3,6-di-2-pyridyl-1,2,4,5-tetrazine or **T5** tetrazine (30 mg, 127 µmol) was dissolved in 2 mL ACN/H_2O (3:1). The mixture was stirred at 37 ºC for 2 h and the solvent was removed in vacuo.
The product was purified by RP-HPLC using a Phenomenex HPLC column (Luna 10 C8 100Å, 10×250 mm). Eluent A: 0.1 M TEAB buffer (pH 7.5), B: 60% acetonitrile in 0.1 M TEAB (pH 7.5). The complicated chromatogram gave mixture of diasteroisomers of the desired product (3:7, DCM/MeOH). MS and NMR confirmed formation of the desired triphosphate-tetrazine conjugate **dC^{4TCOT5}MP**, however, complete assignment of ^{1}H and ^{13}C NMR spectra was complicated by several factors. First, concentration of the sample was not sufficient for direct observation of ^{13}C NMR spectrum. Instead, 2D correlation experiments such as H,C-HSQC and H,C-HMBC were used to identify structural fragments of the product in 1H NMR spectrum, such as 2-deoxyribose, nucleobase, linker, cyclooctane ring and pyridyl moiety (see Fig. S2). Second, based on reaction mechanism,[1] click reaction of substituted trans-cyclooctene with tetrazine provides a mixture of several isomers that contributes to complexity of NMR spectra as well.

HR/MS (ESI-) for $C_{33}H_{37}N_8O_9P$ calculated: 719.23483 $[M-H]^-$, found 719.23468 $[M-H]^-$.

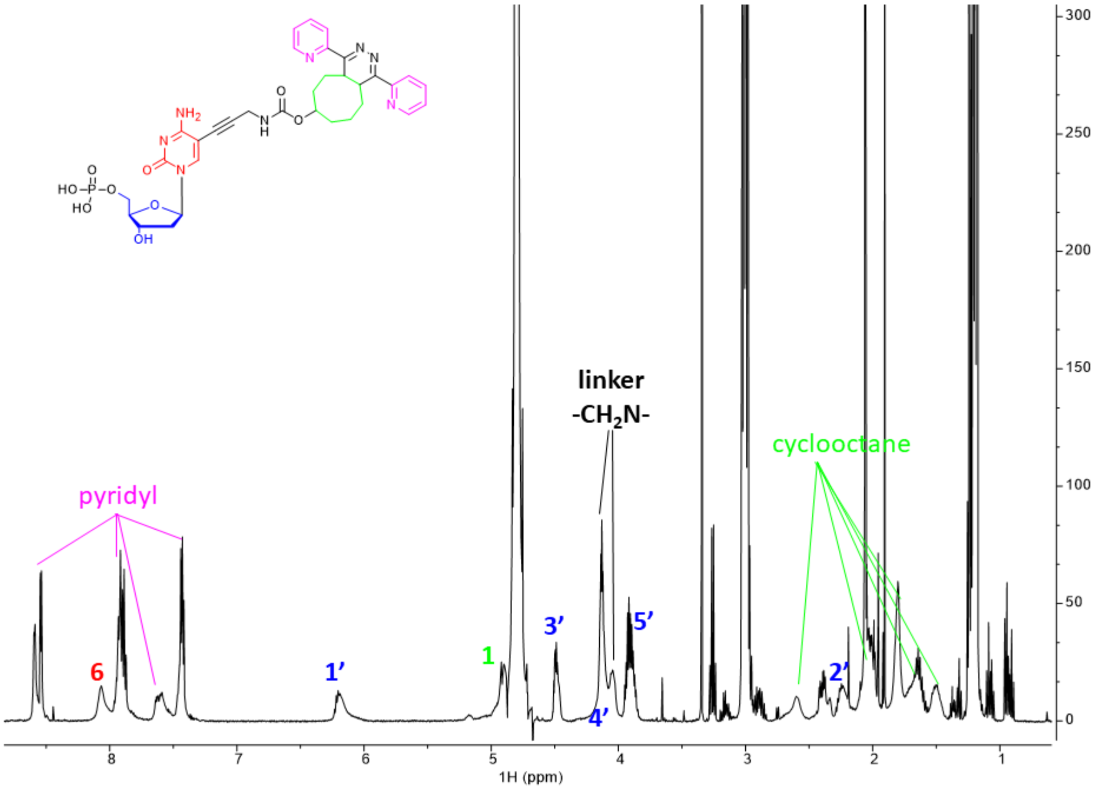

Figure S2. Assignment of structural fragments of **dC^{4TCOT5}MP** in ^{1}H NMR spectrum.

2. Experimental section - biochemistry

General remarks

All gels were analysed by fluorescence imaging using Typhoon FLA 9500 (GE Healthcare). Mass spectra of short DNAs were measured by UltrafleXtreme MALDI-TOF/TOF (Bruker) mass spectrometer with 1 kHz smartbeam II laser technology. The matrix consisted of 3-hydroxypicolinic acid (HPA)/ picolinic acid (PA)/ ammonium tartrate in ratio 9/ 1/ 1. UV-Vis spectra were measured at room temperature on NanoDrop1000 (ThermoScientific). Fluorescence was measured on a Fluoromax 4 spectrofluorimeter (HORIBA Scientific). Samples were concentrated on CentriVap Vacuum Concentrator system (Labconco). Synthetic oligonucleotides (primers, templates and biotinylated templates; for sequences see Table S1) were purchased from Generi Biotech (Czech Republic). Natural nucleoside triphosphates (dATP, dGTP, dTTP, dCTP) were purchased from Thermo Scientific. KOD XL DNA polymerase and corresponding polymerase reaction buffer from Merck Life Sciences,

streptavidin magnetic particles were obtained from Sigma Aldrich (Merck), QIAquick® Nucleotide Removal Kit QIAGEN (Biotech, Czech Republic). Milli-Q water was used for all experiments. PAGE stop solution used after PEX reactions contains: 95% [v/v] formamide, 0.5 mM EDTA, 0.025% [w/v] bromophenol blue, 0.025% [w/v] xylene cyanol, 0.025% SDS in MilliQ water. Samples after PEX reaction were analyzed by 12.5% PAGE (acrylamide/bisacrylamide 19:1, 25% urea) under denaturing conditions (50 min, 50 °C, 1 X TBE buffer) or by 12.5% or 20 % native PAGE (acrylamide/bisacrylamide 19:1, no urea) under following conditions of 3 h, 10 mA, RT, 1 X TBE buffer. 6X Loading buffer for native PAGE contains: 40% [w/v] saccharose, 0.02% [w/v] bromophenol blue, 0.02% [w/v] xylene cyanol in Milli-Q water. Tetrazine-coumarine conjugates **T1** and **T2** and PINK tetrazine **T3** were synthetized by Dr. Milan Vrabel`s group according to published methods.[3,6] Pyrimidyl-Tetrazine-5-TAMRA **T4** was purchased from (Jena Bioscience (CLK-097). Other chemicals were of analytical grade.

2.1 Lists of sequences of primers, templates and oligonucleotides used in this study

Table S1. List of sequences of primers and templates used in this study.

Name	Sequence (5’→3’)	Length
**Prim A**	5’-CATGGGCGGCATGGG-3’	15 nt
**Prim A_FAM** [a]	5’-CATGGGCGGCATGGG-3’	15 nt
**Temp19_1C**	5’-CCCG<u>CCCATGCCGCCCATG</u>-3’	19 nt
**Temp19_1C_bio** [b]	5’-CCCG<u>CCCATGCCGCCCATG</u>-3’	19 nt
**Temp19_1C_2xbio** [c]	5’-CCCG<u>CCCATGCCGCCCATG</u>-3’	19 nt

[a] 5’-(6-FAM)-labelled; [b] 5’-biotinylated; [c] 5’double-biotinylated; in the template oligonucleotides the segments forming duplex with the primer are underlined.

Table S2. List of nucleic acids used in this study

Name	Sequence (3’→5’)	Length
**19DNA_C$^{natural\ a}$**	3`-GGG**C**GGGTACGGCGGGTAC-5´	19 nt
**19DNA_C$^{4TCO\ a}$**	3`-GGG**C^{4TCO}**GGGTACGGCGGGTAC-5´	19 nt
**19DNA_C$^{2TCO\ a}$**	3`-GGG**C^{2TCO}**GGGTACGGCGGGTAC-5´	19 nt
**19DNA_C$^{p4TCO\ a}$**	3`-GGG**C$^{p4TCO}$**GGGTACGGCGGGTAC-5`	19 nt
**19DNA_C$^{p2TCO\ a}$**	3`-GGG**C$^{p2TCO}$**GGGTACGGCGGGTAC-5`	19 nt
**19DNA_C$^{pBCN\ a}$**	3`-GGG**C^{pBCN}**GGGTACGGCGGGTAC-5´	19 nt
**19ON_C^{4TCO}**	3`-GGG**C^{4TCO}**GGGTACGGCGGGTAC-5´	19 nt
**19ON_C^{2TCO}**	3`-GGG**C^{2TCO}**GGGTACGGCGGGTAC-5´	19 nt
**19ON_C^{p4TCO}**	3`-GGG**C$^{p4TCO}$**GGGTACGGCGGGTAC-5`	19 nt
**19ON_C$^{p2TCO\ a}$**	3`-GGG**C$^{p2TCO}$**GGGTACGGCGGGTAC-5`	19 nt
**19ON_C^{p2TCO}**	3`-GGG**C$^{p2TCO}$**GGGTACGGCGGGTAC-5`	19 nt
**19ON_C^{pBCN}**	3`-GGG**C^{pBCN}**GGGTACGGCGGGTAC-5´	19 nt
**19ON_C$^{pBCN\ a}$**	3`-GGG**C^{pBCN}**GGGTACGGCGGGTAC-5´	19 nt

[a] 5`-(6-FAM)-labelled, ONx-single-stranded DNA; DNAx-double-stranded DNA, the complementary strand is unmodified; primer regions underlined.

2.2 Enzymatic synthesis of TCO-, BCN-modified DNA

2.2.1 Single incorporation of dC^{4TCO}TP or dC^{2TCO}TP or dC^{p4TCO}TP or dC^{p2TCO}TP or dC^{pBCN}TP using 19-mer template-Analytical scale

The reaction mixture (20 μL) contained primer **Prim A_FAM** (3 μM, 1.5 μL), template **Temp19_1C** (3 μM, 1 μL), KOD XL DNA polymerase (0.25 U/μL, 0.11 μL), natural dGTP (4 mM, 0.5 μL), either natural or modified dCTP (4 mM, 0.3 μL) in enzyme reaction buffer (10 X, 2.05 μL) and water 14.65 μL. The reaction mixture was incubated for 20 min at 60 °C in a thermal cycler. The PEX reaction was stopped by addition of PAGE stop solution (20 μL) and heated for 3 min at 95 °C. Samples were separated with 12.5% denaturing PAGE and visualized using fluorescence imaging (Figure S3-S4).

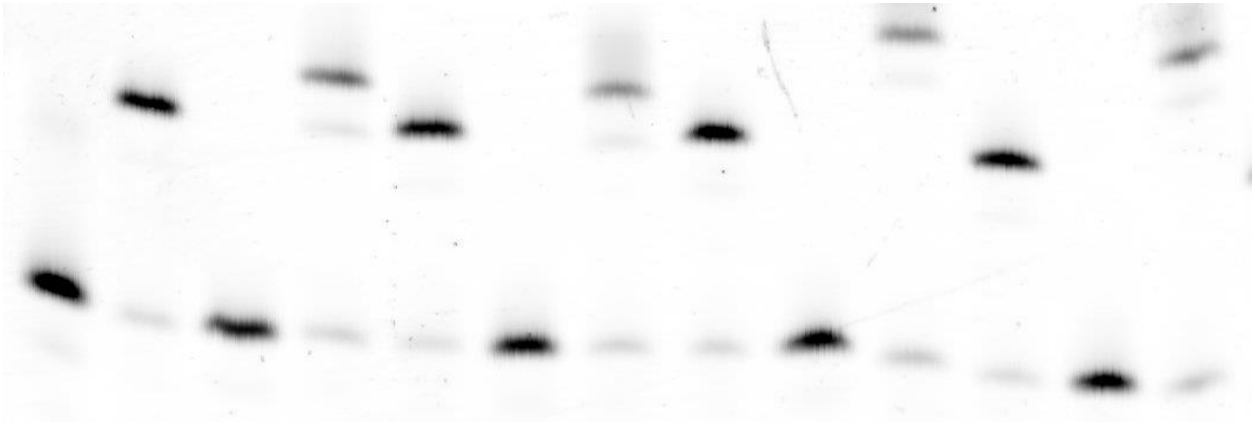

P C+ C- dC^{4TCO} C+ C- dC^{2TCO} C+ C- dC^{p4TCO} C+ C- dC^{p2TCO}

1 2 3 4 5 6 7 8 9 10 11 12 13

Figure S3. Denaturing PAGE analysis of PEX using KOD XL DNA polymerase and template **Temp19_1C** (lane 2-13). (P): primer **Prim A_FAM**, lane 1; (C+): natural dGTP, dCTP lanes 2,5,8,11; (C-): negative control without dCTP, lanes 3,6,9,12; (dC^{4TCO}): modified DNA containing **$dC^{4TCO}TP$**, dGTP, lane 4; ($dC^{2TCO}TP$): modified DNA containing **$dC^{2TCO}TP$**, dGTP, lane 7; ($dC^{p4TCO}TP$): modified DNA containing **$dC^{p4TCO}TP$**, dGTP, lane 10; ($dC^{p2TCO}TP$): modified DNA containing **$dC^{p2TCO}TP$**, dGTP, lane 13.

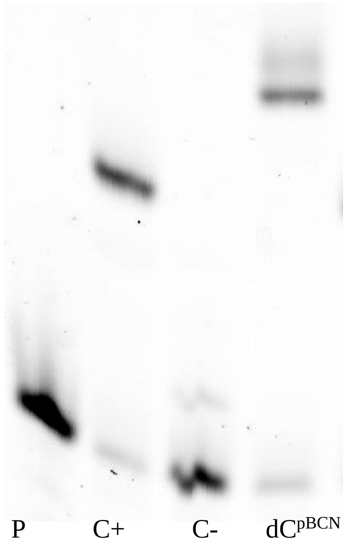

P C+ C- dC^{pBCN}

1 2 3 4

Figure S4. Denaturing PAGE analysis of PEX using KOD XL DNA polymerase and template **Temp19_1C** (lane 2-4). (P): primer **Prim A_FAM**, lane 1; (C+): natural dGTP, dCTP lane 2; (C-): negative control without dCTP, lane 3; (dC^{pBCN}): modified DNA containing **$dC^{pBCN}TP$**, dGTP lane 4.

2.2.2. Single incorporation of $dC^{4TCO}TP$ or $dC^{2TCO}TP$ or $dC^{p4TCO}TP$ or $dC^{p2TCO}TP$ or $dC^{pBCN}TP$ using 19-mer template-Semi-preparative scale

The reaction mixture (20 μL) contained KOD XL DNA polymerase (0.25 U/μL, 0.2 μL), primer **Prim A** (50 μM, 1 μL), template **Temp19_1C_2xbio** (50 μM, 1.5 μL), dGTP (4 mM, 0.5 uL), dCTP (modified; 4 mM, 0.3 μL each), in KOD XL reaction buffer (10 X, 2.05 μL). The reaction mixture was incubated in a thermal cycler for 20 min at 60 °C and stopped by cooling to 4 °C.

Biotinylated PEX products were purified using DB Streptavidin magneto-separation procedure and then analysed by MALDI-TOF mass spectrometry and used for fluorescence turn-on measurements in case of **19ON_C**^{p4TCO} and **19ON_C**^{pBCN} (See Table S3 and Section 7 for copies of mass spectra).

2.3. DB Streptavidin magneto-separation procedure:

Streptavidin particles (Roche; 50 μL) were washed with binding buffer (3 × 200 μL; 10 mM Tris, 1 mM EDTA, 100 mM NaCl, pH 7.5). The PEX solution (50 μL) was mixed with binding buffer (200 μL) and incubated for 30 min at 15 °C and 1400 rpm. The magnetic beads were collected on a magnet (DynaMagTM-2, Invitrogen), and washed with wash buffer (3 × 300 μL; 10 mM Tris, 1 mM EDTA, 500 mM NaCl, pH 7.5) and water (4 × 300 μL). Then water (50 μL) was added and the sample was denatured for 2 min at 900 rpm and 75 °C. The beads were collected on a magnet and the solution was transferred into a clean vial. The product was evaporated to dryness, then dissolved in water and analysed by MALDI-TOF mass spectrometry.

2.3.1. MALDI data of dC^{4TCO} or dC^{2TCO} or dC^{p4TCO} or dC^{p2TCO} or dC^{pBCN}-modified nucleic acids.

All copies of mass spectra of **dC**^{4TCO} or **dC**^{2TCO} or **dC**^{p4TCO} or **dC**^{p2TCO} or **dC**^{pBCN}-modified nucleic acids are in Section 8.

Table S3. Overview of dC^{4TCO} or dC^{2TCO} or dC^{p4TCO} or dC^{p2TCO} or dC^{pBCN}-modified nucleic acids and their masses

Nucleic acids	Mw calculated [Da]	Mw found [Da]	Δ [Da]	Figure Number
**19ON_ C^{4TCO}**	6156.0	6157.2 [b]	1.2	Figure S50
**19ON_C^{2TCO}**	6156.0	6159.3 [b]	3.3	Figure S53
**19ON_C^{p4TCO}**	6359.1	6360.1 [b]	1.0	Figure S56
**19ON_C^{p2TCO}**	6359.1	6360.6 [b]	1.5	Figure S59
**19ON_C^{pBCN}**	6383.1	6384.6 [b]	1.5	Figure S62
**19ON_C^{pBCN}** [a]	6920.1	6918.4	1.7	Figure S69
**19DNA_C^{pBCN}** [a]	6920.1	6921.3 [b]	1.2	Figure S70
**19ON_C^{p4TCO}** [a]	6896.1	6896.1	0	Figure S71
**19DNA_C^{p4TCO}** [a]	6896.1	6897.6 [b]	1.5	Figure S72

[a] 5`-(6-FAM)-labelled, ON^{X}-single stranded; DNA^{X}-double-stranded DNA
[b] Nucleic acids measured in positive mode.

3. Fluorescence turn-on measurements and LC/MS characterization of dC^{XTY}TP click products

Fluorescence measurements were performed on a FluoroMax 4 spectrofluorometer (Jobin Yvon, Horiba) from *Perkin Elmer* equipped with a 450 W xenon lamp and a single cuvette reader using data algebra formula S1c/R1c.

The MS spectra were measured on UPLC Agilent 1290 Infinity II bio system equipped with DAD and LC/MSD XT MS (operating in negative mode) detectors, with bioZen™ Oligo LC column (1.7 µm, 50×2.1 mm; Phenomenex®) using a linear gradient of 12 mM Et_3N and 300 mM HFIP in H_2O to 12 mM Et_3N and 300 mM HFIP in MeOH in 30 min.

3.1 Fluorescence measurements and LC-MS characterization of dC^{4TCO}TP or dC^{2TCO}TP with T1 or T2 tetrazines and of dC^{p4TCO}TP or dC^{p2TCO}TP or dC^{pBCN}TP with T1, T2 or T3 tetrazines (dC^{XTY}TP)

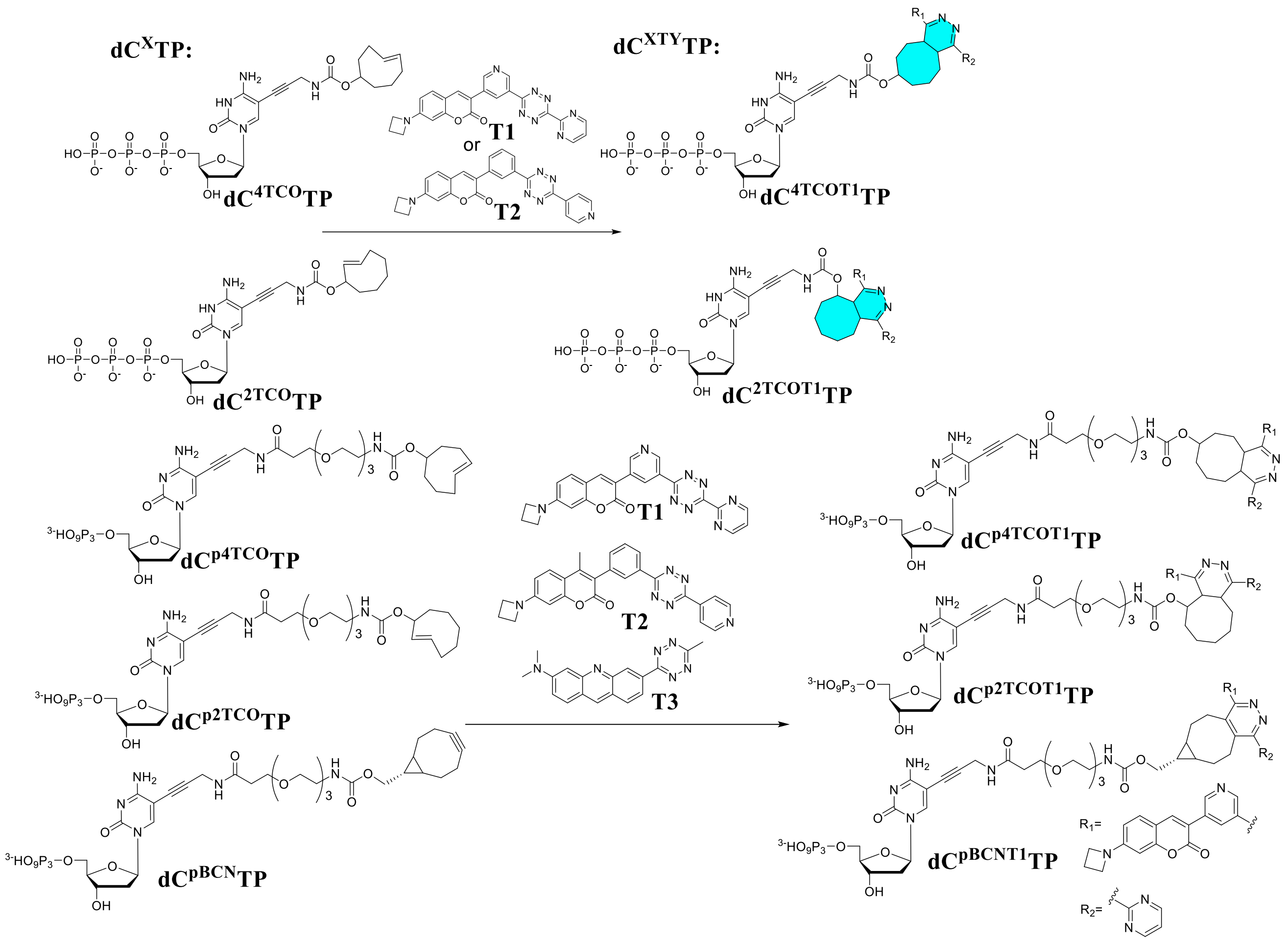

Scheme S2. Reaction scheme of the click product formed in the reaction of **dC^{4TCO}TP** or **dC^{2TCO}TP** with **T1** or **T2**, **dC^{p4TCO}TP** or **dC^{p2TCO}TP** or **dC^{pBCN}TP** with **T1**, **T2**, **T3** tetrazines. Only one isomer of the click product is shown for clarity.

Procedure: Freshly prepared stock solution of **T1** tetrazine (1 mM in DMSO) was used for the measurements. The turn-on fluorescence measurements were performed as follows: 1 µL of the tetrazine stock solution was diluted in 1 mL PBS buffer (1 X, pH 7.4, giving 1 µM final concentration) and the fluorescence was measured using 370 nm excitation (slit 3) and 400-650 nm for emission.

To this solution was added solution of the triphosphates (**dC^{4TCO}TP** or **dC^{2TCO}TP** or **dC^{p4TCO}TP** or **dC^{p2TCO}TP** or **dC^{pBCN}TP)**: 1 µL from 1 mM fresh stock in water, giving 1 µM final concentration, 1 equiv. to tetrazine). The cuvette was inserted into the fluorescence spectrophotometer and the measurement was started. The spectra were recorded at several time points and all measurements were typically repeated two-three times. The final measurement was performed after ca. 30 minutes. The data were processed using Origin software. All spectra were subtracted from the baseline (PBS buffer

as the blank). The turn-on values were calculated from the observed fluorescence intensities of the click products at the emission maximum (485 nm) after ca. 15 min of reaction divided by the highest residual fluorescence of the quenched coumarin tetrazine dye. These values are presented for **dC^{4TCO}TP**, **dC^{2TCO}TP**, **dC^{p4TCO}TP**, **dC^{p2TCO}TP** in Figure S5-A,B,C,D and for **dC^{pBCN}TP** in Figure S6 and Table S4 in the main text. The highest fluorescence signal intensity was recorded for reaction with the axial **dC^{p4TCO}TP** as listed in Table S4. Fluorescence time lapse measurements showing changes in the fluorescence signal of the click products over 30 min for **T1** (Figure S10-A). Fluorescence time-lapse measurements of **dC^{p4TCO}TP** or **dC^{p2TCO}TP** or **dC^{pBCN}TP** with **T1** tetrazine showing changes in the fluorescence signal of the click products in time (Figure S11 and Table S6).

Due to the lower reactivity of the **T2** tetrazine, the fluorescence measurements were performed as follows:

Procedure: 1 μL of 1 mM solution of **dC^{4TCO}TP**, **dC^{2TCO}TP**, **dC^{pBCN}TP**, **dC^{p4TCO}TP** or **dC^{p2TCO}TP** were combined with 1 μL of 1 mM solution of **T2** tetrazine in 8 μL PBS and the reaction mixtures were incubated in the dark at room temperature for 1 hour. Then, the reaction mixtures were diluted to 1 mL with PBS to give final 1 μM solution and the fluorescence was measured using 370 nm excitation wavelength and 400-650 nm emission window (slit 3). The values are presented in Figures S7-A,B, S8-A,B,C, S10-B and Table S4.

Due to the lower reactivity of the **T3** tetrazine, the fluorescence measurements were performed as follows:

Procedure: 1 uL of 1 mM solution of **dC^{pBCN}TP** or **dC^{p4TCO}TP** were combined with 1 uL of 1 mM solution of **T3** tetrazine and the reaction mixtures were incubated in the dark at room temperature for 2 hours. Then, the reaction mixtures were diluted to 1 mL with PBS and the fluorescence was measured using 500 nm excitation wavelength and 525-750 nm emission window (slit 3). The values are presented in Figure 9-A,B and Table S4.

Preparation of nucleotide-tetrazine conjugates dC^{XTY}TP for LC/MS characterization

dC^{X}TP (**X**= **-4TCO** or **-2TCO** or **-p4TCO** or **-p2TCO** or **-pBCN**) dissolved in water (10 μM, 5 μL) was mixed with excess of **T1** or **T2** or **T3** tetrazines dissolved in DMSO (100 μM, 5 μL). The mixture containing **T1** or **T2** was left stirring at 37 °C for 30 min. The mixture containing **T3** was left stirring

at 37 °C for 18h. The identity of the **dC^{X}TP** controls and **dC^{XTY}TP** click products was verified by analytical LC/MS and by high resolution MS. From MS spectra addition of water to the click product (**dC^{XTY}TP**) was observed[4]. Estimated formation (%) of **dC^{XTY}TP** click products are shown in Table S5.

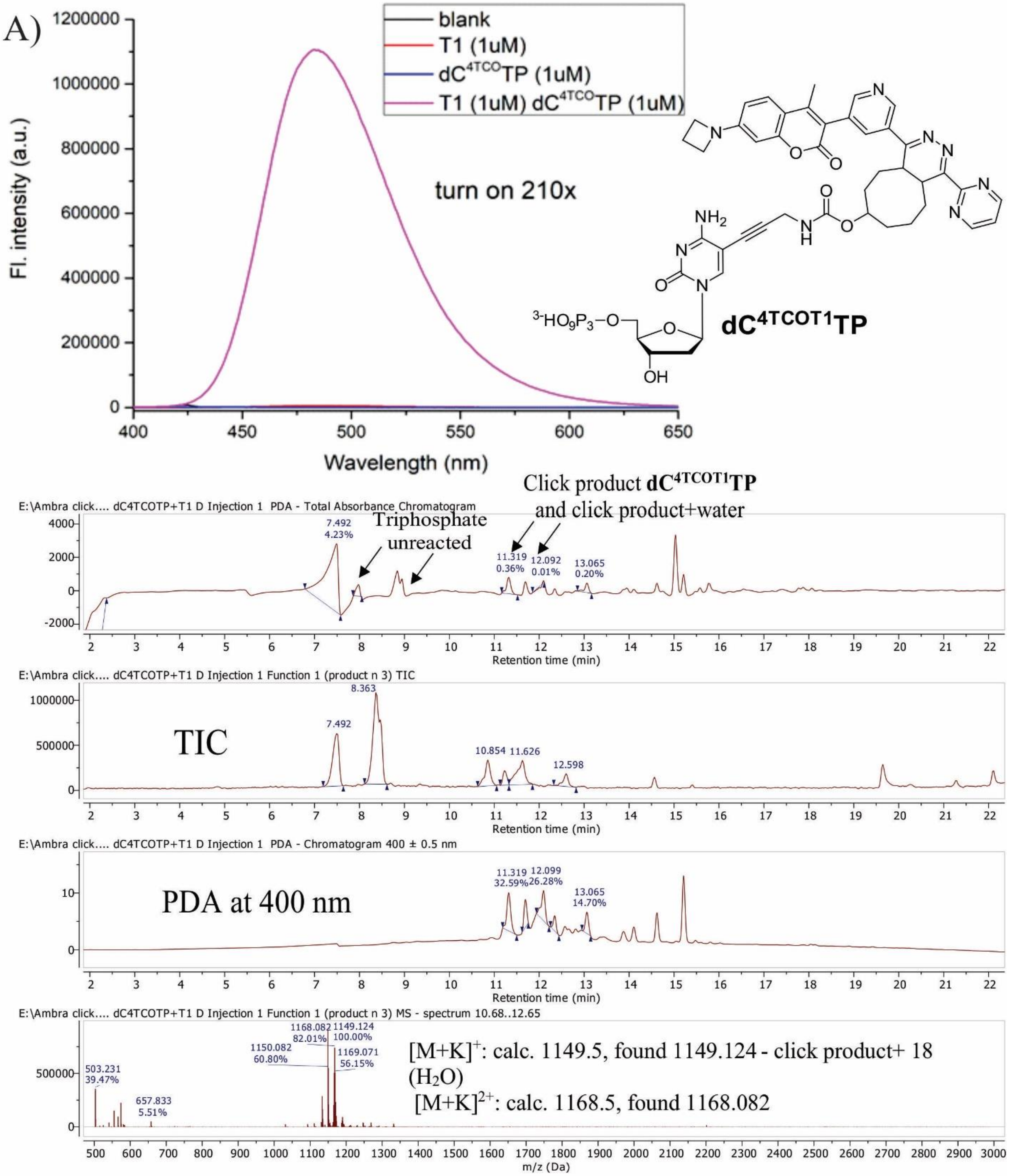

LC-MS characterization of **dC^{4TCOT1}TP** click product.

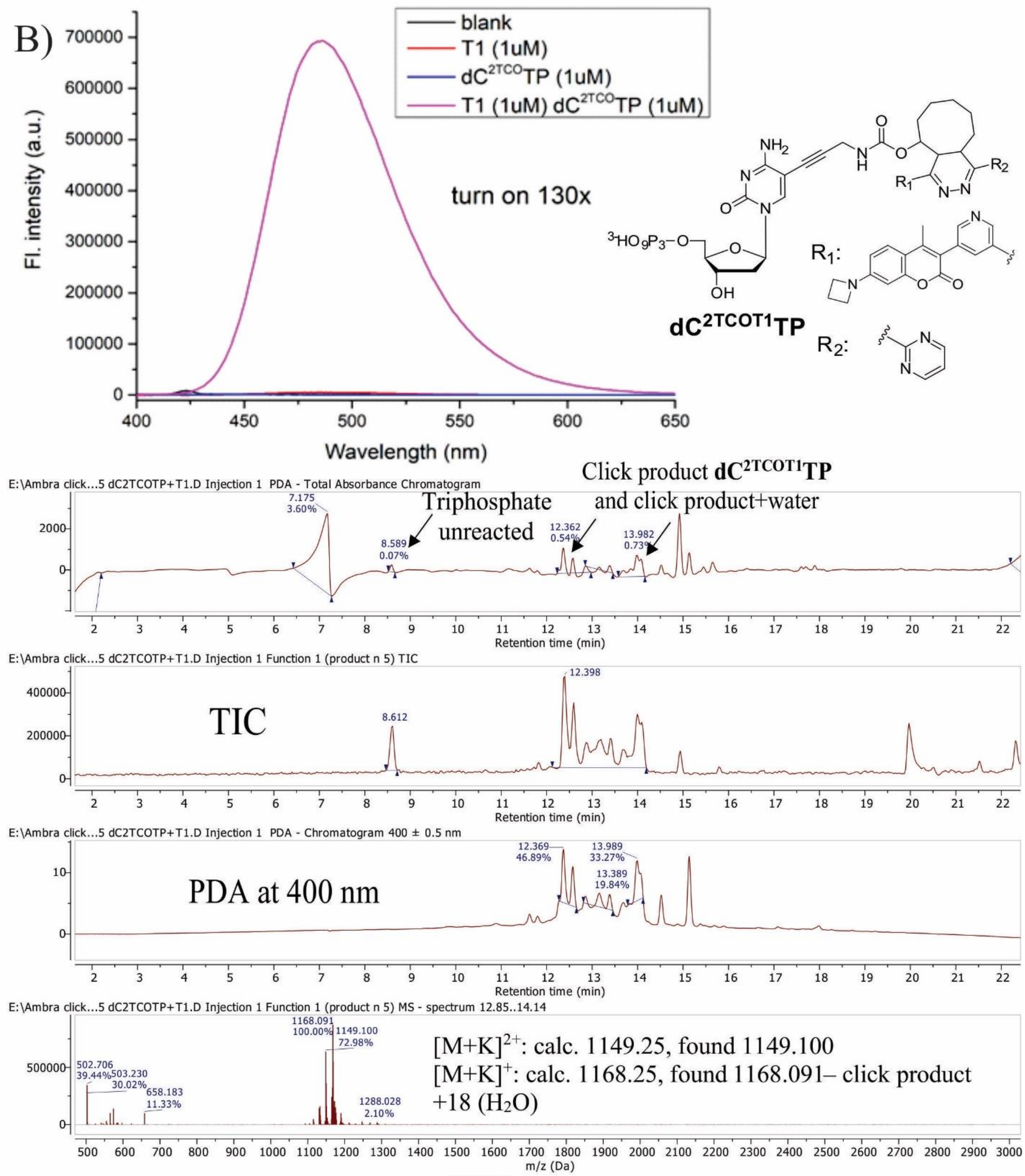

LC/MS characterization of **dC^{2TCOT1}TP** click product.

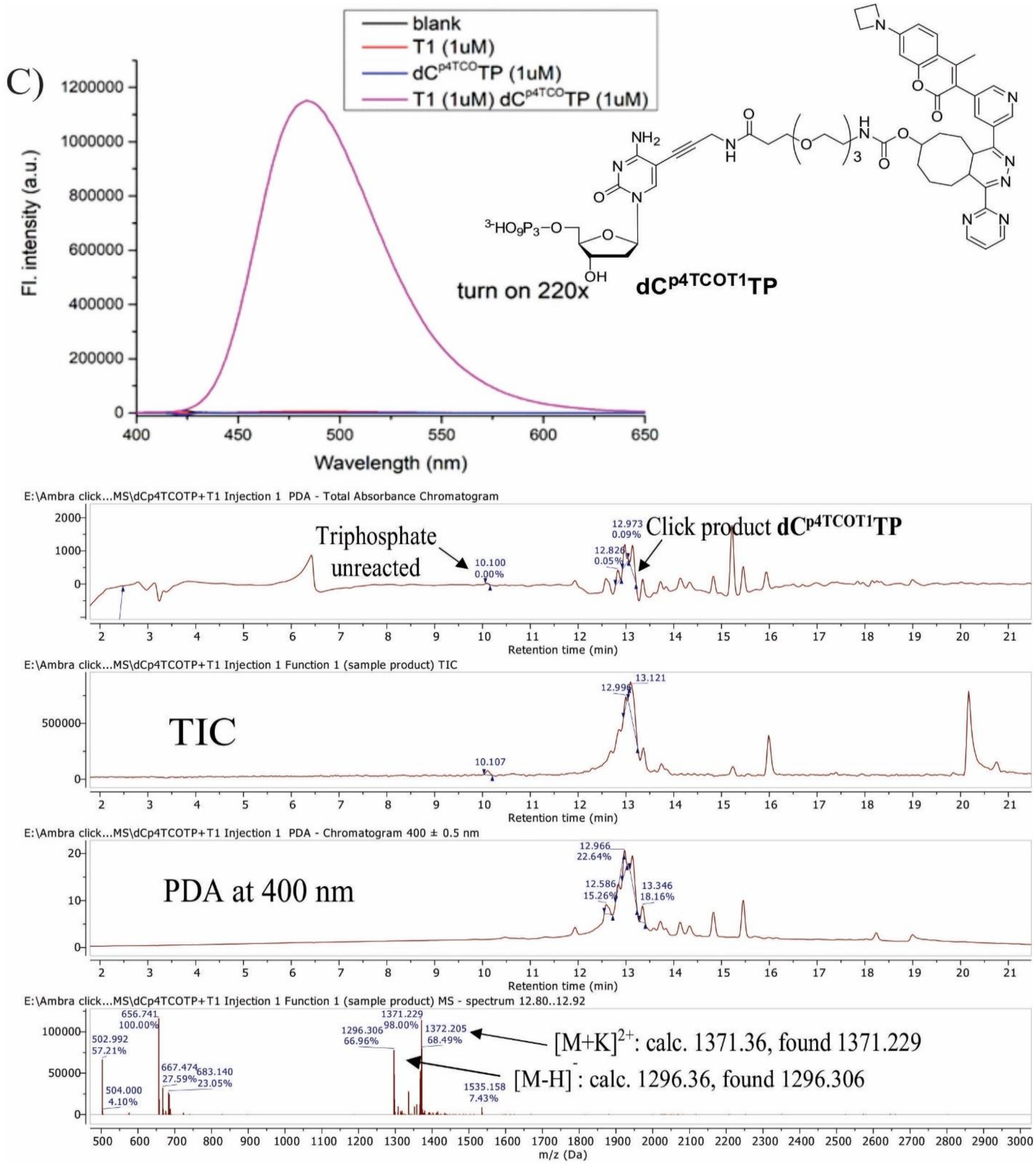

LC-MS characterization of **dC^{p4TCOT1}TP** click product.

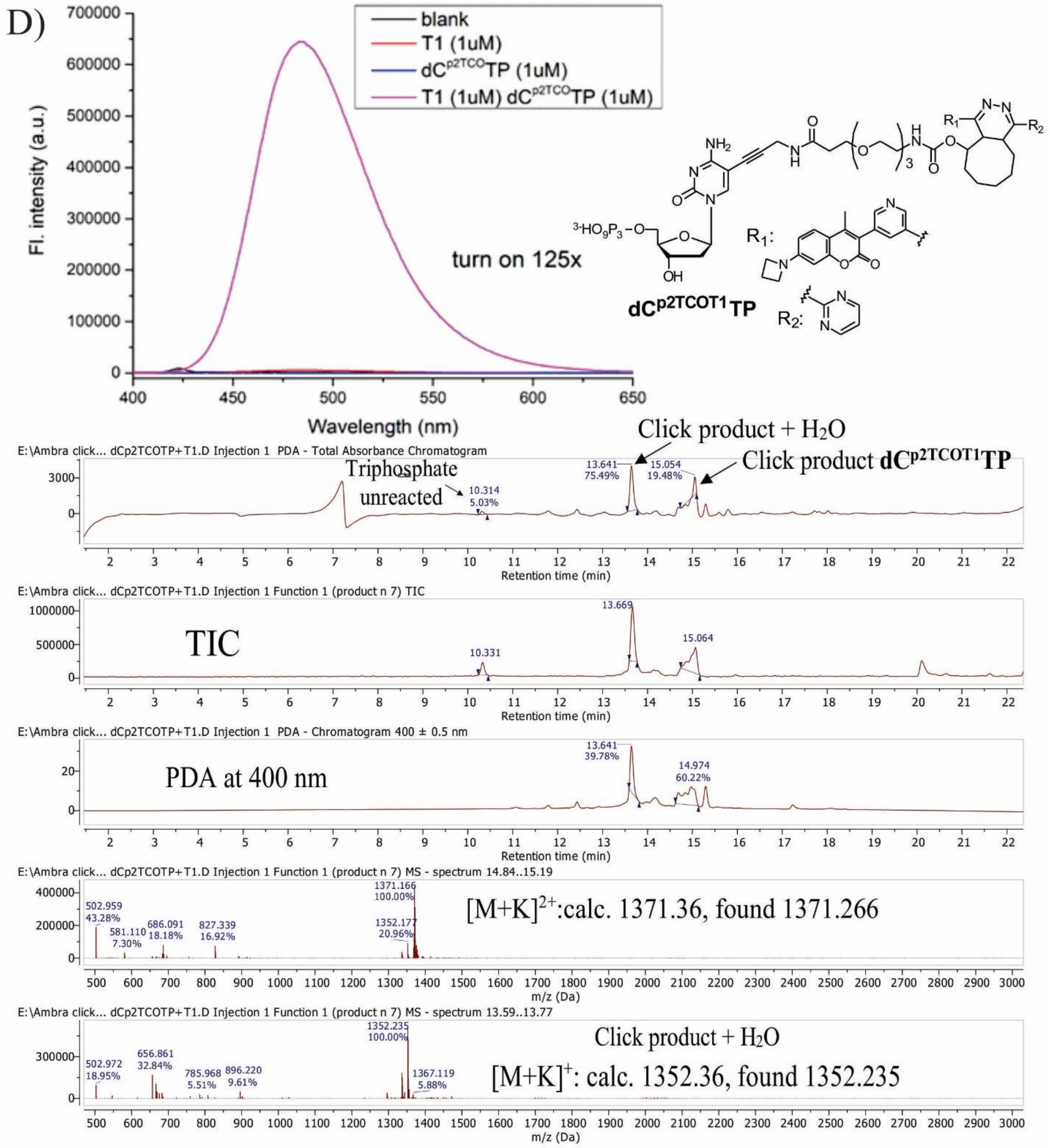

LC-MS characterization of **dC^{p2TCOT1}TP** click product.

Figure S5. Fluorescence turn on measurements and LC-MS spectra of the click products formed in the reaction of **T1** tetrazine with A) **dC^{4TCO}TP**, B) **dC^{2TCO}TP**, C) **dC^{p4TCO}TP**, D) **dC^{p2TCO}TP** (indicated as number-x = x-fold). Fluorescence spectra of the click products (magenta line) are shown

in relation to the respective quenched tetrazine (red line), the triphosphate alone (blue line) and PBS blank (black line) to indicate the fluorescence turn-on ratio.

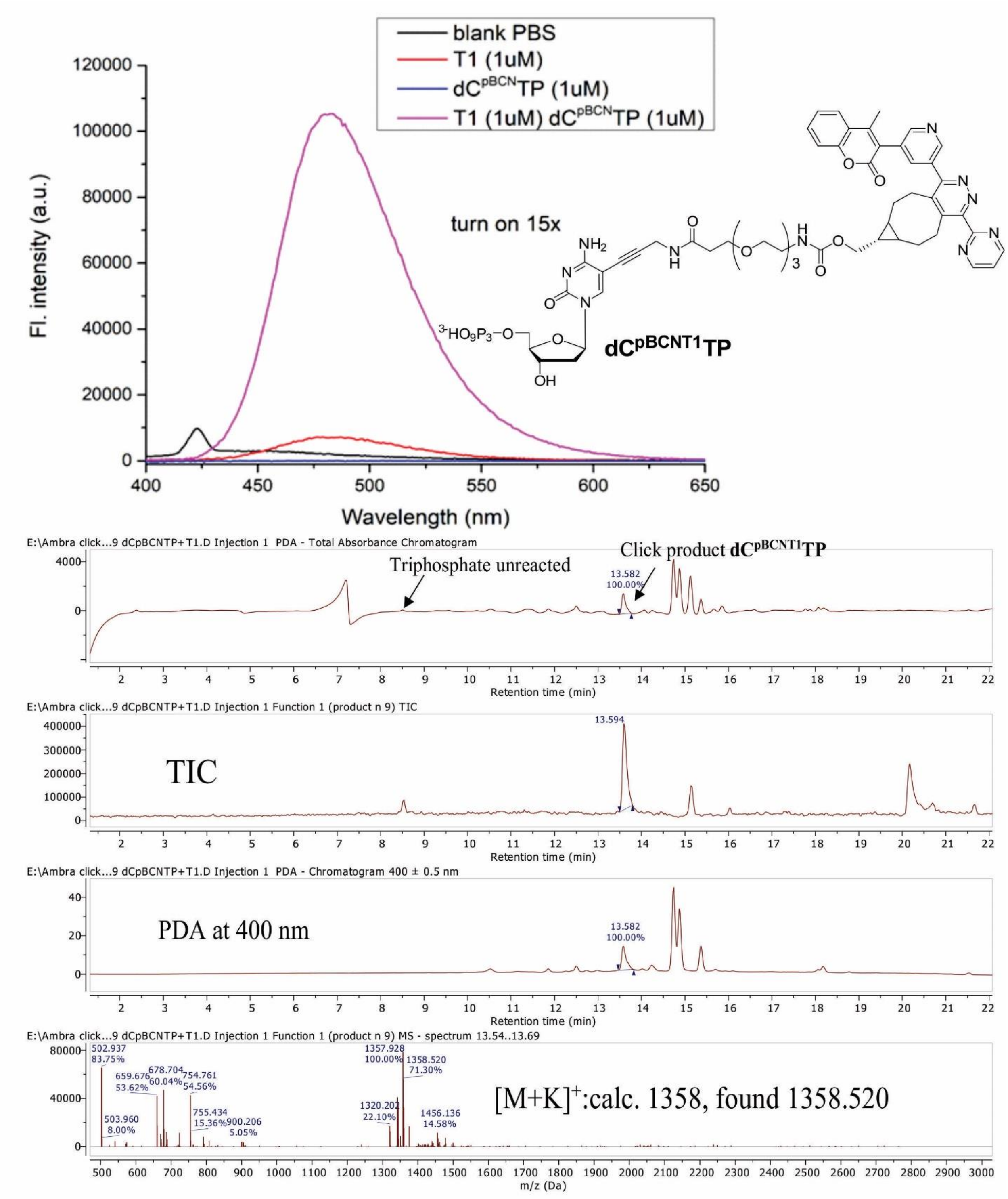

LC-MS characterization of **dC^{pBCNT1}TP** click product.

Figure S6. Fluorescence turn on meaurement and LC-MS spectrum of the click product formed in the reaction of **T1** tetrazine with **dC^{pBCN}TP** (indicated as number-x = x-fold). Fluorescence spectra of the click product (magenta line) are shown in relation to the respective quenched tetrazine (red line), the triphosphate alone (blue line) and to PBS blank (black line) to indicate the fluorescence turn-on ratio.

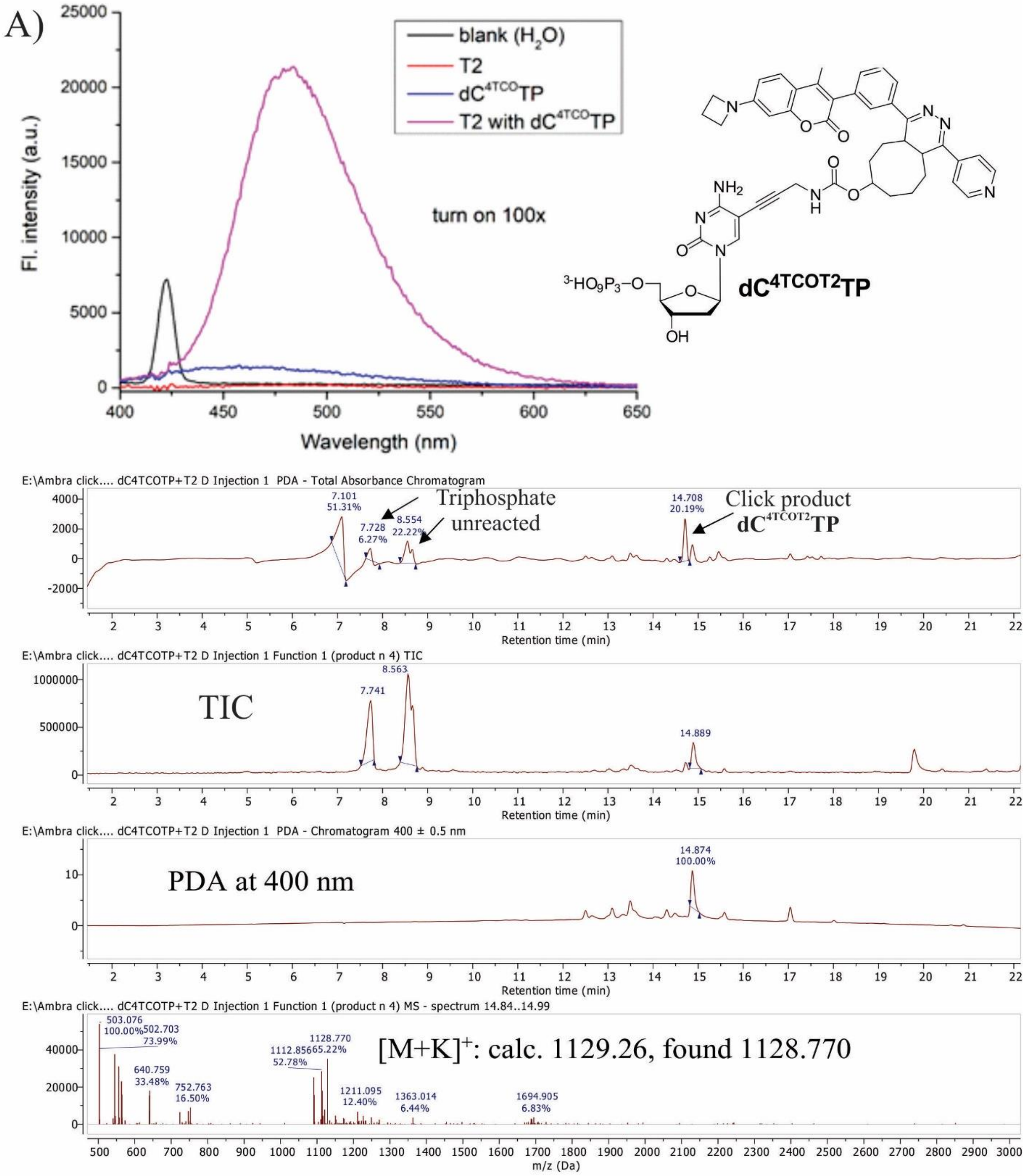

LC-MS characterization of **dC^{4TCOT2}TP** click product.

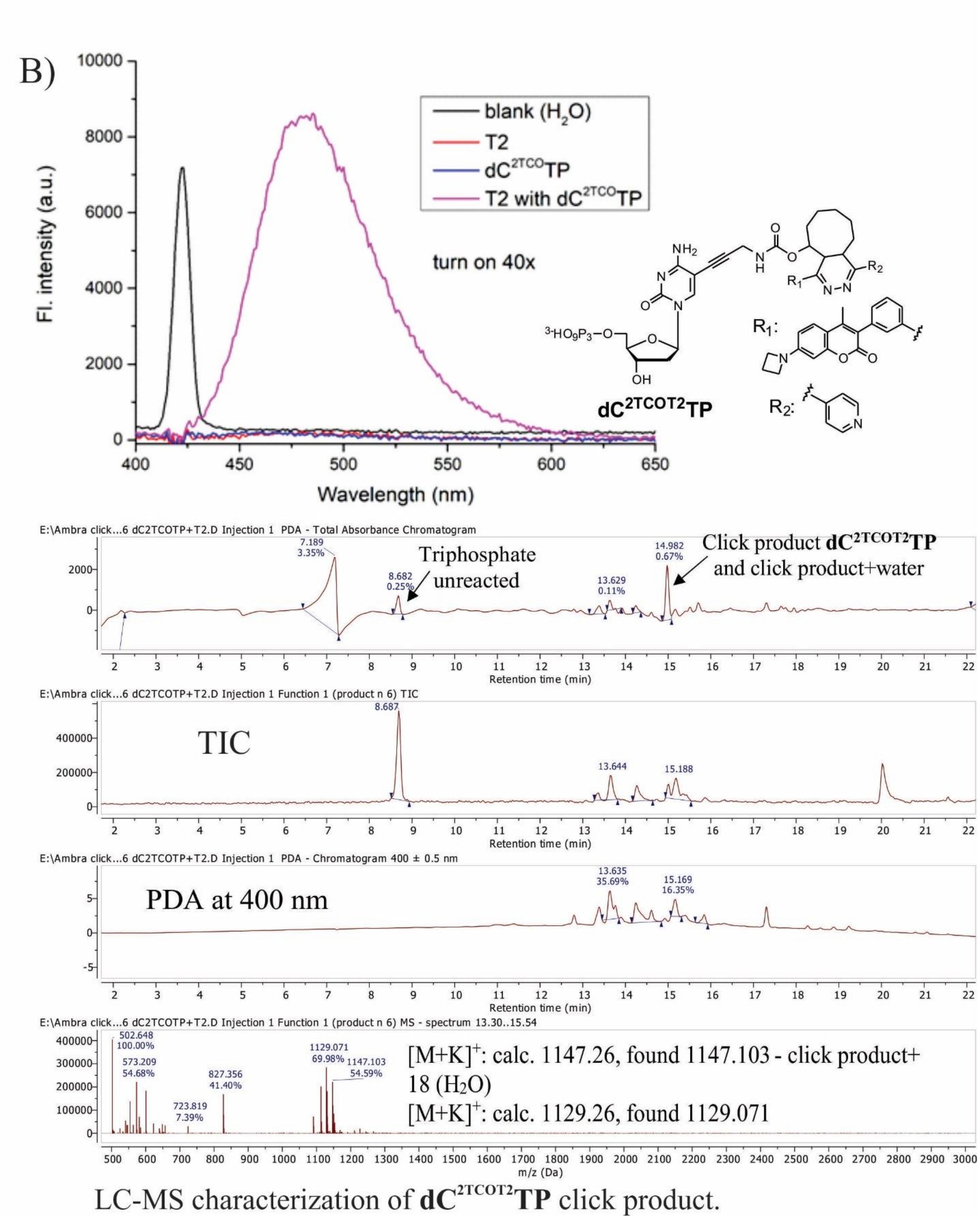

Figure S7. Fluorescence turn on measurement and LC-MS spectra of the click product formed in the reaction of **T2** tetrazine with A) **dC^{4TCO}TP** (indicated as number-x = x-fold), B) **dC^{2TCO}TP**.

Fluorescence spectra of the click product (magenta line) are shown in relation to the respective quenched tetrazine (red line), the trisphosphate alone (blue line) and to PBS blank (black line) to indicate the fluorescence turn-on ratio.

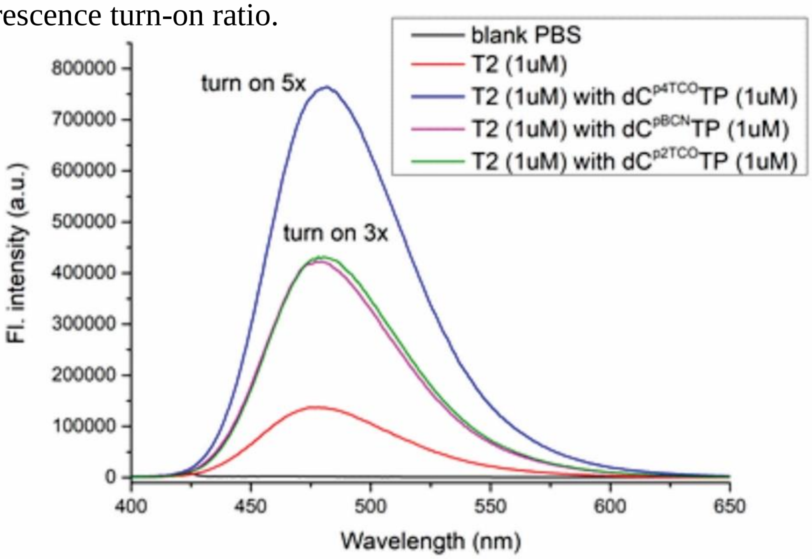

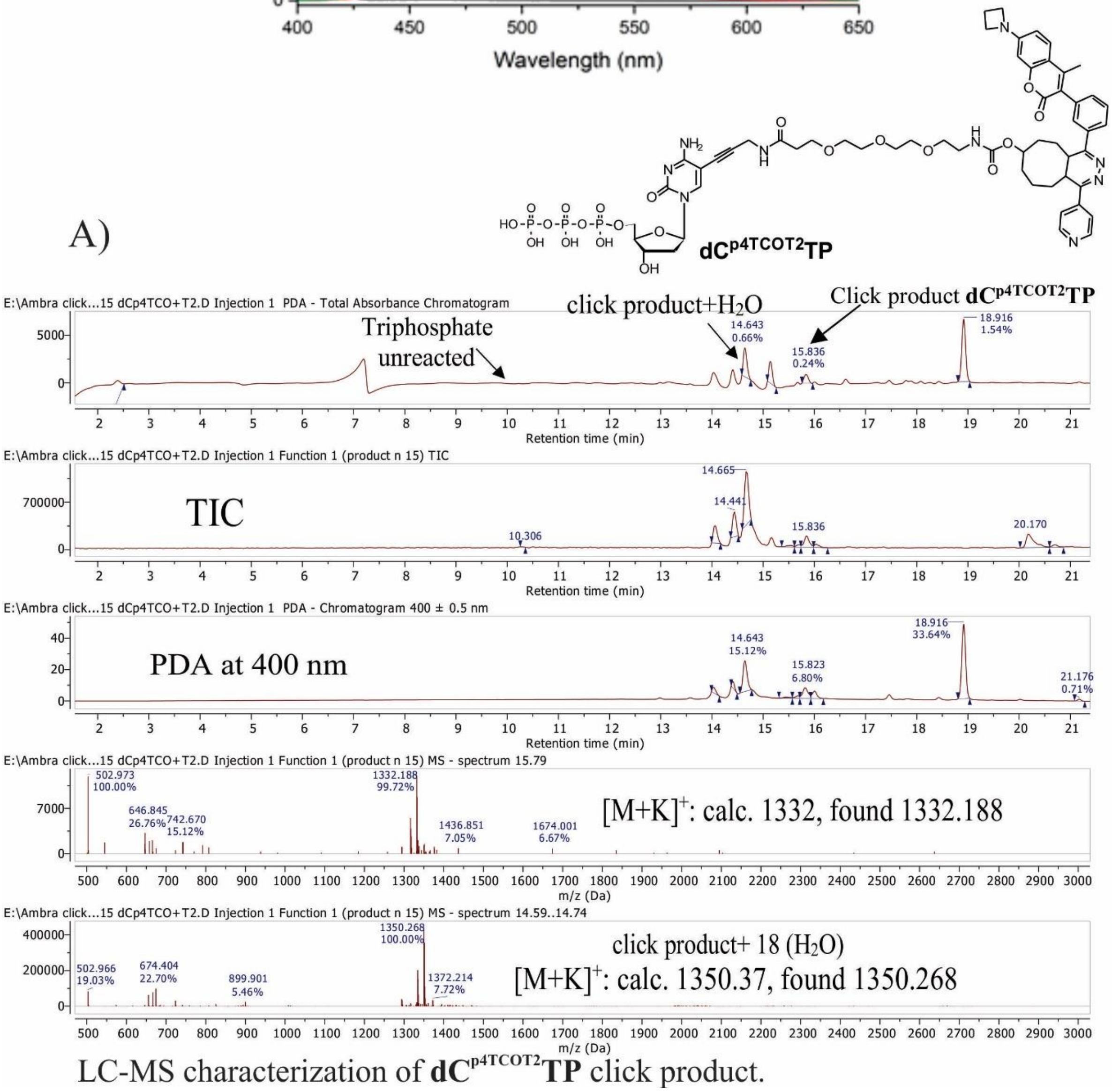

LC-MS characterization of **dC**^{p4TCOT2}**TP** click product.

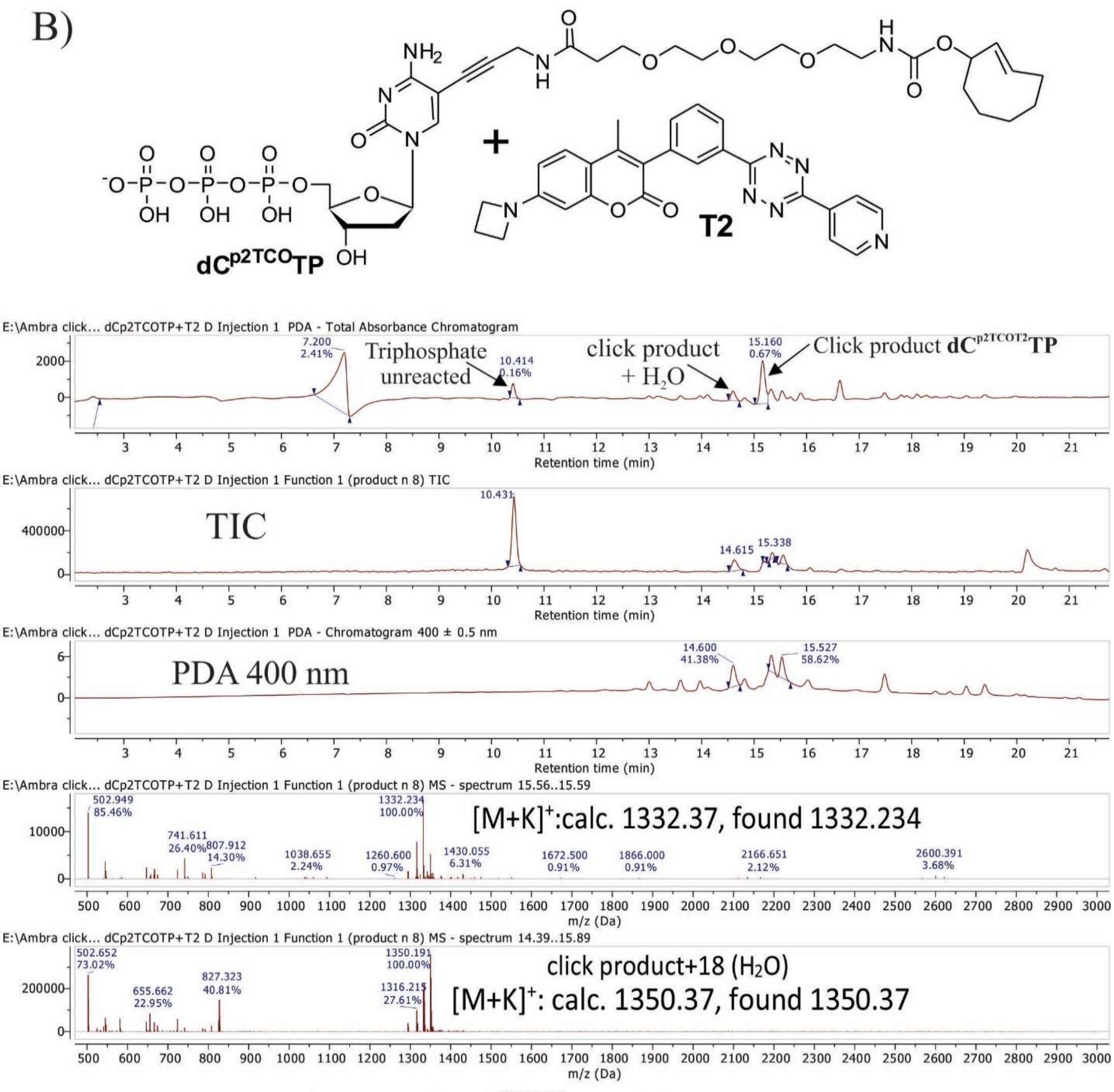

LC-MS characterization of **dC^{p2TCOT2}TP** click product.

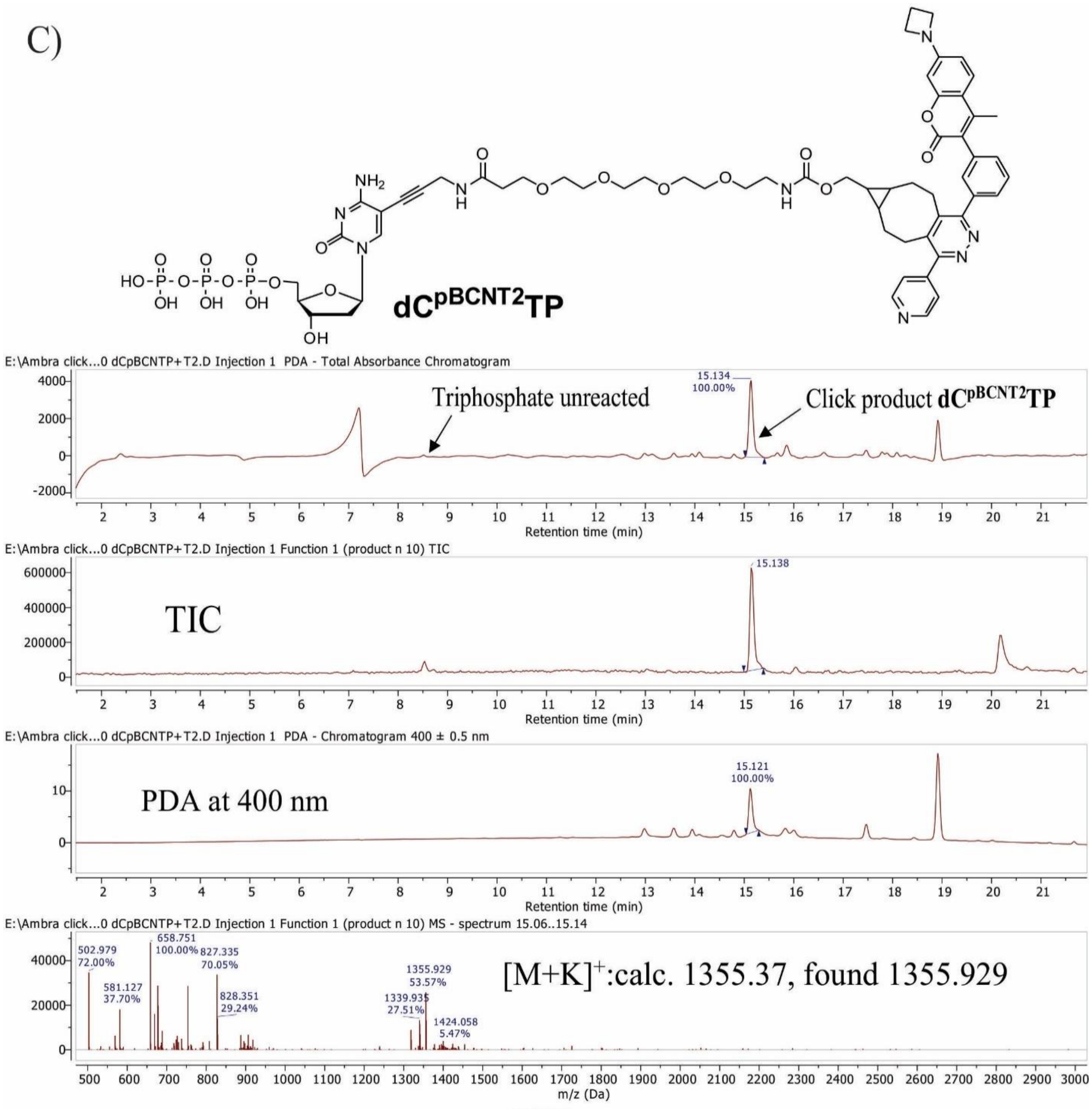

LC-MS characterization of **dC^{pBCNT2}TP** click product.

Figure S8. Fluorescence turn on meaurements and LC-MS spectra of the click products formed in the reaction of **T2** tetrazine with A) **dC^{p4TCO}TP** (indicated as number-x = x-fold), B) **dC^{p2TCO}TP** and C) **dC^{pBCN}TP**. Fluorescence spectra of the click products (blue, magenta and green line) are shown in relation to the respective quenched tetrazine (red line) and to PBS blank (black line) to indicate the fluorescence turn-on ratio.

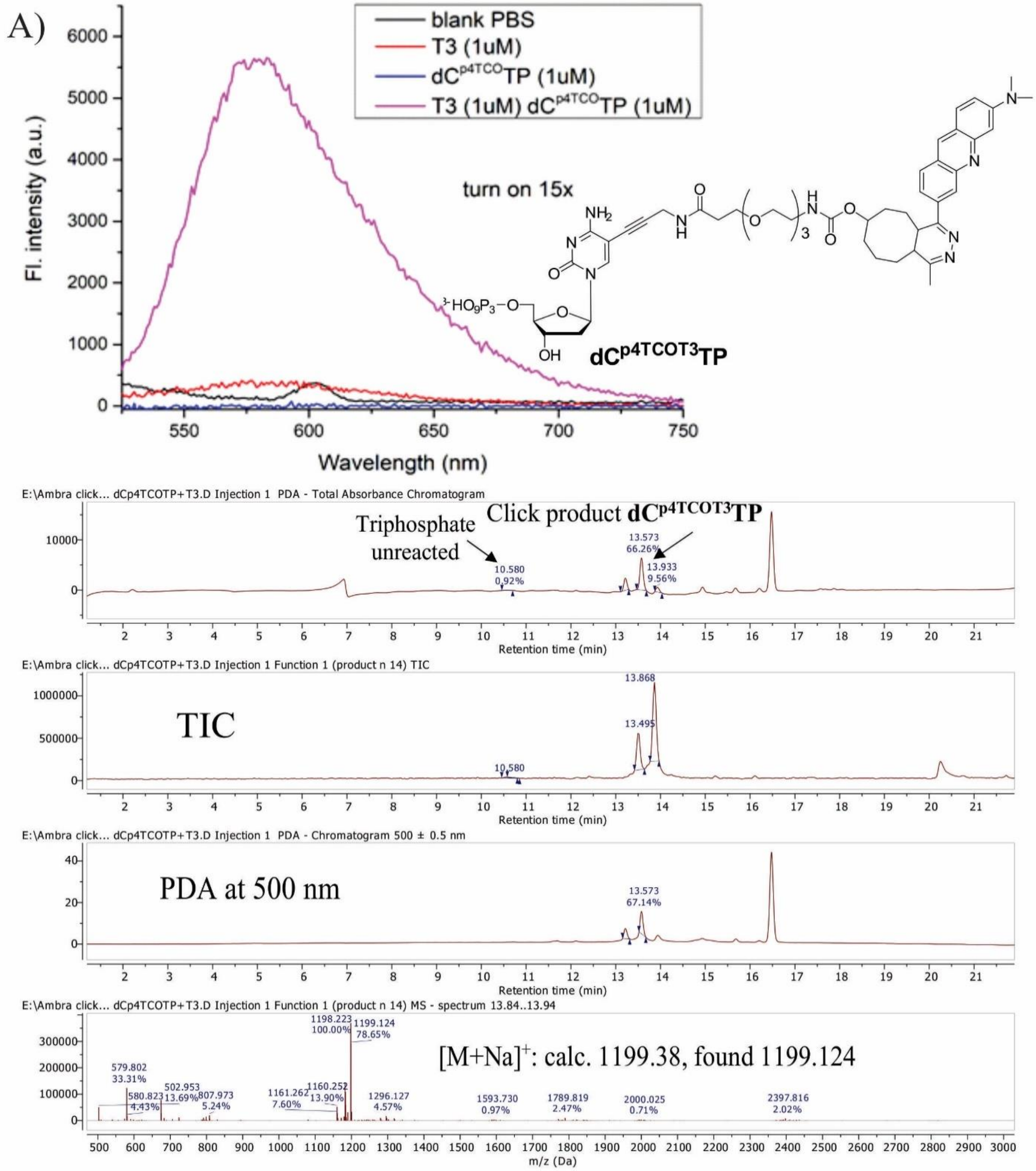

LC-MS characterization of **dC^{p4TCOT3}TP** click product.

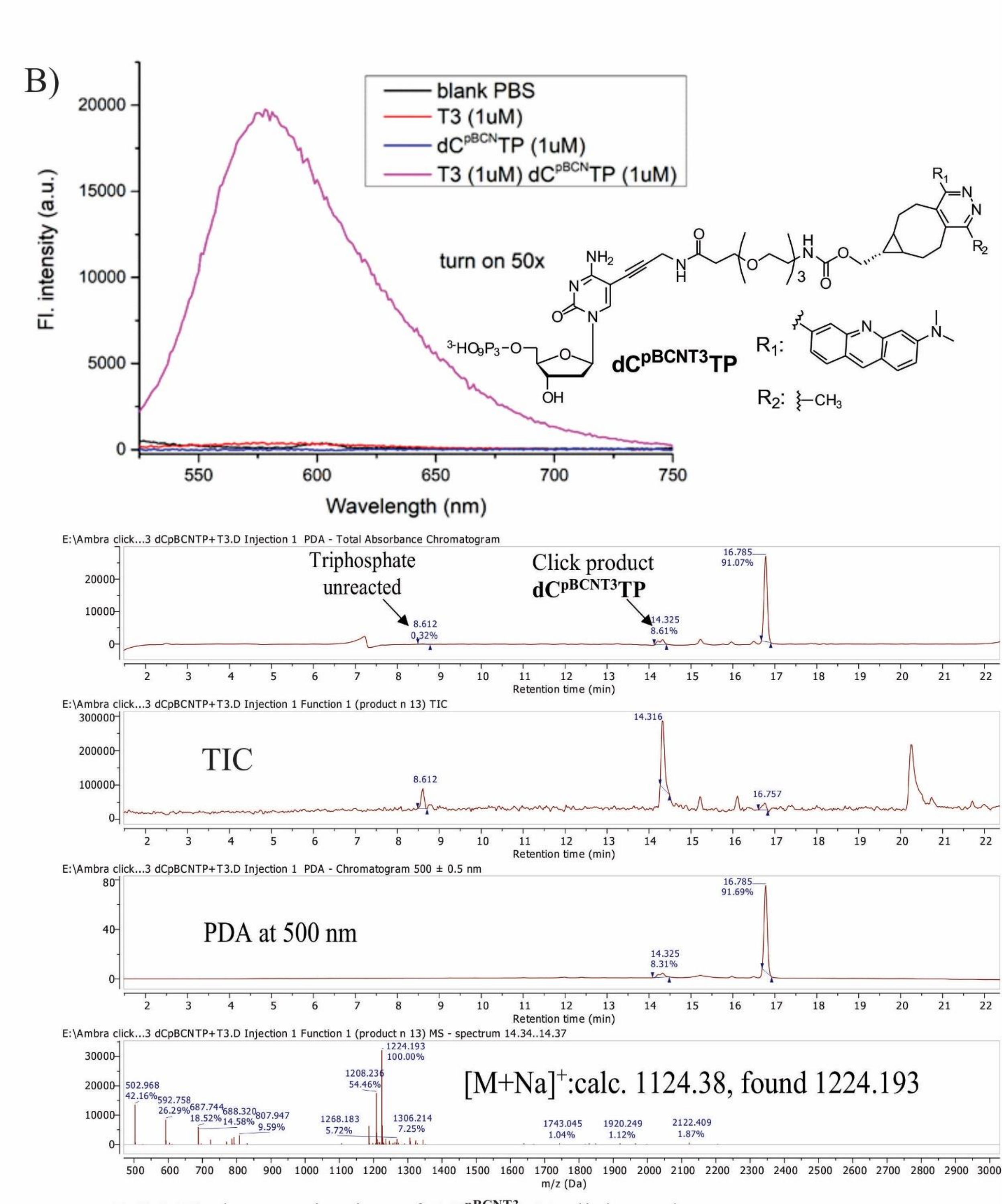

LC-MS characterization of **dC^{pBCNT3}TP** click product.

Figure S9. Fluorescence turn on measurements and LC-MS spectra of the click products formed in the reaction of **T3** tetrazine with A) **dC^{p4TCO}TP**, B) **dC^{pBCN}TP** (indicated as number-x = x-fold). Fluorescence spectra of the click products (magenta line) are shown in relation to the respective

quenched tetrazine (red line), the triphosphate alone (blue line) and PBS blank (black line) to indicate the fluorescence turn-on ratio.

Table S4. Summary of the turn-on values for the click products

Tetrazine	Triphosphate	Fluorescence enhancement[a]
T1	**dC^{4TCO}TP**	210-fold
T2	**dC^{4TCO}TP**	100-fold
T1	**dC^{2TCO}TP**	130-fold
T2	**dC^{2TCO}TP**	40-fold
T1	**dC^{p4TCO}TP**	220-fold
T3	**dC^{p4TCO}TP**	15-fold
T1	**dC^{p2TCO}TP**	125-fold
T1	**dC^{pBCN}TP**	15-fold
T3	**dC^{pBCN}TP**	50-fold
T1	**19ON_C^{p4TCO}**	110-fold
T1	**19ON_C^{pBCN}**	8.5-fold
T3	**19ON_C^{pBCN}**	14-fold

[a]determined in PBS buffer (pH 7.4) using 370 nm excitation and calculated by dividing the fluorescence value at 485 nm for click product and that of the quenched **T1** or **T2** or **T3** tetrazines.

Table S5. Estimated conversions of **dC^{XTY}TP** click products.

**Click product code (dC^{XTY}TP)**	**Conversion (%)**
**dC^{4TCOT1}TP**	58%
**dC^{4TCOT2}TP**	27%
**dC^{2TCOT1}TP**	78%
**dC^{2TCOT2}TP**	49%
**dC^{p4TCOT1}TP**	94%
**dC^{p4TCOT2}TP**	95%
**dC^{p4TCOT3}TP**	88%
**dC^{p2TCOT1}TP**	90%
**dC^{p2TCOT2}TP**	55%
**dC^{pBCNT1}TP**	75%
**dC^{pBCNT2}TP**	74%
**dC^{pBCNT3}TP**	73%

A)

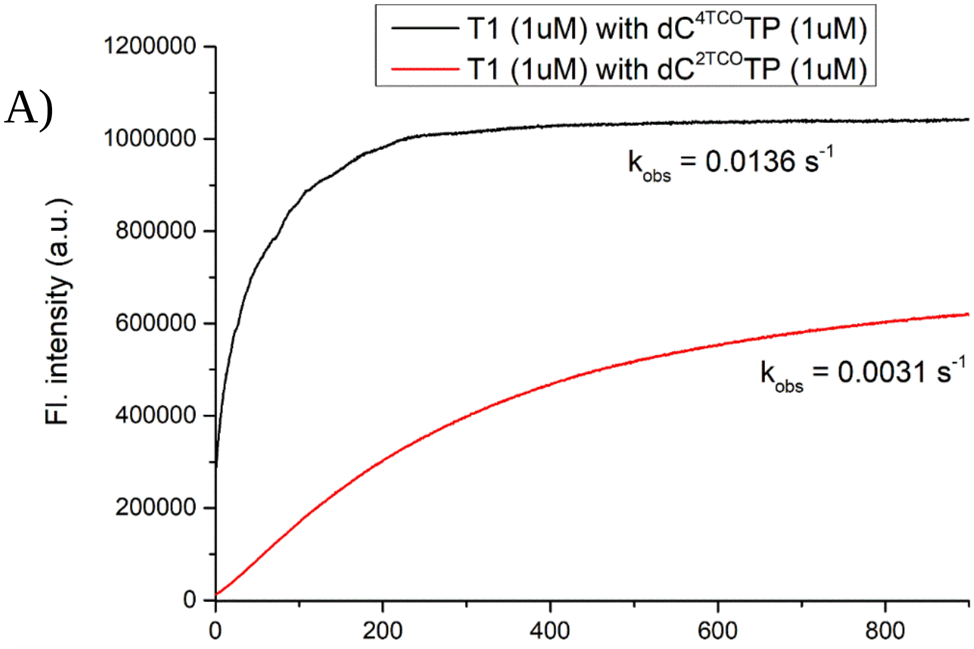
T1 (1uM) with dC4TCOTP (1uM)
T1 (1uM) with dC2TCOTP (1uM)
k obs = 0.0136 s-1
k obs = 0.0031 s-1
Fl. intensity (a.u.)
1200000
1000000
800000
600000
400000
200000
0
0
200
400
600
800

B)

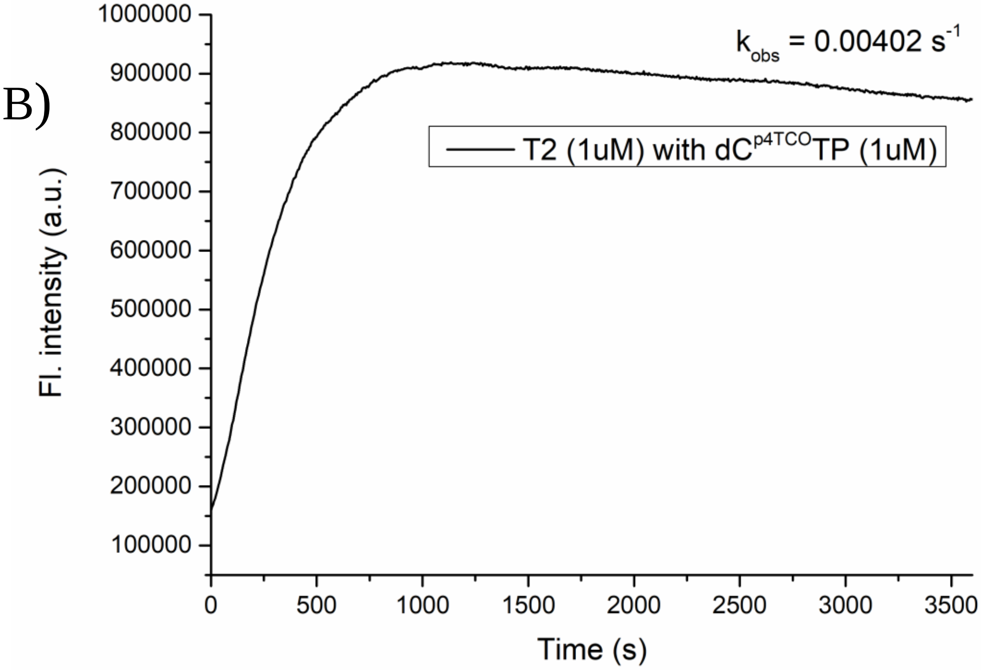
k obs = 0.00402 s-1
T2 (1uM) with dCp4TCOTP (1uM)
Fl. intensity (a.u.)
1000000
900000
800000
700000
600000
500000
400000
300000
200000
100000
0
500
1000
1500
2000
2500
3000
3500
Time (s)

C)

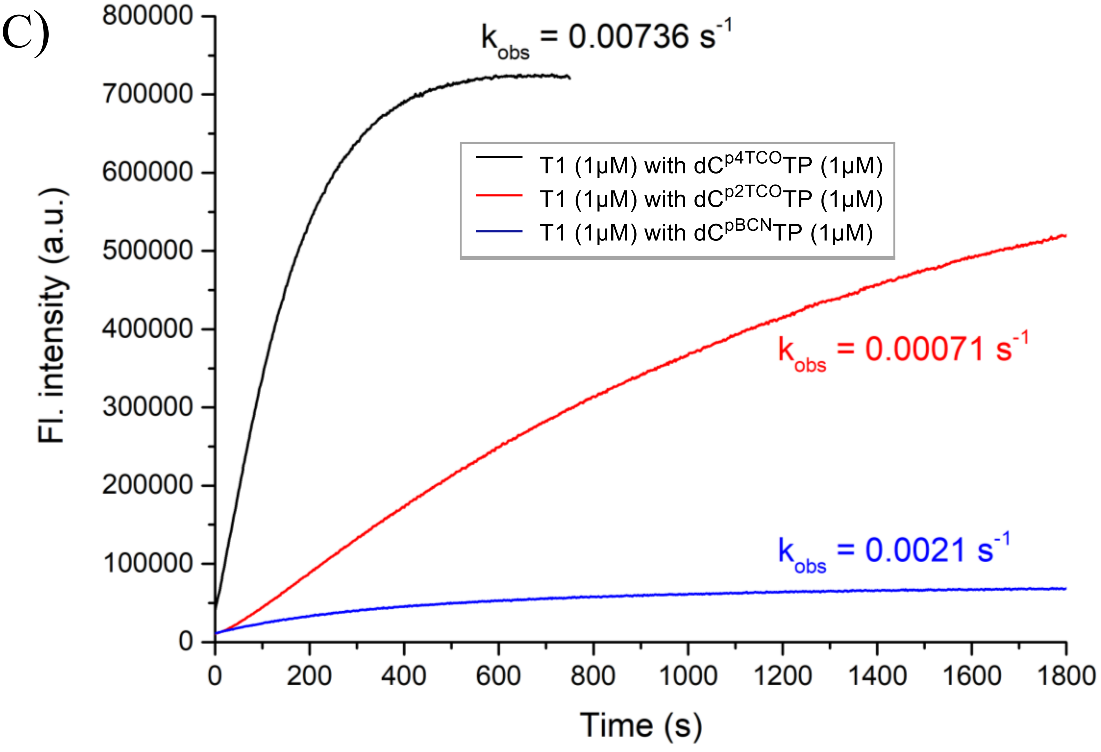

Figure S10. Fluorescence time lapse measurements showing changes in the fluorescence signal of A) the click products over 30 min for **T1** or B) 1h for **T2**. A) The fluorescence intensities were finally plotted as the function of time showing that **dC^{4TCO}TP** reacts with **T1** 4.5x faster than **dC^{2TCO}TP** Excitation was set to 370 nm (slit 3) and the fluorescence was collected at 485 nm (slit 3) over 30 minutes (interval time 0.5 s) or 1h for **T2** (interval time 2s). The data were subtracted from the background fluorescence signal of PBS, C) Fluorescence time-lapse measurements of **dC^{p4TCO}TP** or **dC^{p2TCO}TP** or **dC^{pBCN}TP** with **T1** tetrazine showing changes in the fluorescence signal of the click products in time. The observed rate constants were calculated by fitting the curves with single exponential equation using the Origin Pro software.

Table S6. Fluorescence intensities at half-life of reactions between **dC^{p4TCO}TP** or **dC^{p2TCO}TP** or **dC^{pBCN}TP** and **T1**.

	**Fl intensity dC^{p4TCO}TP** (a.u.)	**Fl intensity dC^{p2TCO}TP** (a.u.)	**Fl intensity dC^{pBCN}TP** (a.u.)
$t_{1/2}$ **T1** with **dC^{p4TCO}TP** = 135 s	422000	-	-
$t_{1/2}$ **T1** with **dC^{p2TCO}TP** = 1408 s	-	460000	-
$t_{1/2}$ **T1** with **dC^{pBCN}TP** = 475 s	-	-	48600

4. Reaction of 19DNA_C^{4TCO} or 19DNA_C^{2TCO} or 19DNA_C^{p4TCO} or 19DNA_C^{p2TCO} or 19DNA_C^{pBCN} with tetrazines

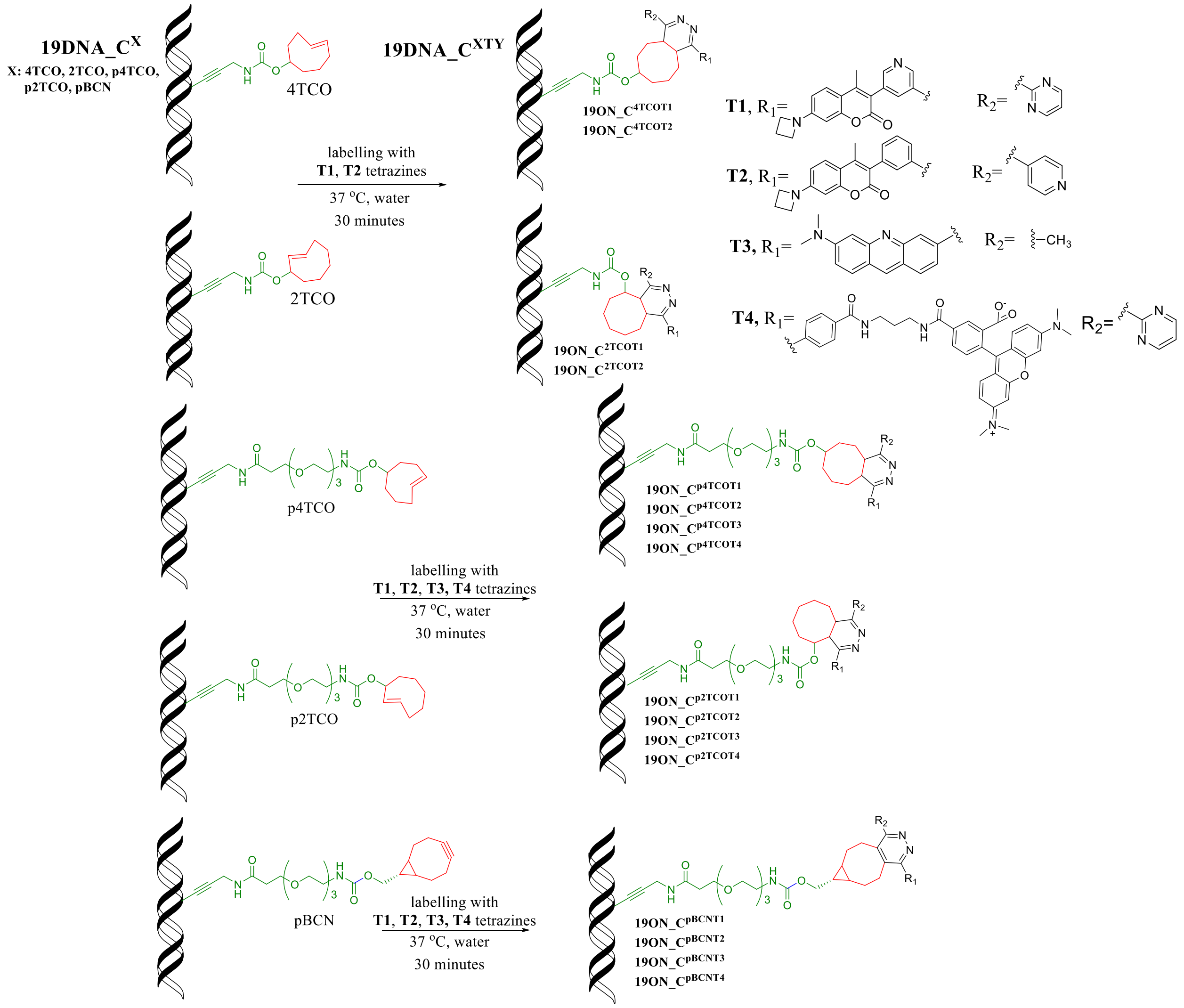

Scheme S3. Reaction of modified DNA with **T1**, **T2**, **T3**, **T4** tetrazines. Only one isomer of the click product is shown for clarity.

4.1. Reaction of 19DNA_C^X (X = 4TCO or 2TCO or p4TCO or p2TCO or pBCN) with T1 or T2 tetrazines and of 19DNA_C^X (X = p4TCO or pBCN) with T4 tetrazine

19DNA_C^{natural} and **19DNA_C^X** were prepared in semi-preparative scale using 6-FAM-labelled primer as described above (Section 2.2.2) and firstly used as negative controls (without treatment with **T1**, **T2**, **T4**) and then modified **19DNA_C^X** were used for post-synthetic labelling with **T1**, **T2** and **T4** tetrazines. The crude mixture for each **19DNA_C^X**, after PEX, was purified by QIAGEN column and

subsequently **T1** or **T2** or **T4** tetrazine (each either 10 µM or 100 µM in 5 µL DMSO) was added to the purified **19DNA_C^{X}** (0.4 µM in 5 µL H_2O). The reaction (10 µL) with final concentration of **19DNA_C^{X}** 0.2 µM and of **T1** or **T2** or **T4** tetrazine 5 µM or 50 µM was incubated for 30 min at 37 °C in a thermal cycler. After incubation with tetrazine, the reaction was purified again by QIAGEN column to remove the excess of unreacted tetrazine. PAGE stop solution (10 µL) was added to the purified reaction (10 µL) and samples were analysed by 12.5% denaturing PAGE and visualized using fluorescence imaging (Figure S12 – click reaction with **T1**, Figure S13 – click reaction with **T2**, Figure S14 – click reaction with **T4**). To explain multiple bands observed in Figure S12-A (lines 6,7) after click reaction between **19ON_C^{2TCO}** and **T1** (as described above), LC-MS analysis was performed and several isomers of click product were observed (Figure S11-B) and confirmed by the same mass (Figure S11-D,E,F). For deconvoluted ESI-MS spectra Unidec (Universal Deconvolution of Mass and Ion mobility spectra) software[7] was used. Next, the reaction mixture (**19ON_C^{2TCO}** after click reaction with **T1)** was purified on semipreparative scale by analytical HPLC. Several compounds were isolated and analysed on denaturing PAGE gel (Figure S12-B).

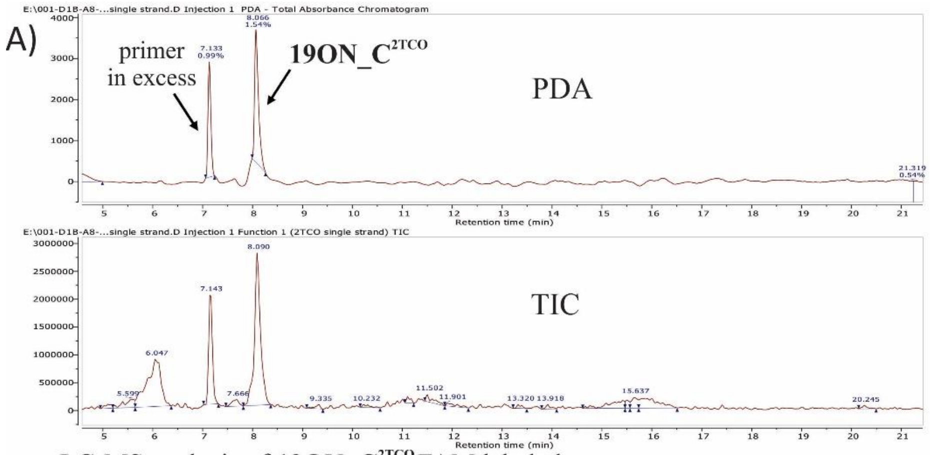

LC-MS analysis of **19ON_C^{2TCO}** FAM labeled

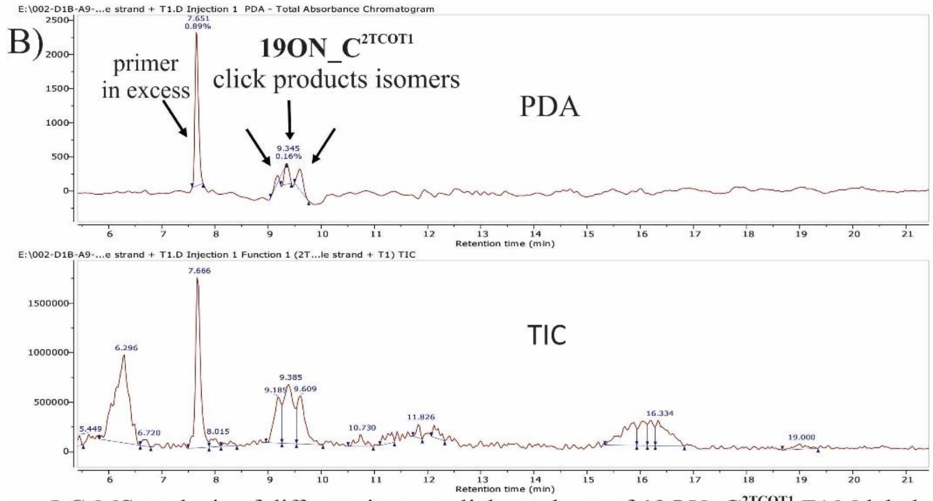

LC-MS analysis of different isomer click products of **19ON_C^{2TCOT1}** FAM labeled

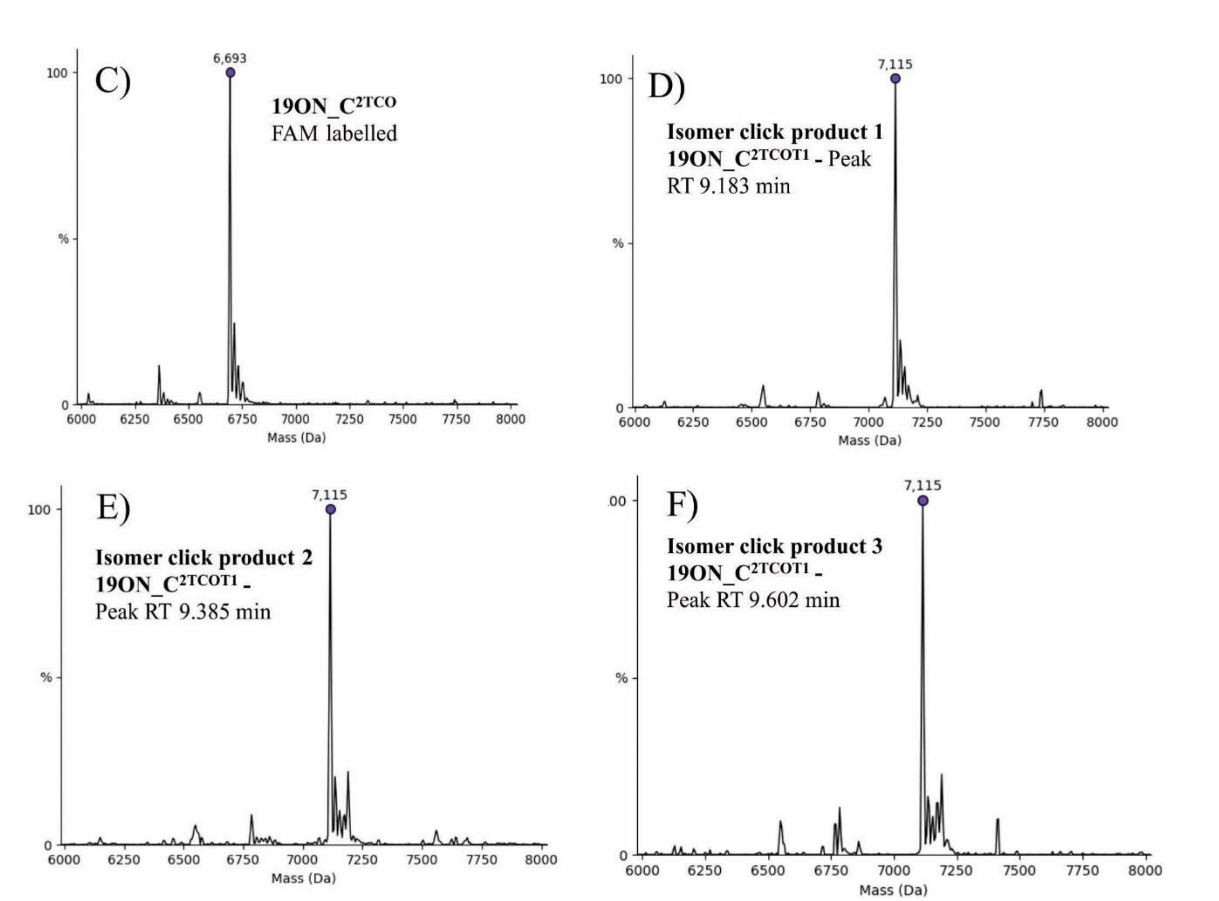

Figure S11. A) LC-MS analysis of **19ON_C^{2TCO}** FAM labelled, B) LC-MS analysis of isomer click products **19ON_C^{2TCOT1}** FAM labelled, C) ESI-MS(-) spectrum of **19ON_C^{2TCO}** FAM labelled, calculated [M] 6693.03 Da; found 6693 Da, D), E), F) ESI-MS(-) spectrum of isomer click products. **19ON_C^{2TCOT1}** FAM labelled, calculated [M] 7115.18 Da; found 7115 Da.

A)

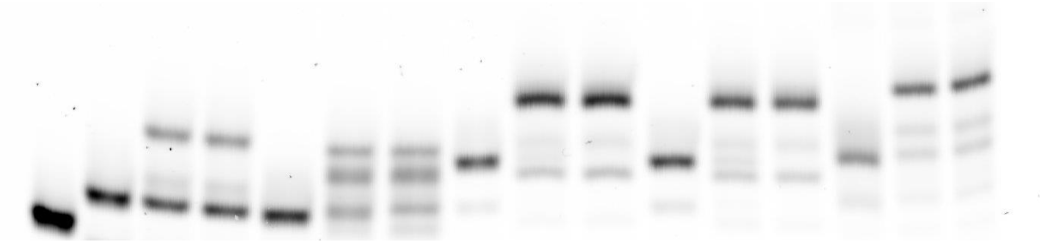

	1	2	3	4	5	6	7	8	9	10	11	12	13	14	15	16
**19DNA^{natural}**	+	-	-	-	-	-	-	-	-	-	-	-	-	-	-	-
**19DNA_C^{4TCO}**	-	+	+	+	-	-	-	-	-	-	-	-	-	-	-	-
**19DNA_C^{2TCO}**	-	-	-	-	+	+	+	-	-	-	-	-	-	-	-	-
**19DNA_C^{p4TCO}**	-	-	-	-	-	-	-	+	+	+	-	-	-	-	-	-
**19DNA_C^{p2TCO}**	-	-	-	-	-	-	-	-	-	-	+	+	+	-	-	-
**19DNA_C^{pBCN}**	-	-	-	-	-	-	-	-	-	-	-	-	-	+	+	+
**T1 (5 µM)**	-	-	+	-	-	+	-	-	+	-	-	+	-	-	+	-
**T1 (50 µM)**	-	-	-	+	-	-	+	-	-	+	-	-	+	-	-	+

B)

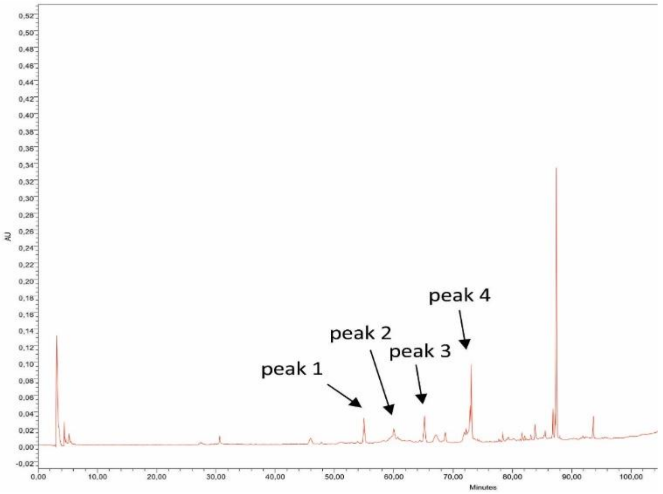

Chromatogram of click reaction between **19ON_C^{2TCO}** semipreparative scale and **T1** after analytical HPLC purification.

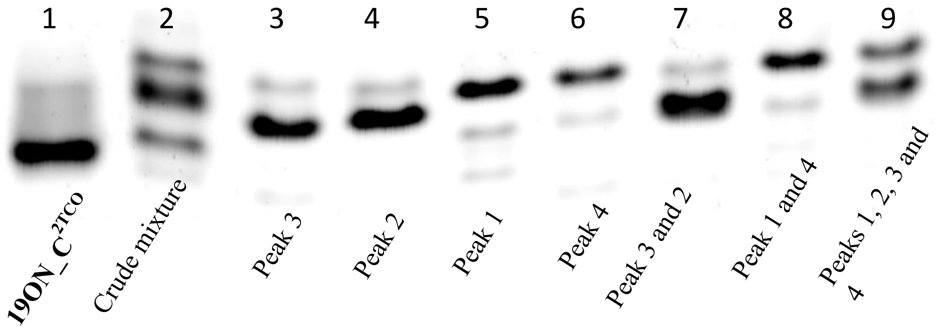

Figure S12. A) Denaturing PAGE analysis of tetrazine-DNA click reaction of **19DNA_C^{4TCO}** with **T1** (10 µM) lane 3 or **T1** (100 µM) lane 4; of **19DNA_C^{2TCO}** with **T1** (10 µM) lane 6 or **T1** (100 µM) lane 7; of **19DNA_C^{p4TCO}** with **T1** (10 µM) lane 9 or **T1** (100 µM) lane 10; of **19DNA_C^{p2TCO}** with **T1** (10 µM) lane 12 or **T1** (100 µM) lane 13; of **19DNA_C^{pBCN}** with **T1** (10 µM) lane 15 or **T1** (100 µM) lane 16. Reaction conditions: **19DNA_C^{X}** in water and **T1** tetrazine in DMSO incubated at 37 °C, 30 minutes. Negative controls (-): **19DNA^{natural}** lane 1, **19DNA_C^{4TCO}** lane 2, **19DNA_C^{2TCO}** lane 5, **19DNA_C^{p4TCO}** lane 8, **19DNA_C^{p2TCO}** lane 11, **19DNA_C^{pBCN}** lane 14.
B) Chromatogram of semipreparative scale click reaction of **19ON_C^{2TCO}** with **T1** after analytical HPLC purification and denaturing PAGE analysis of three isomer click products of **19ON_C^{2TCOT1}**. In line 7 peak 3 and 2 were loaded together and they have shown slightly different mobility shift on gel (not 100 % overlapped). In line 8 peak 1 and 4 have shown the identical mobility on gel. In line 9 all peaks were mixed together.

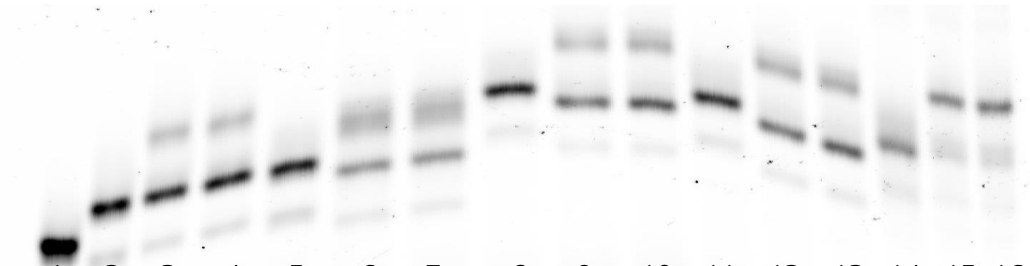

	1	2	3	4	5	6	7	8	9	10	11	12	13	14	15	16
**19DNA^{natural}**	+	-	-	-	-	-	-	-	-	-	-	-	-	-	-	-
**19DNA_C^{4TCO}**	-	+	+	+	-	-	-	-	-	-	-	-	-	-	-	-
**19DNA_C^{2TCO}**	-	-	-	-	+	+	+	-	-	-	-	-	-	-	-	-
**19DNA_C^{p4TCO}**	-	-	-	-	-	-	-	+	+	+	-	-	-	-	-	-
**19DNA_C^{p2TCO}**	-	-	-	-	-	-	-	-	-	-	+	+	+	-	-	-
**19DNA_C^{pBCN}**	-	-	-	-	-	-	-	-	-	-	-	-	-	+	+	+
**T2 (5 µM)**	-	-	+	-	-	+	-	-	+	-	-	+	-	-	+	-
**T2 (50 µM)**	-	-	-	+	-	-	+	-	-	+	-	-	+	-	-	+

Figure S13. Denaturing PAGE analysis of tetrazine-DNA click reaction of **19DNA_C^{4TCO}** with **T2** (10 µM) lane 3 or **T2** (100 µM) lane 4; of **19DNA_C^{2TCO}** with **T2** (10 µM) lane 6 or **T2** (100 µM)

lane 7; of **19DNA_C^{p4TCO}** with **T2** (10 µM) lane 9 or **T2** (100 µM) lane 10; of **19DNA_C^{p2TCO}** with **T2** (10 µM) lane 12 or **T2** (100 µM) lane 13; of **19DNA_C^{pBCN}** with **T2** (10 µM) lane 15 or **T2** (100 µM) lane 16. Reaction conditions: **19DNA_C^{X}** in water and **T2** tetrazine in DMSO incubated at 37 °C, 30 minutes. Negative controls (-): **19DNA^{natural}** lane 1, **19DNA_C^{4TCO}** lane 2, **19DNA_C^{2TCO}** lane 5, **19DNA_C^{p4TCO}** lane 8, **19DNA_C^{p2TCO}** lane 11, **19DNA_C^{pBCN}** lane 14.

Scanned at 532 nm

(T4- λ_{ex} TAMRA)

Scanned at 473 nm

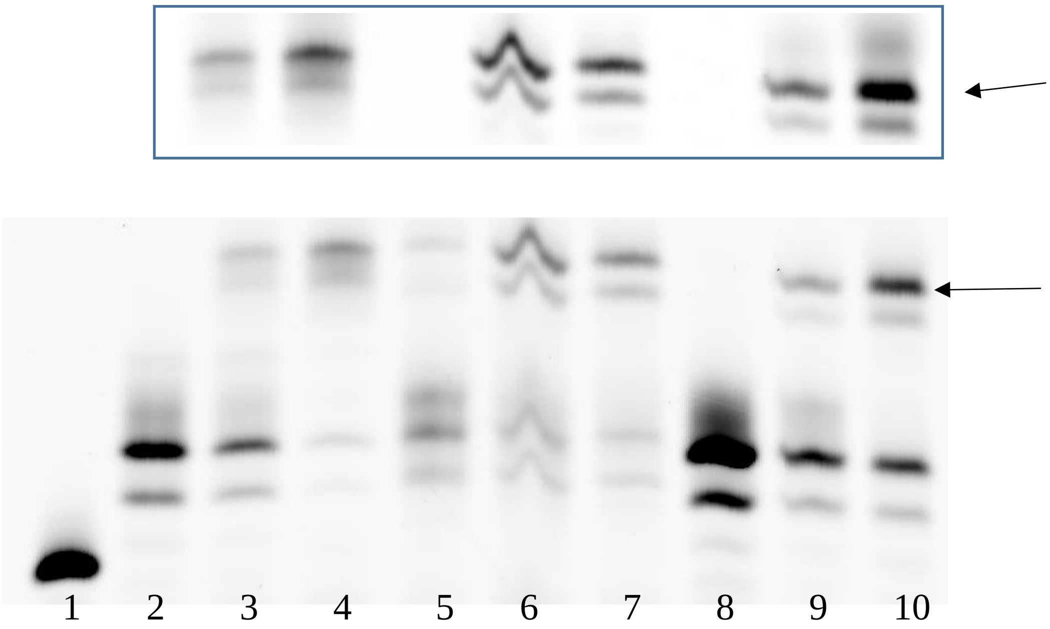

	1	2	3	4	5	6	7	8	9	10
**19DNA^{natural}**	+	-	-	-	-	-	-	-	-	-
**19DNA_C^{p4TCO}**	-	+	+	+	-	-	-	-	-	-
**19DNA_C^{pBCN}**	-	-	-	-	+	+	+	-	-	-
**19DNA_C^{p2TCO}**	-	-	-	-	-	-	-	+	+	+
**T4 (5 µM)**	-	-	+	-	-	+	-	-	+	-
**T4 (50 µM)**	-	-	-	+	-	-	+	-	-	+

Figure S14. Denaturing PAGE analysis of tetrazine-DNA click reaction of **19DNA_C^{p4TCO}** with **T4** (10 µM) lane 3 or **T4** (100 µM) lane 4; of **19DNA_C^{pBCN}** with **T4** (10 µM) lane 6 or **T4** (100 µM) lane 7; of **19DNA_C^{p2TCO}** with **T4** (10 µM) lane 9 or T4 (100 µM) lane 10. Reaction conditions: **19DNA_C^{X}**

in water and **T4** tetrazine in DMSO incubated at 37 °C, 30 minutes. Negative controls (-): **19DNA^{natural}** lane 1, **19DNA_C^{p4TCO}** lane 2, **19DNA_C^{pBCN}** lane 5, **19DNA_C^{p2TCO}** lane 8.

4.2 Reaction of 19DNA_C^{X} (X = p4TCO or pBCN) with T3 tetrazine.

19DNA_C^{natural} and **19DNA_C^{X}** (**X** = **p4TCO** or **pBCN**) were prepared in semi-preparative scale using 6-FAM-labelled primer as described above (Section 2.2.2) and firstly used as negative controls (without treatment with **T3**) and then modified **19DNA_C^{X}** were used for post-synthetic labelling with T3 tetrazine. The crude mixture, after PEX, was purified by QIAGEN column and subsequently **T3** tetrazine (100 µM or 1 mM in 5 µL DMSO) was added to the purified **19DNA_C^{X}** (0.4 µM in 5 µL H_2O). The reaction (10 µL) with final concentration of **19DNA_C^{X}** 0.2 µM and of **T3** tetrazine 50 µM or 500 µM was incubated for 18 h (due to low reactivity) at 37 °C in a thermal cycler. After incubation with tetrazine, the reaction was purified by QIAGEN column to remove the excess of unreacted tetrazine. Then, magneto-separation was applied to have single-stranded clicked **19ON_C^{X}** products. PAGE stop solution (10 µL) was added to the purified reaction (10 µL) and samples were analysed by 12.5% or 20% NATIVE PAGE and visualized using fluorescence imaging (Figure S15-A,B). By 12.5% there was any mobility shift between modified DNA and the clicked product, even though all click products were confirmed by MALDI (See Table S7). Instead, samples analysed by 20% NATIVE PAGE were easily distinguished by different mobility shift (Figure S15-B).

A)

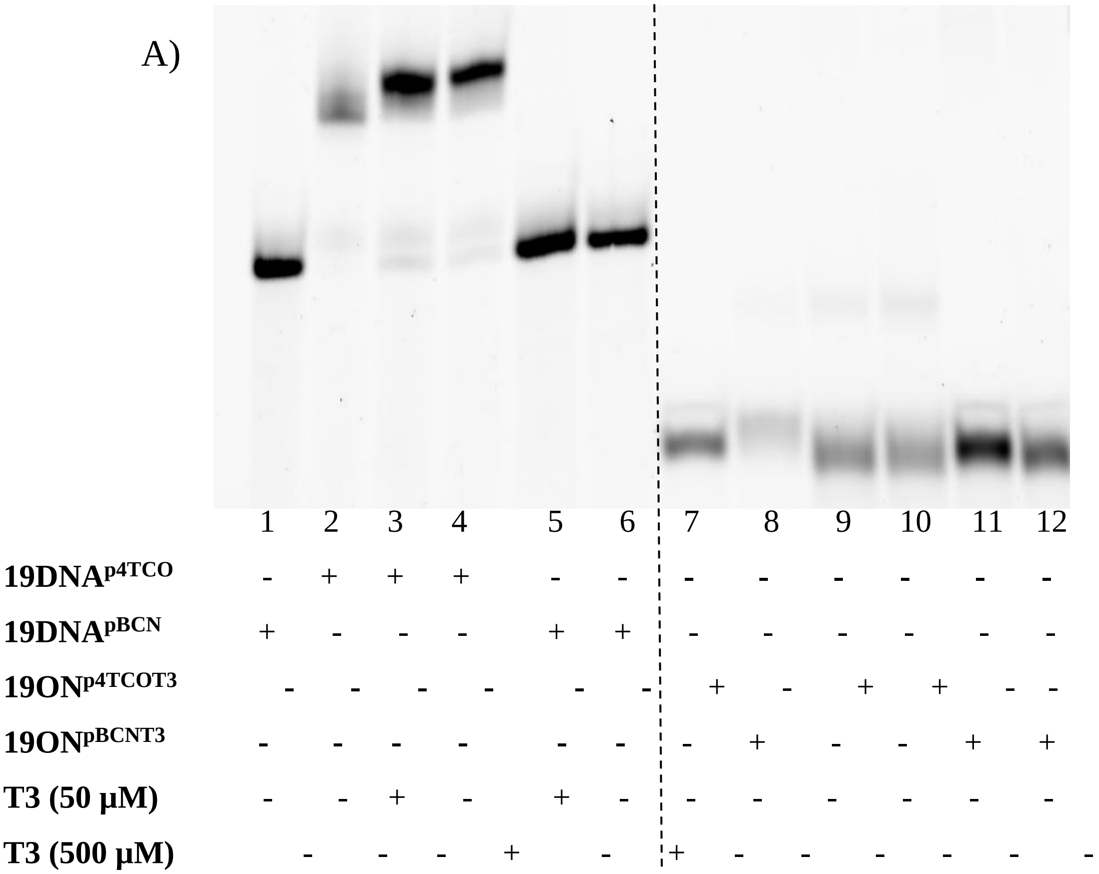
1 2 3 4 5 6 7 8 9 10 11 12
19DNAp4TCO - + + + - - - - - - - -
19DNApBCN + - - - + + - - - - - -
19ONp4TCOT3 - - - - - - + - + + - -
19ONpBCNT3 - - - - - - - + - - + +
T3 (50 µM) - - + - + - - - - - - -
T3 (500 µM) - - - + - + - - - - - -

B)

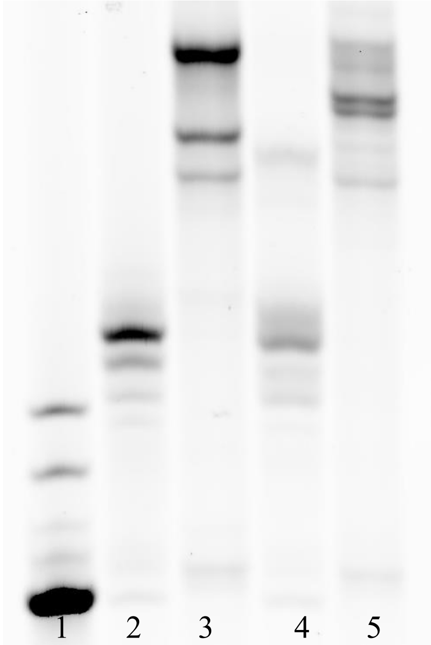

**19DNA**^{natural}	+	-	-	-	-
**19DNA**^{p4TCO}	-	+	+	-	-
**19DNA**^{pBCN}	-	-	-	+	+
**T3 (500 µM)**	-	-	+	-	+

Figure S15. A) 12.5% NATIVE PAGE analysis about the formation of tetrazine-DNA click products between **19DNA_C**^{p4TCO} with **T3** tetrazine tested at 100 µM (lane 3) or 1 mM (lane 4) concentration; **19DNA_C**^{pBCN} with **T3** tetrazine tested at 100 µM (lane 5) or at 1 mM (lane 6) concentration compared to single-stranded tetrazine-ON click products **19ON_C**^{p4TCOT3} (lanes 9,10); **19ON_C**^{pBCNT3} (lanes 11,12) produced by magneto-separation. Controls: **19DNA_C**^{pBCN} (lane 1), **19DNA_C**^{p4TCO} (lane 2), **19ON_C**^{p4TCO} (lane 7), **19ON_C**^{pBCN} (lane 8).

B) 20% NATIVE PAGE analysis about the formation of tetrazine-DNA click products between **19DNA_C**^{p4TCO} with **T3** tested at 1 mM (lane 3); **19DNA_C**^{pBCN} with **T3** tetrazine tested at 1 mM (lane 5). Controls: **19DNA_C**^{natural} (lane 1), **19DNA_C**^{p4TCO} (lane 2), **19ON_C**^{pBCN} (lane 4).

4.3. Formation of tetrazine-ONs click products (19ON_C^{XTY}) - MALDI-TOF analysis.

Using **Temp19_1C_2xbio** and **primer PrimA**, **19DNA_C^{4TCO}**, **19DNA_C^{2TCO}**, **19DNA_C^{p4TCO}** and **19DNA_C^{p2TCO}** or **19ON_C^{pBCN}** were prepared by PEX in semi-preparative scale as described above (Section 2.2.2). The reaction mixture (20 µL) containing **19DNA_C^{4TCO}** or **19DNA_C^{2TCO}** or **19DNA_C^{p4TCO}** or **19DNA_C^{p2TCO}** or **19ON_C^{pBCN}** (0.5 µM in water) and tetrazine (**T1** or **T2**, **T3**[6] or **T4**, each 100 µM in DMSO) were stirred at 37 °C for 30 min, except for **T3** which was kept overnight due to lower reactivity. Subsequently, the products were purified by magneto-separation, as described above (Section 2.3) to get **19ON_C^{4TCOT1}** or **19ON_C^{4TCOT2}**, **19ON_C^{2TCOT1}** or **19ON_C^{2TCOT2}**, **19ON_C^{p4TCOT1}** or **19ON_C^{p4TCOT2}** or **19ON_C^{p4TCOT3}** or **19ON_C^{p4TCOT4}**, **19ON_C^{p2TCOT1}** or **19ON_C^{p2TCOT2}**, **19ON_C^{pBCNT1}** or **19ON_C^{pBCNT2}** or **19ON_C^{pBCNT3}** or **19ON_C^{pBCNT4}** tetrazine-linked products. The resulted ONs were analysed by MALDI-TOF mass spectrometry (See Table S7 and copies of mass spectra in Section 8).

Table S7. MALDI data of tetrazine-linked modified nucleic acids adducts

Nucleic acids	Mw calculated [Da]	Mw found [Da]	Δ [Da]	Figure Number
**19ON_C^{4TCOT2}**	6576.1	6577.1[b]	1.0	Figure S51
**19ON_C^{4TCOT1}**	6578.1	6578.2	0.02	Figure S52
**19ON_C^{2TCOT1}**	6578.1	6580.4	2.3	Figure S54
**19ON_C^{2TCOT2}**	6576.1	6579.5	3.4	Figure S55
**19ON_C^{p4TCOT1}**	6781.2	6782.4 [b]	1.2	Figure S57
**19ON_C^{p4TCOT2}**	6779.3	6780.4 [b]	1.1	Figure S58
**19ON_C^{p2TCOT1}**	6781.2	6782.8 [b]	1.6	Figure S60
**19ON_C^{p2TCOT2}**	6779.3	6780.6 [b]	1.3	Figure S61
**19ON_C^{pBCNT1}**	6805.3	6804.8	0.5	Figure S63
**19ON_C^{pBCNT2}**	6803.3	6803.2	0.1	Figure S64
**19ON_C^{p4TCOT4}**	7077.4	7078.9	1.5	Figure S65
**19ON_C^{pBCNT4}**	7103.4	7103.2	0.2	Figure S66
**19ON_C^{pBCNT3}**	6671.2	6670.8 [b]	0.5	Figure S67
**19ON_C^{p4TCOT3}**	6647.2	6646.7	0.6	Figure S68
**19DNA_C^{p4TCOT3}** [a]	7184.2	7184.4 [b]	0.2	Figure S73
**19ON_C^{p4TCOT3}** [a]	7184.4	7185.6 [b]	1.2	Figure S74
**19DNA_C^{pBCNT3}** [a]	7208.2	7209.9 [b]	1.7	Figure S75
**19ON_C^{pBCNT3}** [a]	7208.2	7209.4 [b]	1.2	Figure S76

[a] 5`-(6-FAM)-labelled, ON^{X}-single stranded DNA, DNA^{X}-double strand DNA. [b] Nucleic acids were measured in positive mode.

5. Oligonucleotide fluorescence turn-on measurement of 19ON_C^{p4TCO} or 19ON_C^{pBCN} with T1 or T3 tetrazine (19ON_C^{XTY})

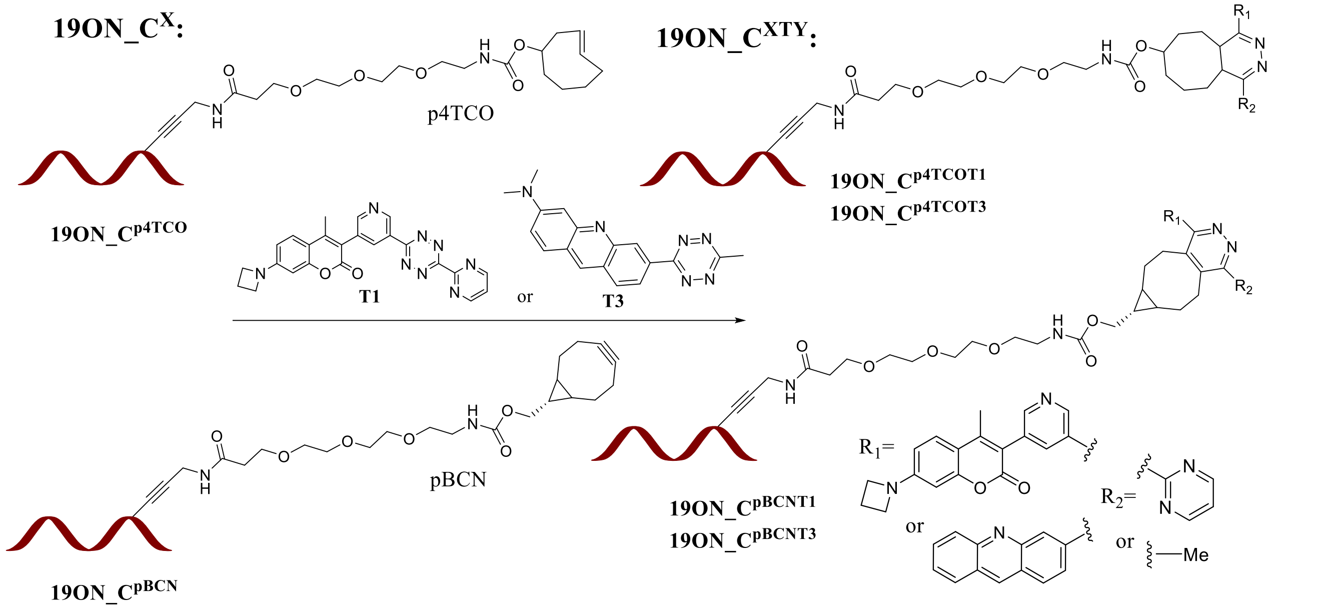

Scheme S4. Reaction scheme of the click product formed in the reaction of **19ON_C^{p4TCO}** or **19ON_C^{pBCN}** with **T1** or **T3** tetrazine. Only one isomer of the click product is shown for clarity.

19ON_C^{p4TCO} and **19ON_C^{pBCN}** were prepared in semi-preparative scale as described in Section 2.2.2 using template **19ON_C_2xbio** and primer **PrimA**, then magneto-separation was applied. Freshly prepared stock solution of the tetrazine **T1** (0.1 mM in DMSO) was used for the measurement. The turn-on fluorescence measurements were performed as follows:

1 µL of the tetrazine stock solution (0.1 mM in DMSO) was diluted in 100 µL PBS buffer (1 X, pH 7.4, giving 1 µM final concentration) and the fluorescence was measured using 370 nm excitation (slit 3) and 400-650 nm for emission. To this solution was added solution of the **19ON_C^{p4TCO}** or **19ON_C^{pBCN}** (5.5 µL from 18 µM fresh stock in water, giving 1 µM final concentration, 1 equiv. to

T1 tetrazine). The spectra were recorded at several time points (final measurements after 30 minutes) and all measurements were typically repeated two-three times.

Due to the lower reactivity of the **T3** tetrazine, the fluorescence measurements were performed as follows: 5.5 µL of 18 µM solution of **19ON_C$^{\mathbf{p4TCO}}$** or 7 µL of 14 µM solution of **19ON_C$^{\mathbf{pBCN}}$** was combined with 1 µL of 0.1 mM solution of **T3** tetrazine and the reaction mixtures were incubated in the dark at room temperature for 3 hours. Then, the reaction mixtures were diluted to 100 µL with PBS and the fluorescence was measured using 500 nm excitation wavelength and 525-750 nm emission window (slit 3).

The cuvette was inserted into the fluorescence spectrophotometer and the measurement was started. The spectra were recorded at several time points (final measurements after 30 minutes) and all measurements were typically repeated two-three times. The data were processed using OriginPro software. All spectra were subtracted from the baseline (PBS buffer as the blank). The turn-on values were calculated from the observed fluorescence intensities of the click products at the emission maximum (475 nm) after ca. 30 min of reaction divided by the highest residual fluorescence of the quenched coumarin tetrazine dye at the same wevelength (475 nm). The fluorescence turn-on values of the click products formed in the reaction of **19ON_C$^{\mathbf{p4TCO}}$** and **19ON_C$^{\mathbf{pBCN}}$** with **T1** tetrazine are shown in Figures S16 and S17, respectively. The fluorescence turn-on values of the click product formed in the reaction of **19ON_C$^{\mathbf{pBCN}}$** with **T3** tetrazine is shown in Figure S18, in case of **19ON_C$^{\mathbf{p4TCO}}$** with **T3** we did not observed any turn on in the fluorescence, probably due to isomerization of the *trans*-cyclooctene.

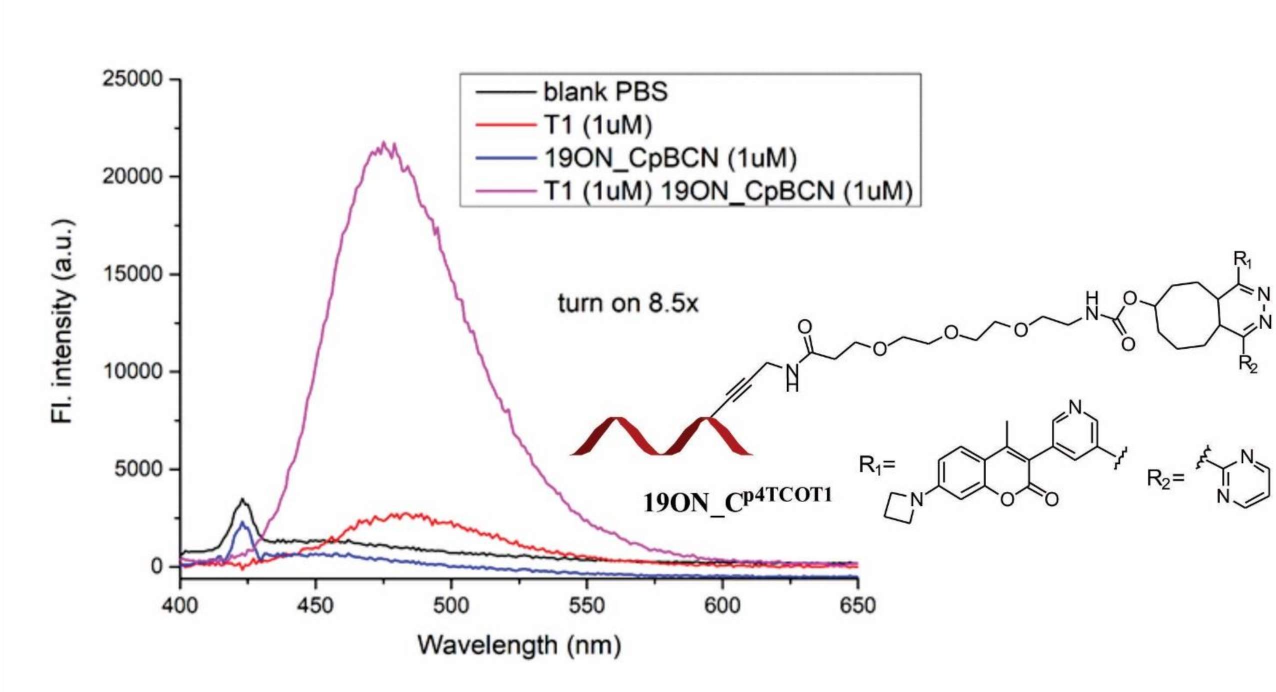

Figure S16. Fluorescence spectra of the click product formed in the reaction of tetrazine **T1** with **19ON_C^{p4TCO}** (indicated as number-x = x-fold). Fluorescence spectra of the tetrazine-linked DNA after reaction with **T1** (magenta line) are shown in relation to the respective quenched tetrazine (red line), to the **19ON_C^{p4TCO}** alone (blue line) and to PBS blank (black line) to indicate the fluorescence turn-on ratio. Only one isomer of the click product is shown for clarity.

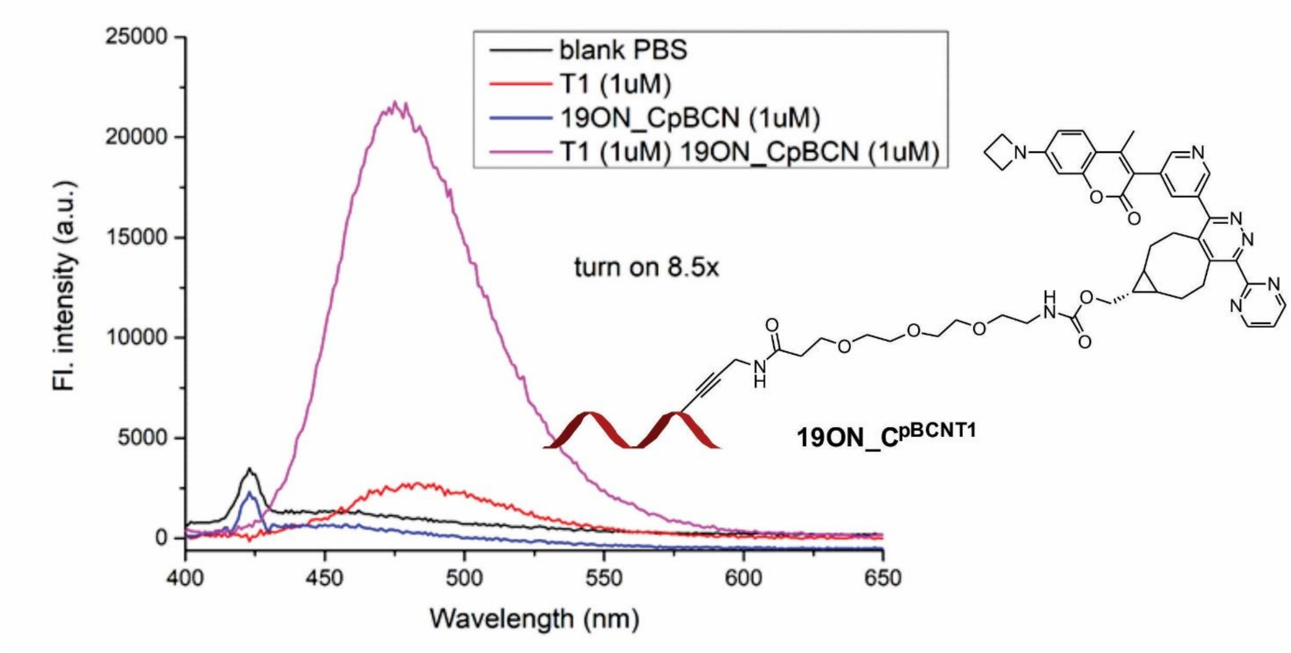

Figure S17. Fluorescence spectra of the click product formed in the reaction of **T1** tetrazine with **19ON_C^{pBCN}** (indicated as number-x = x-fold). Fluorescence spectra of the tetrazine-linked DNA after reaction with **T1** (magenta line) are shown in relation to the respective quenched tetrazine (red line), to the **19ON_C^{pBCN}** alone (blue line) and to PBS blank (black line) to indicate the fluorescence turn-on ratio.

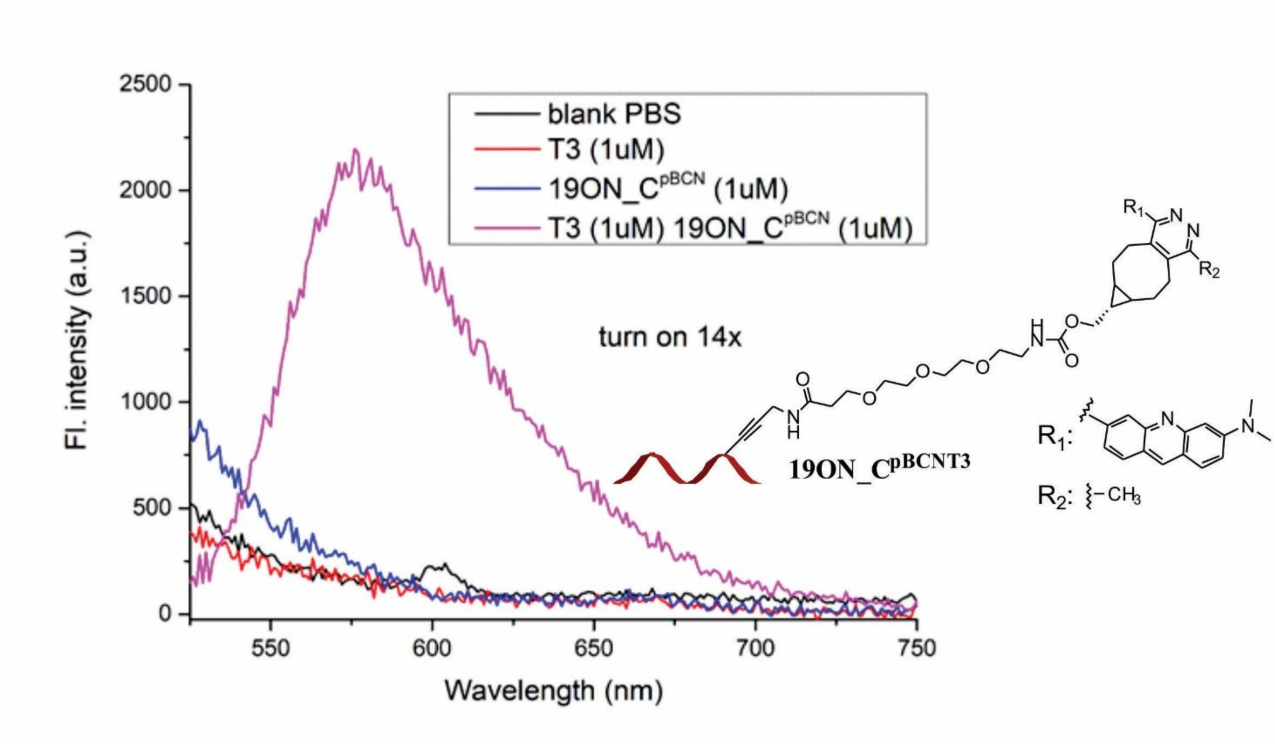

Figure S18. Fluorescence spectra of the click product formed in the reaction of **T3** tetrazine with **19ON_C^{pBCN}** (indicated as number-x = x-fold). Fluorescence spectra of the tetrazine-linked DNA after reaction with **T3** (magenta line) are shown in relation to the respective quenched tetrazine (red line), to the **19ON_C^{pBCN}** alone (blue line) and to PBS blank (black line) to indicate the fluorescence turn-on ratio.

6. Labelling of DNA using dC^{p4TCO} or dC^{p2TCO}TP or dC^{pBCN}TP derivatives delivered to U-2 OS cells by SNTT1[5]

Experimental part:

Materials

Cell cultures: U-2 OS (ATCC HTB-96)

SNTT1; Synthetic Nucleoside Triphosphate Transporter, identical with commercially available BioTracker NTP-*Transporter* molecule (Merck *SCT064*), was used as a 10 mM stock solution in tricine buffer.

L15 medium (Gibco, 21083-027)

FBS (Capricorn, cat.no. FBS-HI-12A)

GlutaMAX-I (Gibco, cat.no. 35050-038)

Tricine buffer

Preparation procedure – dissolve following compounds (cell culture quality) in 980 mL of pure DI (MilliQ) water:

Tricine (0.896 g; 5 mmol),

Glucose (2 g; 11 mmol),

NaCl (7.325 g; 125 mmol),

$CaCl_2$ anhydrous (0.200 g; 1.8 mmol),

$MgSO_4$ anhydrous (0.100 g; 0.8 mmol),

KCl (0.400 g; 5.4 mmol),

Adjust pH of the buffer to 7.40 (at 31 °C) with NaOH (1 mol/L; approx. 850 μL).

Add pure (MilliQ) water to 1000 mL.

Filter the solution through 0.22 μm sterile filter. Keep under sterile conditions at 0-4 °C.

Tetrazines **T1**, **T2** and **T3**: working solutions of **T1**, **T2** and **T3** were prepared from the corresponding stock solutions in DMSO (**T1**, **T2** - 1 mM in DMSO; **T3** - 10 mM in DMSO) by dilution with L15 complete medium to the desired concentrations prior to use.

Tetrazine **T4**: Pyrimidyl-Tetrazine-5-TAMRA (Jena Bioscience; CLK-097) was diluted with DMSO to 10 mM stock solution which was than diluted with L15 complete medium to the desired concentrations prior to use.

DNA-staining solution: Prepare stock solution of DAPI in water (1 mg/ml). Dilute this stock solution with PBS in ratio 1:100 (1µL of DAPI stock solution per 100 µL of PBS) to make the final working reagent, further referred to as "DNA-staining solution".

Instrumentation

Confocal microscope ZEISS LSM 980 fitted with an incubator and objective 40x/1.2 W C-Apochromat (Carl Zeiss Jena GmbH, Germany)

LUNA Automated Cell Counter

Labelling of DNA using dC^{p4TCO}TP or dC^{pBCN}TP and tetrazines T1 or T2 in live U2 OS cells

1) Preparation of the cell culture

U-2 OS cells (ATCC HTB-96) were cultured in a 96 Well Glass Bottom Plate (Cellvis, P96-1.5H-N) in complete medium [L-15 (Gibco, 21083-027) supplemented with 10% FBS (Capricorn, cat.no. FBS-HI-12A) and 1% GlutaMAX-I (Gibco, cat.no. 35050-038)] in an incubator (37°C, no additional CO_2) to the confluency of about 70%.

General procedure for the incorporation of dC^{X}TP (X= 2TCO, 4TCO, p2TCO, p4TCO, pBCN) into DNA of U2OS cells and subsequent labelling with a tetrazine T1 or T2

The medium was aspirated and the cells were washed with pre-warmed (37°C) tricine buffer. Then the cells were treated with 40 µL of an equimolar mixture of **dC^{X}TP** and **SNTT 1** (10 µM) in tricine buffer (37°C, 5 min). The mixture was then removed and complete L-15 medium (80 µL) was added to the cells. The cell culture was incubated in complete medium (37°C) for 60 min and then stock solution of a tetrazine (**T1** or **T2**) was added to achieve the final desired concentration (1 µM) in media. The plate with live cells was mounted onto microscope (ZEISS LSM 980) and

incubated (37°C) for a further 15-minute period. The samples were irradiated sequentially with a laser beam at 405 nm, the detector range was set to 410 – 499 nm for the coumarine detection.

Procedure for the labeling with dC^{p4TCO}TP and tetrazine T1 and subsequent staining of nuclei with DRAQ5

The medium was aspirated and the cells were washed with pre-warmed (37°C) tricine buffer. Then the cells were treated with 40 µL of an equimolar mixture of **dC^{p4TCO}TP** and **SNTT 1** (10 µM) in tricine buffer (37°C, 5 min). The mixture was then removed and complete L-15 medium (80 µL) was added to the cells. The cell culture was incubated in complete medium (37°C) for 60 min and then stock solution of tetrazine **T1** was added so that the final concentration in media was 1 µM. The plate with live cells was mounted onto microscope (ZEISS LSM 980) and incubated (37°C) for a further 10-minute period. Then DRAQ5 (2.5 µM) was added to the medium, the cells were further incubated for 5 minutes before they were imaged. The samples were irradiated sequentially with a laser beam at 405 nm, the detector range was set to 410 – 499 nm for the coumarine detection. The DRAQ5 dye was excited at 639 nm, detected at 642 – 756 nm.

Procedure for the labeling with with dC^{pBCN}TP and tetrazine T3 and subsequent staining of nuclei with HOECHST 33342

The medium was aspirated and the cells were washed with pre-warmed (37°C) tricine buffer. Then the cells were treated with 40 µL of an equimolar mixture of **dC^{pBCN}TP** and **SNTT 1** (10 µM) in tricine buffer (37°C, 5 min). The mixture was then removed and complete L-15 medium (80 µL) was added to the cells. The cell culture was incubated in complete medium (37°C) for 60 min and then stock solution of tetrazine **T3** was added to the well so that the final concentration in media was 10 µM. The plate was mounted onto microscope (ZEISS LSM 980) and further incubated (37°C; 4 hours in total); in the course of incubation the cells were imaged by confocal microscopy in 30 min periods to check for optimal incubation time. After 4 hours of incubation, HOECHST 33342 (3 µM) was added to the medium, the cells were further incubated for 5 minutes before they were imaged. The samples were irradiated sequentially with a laser beam at 405 nm, the detector range was set to 410 – 499 nm for HOECHST 33342 detection. The PINK dye was excited at 488 nm, detected at 510 – 641 nm.

Procedure for the labeling with dC^{pBCN}TP and tetrazine T4 (pyrimidyl-tetrazine-5-TAMRA) in live cells

The medium was aspirated and the cells were washed with pre-warmed (37°C) tricine buffer. Then the cells were treated with 40 µL of an equimolar mixture of **dC^{pBCN}TP** and **SNTT 1** (10 µM) in tricine buffer (37°C, 5 min). The mixture was then removed and complete L-15 medium (80 µL) was added to the cells. The cell culture was incubated in complete medium (37°C) for 60 min and then stock solution of tetrazine **T4** was added to the well so that the final concentration in media was 20 µM. The plate was mounted onto microscope (ZEISS LSM 980) and further incubated (37°C; 45 min in total); in the course of incubation the cells were imaged by confocal microscopy in 10 min periods to check for optimal incubation time. After 45 min of incubation, the samples were irradiated with a laser beam at 561 nm, the detector range was set to 534 – 690 nm.

Procedure for the labeling with dC^{pBCN}TP and tetrazine T4 (pyrimidyl-tetrazine-5-TAMRA) in fixed cells

The medium was aspirated and the cells were washed with pre-warmed (37°C) tricine buffer. Then the cells were treated with 40 µL of an equimolar mixture **dC^{pBCN}TP** and **SNTT 1** (10 µM) in tricine buffer (37°C, 5 min). The mixture was then removed and complete L-15 medium (80 µL) was added to the cells. The cell culture was incubated in complete medium (37°C) for 60 – 90 min. After this period the cells were washed with cold PBS and cold methanol (-20°C) was added. The plate was kept at 0° C for 60 min, then methanol was aspirated, the fixed cells were washed with PBS and allowed to warm up to room temperature and then stock solution of tetrazine pyrimidyl-tetrazine-5-TAMRA (2 µM) in PBS was added. The plate was mounted onto microscope (ZEISS LSM 980) and further incubated (37°C); in the course of incubation the cells were imaged by confocal microscopy in 10 min periods to check for optimal incubation time (30 – 40 min). After 30 min of incubation, HOECHST 33342 (3 µM) was added to the medium, the cells were further incubated for 5 minutes before they were imaged. The samples were irradiated sequentially with a laser beam at 405 nm, the detector range was set to 410 – 499 nm for HOECHST 33342 detection. TAMRA dye was excited at 561 nm, detected at 534 – 690 nm.

Protocol for the cell cycle progression experiment

The cells were cultured in Leibovitz's (1X) L-15 Medium, no phenol red (cat.no. 21083-027, Gibco^{TM}, Fisher Scientific, Sweeden) supplemented with 10 % Fetal bovine serum (cat. no. F9665, Sigma-Aldrich, USA). Aproximately 0.5 million of U 2-OS cells were seeded in a well of a 6-well plate (cat. no. 3046 FALCON, Becton Dickinson, USA) and cultured for 24 hours (37°C, no additional CO_2). Then the cells were washed with pre-warmed tricine buffer and treated with 500 µL an equimolar mixture **dC^{p4TCO}TP** and **SNTT 1** (10 µM) in tricine buffer (37°C, 5 min). The mixture was then removed and complete L-15 medium (2 mL) was added to the cells and the cells were incubated for 60 min (37°C, no additional CO_2). After this period, the cells were washed with PBS (phosphate buffered saline), dissociated with TripLE^{TM} (cat. no. 12604-013, Gibco^{TM}, Fisher Scientific, Sweeden) and fixed with methanol for 60 min at 4 °C as described above. Subsequently the cells were washed with PBS and incubated with 2 µM pyrimidyl-tetrazine-5-TAMRA **(T4)** in PBS for 30 minutes at 37 °C in thermoblock. The cells were then centrifuged (300 x g ; 3 min) to remove **T4** solution and PBS (0.25 mL) was added. DNA-staining solution (100 µL; see above for the preparation of working solution of DAPI) was added and the cell suspension and the cells were analyzed by flow cytometry (BD LSRFortessaTM). For TAMRA, yellow-green laser (561 nm) was used with detection filters 582/15-nm band pass. For DAPI, UV laser (355 nm) was used with detection filter 450/50-nm band pass. Flow rate was set lower than 400 events/second and 20 000 events were collected. For analysis FlowJo™ v10.6.1 (Becton, Dickinson & Company) was used.

Table S8. Labelling of DNA in live U2OS cells

tetrazine/ **dC^{X}TP**	**dC^{2TCO}TP**	**dC^{4TCO}TP**	**dC^{p2TCO}TP**	**dC^{p4TCO}TP**	**dC^{pBCN}TP**
**T1**	unspecific staining **Figure S19**	unspecific staining **Figure S22 – S23**	visible DNA foci **Figure S26**	very clearly visible DNA foci **Figure S29 – S30** **Figure 3A–3C** in the main text	visible DNA foci **Figure S35**
**T2**	unspecific staining **Figure S20**	unspecific staining **Figure S24**	unspecific staining **Figure S27**	visible DNA foci **Figure S31**	visible DNA foci **Figure S36**
**T3**	unspecific staining **Figure S21**	unspecific staining **Figure S25**	unspecific staining **Figure S28**	unspecific staining **Figure S32**	visible DNA foci **Figure S37** **Figure 3D–3F** in the main text
**T4**[1)]	n.d.	n.d.	n.d.	live cells: weak labeling of DNA foci **Figure S33** fixed cells: very clearly visible DNA foci **Figure S34**	live cells: weak labeling of DNA foci **Figure S38** fixed cells: very clearly visible DNA foci **Figure S39** **Figure 4** in the main text

1) T4 was used with both live and fixed cells.

$dC^{2TCO}TP$

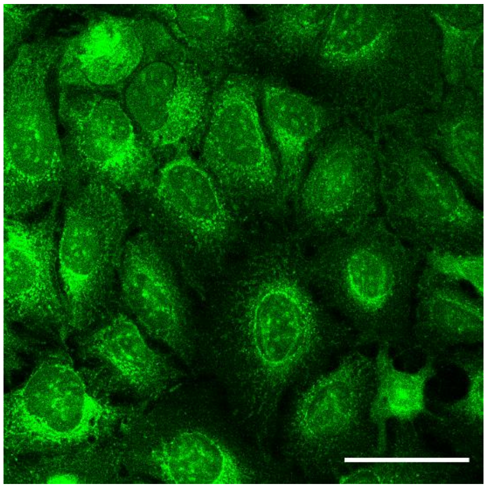

Figure S19. U2OS; **$dC^{2TCO}TP$ + T1**

Transport **$dC^{2TCO}TP$/SNTT1**: 5 min / 37° C.
Incubation in medium: 60 min / 37° C;
Labelling with **T1**: 15 min / 37° C.
Scale bar: 50 µm

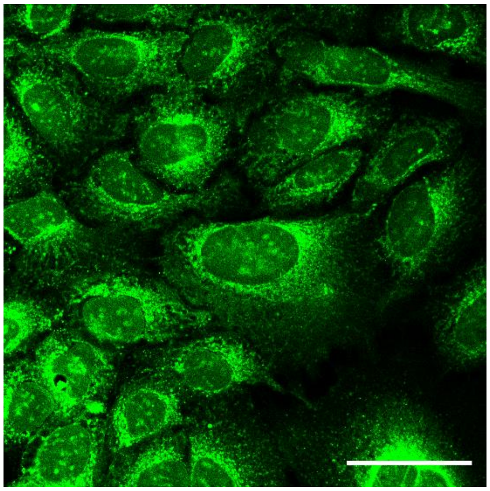

Figure S20. U2OS; **$dC^{2TCO}TP$ + T2**

Transport **$dC^{2TCO}TP$/SNTT1**: 5 min / 37° C.
Incubation in medium: 60 min / 37° C;
Labelling with **T2**: 15 min / 37° C.
Scale bar: 50 µm

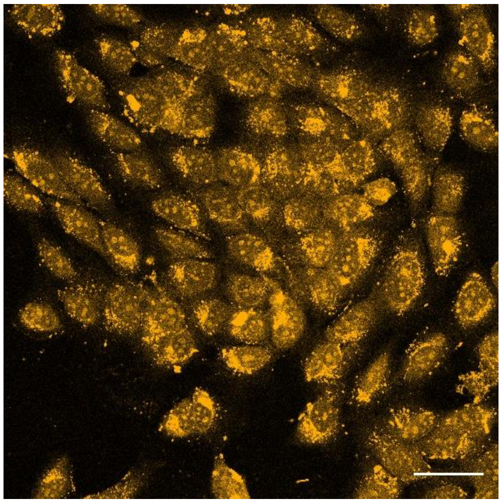

Figure S21. U2OS; **$dC^{2TCO}TP$ + T3**

Transport **$dC^{2TCO}TP$/SNTT1**: 5 min / 37° C.
Incubation in medium: 60 min / 37° C;
Labelling with **T3**: 4 hours / 37° C.
Scale bar: 50 µm

dC^{4TCO}TP

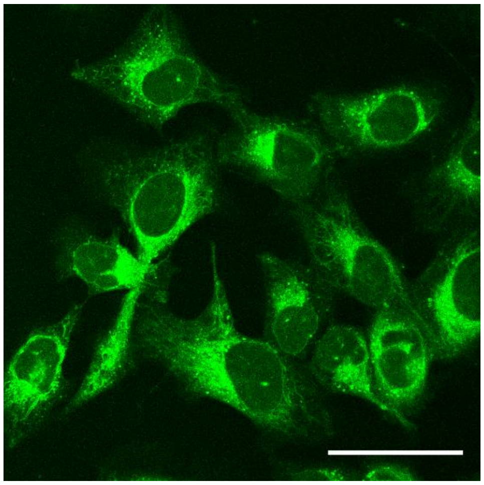

Figure S22. U2OS; **dC^{4TCO}TP** + **T1**

Transport **dC^{4TCO}TP/SNTT1**: 5 min / 37° C.
Incubation in medium: 60 min / 37° C;
Labelling with **T1**: 15 min / 37° C.
Scale bar: 50 µm

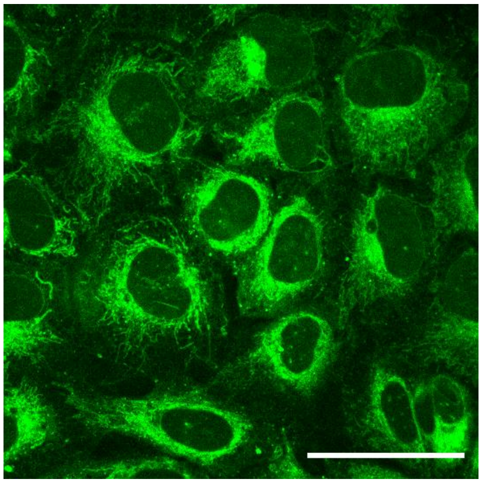

Figure S23. U2OS; + **T1**

Labelling with **T1** only (negative control experiment) 15 min / 37° C.
Scale bar 50 µm

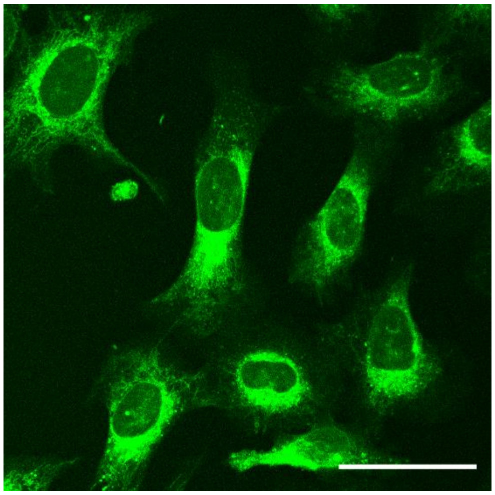

Figure S24. U2OS; **dC^{4TCO}TP** + **T2**

Transport **dC^{4TCO}TP/SNTT1**: 5 min / 37° C.
Incubation in medium: 60 min / 37° C;
Labelling with **T2**: 15 min / 37° C.
Scale bar: 50 µm

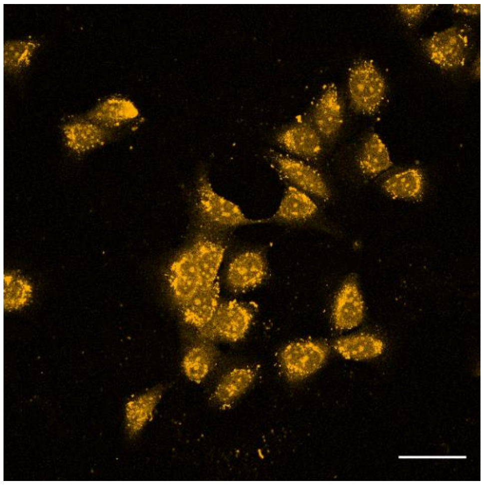

Figure S25. U2OS; **dC^{4TCO}TP** + **T3**

Transport **dC^{4TCO}TP/SNTT1**: 5 min / 37° C.
Incubation in medium: 60 min / 37° C;
Labelling with **T3**: 4 hours / 37° C.
Scale bar: 50 µm

dC^{p2TCO}TP

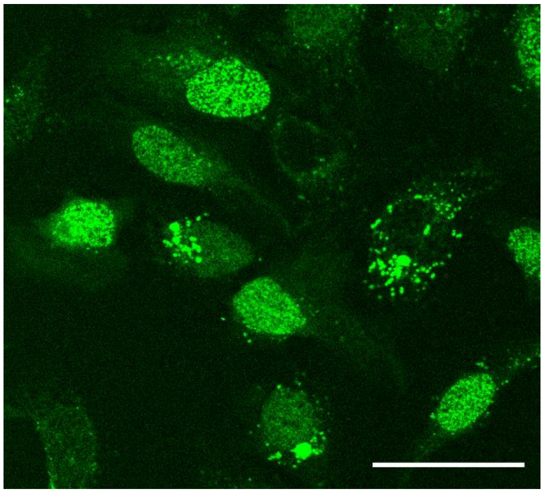

Figure S26. U2OS; **dC^{p2TCO}TP** + **T1**

Transport **dC^{p2TCO}TP** /**SNTT1**: 5 min / 37° C.
Incubation in medium: 60 min / 37° C;
Labelling with **T1**: 15 min / 37° C.
Scale bar: 50 µm

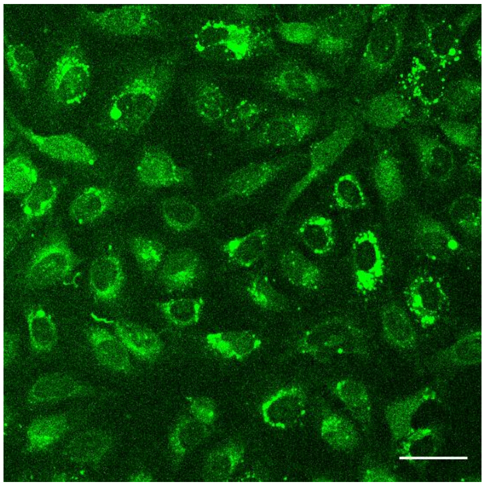

Figure S27. U2OS; **dC^{p2TCO}TP** + **T2**

Transport **dC^{p2TCO}TP** /**SNTT1**: 5 min / 37° C.
Incubation in medium: 60 min / 37° C;
Labelling with **T2**: 15 min / 37° C.
Scale bar: 50 µm

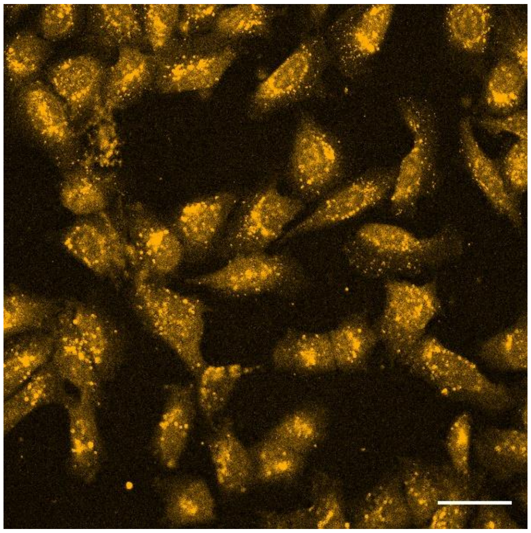

Figure S28. U2OS; **dC^{p2TCO}TP** + **T3**

Transport **dC^{p2TCO}TP** /**SNTT1**: 5 min / 37° C.
Incubation in medium: 60 min / 37° C;
Labelling with **T3**: 4 hours / 37° C.
Scale bar: 50 µm

$dC^{p4TCO}TP$

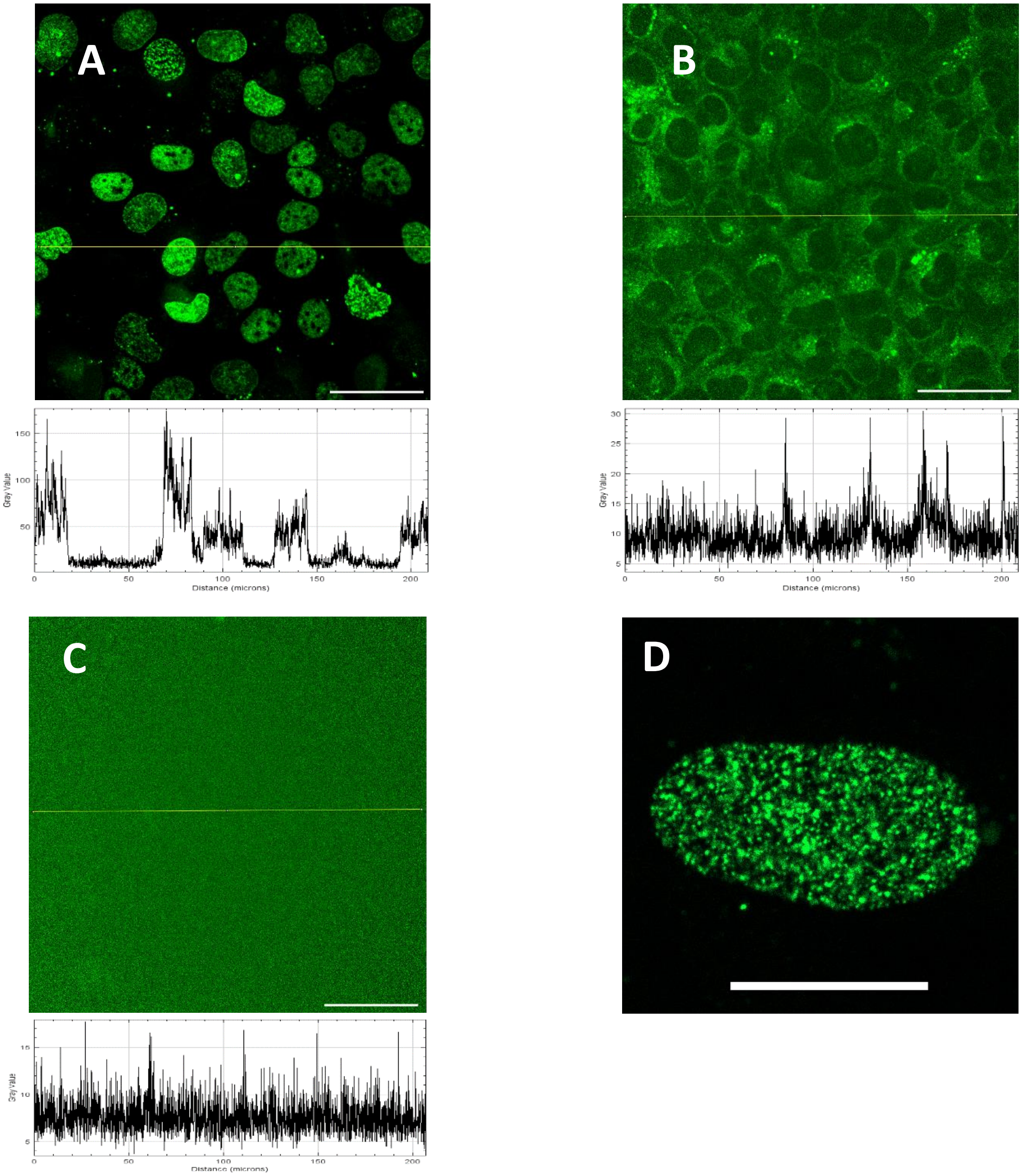

Figure S29. U2OS; **$dC^{p4TCO}TP$** + **T1** (60 minutes incubation time)
Images were acquired under identical settings. Graphs below images show intensities under the indicated yellow lines. The intensities were measured using raw files while the prints were adjusted (brightness and contrast) for better visual evaluation.
A) Transport **$dC^{p4TCO}TP$ /SNTT1,** 5 min / 37° C followed incubation in medium for 60 min / 37° C, then labelling with **T1**: 15 min / 37° C; Scale bar: 50 µm
B) Control experiment 1: Treatment of U2OS cells with **T1** only, 15 min / 37° C; Scale bar: 50 µm
C) Control experiment 2: Autofluorescence of U2OS cells; Scale bar: 50 µm
D) Zoom of U2OS cell showing DNA foci with incorporated **dC^{p4TCO}** and labeled with **T1**; Scale bar: 25 µm

dC^{p4TCO}TP

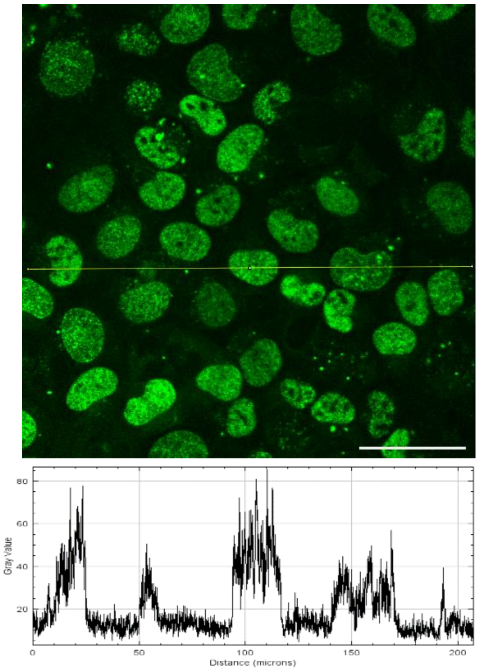

Figure S30. U2OS; **dC^{p4TCO}TP** + **T1** (6 hours incubation time)
Images were acquired under identical settings as for images in Figure S29. Graphs below images show intensities under the indicated yellow lines. The intensities were measured using raw files while the prints were adjusted (brightness and contrast) for better visual evaluation. Transport **dC^{p4TCO}TP /SNTT1,** 5 min / 37° C followed incubation in medium for 6 hours / 37° C, then labelling with **T1**: 15 min / 37° C; Scale bar: 50 µm
Control experiment 1 (**T1** only): see Figure S29B; Control experiment 2 (cell autofluorescence): see Figure S29C

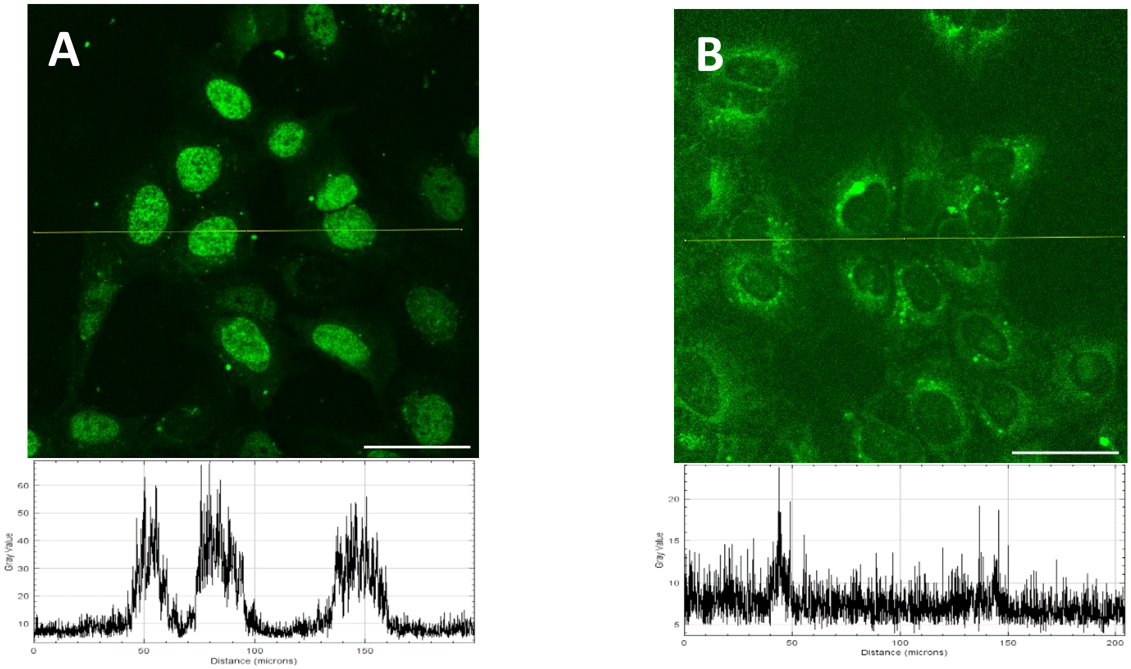

Figure S31. U2OS; **dC^{p4TCO}TP** + **T2**
Images were acquired under identical settings (the same as for images in Figure S29). Graphs below images show intensities under the indicated yellow lines. The intensities were measured using raw files while the prints were adjusted (brightness and contrast) for better visual evaluation.
A) Transport **dC^{p4TCO}TP /SNTT1,** 5 min / 37° C followed incubation in medium for 60 min / 37° C, then labelling with **T2**: 15 min / 37° C; Scale bar: 50 µm
B) Control experiment 1: Treatment of U2OS cells with **T2** only, 15 min / 37° C; Scale bar: 50 µm
Autofluorescence of cells is shown in Figure S29C.

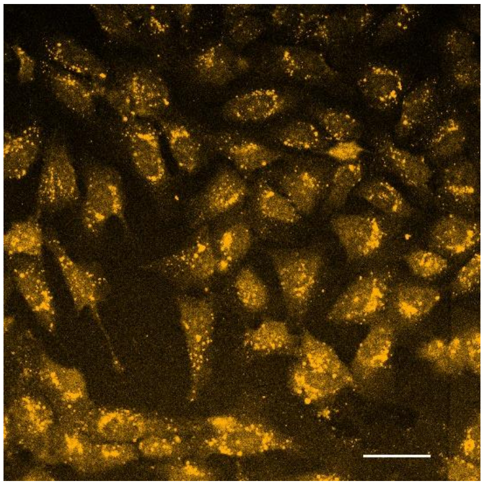

Figure S32. U2OS; **dC^{p4TCO}TP** + **T3**

Transport **dC^{p4TCO}TP /SNTT1**: 5 min / 37° C.
Incubation in medium: 60 min / 37° C;
Labelling with **T3**: 4 hours / 37° C.
Scale bar: 50 µm

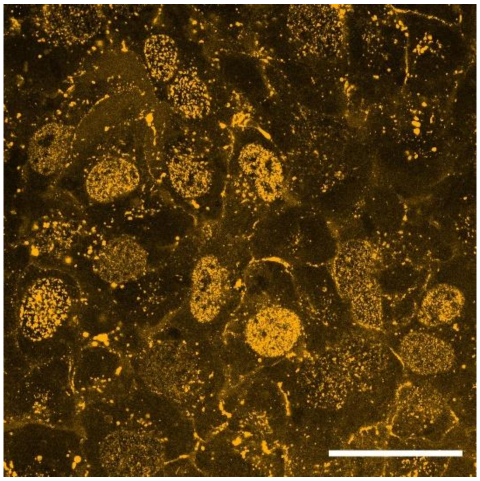

Figure S33. U2OS; **dC^{p4TCO}TP** + **T4**

Transport **dC^{p4TCO}TP /SNTT1**: 5 min / 37° C.
Incubation in medium: 60 min / 37° C;
Labelling with **T4**: 45 min / 37° C.
Scale bar: 50 µm

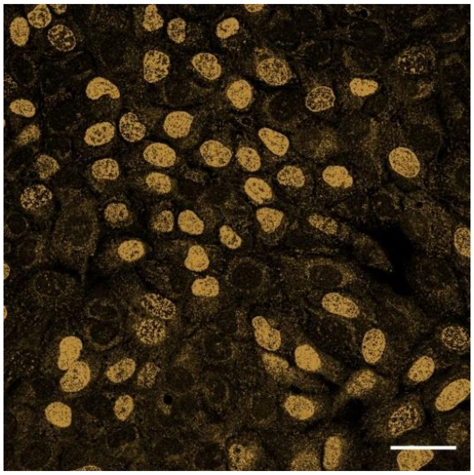

Figure S34. U2OS (fixed); **dC^{p4TCO}TP** + **T4**

Transport **dC^{p4TCO}TP /SNTT1**: 5 min / 37° C.
Incubation in medium: 60 min / 37° C;
Fixation with methanol: 30 min / 0 ° C
Labelling with **T4**: 30 min / 37° C.
Scale bar: 50 µm

dC^{pBCN}TP

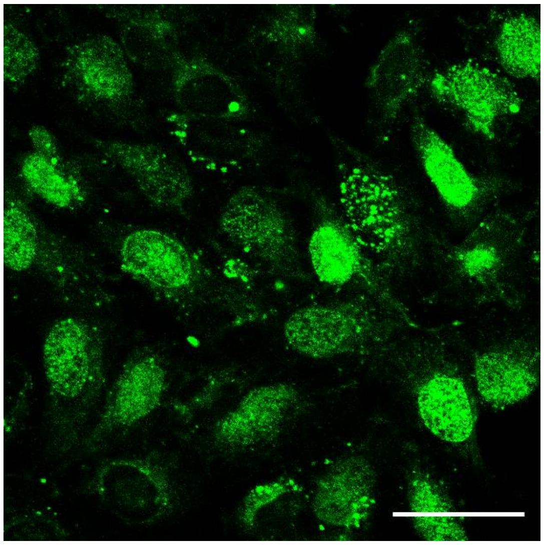

Figure S35. U2OS; **dC^{pBCN}TP** + **T1**

Transport **dC^{pBCN}TP** /**SNTT1**: 5 min / 37° C.
Incubation in medium: 60 min / 37° C;
Labelling with **T1**: 15 min / 37° C.
Scale bar: 50 µm

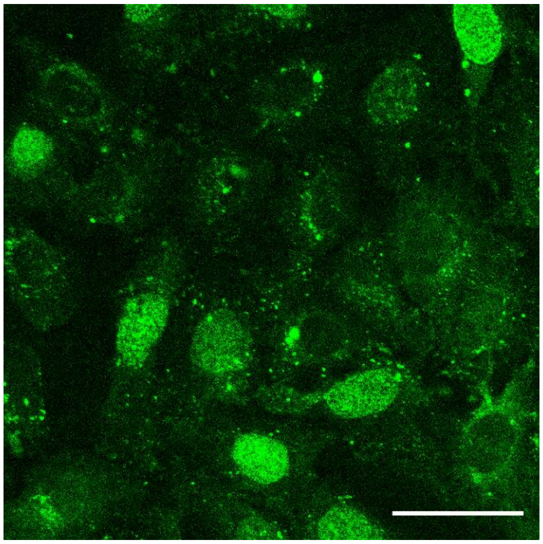

Figure S36. U2OS; **dC^{pBCN}TP** + **T2**

Transport **dC^{pBCN}TP** /**SNTT1**: 5 min / 37° C.
Incubation in medium: 60 min / 37° C;
Labelling with **T2**: 15 min / 37° C.
Scale bar: 50 µm

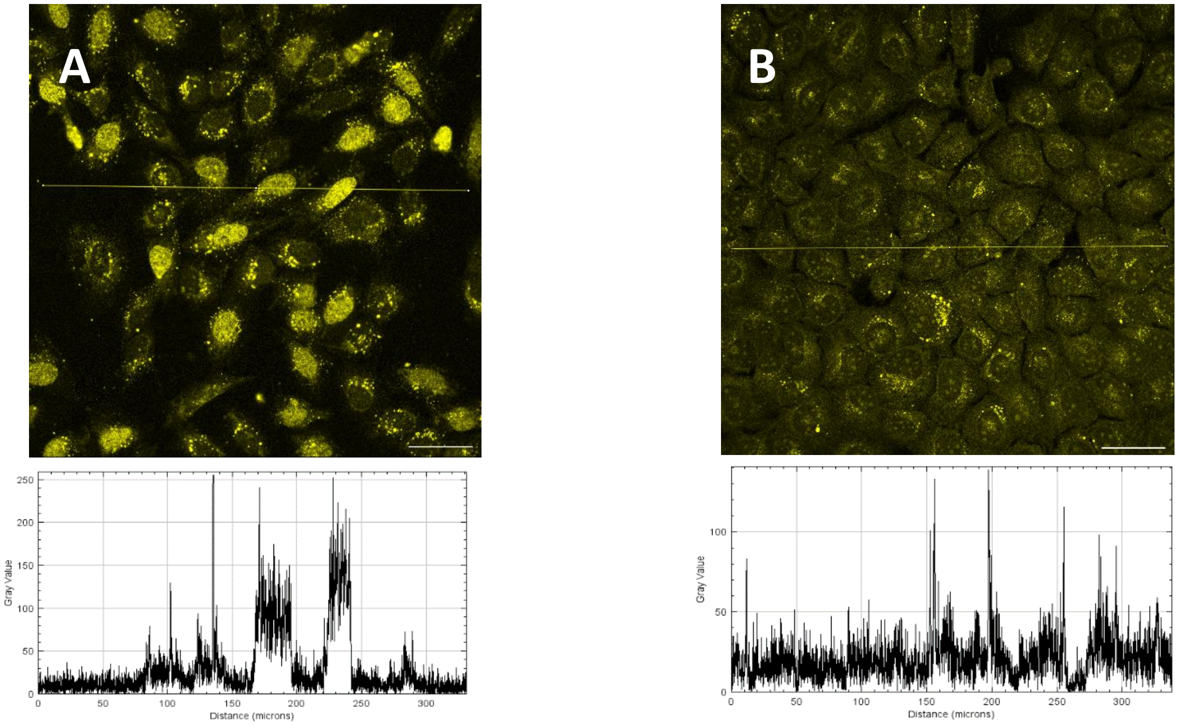

Figure S37. U2OS; **dC^{pBCN}TP** + **T3**
Images were acquired under identical settings. Graphs below images show intensities under the indicated yellow lines. The intensities were measured using raw files while the prints were adjusted (brightness and contrast) for better visual evaluation.
A) Transport **dC^{pBCN}TP /SNTT1,** 5 min / 37° C followed incubation in medium for 60 min / 37° C, then labelling with **T3**: 4 hours / 37° C; Scale bar: 50 µm
B) Control experiment 1: Treatment of U2OS cells with **T3** only, 4 hours / 37° C; Scale bar: 50 µm.

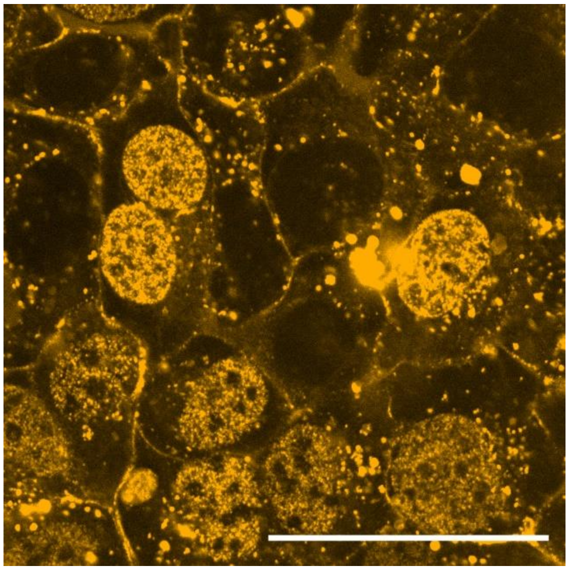

Figure S38. U2OS; **dC^{pBCN}TP** + **T4**

Transport **dC^{pBCN}TP** /**SNTT1**: 5 min / 37° C.
Incubation in medium: 60 min / 37° C;
Labelling with **T4**: 45 min / 37° C.
Scale bar: 50 µm

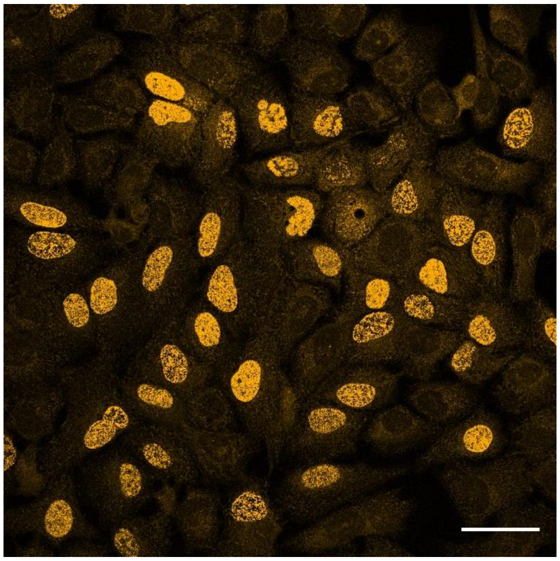

Figure S39. U2OS (fixed); **dC^{pBCN}TP** + **T4**

Transport **dC^{pBCN}TP** /**SNTT1**: 5 min / 37° C.
Incubation in medium: 60 min / 37° C;
Fixation with methanol: 30 min / 0° C
Labelling with **T4**: 45 min / 37° C.
Scale bar: 50 µm

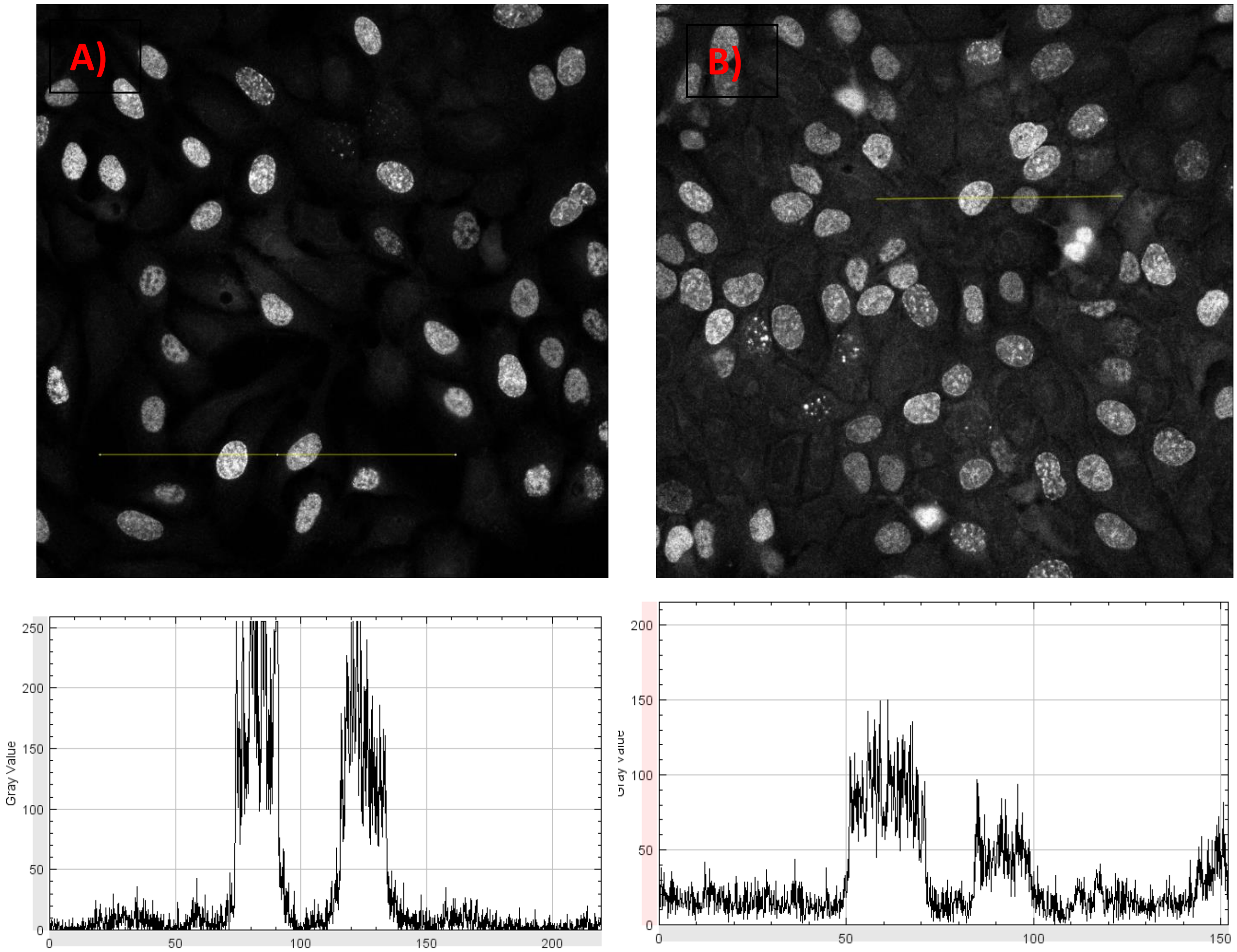

Figure S40. Comparison of contrast in images of U2OS cells labeled with A) **dC^{pBCN}TP** + **T4** and B) **dC^{p4TCO}TP** + **T4.** Images were acquired under identical conditions, acquisition parameters were adjusted according to the brightest nuclei in Image A. Image B) exhibits the lower intensity of labeled DNA foci and higher background fluorescence than image A).

Protocol:
Transport A) **dC^{pBCN}TP** /**SNTT1**: 5 min / 37° C;
B) **dC^{p4TCO}TP** /**SNTT1**: 5 min / 37° C.
Incubation in medium: 60 min / 37° C;
Fixation with methanol: 30 min / 0° C
Labelling with **T4**: 45 min / 37° C.

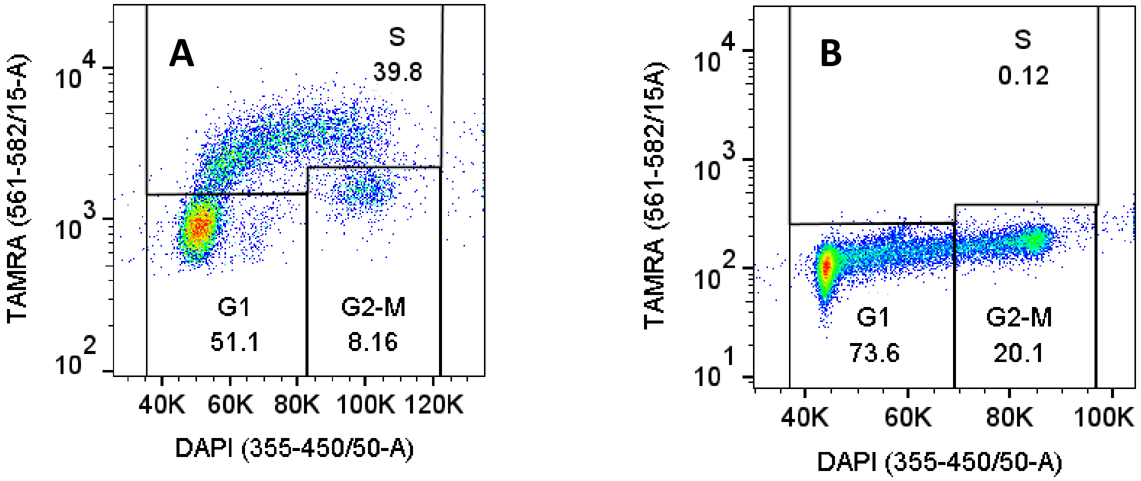

Figure S41. **A**) DNA incorporation-based cell cycle analysis of U2-OS cells. The cells were pulse-treated with a mixture of **dC^{pBCN}TP / SNTT 1** (10 µM) in tricine buffer for 4 min and further incubated in a conditioned medium for 1h. After this period, the cells were fixed with methanol, incubated with 2 µM TAMRA-tetrazine (**T4**) for 30 minutes at 37 °C, counterstained with DAPI and analyzed by flow cytometry. **B**) Control experiment: cells were fixed and treated with tetrazine **T4** only.

7. Copies of NMR Spectra

Figure S42: ^{1}H, ^{13}C, ^{31}P Spectra of compound dC^{4TCO}TP

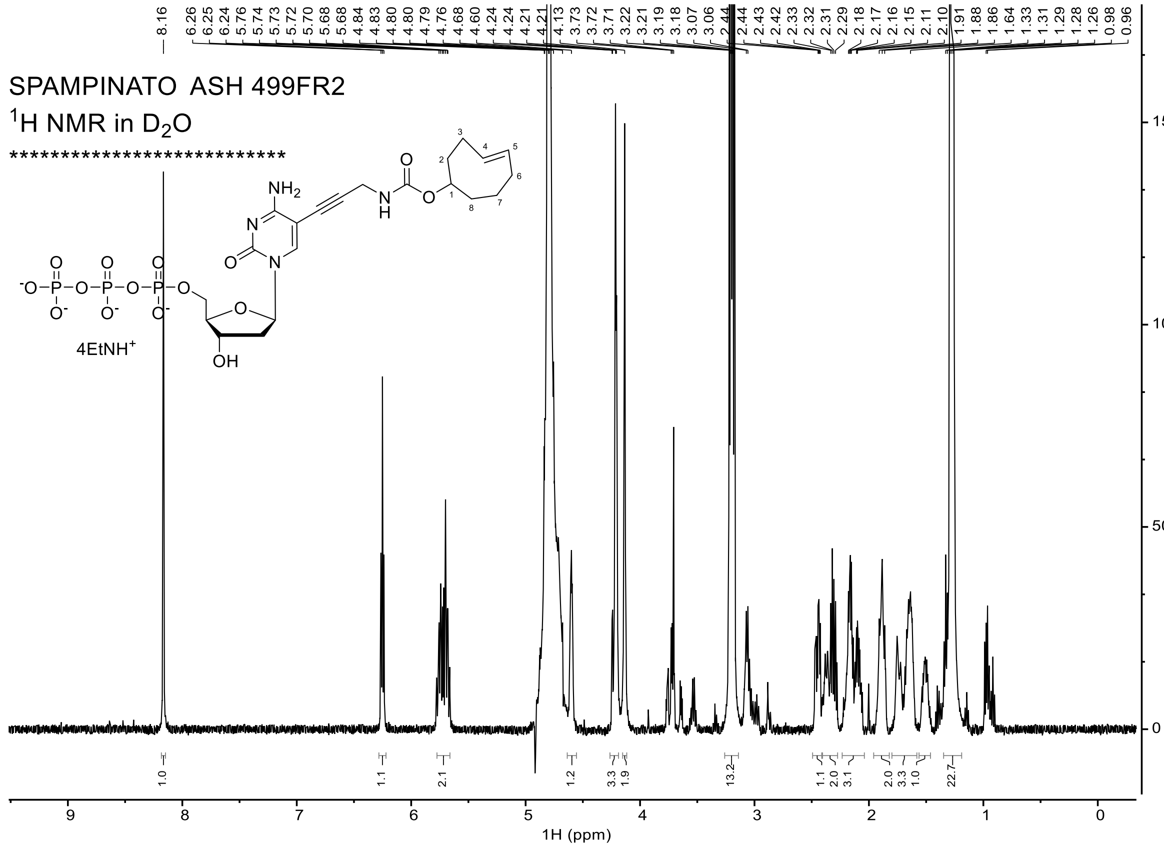

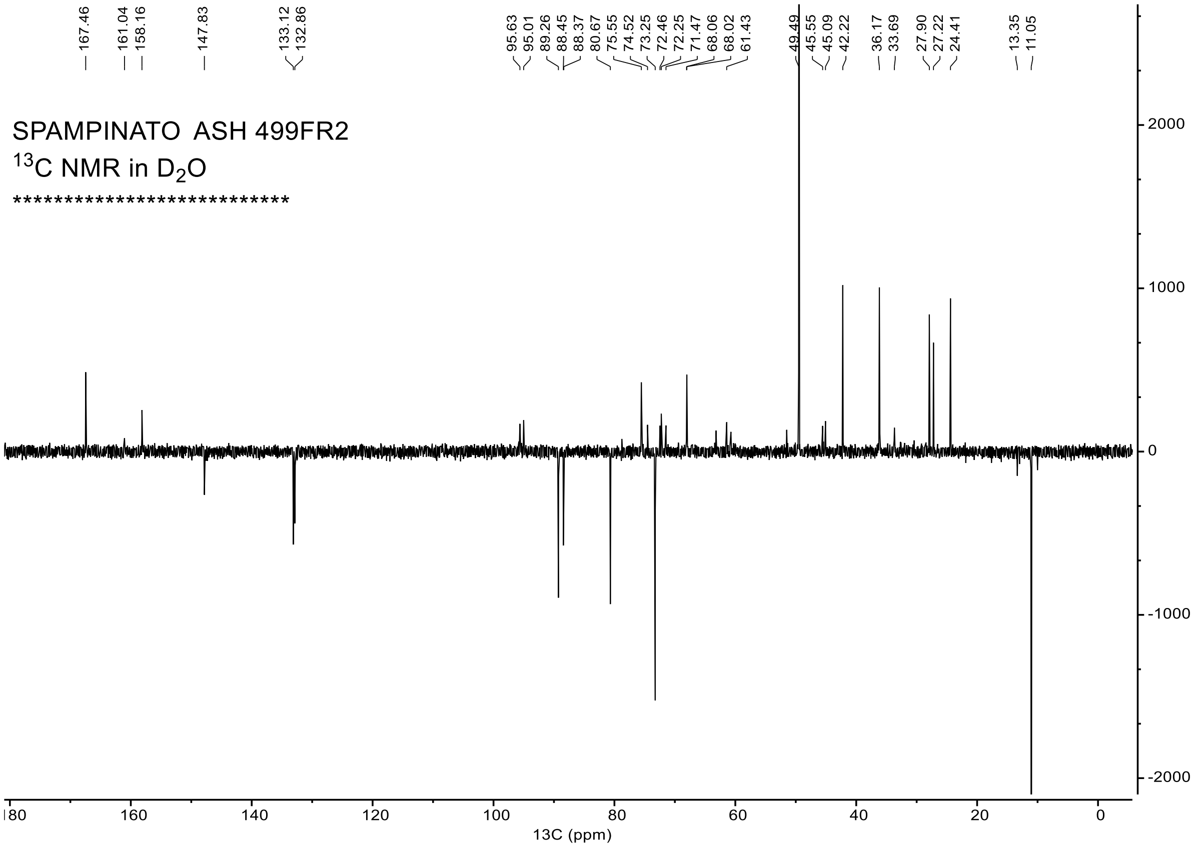
167.46
161.04
158.16
147.83
133.12
132.86
95.63
95.01
89.26
88.45
88.37
80.67
75.55
74.52
73.25
72.46
72.25
71.47
68.06
68.02
61.43
49.49
45.55
45.09
42.22
36.17
33.69
27.90
27.22
24.41
13.35
11.05
SPAMPINATO ASH 499FR2
^{13}C NMR in D_2O
****
13C (ppm)

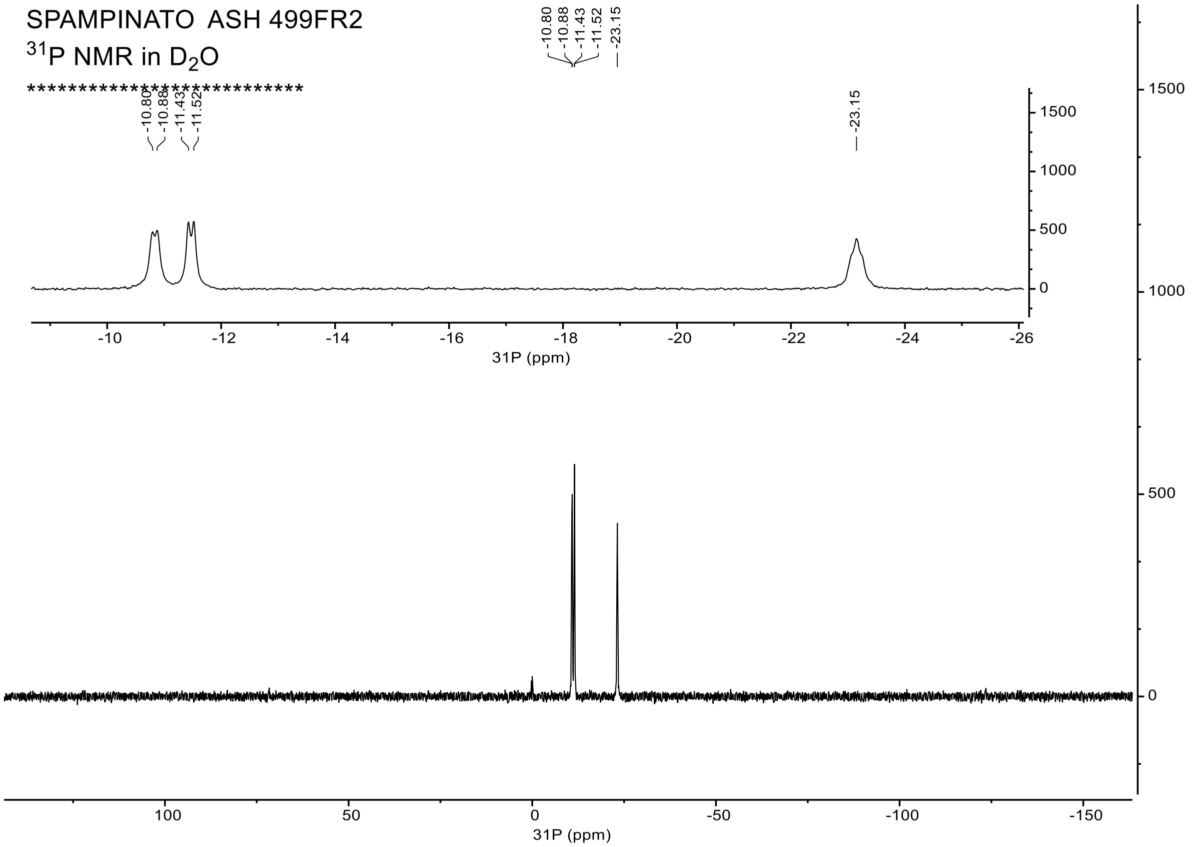
SPAMPINATO ASH 499FR2
^{31}P NMR in D_2O
****
-10.80
-10.88
-11.43
-11.52
-23.15
31P (ppm)
31P (ppm)

Figure S43: ^{1}H, ^{13}C, ^{31}P Spectra of compound $dC^{2TCO}TP$

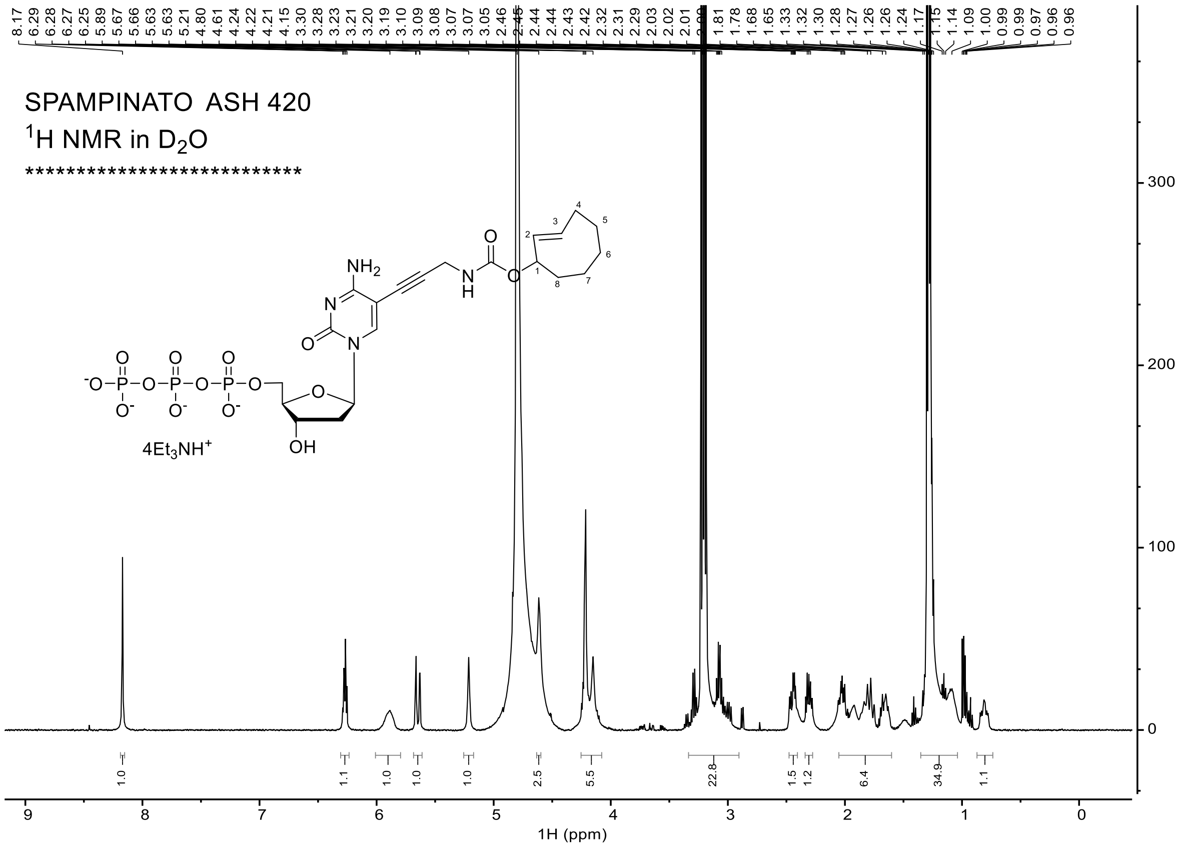

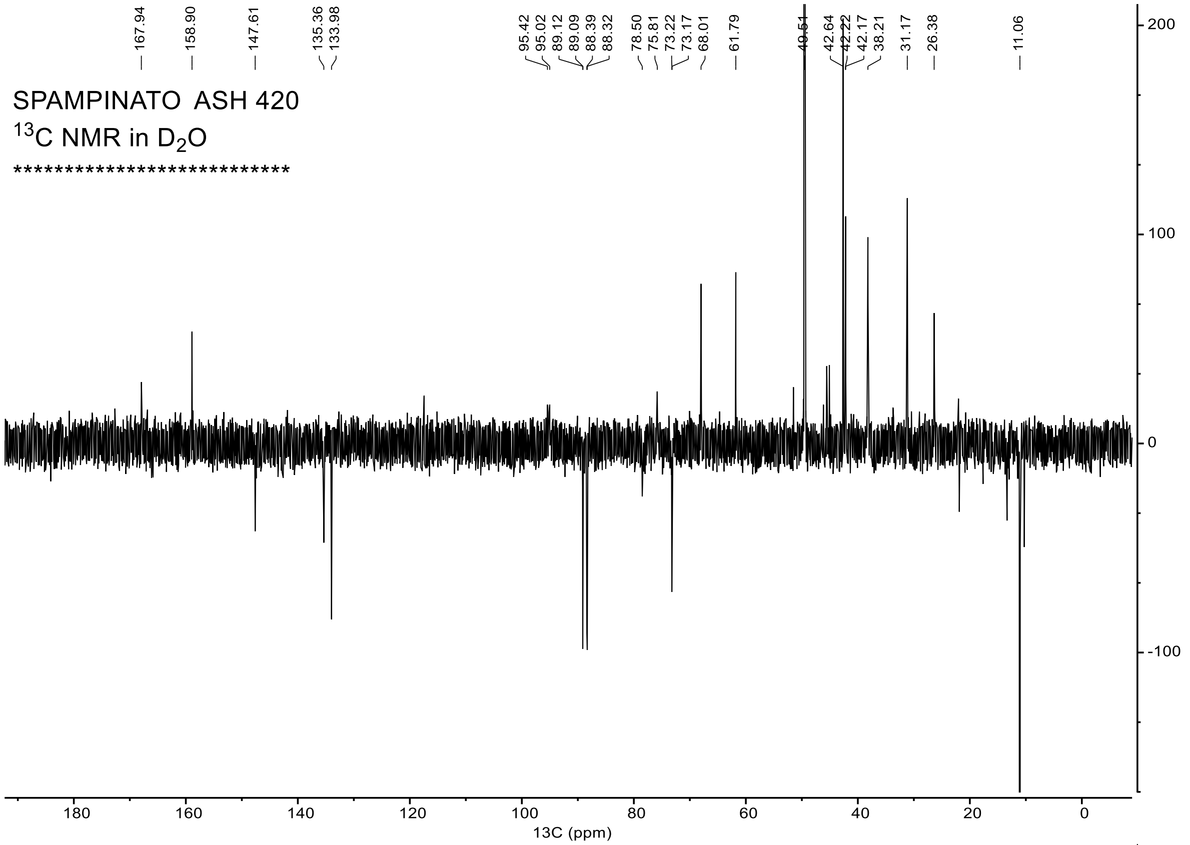
SPAMPINATO ASH 420
13C NMR in D2O
****
167.94
158.90
147.61
135.36
133.98
95.42
95.02
89.12
89.09
88.39
88.32
78.50
75.81
73.22
73.17
68.01
61.79
49.51
42.64
42.22
42.17
38.21
31.17
26.38
11.06
13C (ppm)

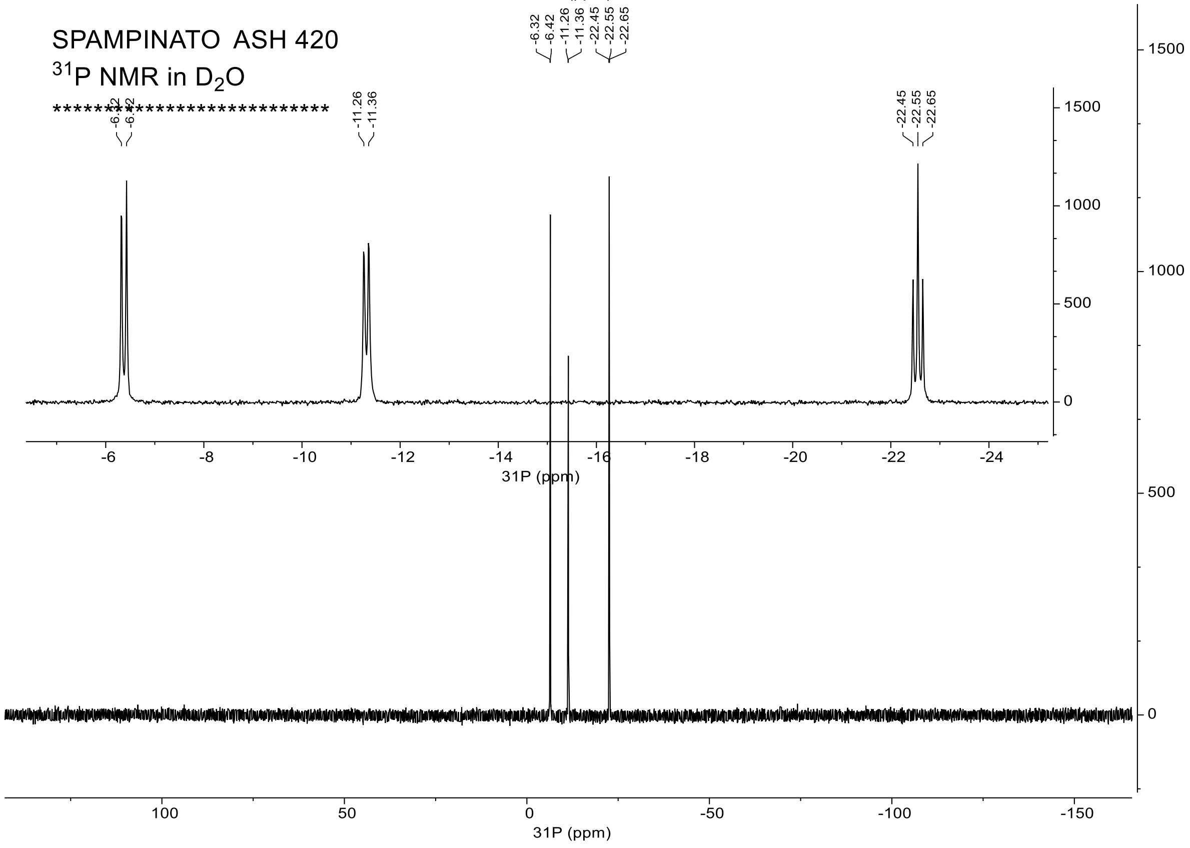
SPAMPINATO ASH 420
31P NMR in D2O
****
-6.32
-6.42
-11.26
-11.36
-22.45
-22.55
-22.65
31P (ppm)

Figure S44: ^{1}H, ^{13}C, ^{31}P Spectra of compound dC^{p4TCO}TP

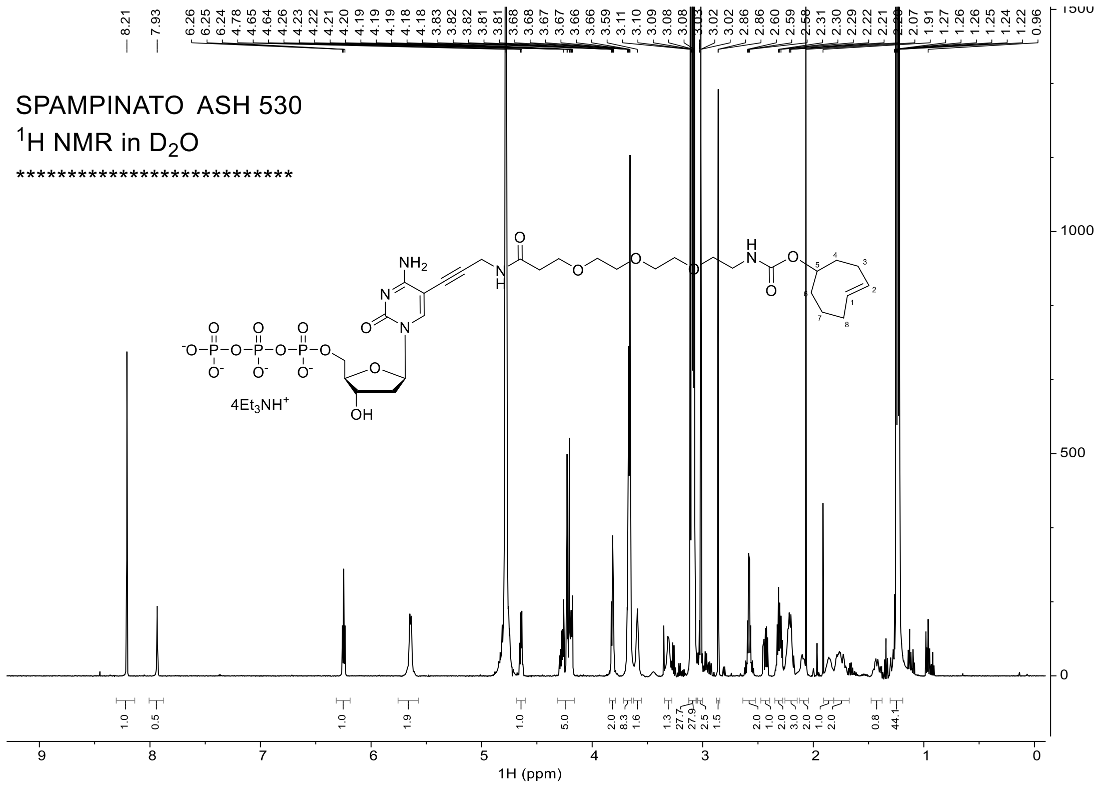

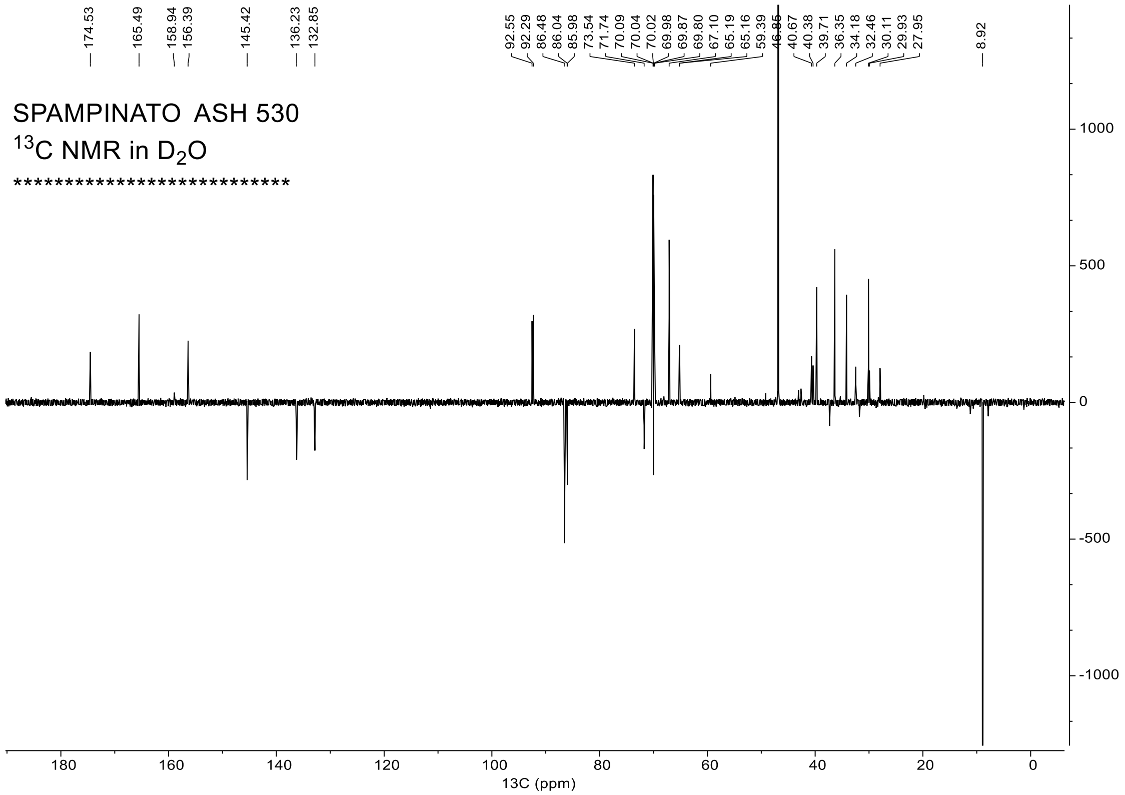

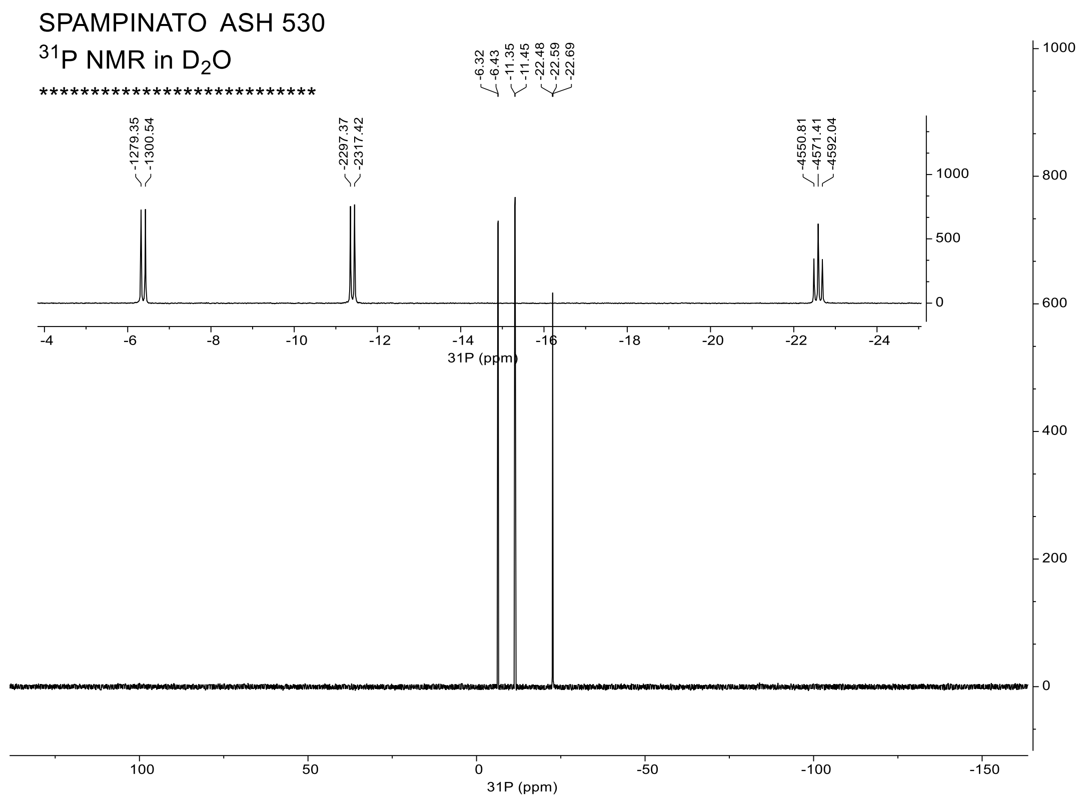

Figure S45: ^{1}H, ^{13}C, ^{31}P Spectra of compound dC^{p2TCO}TP

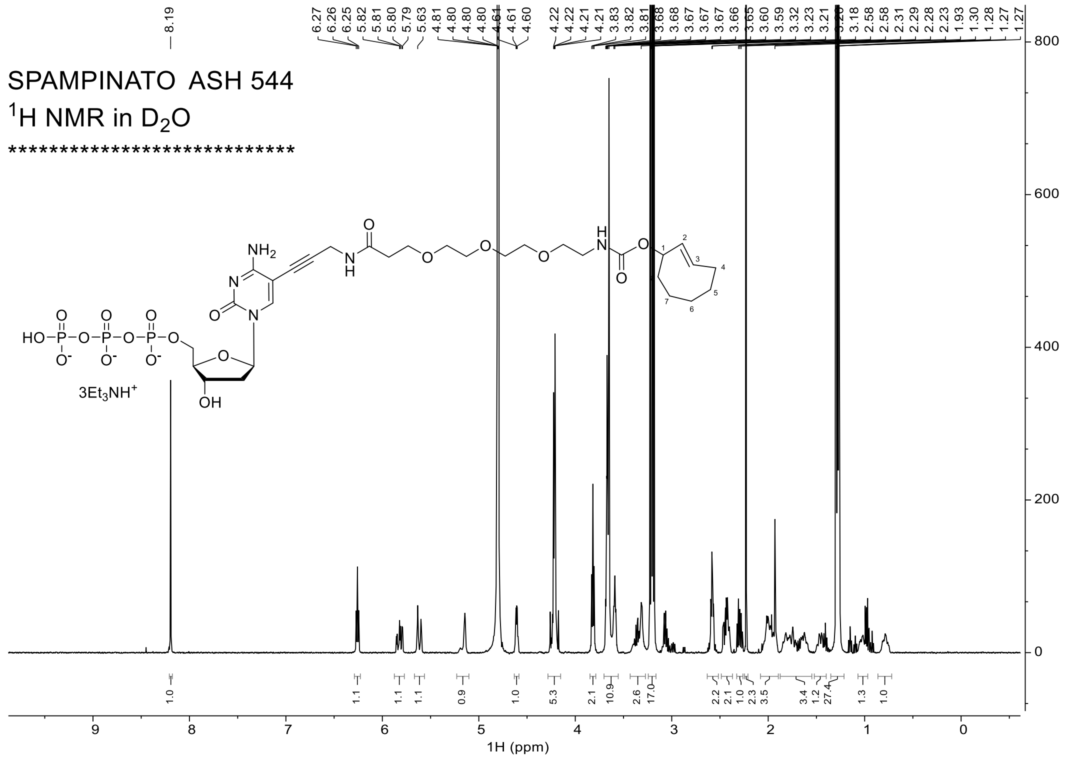

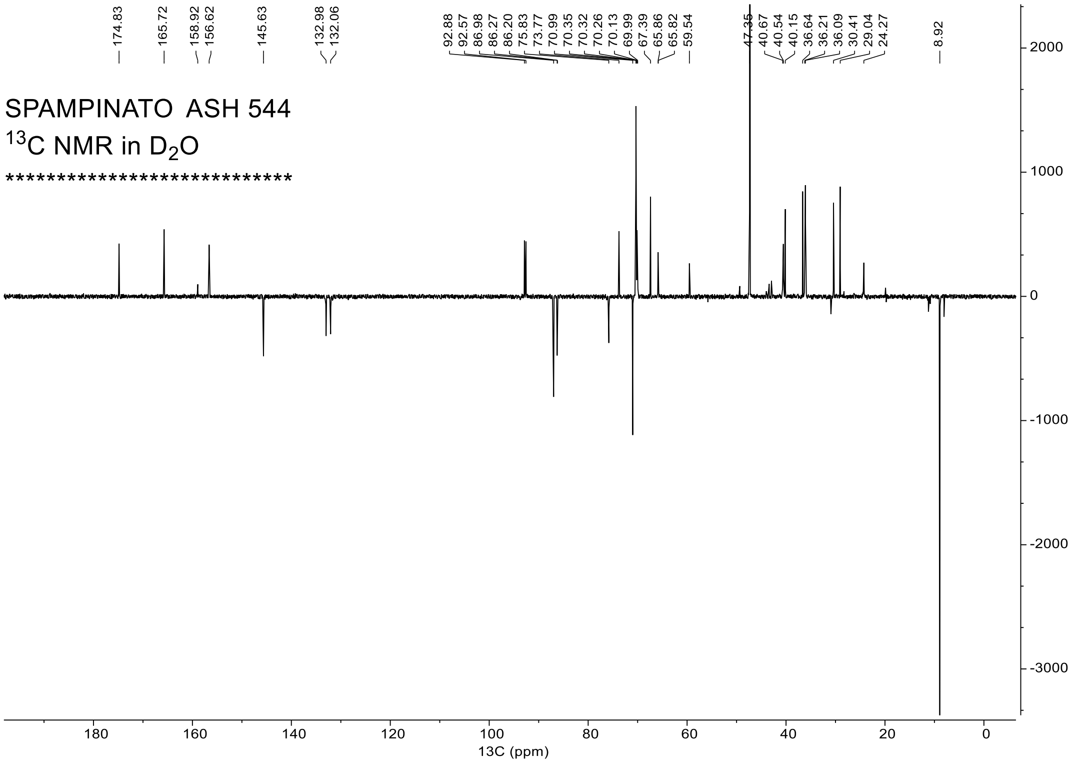
SPAMPINATO ASH 544
13C NMR in D2O
****
174.83
165.72
158.92
156.62
145.63
132.98
132.06
92.88
92.57
86.98
86.20
75.83
73.77
70.99
70.35
70.32
70.26
70.13
69.99
67.39
65.86
65.82
59.54
47.35
40.67
40.54
40.15
36.64
36.21
36.09
30.41
29.04
24.27
8.92
13C (ppm)

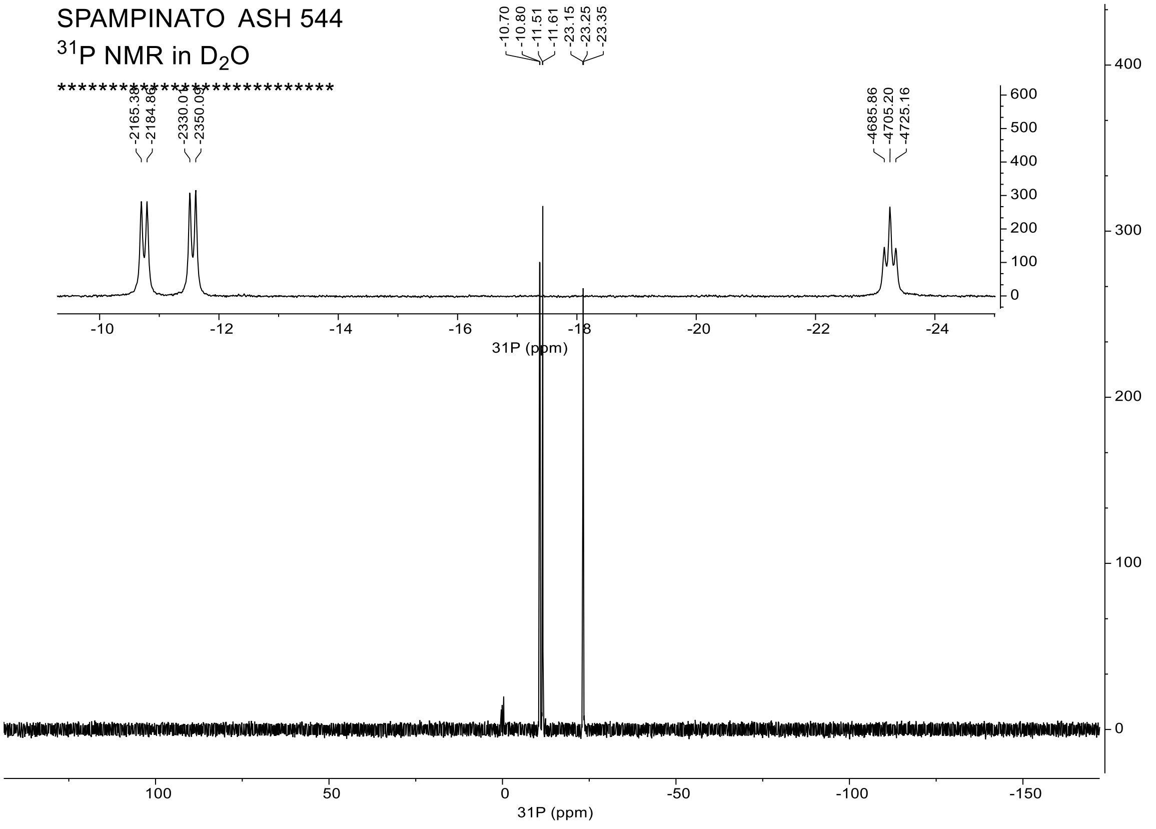
SPAMPINATO ASH 544
31P NMR in D2O
****
-10.70
-10.80
-11.51
-11.61
-23.15
-23.25
-23.35
-4685.86
-4705.20
-4725.16
31P (ppm)
31P (ppm)

Figure S46: ^{1}H, ^{13}C, ^{31}P Spectra of compound dC^{pBCN}TP

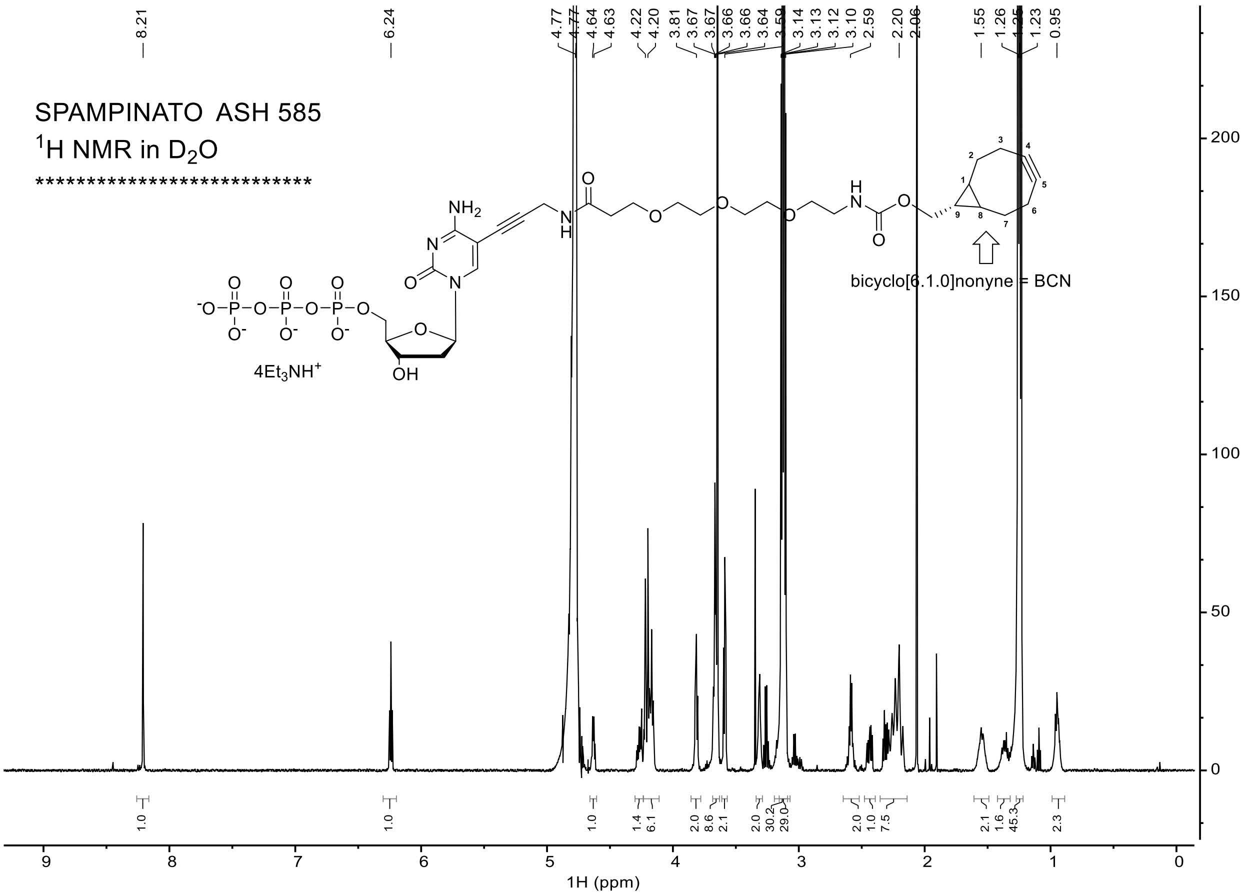

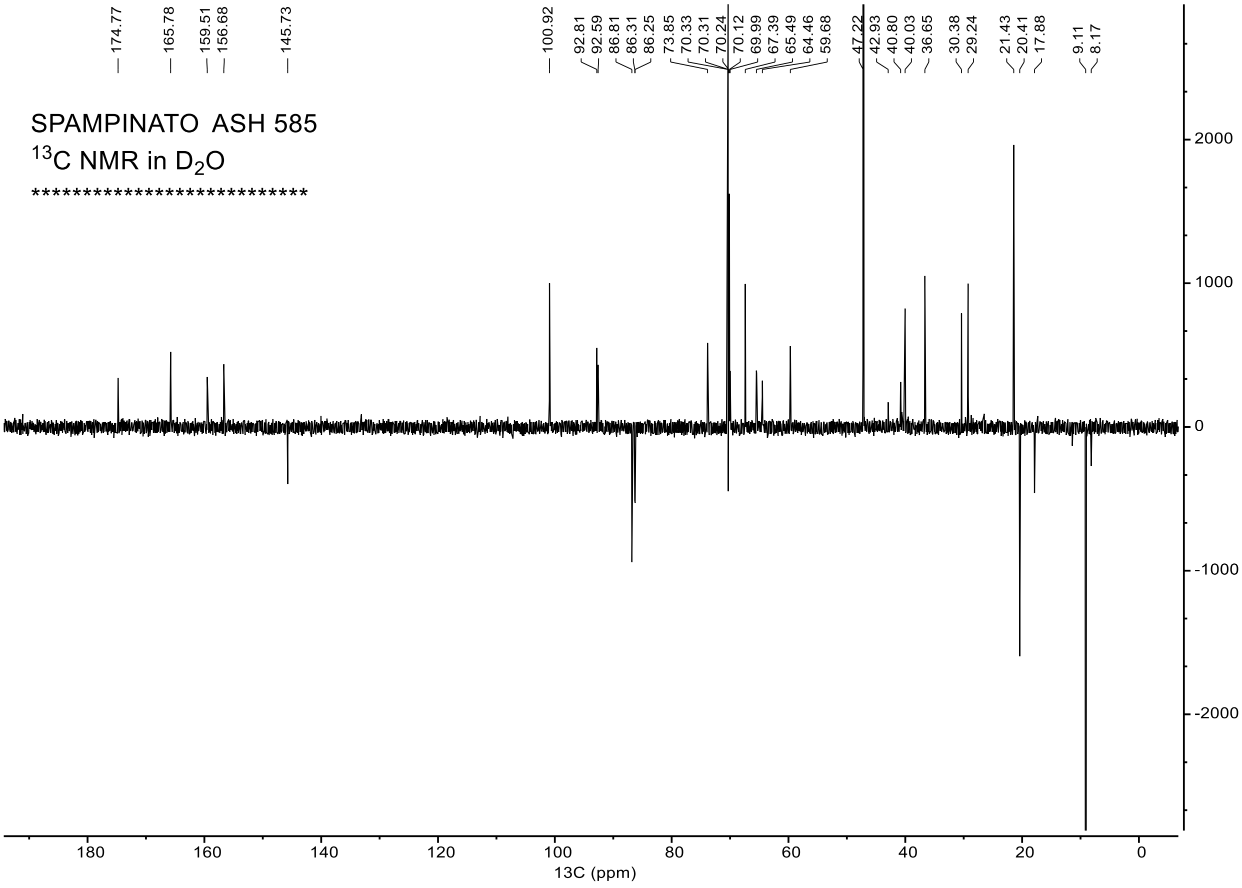
SPAMPINATO ASH 585
^{13}C NMR in D_2O
****
174.77
165.78
159.51
156.68
145.73
100.92
92.81
92.59
86.81
86.31
86.25
73.85
70.33
70.31
70.24
70.12
69.99
67.39
65.49
64.46
59.68
47.22
42.93
40.80
40.03
36.65
30.38
29.24
21.43
20.41
17.88
9.11
8.17
2000
1000
0
-1000
-2000
180
160
140
120
100
80
60
40
20
0
13C (ppm)

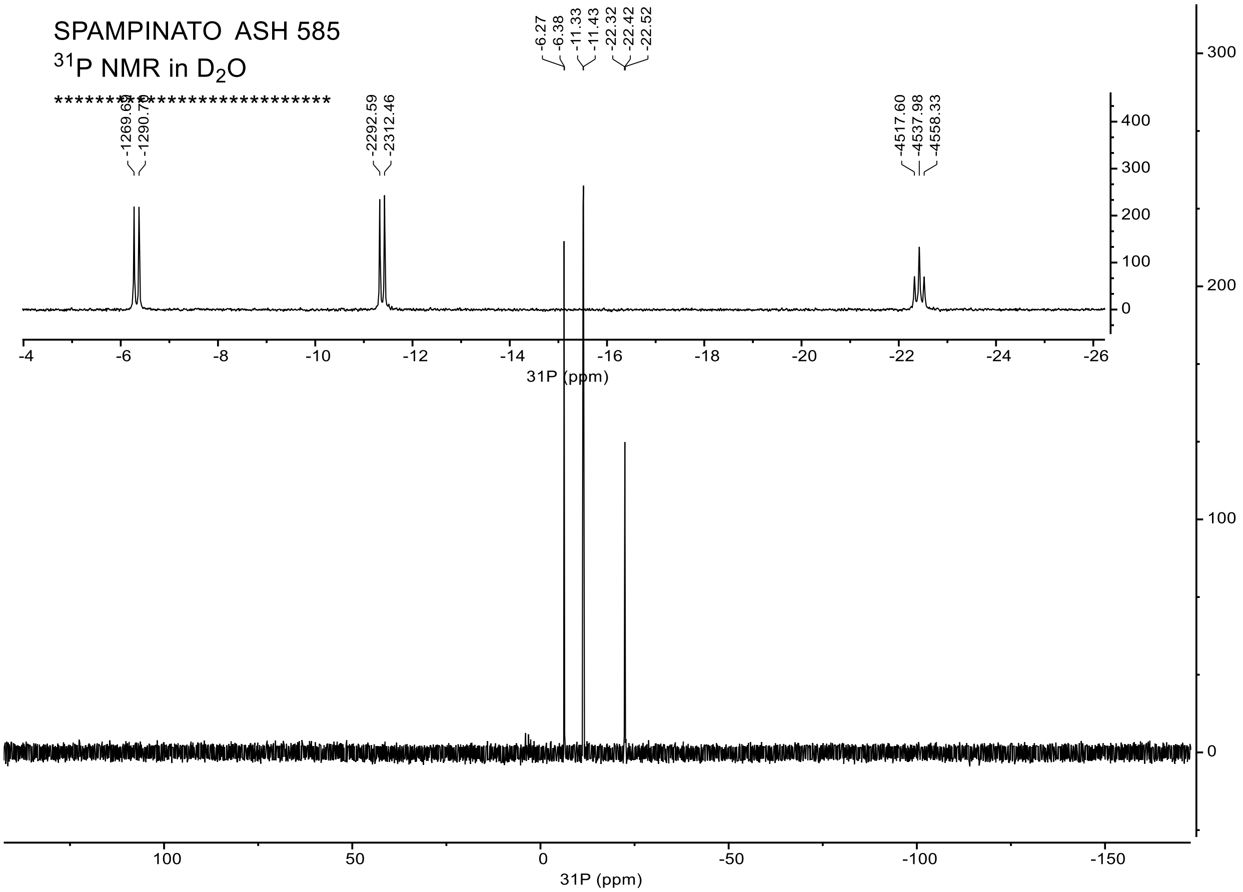
SPAMPINATO ASH 585
31P NMR in D2O
-6.27
-6.38
-11.33
-11.43
-22.32
-22.42
-22.52
-1269.62
-1290.70
-2292.59
-2312.46
-4517.60
-4537.98
-4558.33
31P (ppm)
31P (ppm)

Figure S47. : ^{1}H, ^{13}C, ^{31}P Spectra of compound **dC^{NH2}MP**

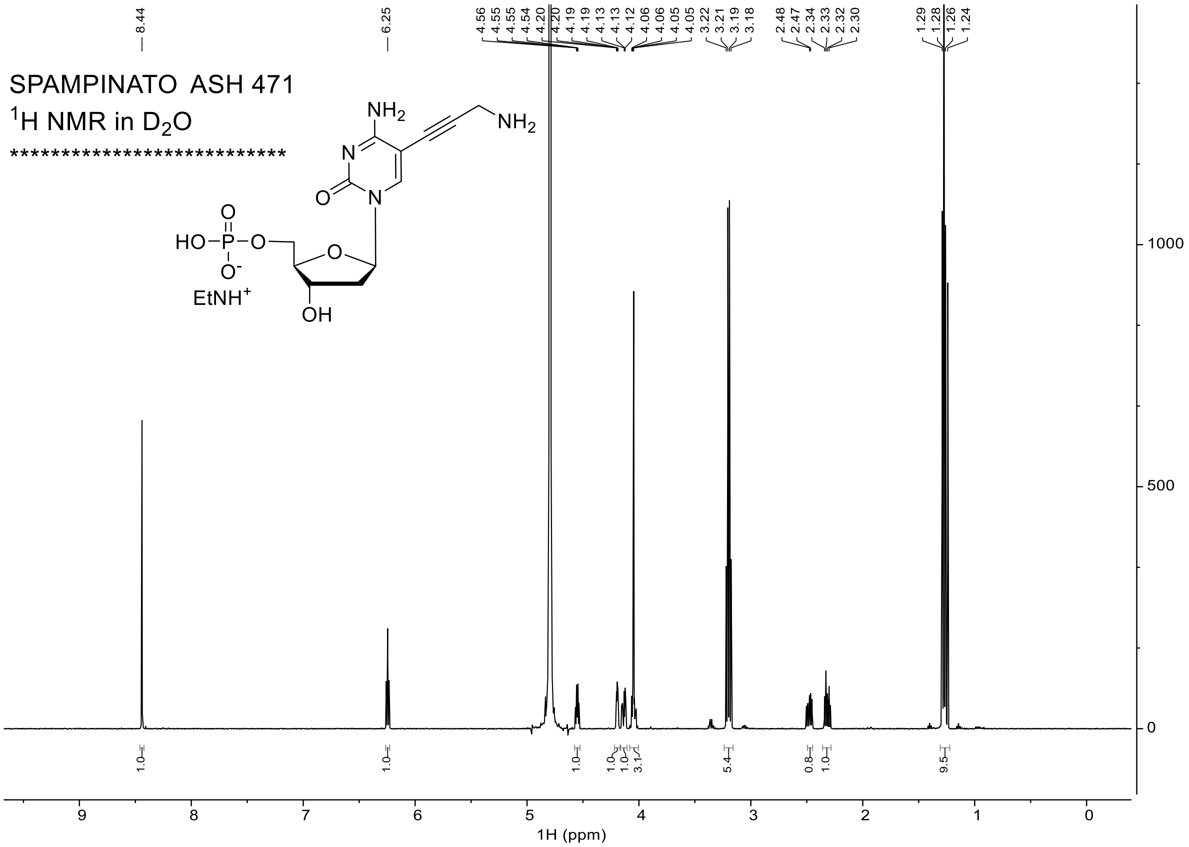

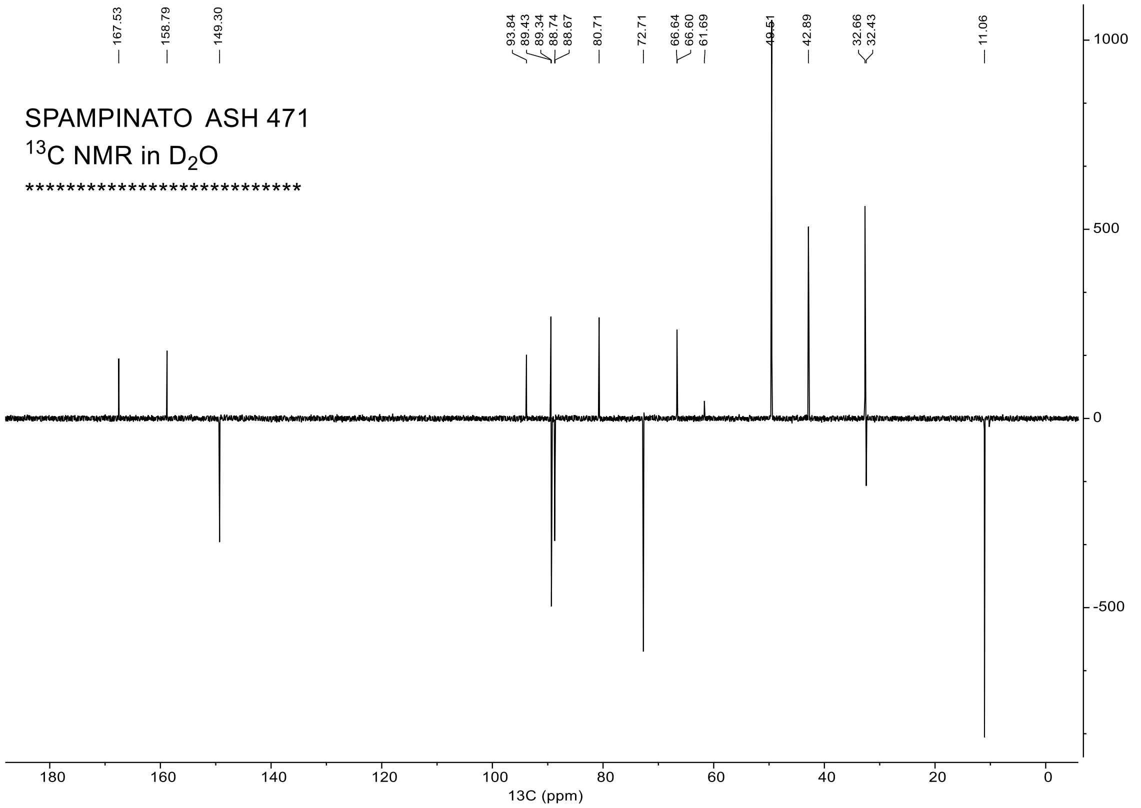
SPAMPINATO ASH 471
13C NMR in D2O
****
167.53
158.79
149.30
93.84
89.43
89.34
88.74
88.67
80.71
72.71
66.64
66.60
61.69
49.51
42.89
32.66
32.43
11.06
1000
500
0
-500
180
160
140
120
100
80
60
40
20
0
13C (ppm)

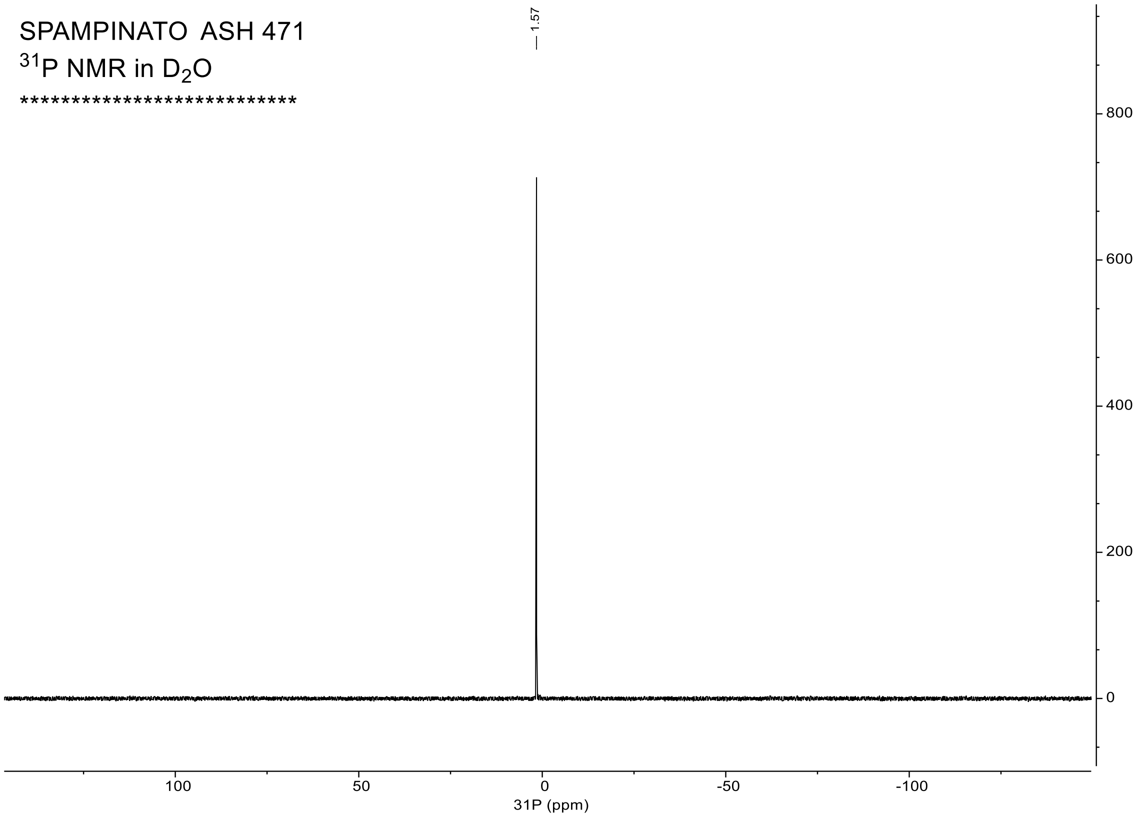

Figure S48: ^{1}H, ^{13}C, ^{31}P Spectra of compound **dC^{PAF3}MP**

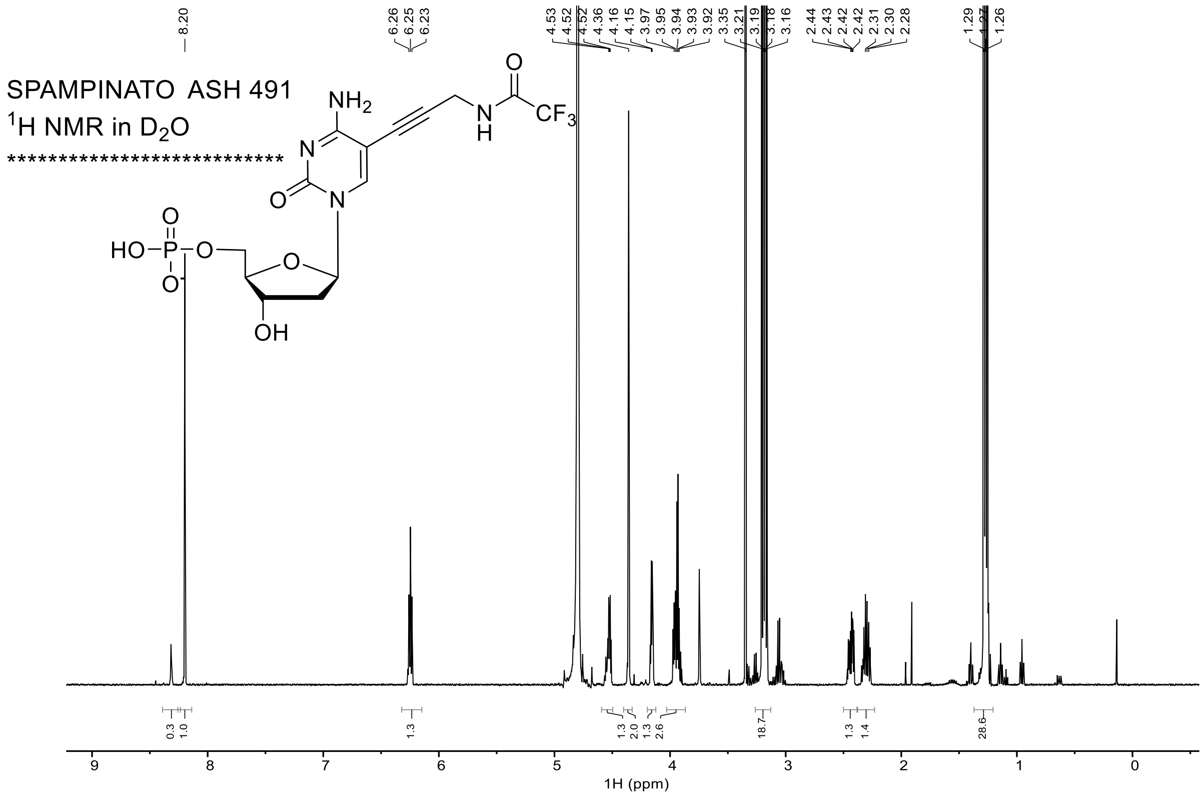

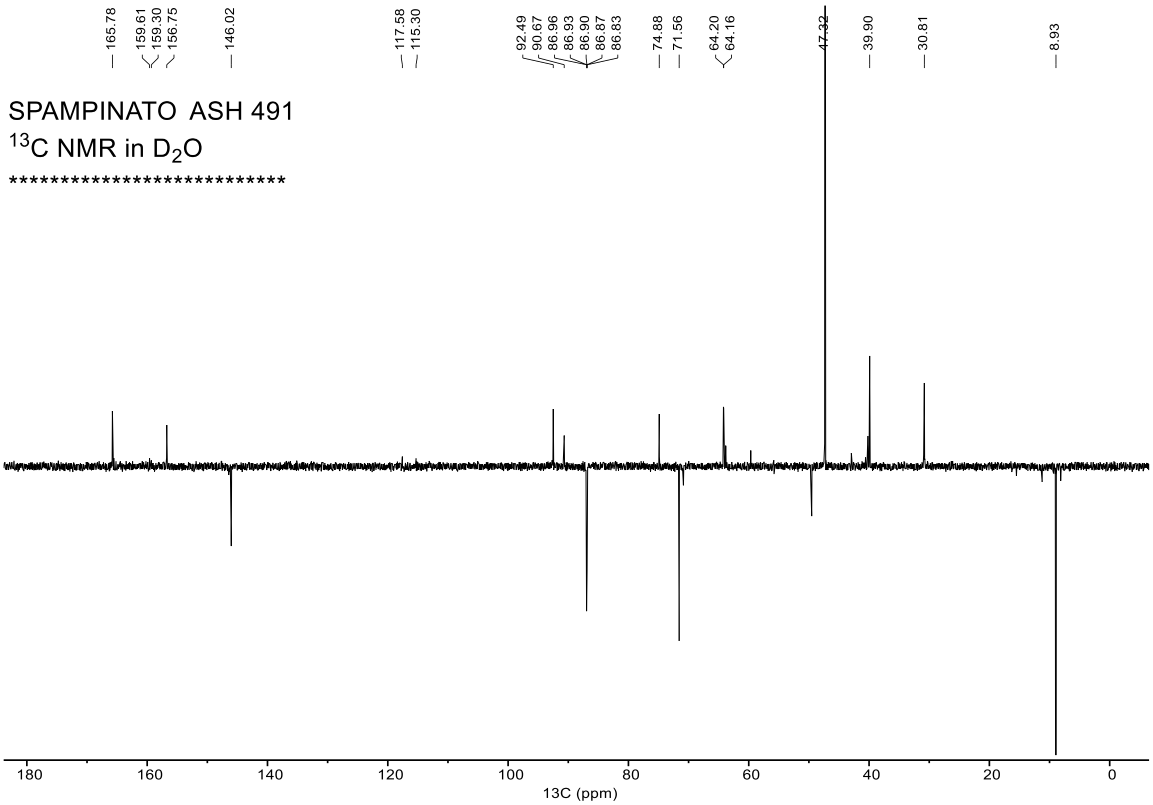
165.78
159.61
159.30
156.75
146.02
117.58
115.30
92.49
90.67
86.96
86.93
86.90
86.87
86.83
74.88
71.56
64.20
64.16
47.32
39.90
30.81
8.93
SPAMPINATO ASH 491
^{13}C NMR in D_2O
****
180
160
140
120
100
80
60
40
20
0
13C (ppm)

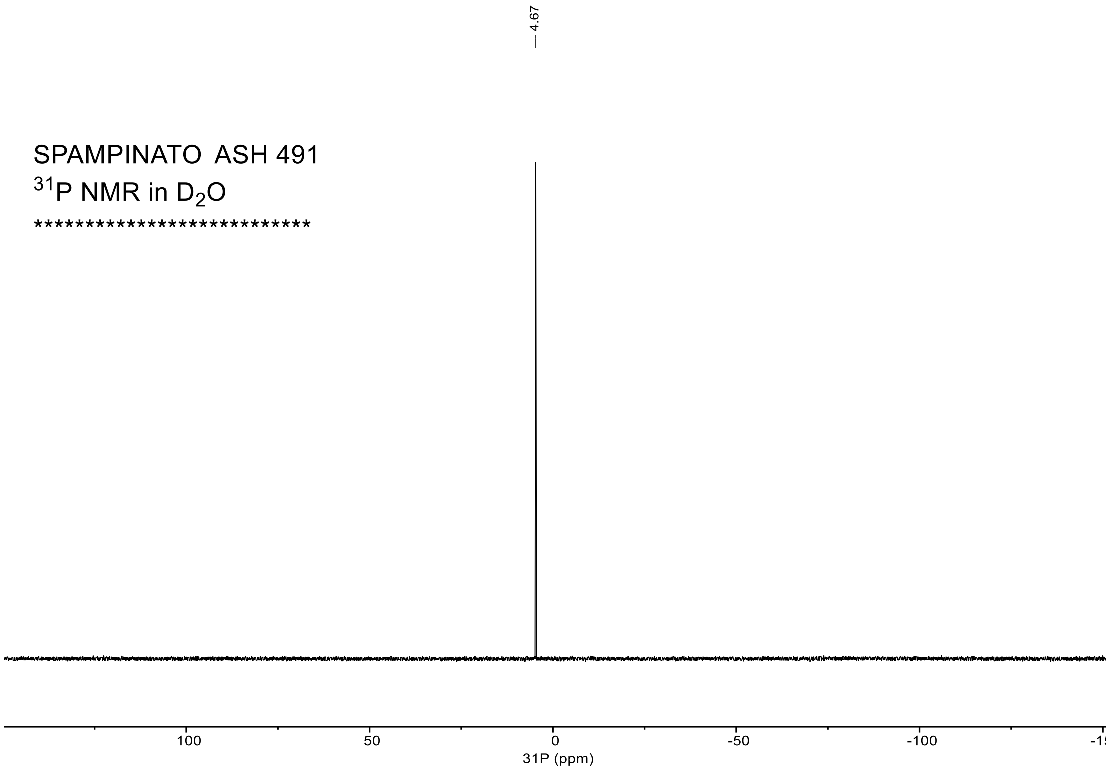
4.67
SPAMPINATO ASH 491
^{31}P NMR in D_2O
****
100
50
0
-50
-100
31P (ppm)

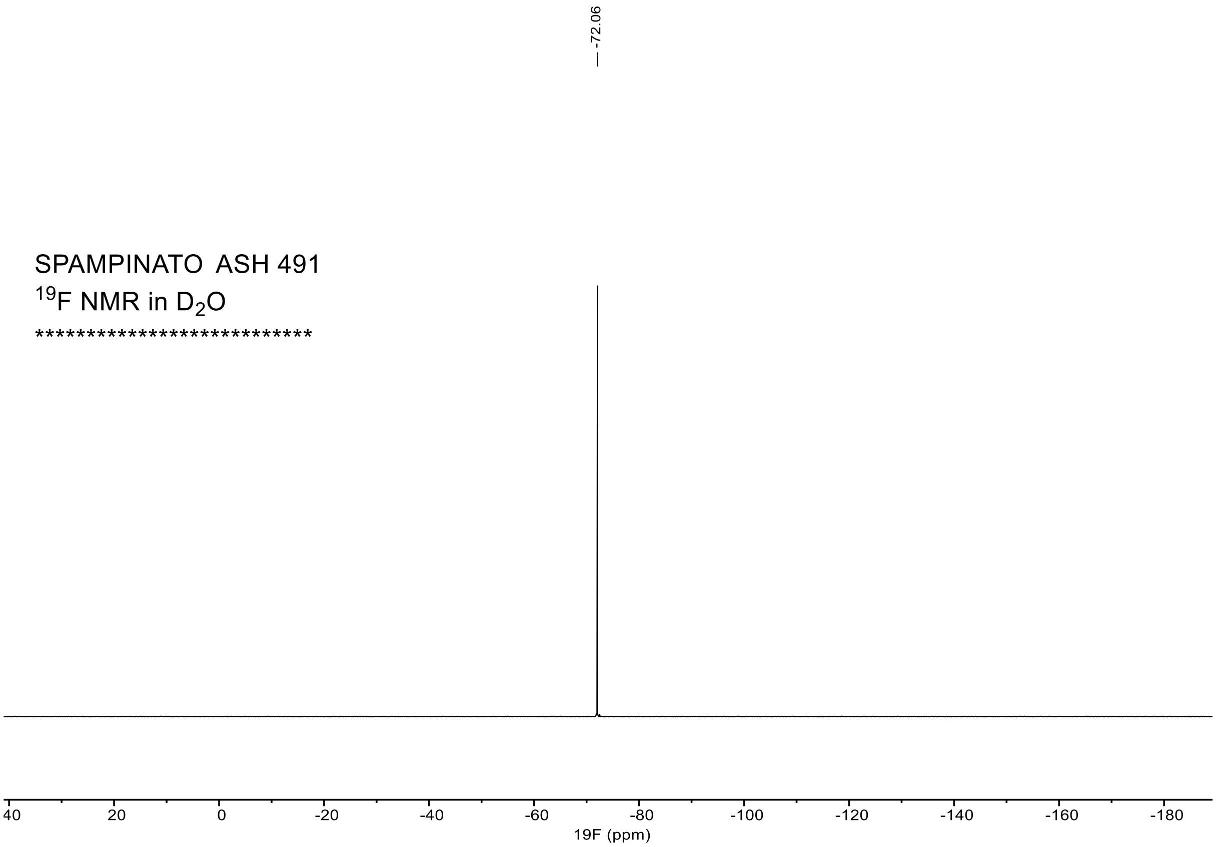
-72.06
SPAMPINATO ASH 491
19F NMR in D2O
****
40
20
0
-20
-40
-60
-80
-100
-120
-140
-160
-180
19F (ppm)

Figure S49: ^{1}H, ^{13}C, ^{31}P Spectra of compound **dC^{4TCO}MP**

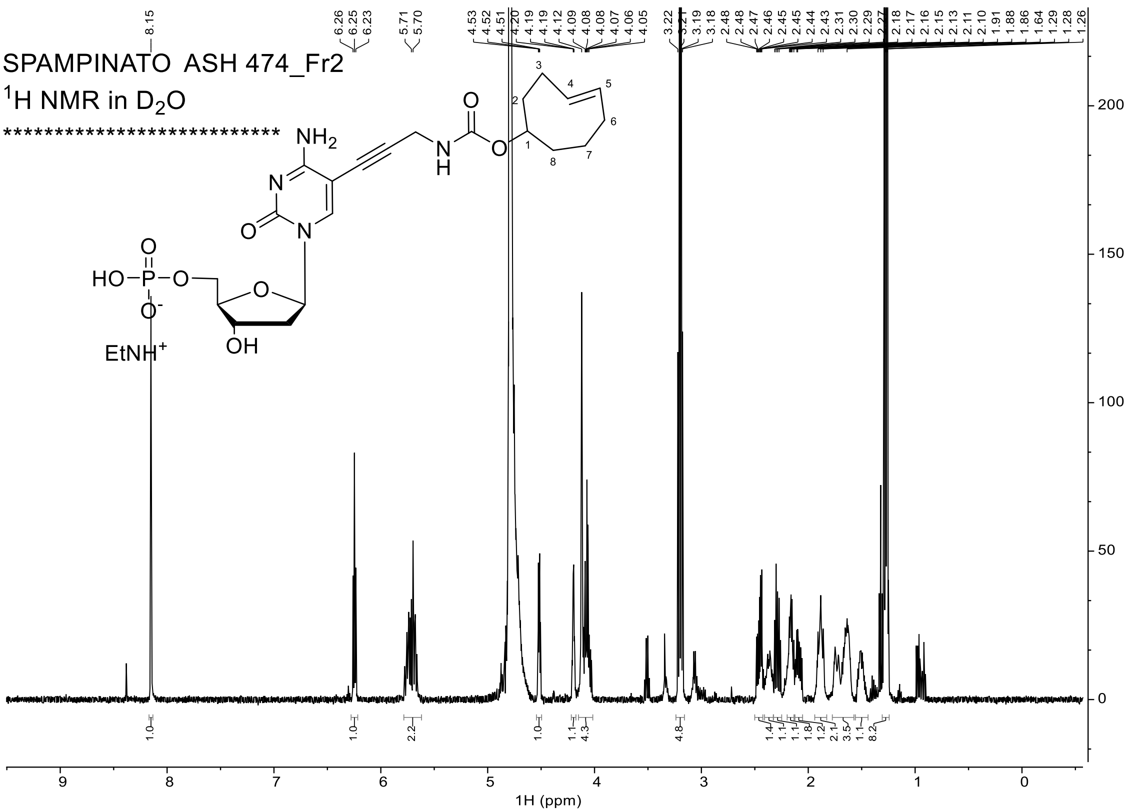

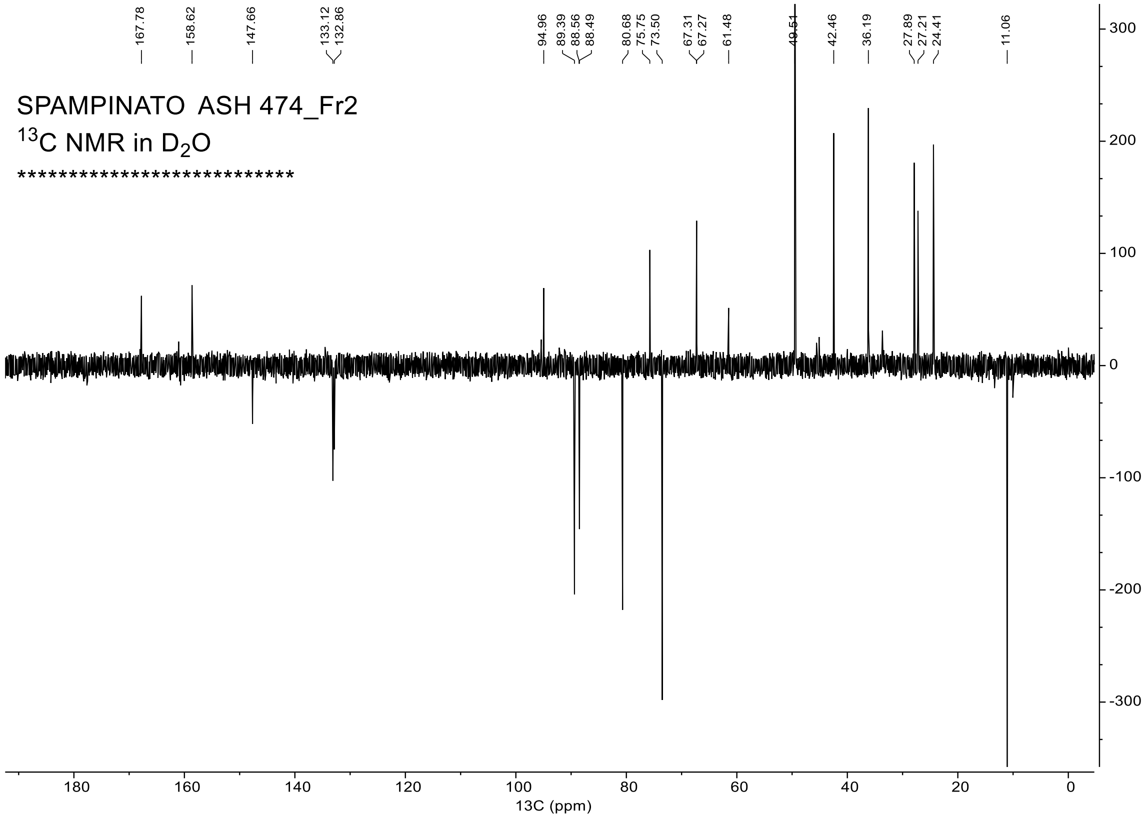

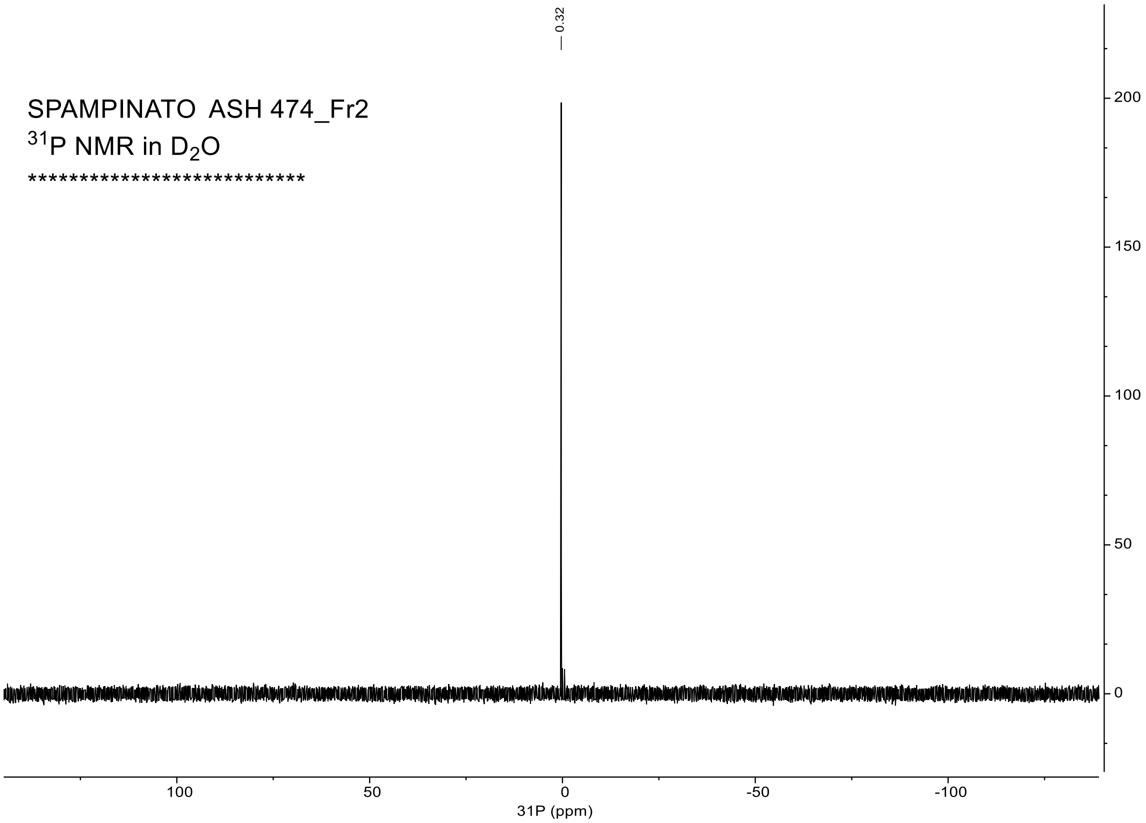
0.32
SPAMPINATO ASH 474_Fr2
31P NMR in D2O
****
200
150
100
50
0
100
50
0
-50
-100
31P (ppm)

8. Copies of MALDI-TOF mass spectra

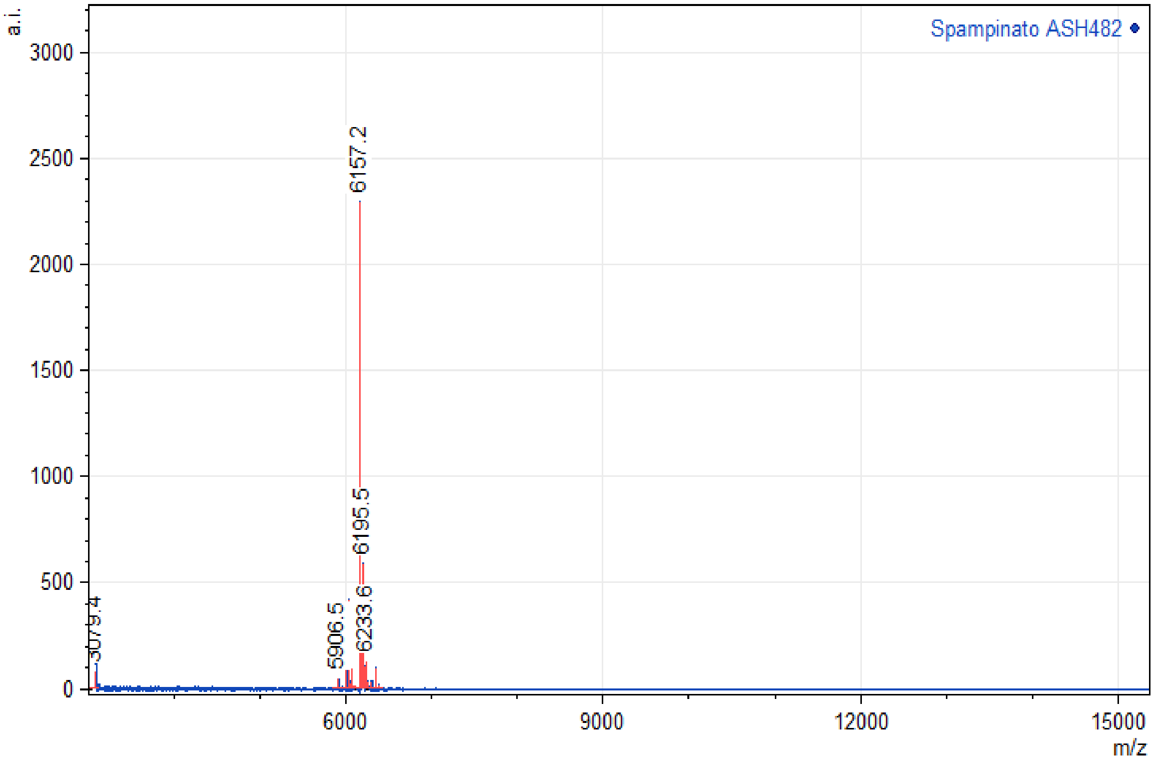

Figure S50. MALDI-TOF MS spectrum of **19ON_C^{4TCO}**: calculated [M] 6156.03 Da; found 6157.2 Da; Δ=1.06 Da.

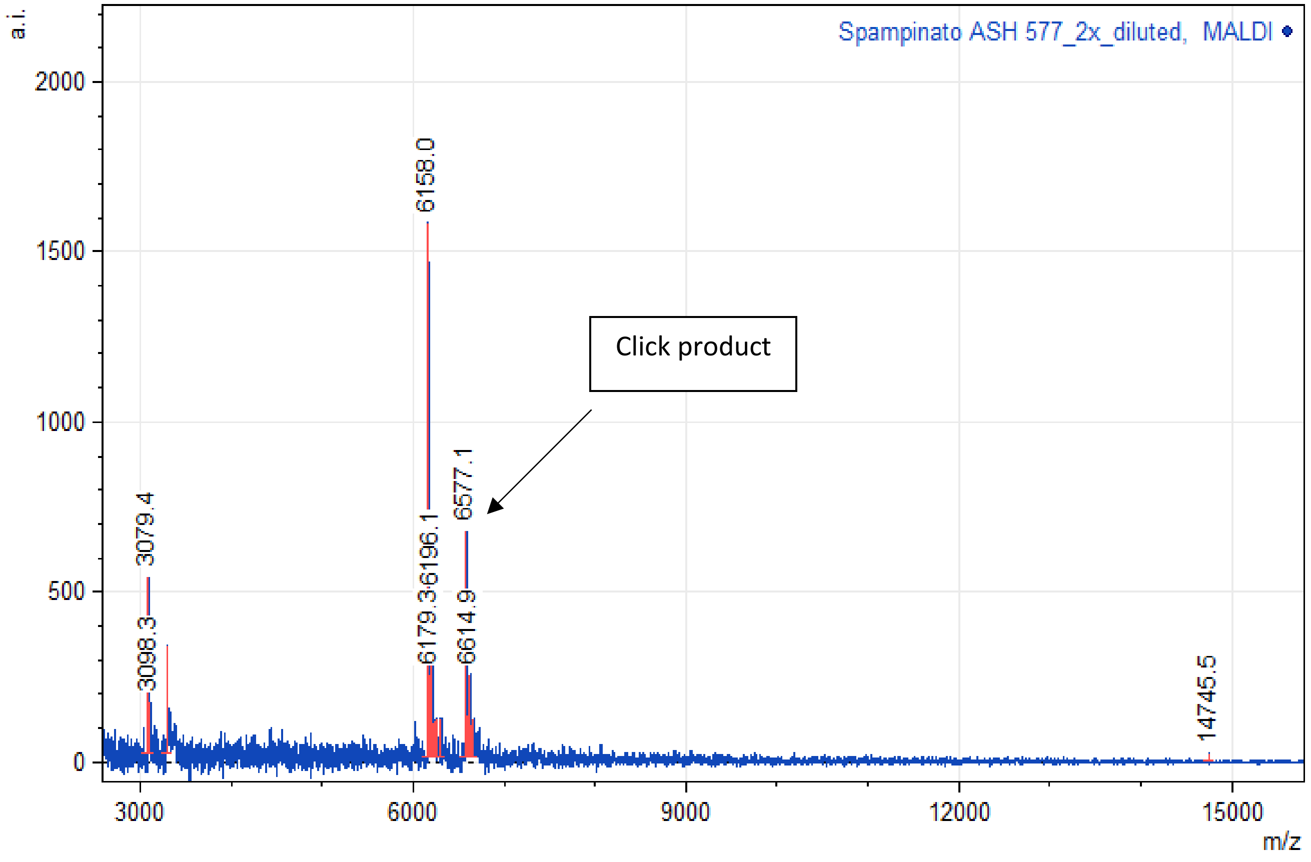

Figure S51. MALDI-TOF MS spectrum of **19ON_C^{4TCOT2}**: calculated [M] 6576.18 Da; found 6577.1 Da; Δ=1.0 Da. The peak at m/z=6158.0 Da is assigned to **19ON_C^{4TCO}**.

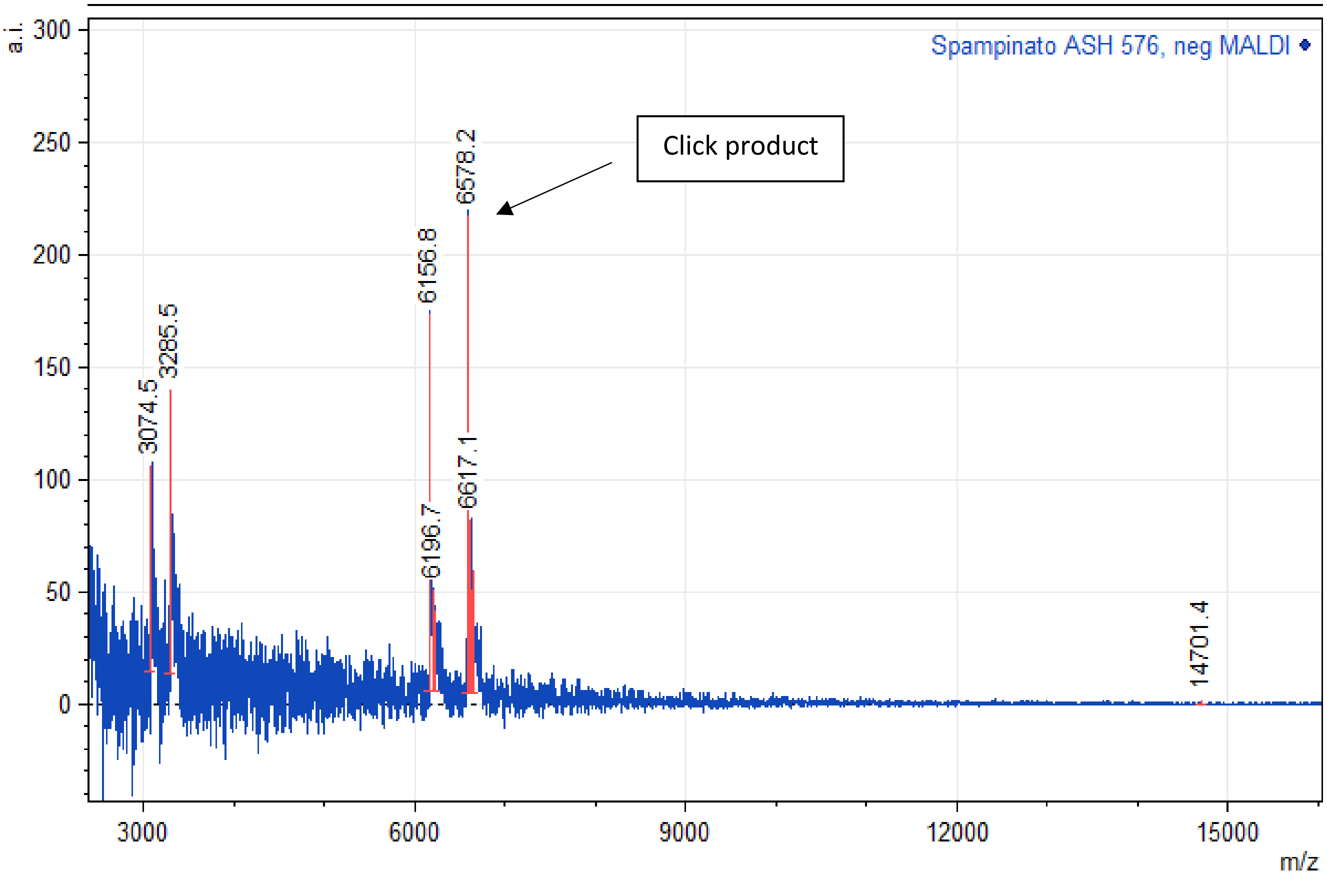

Figure S52. MALDI-TOF MS spectrum of **19ON_C^{4TCOT1}**: calculated [M] 6578.18 Da; found 6578.2 Da; Δ=0.02 Da. The peak at m/z=6156.8 Da is assigned to **19ON_C^{4TCO}**.

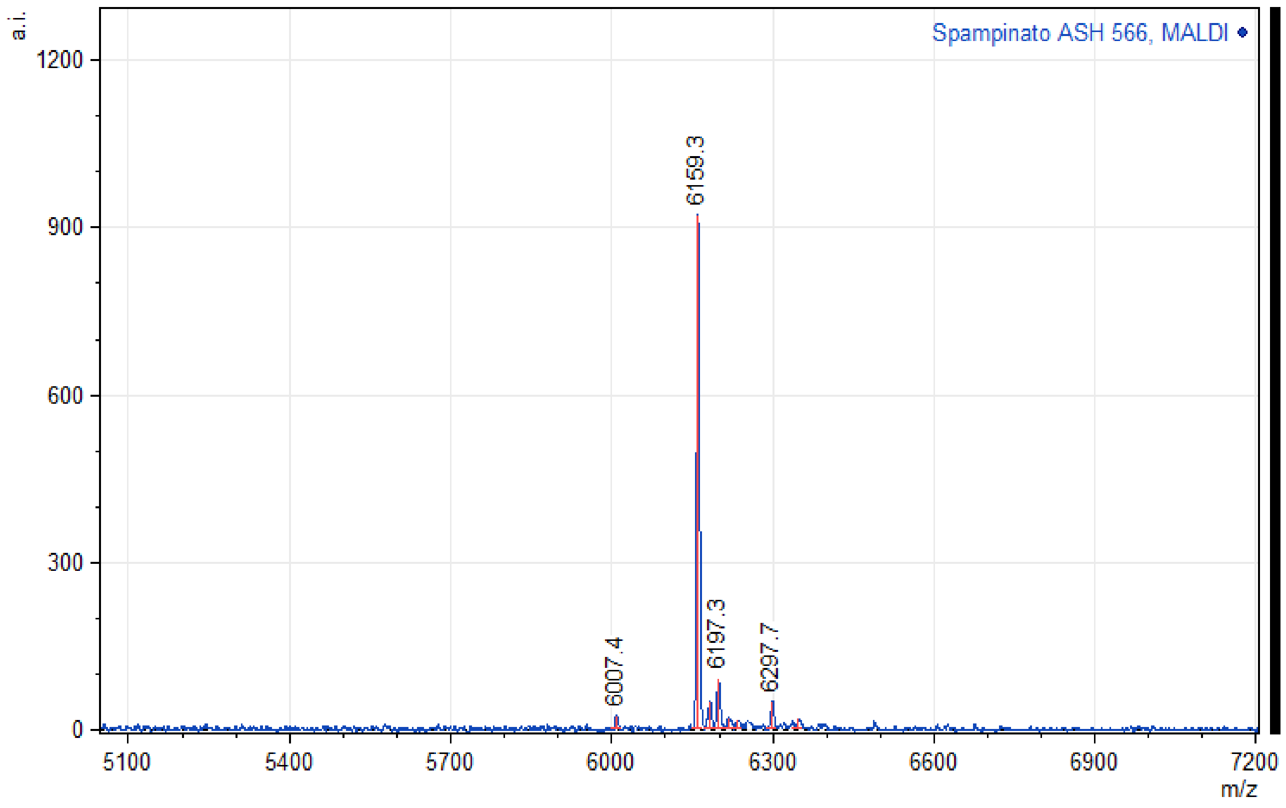

Figure S53. MALDI-TOF MS spectrum of **19ON_C^{2TCO}**: calculated [M] 6156.03 Da; found 6159.3 Da; Δ=3.3 Da.

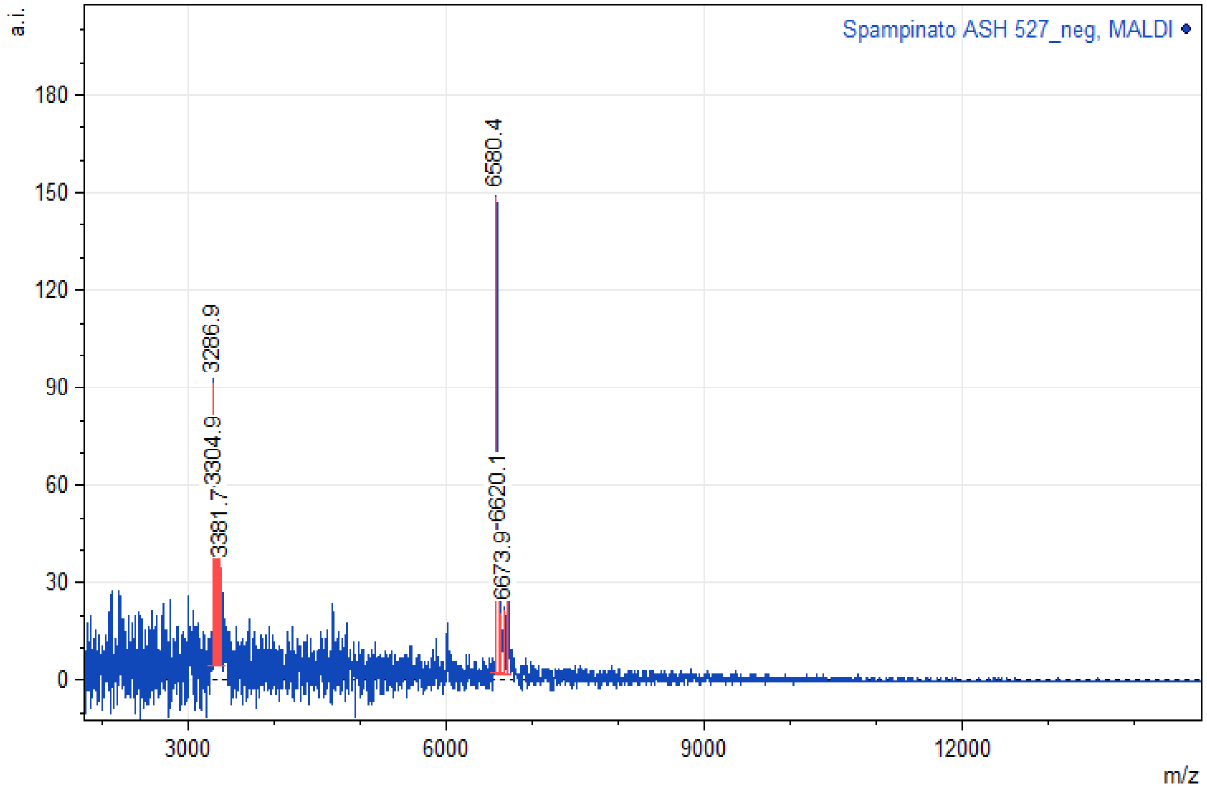

Figure S54. MALDI-TOF MS spectrum of **19ON_C^{2TCOT1}**: calculated [M] 6578.1 Da; found 6580.4 Da; Δ=2.3 Da.

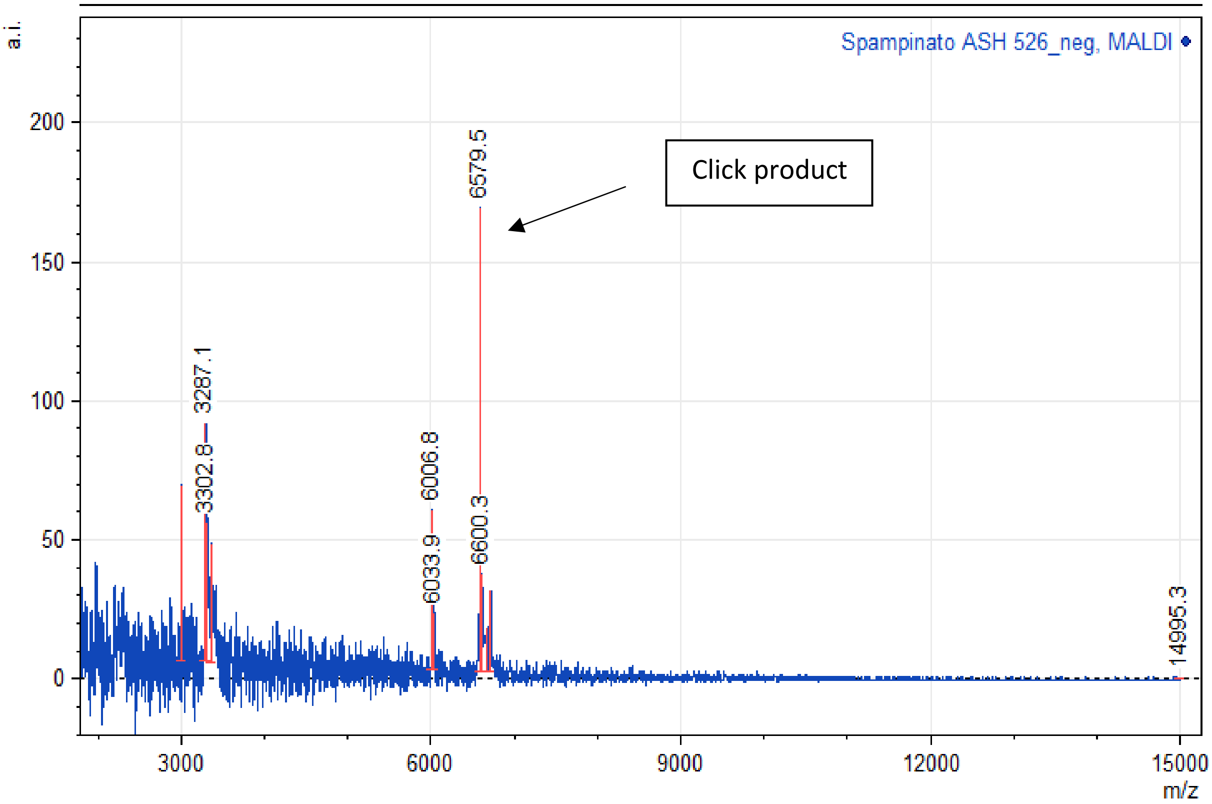

Figure S55. MALDI-TOF MS spectrum of **19ON_C^{2TCOT2}** : calculated [M] 6576.1 Da; found 6579.5 Da; Δ=3.4 Da. The peak at m/z=6006.8 Da is assigned to **19ON_C^{NH2}** due to 2TCO elimination.

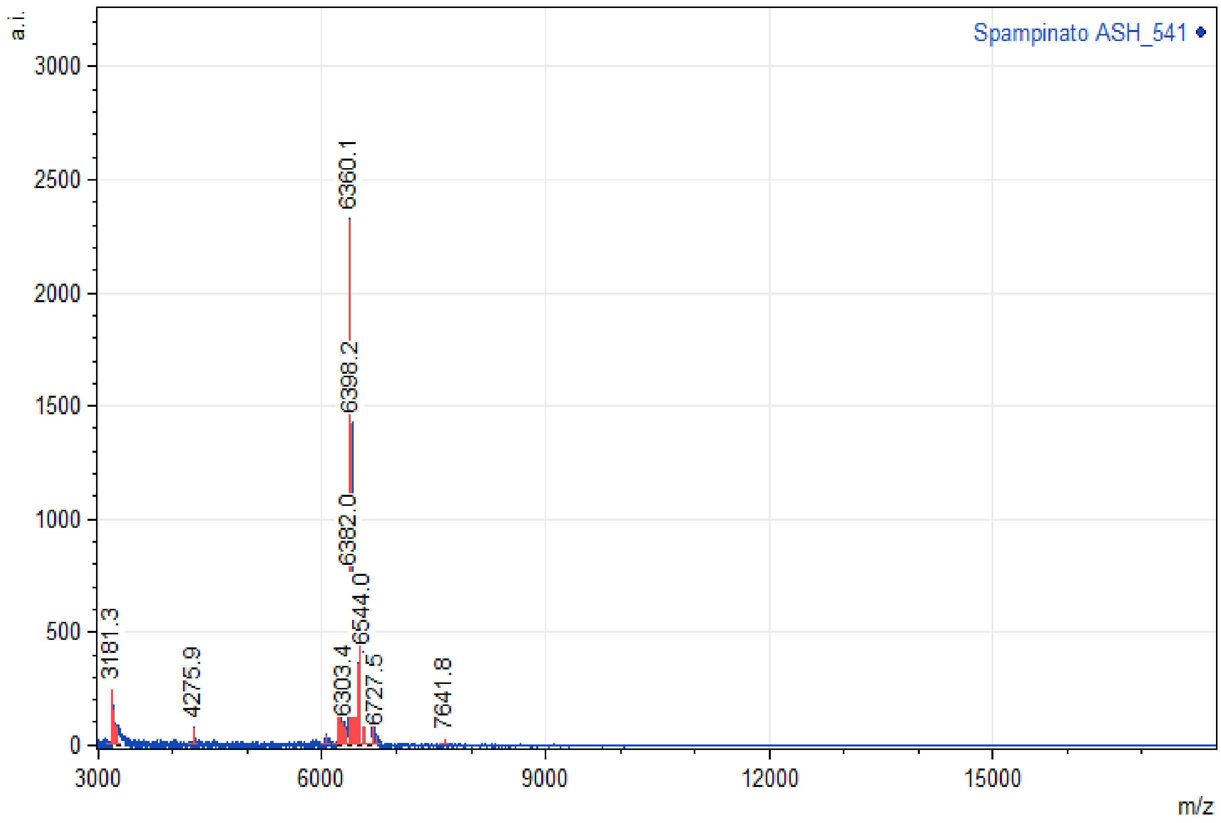

Figure S56. MALDI-TOF MS spectrum of **19ON_C$^{\mathbf{p4TCO}}$**: calculated [M] 6359.14 Da; found 6360.1 Da; Δ=1.0 Da.

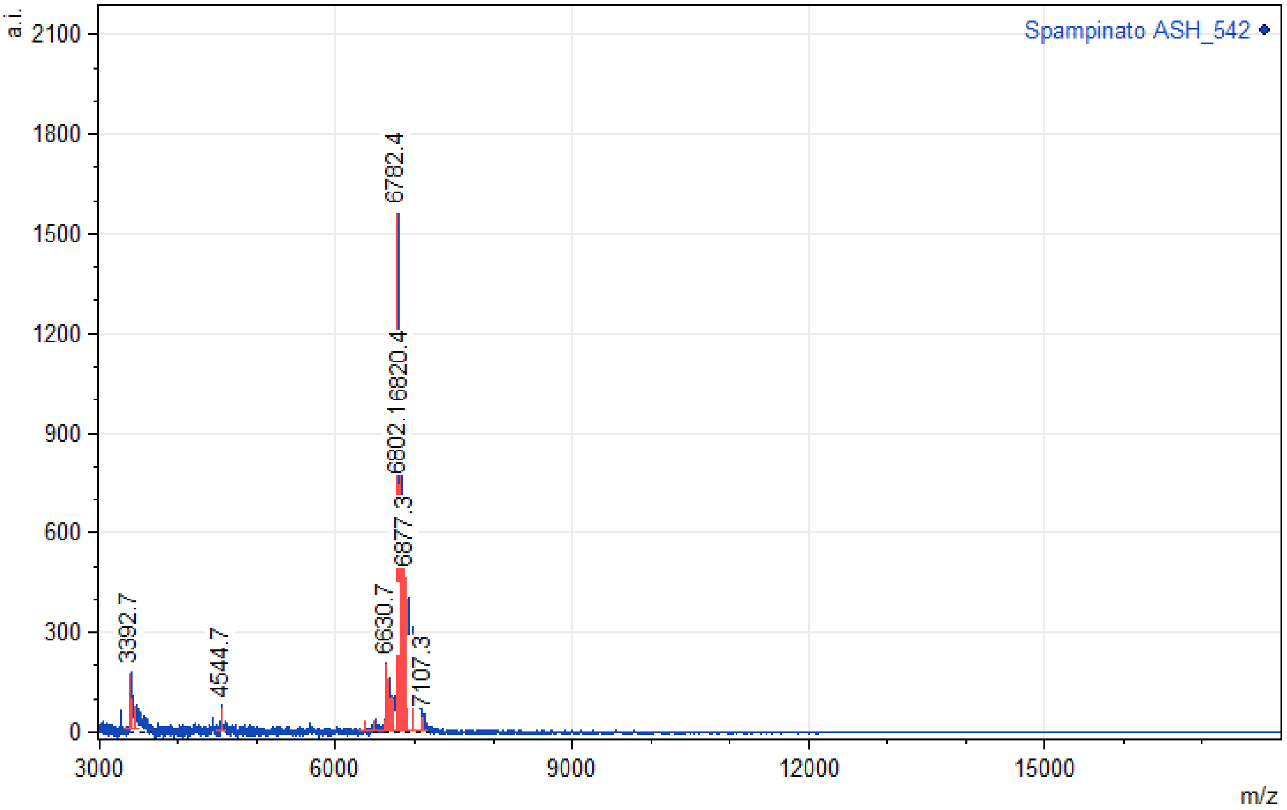

Figure S57. MALDI-TOF MS spectrum of **19ON_C^{p4TCOT1}**: calculated [M] 6781.29 Da; found 6782.4 Da; Δ=1.2 Da.

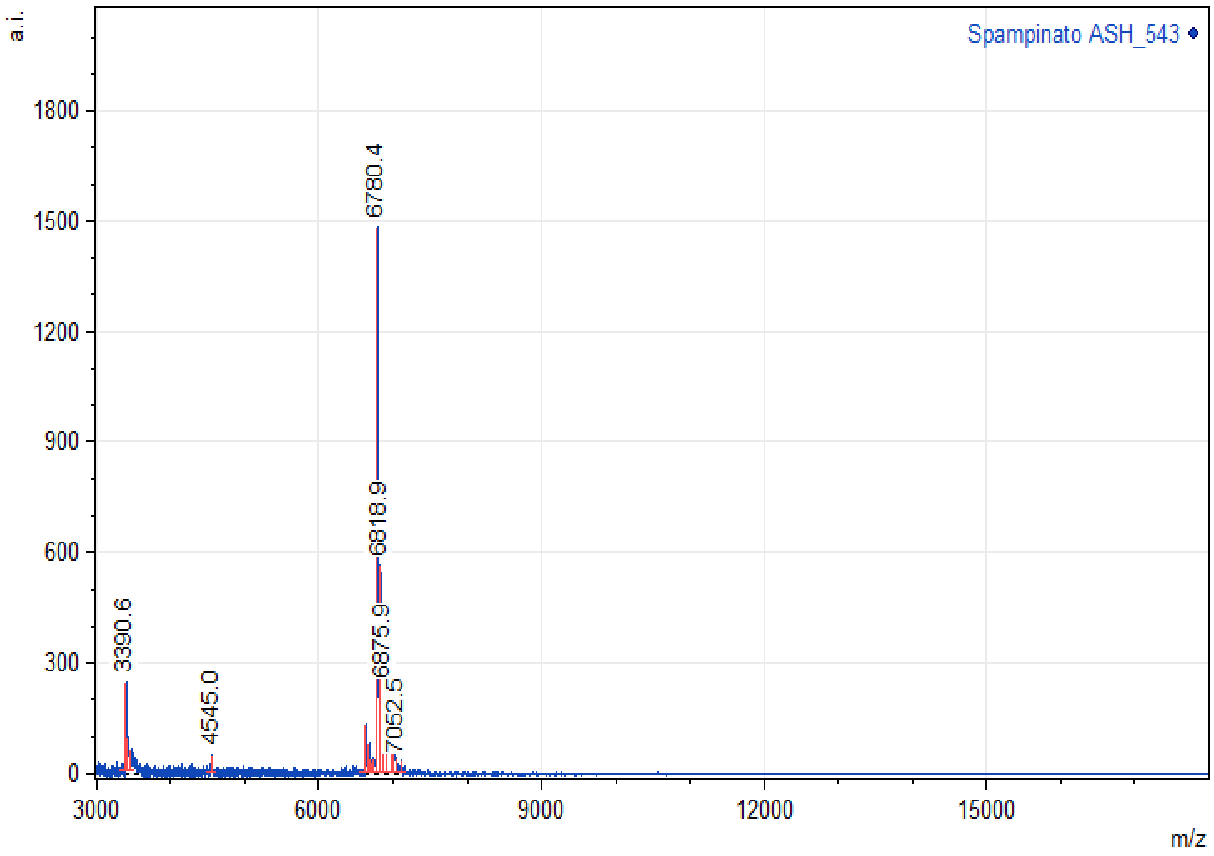

Figure S58. MALDI-TOF MS spectrum of **19ON_C^{p4TCOT2}**: calculated [M] 6779.3 Da; found 6780.4 Da; Δ=1.1 Da.

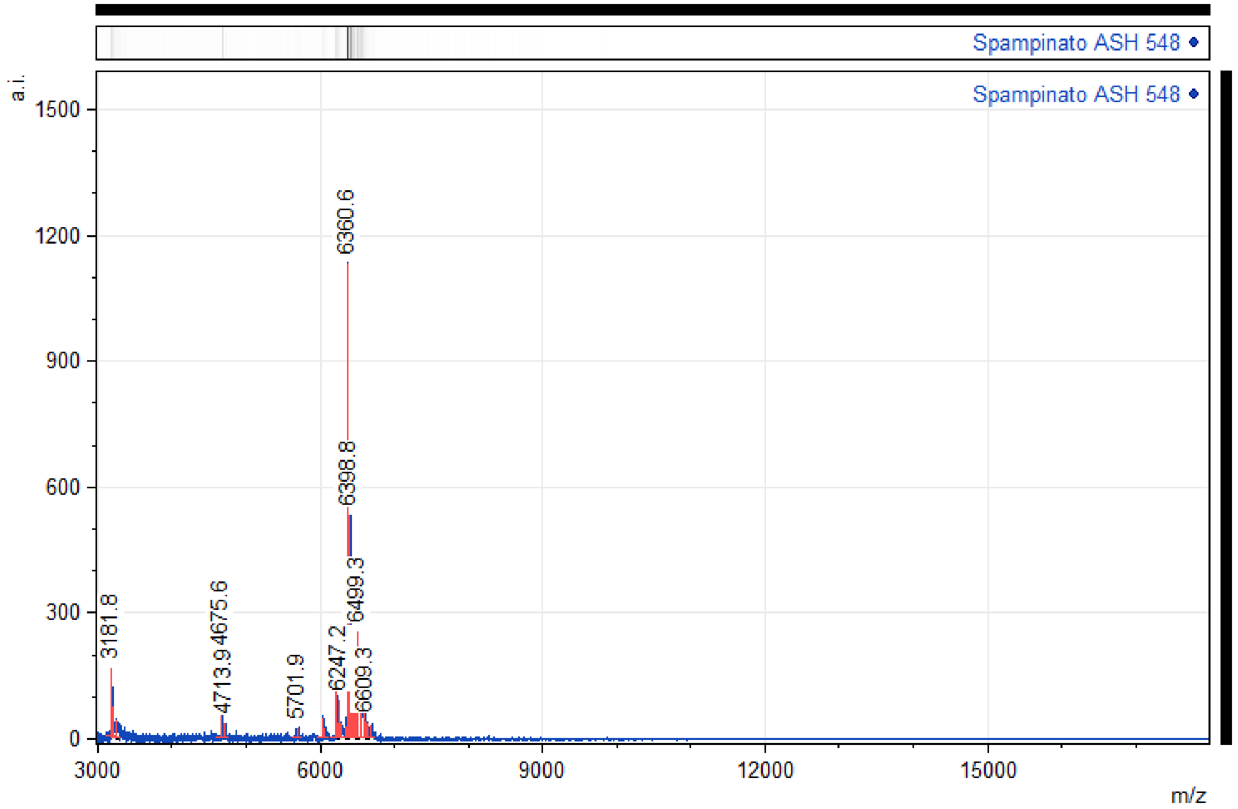

Figure S59. MALDI-TOF MS spectrum of **19ON_C^{p2TCO}**: calculated [M] 6359.1 Da; found 6360.6 Da; Δ=1.5 Da.

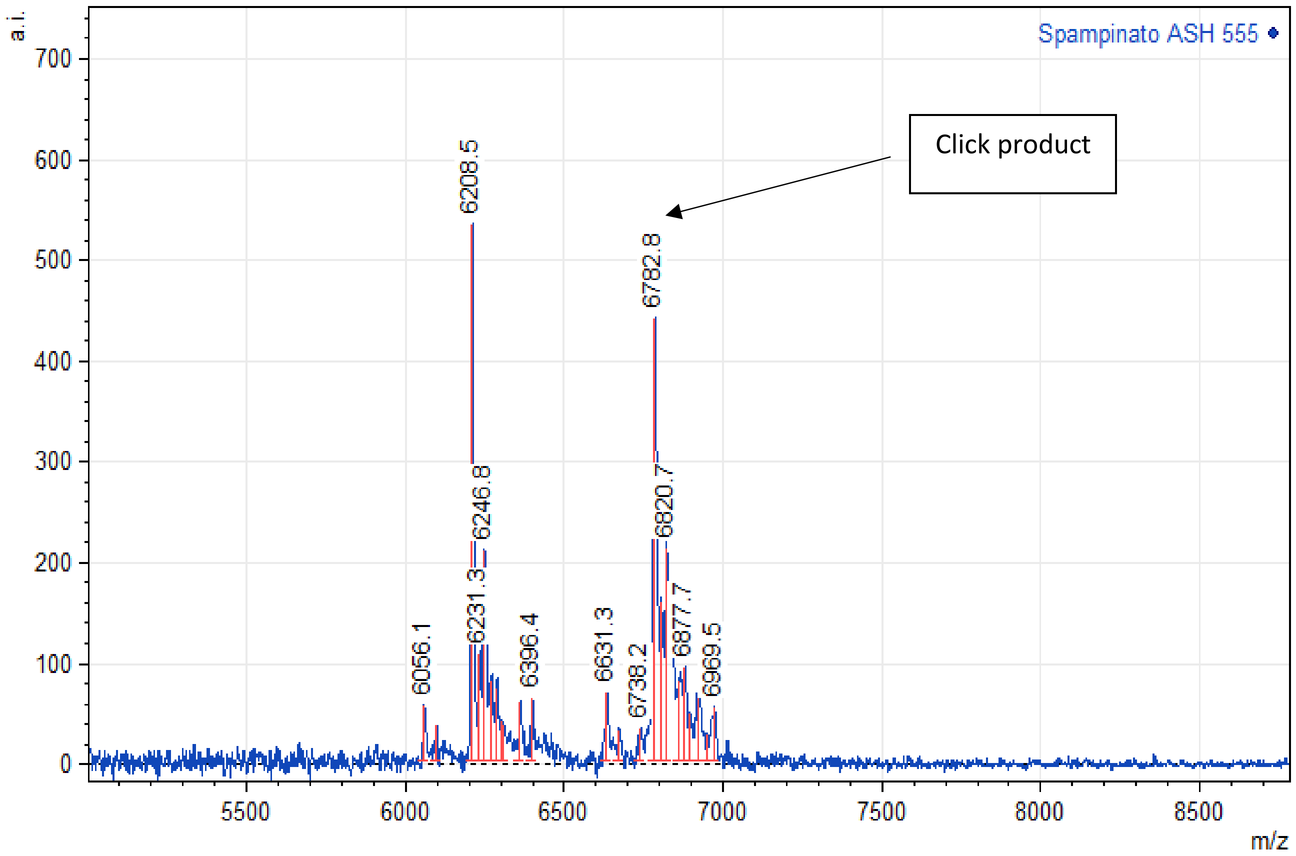

Figure S60. MALDI-TOF MS spectrum of **19ON_C$^{\mathbf{p2TCOT1}}$**; 6781.29 Da; found 6782.8 Da; Δ=1.6 Da. The peak at m/z=6208.5 Da is assigned to **19ON_C$^{\mathbf{pNH2}}$** due to 2TCO elimination.

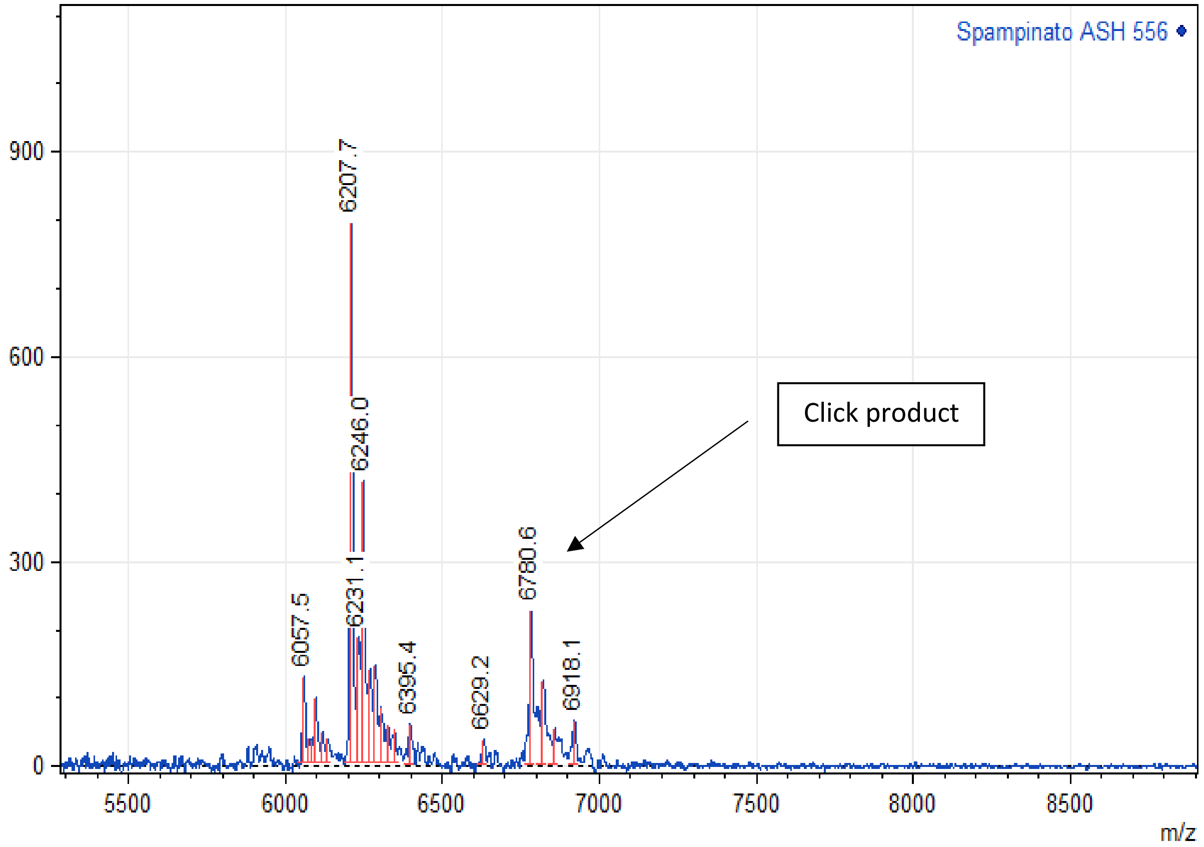

Figure S61. MALDI-TOF MS spectrum of **19ON_C^{p2TCOT2}**: calculated [M] 6779.3 Da; found 6780.6 Da; Δ=1.3 Da. The peak at m/z=6207.7 Da is assigned to **19ON_C^{pNH2}** due to 2TCO elimination.

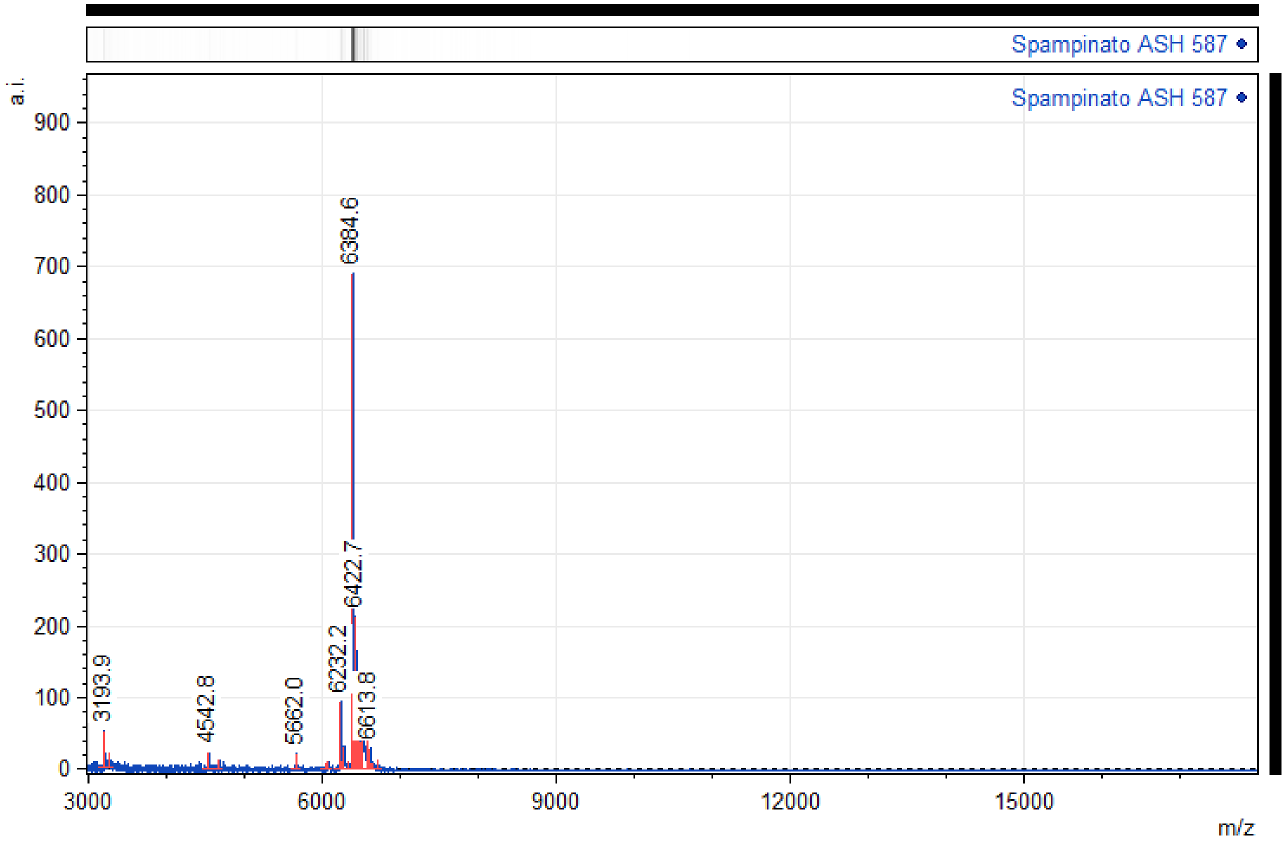

Figure S62. MALDI-TOF MS spectrum of **19ON_C^{pBCN}**: calculated [M] 6383.14 Da; found 6384.6 Da; Δ=1.5 Da.

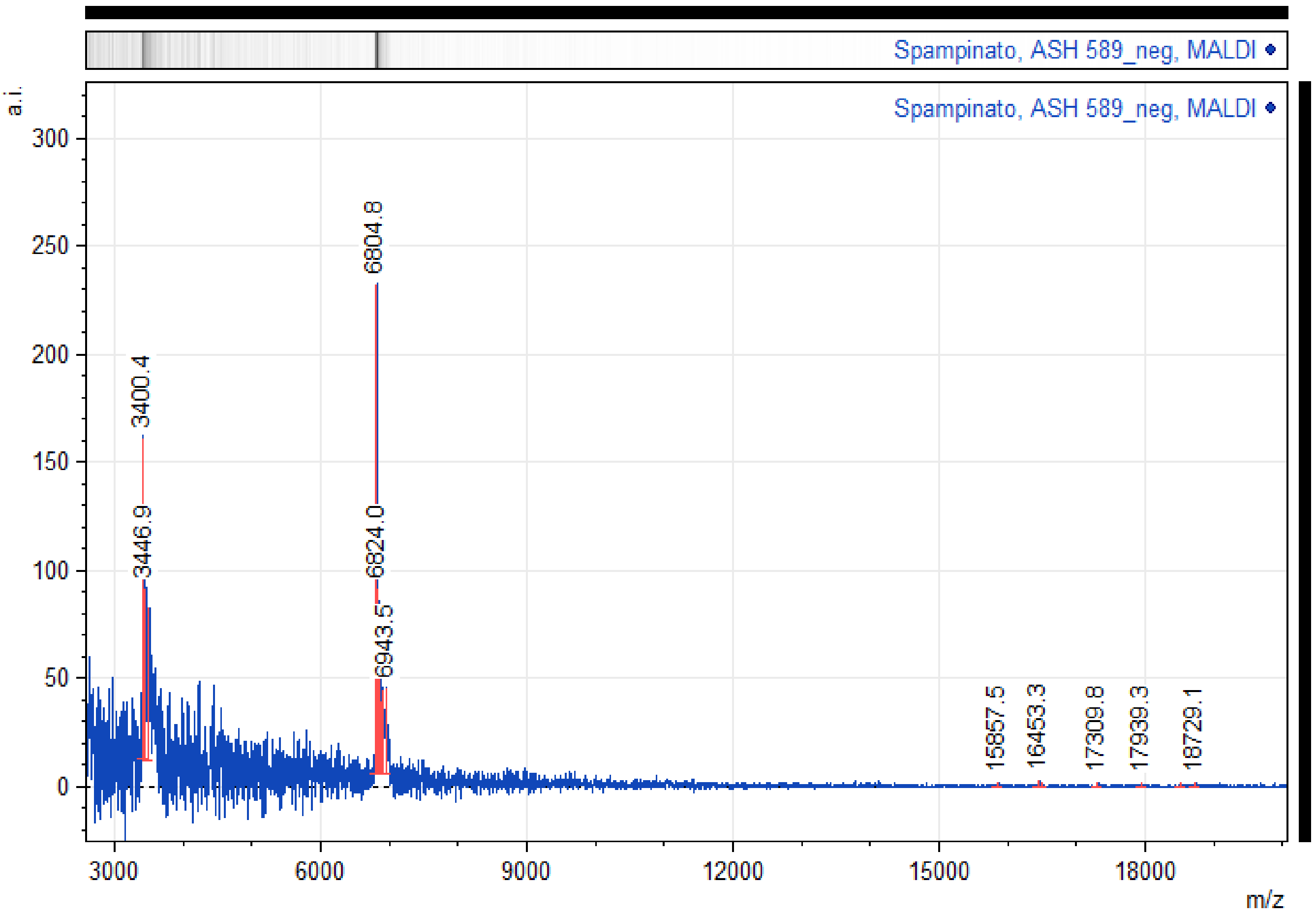

Figure S63. MALDI-TOF MS spectrum of **19ON_C**$^{\mathbf{pBCNT1}}$: calculated [M] 6805.29 Da; found 6804.8 Da; Δ=0.5 Da.

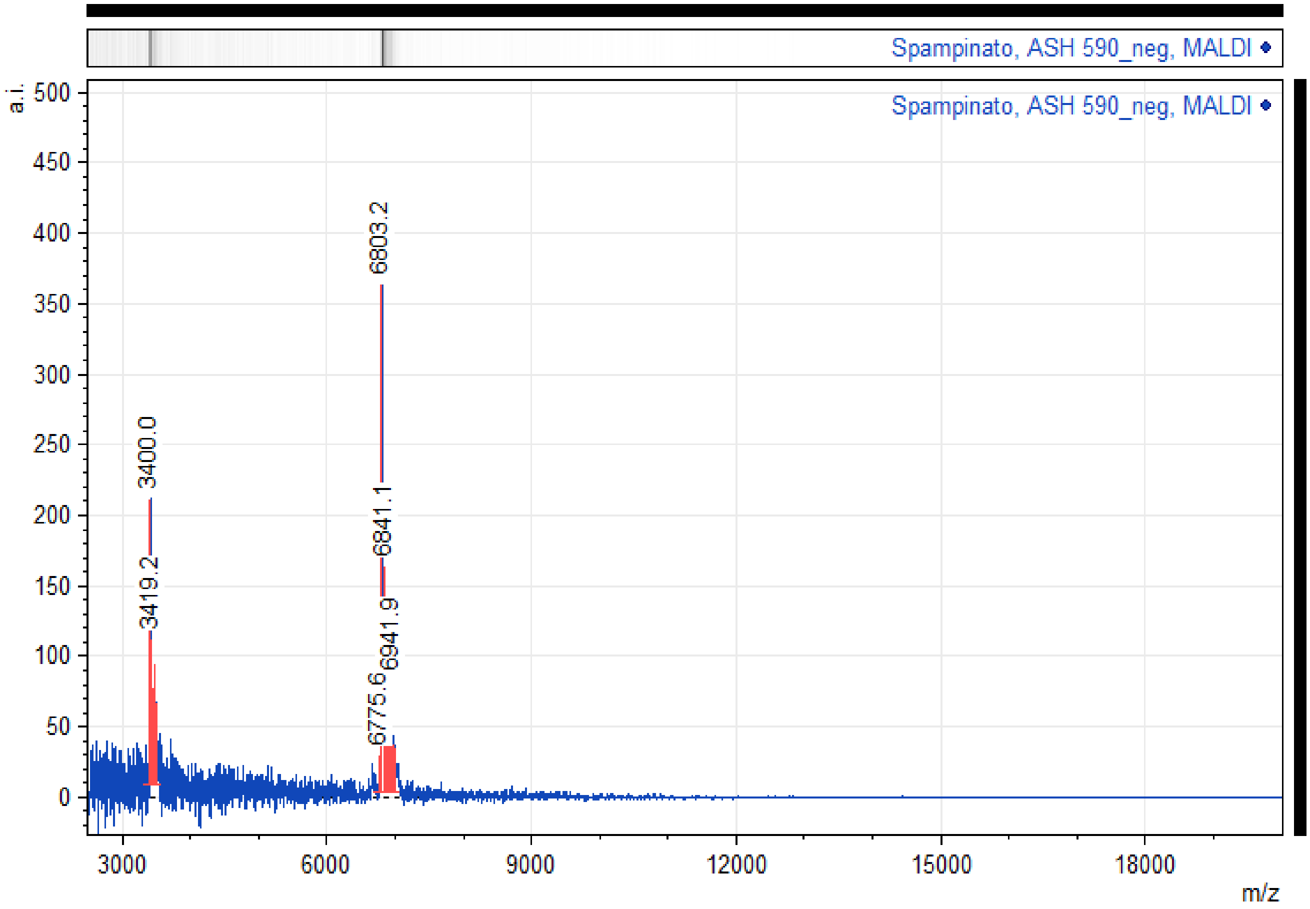

Figure S64. MALDI-TOF MS spectrum of **19ON_C$^{\mathbf{pBCNT2}}$**: calculated [M] 6803.3 Da; found 6803.2 Da; Δ=0.1 Da.

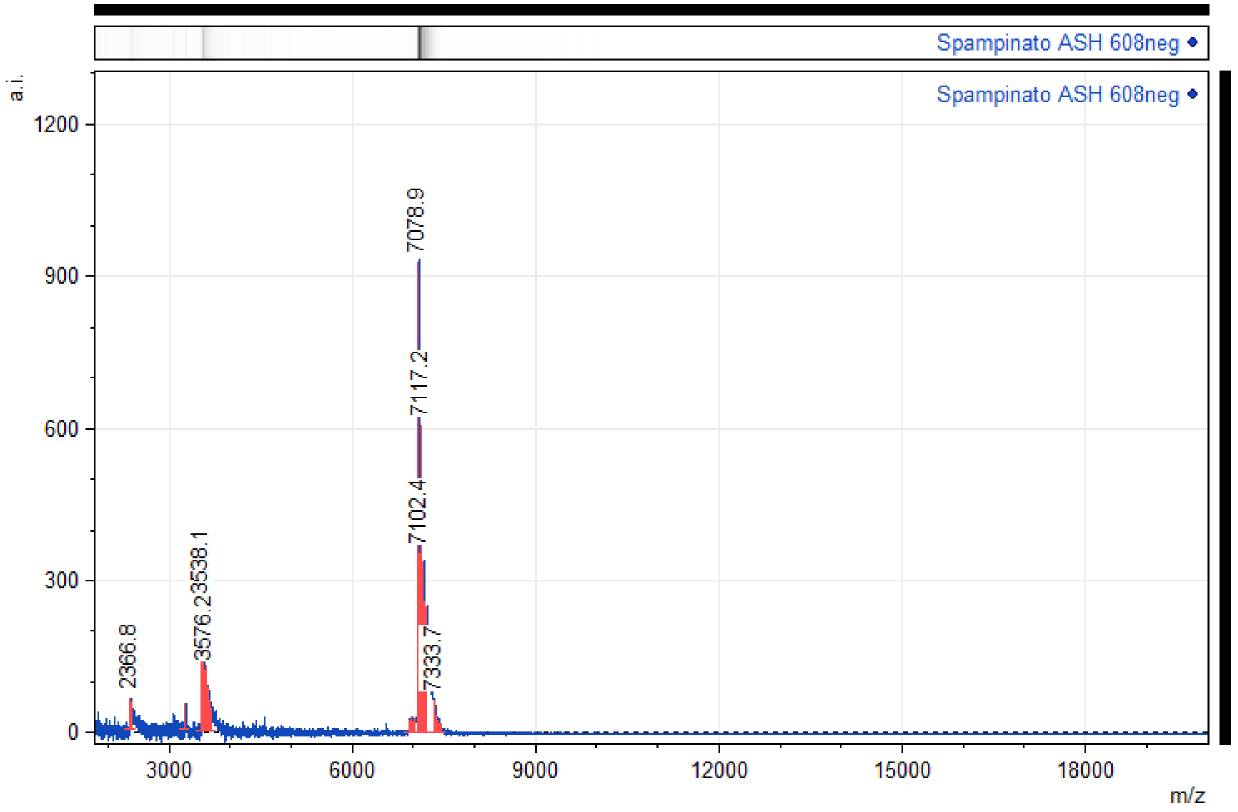

Figure S65. MALDI-TOF MS spectrum of **19ON_C^{p4TCOT4}**: calculated [M] 7077.41 Da; found 7078.9 Da; Δ=1.5 Da.

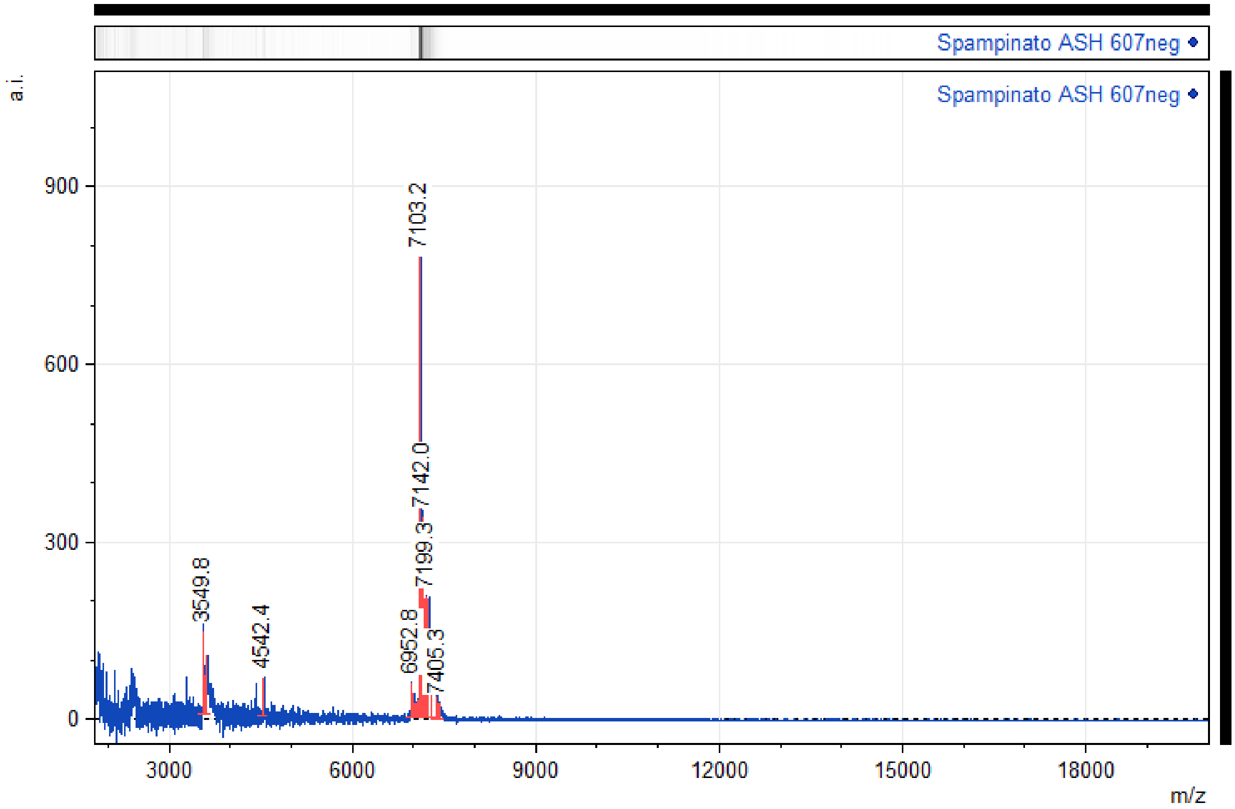

Figure S66. MALDI-TOF MS spectrum of **19ON_C^{pBCNT4}**: calculated [M] 7103.41 Da; found 7103.2 Da; Δ=0.2 Da.

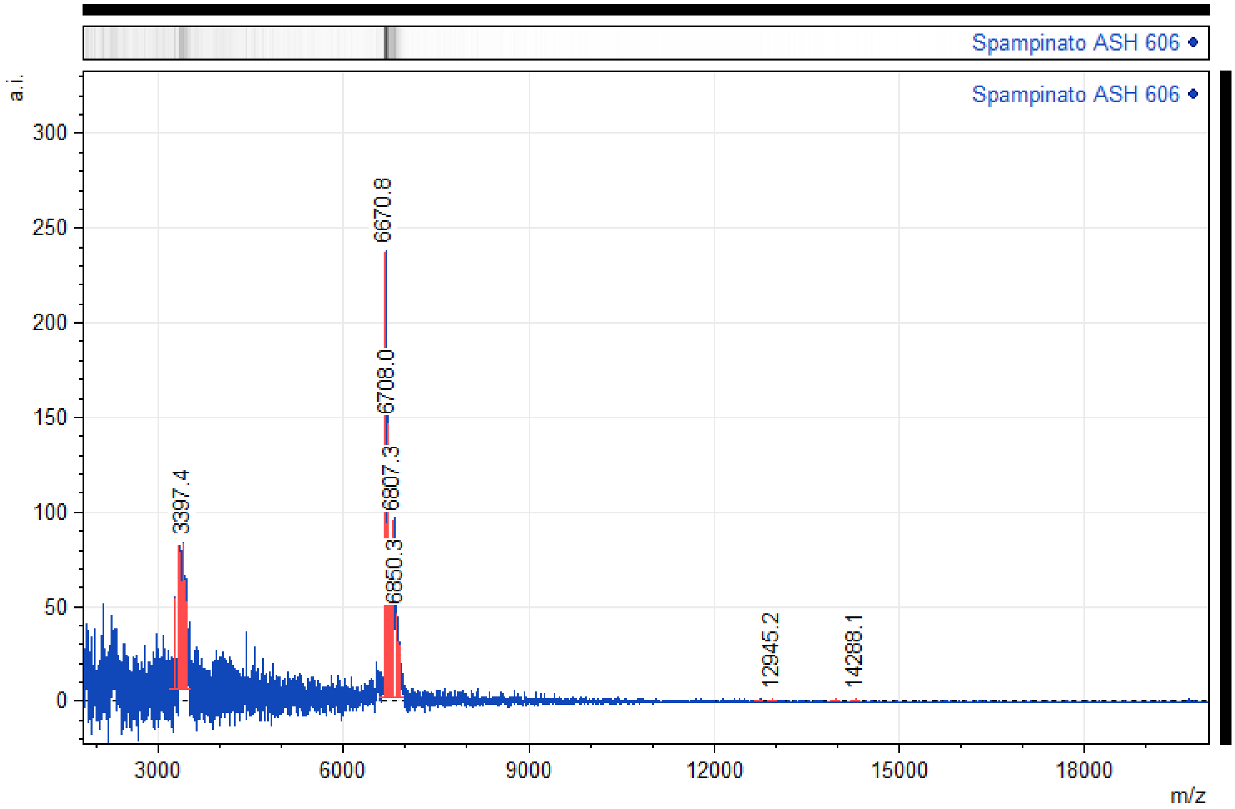

Figure S67. MALDI-TOF MS spectrum of **19ON_C^{pBCNT3}**: calculated [M] 6671.28 Da; found 6670.8 Da; Δ=0.5 Da.

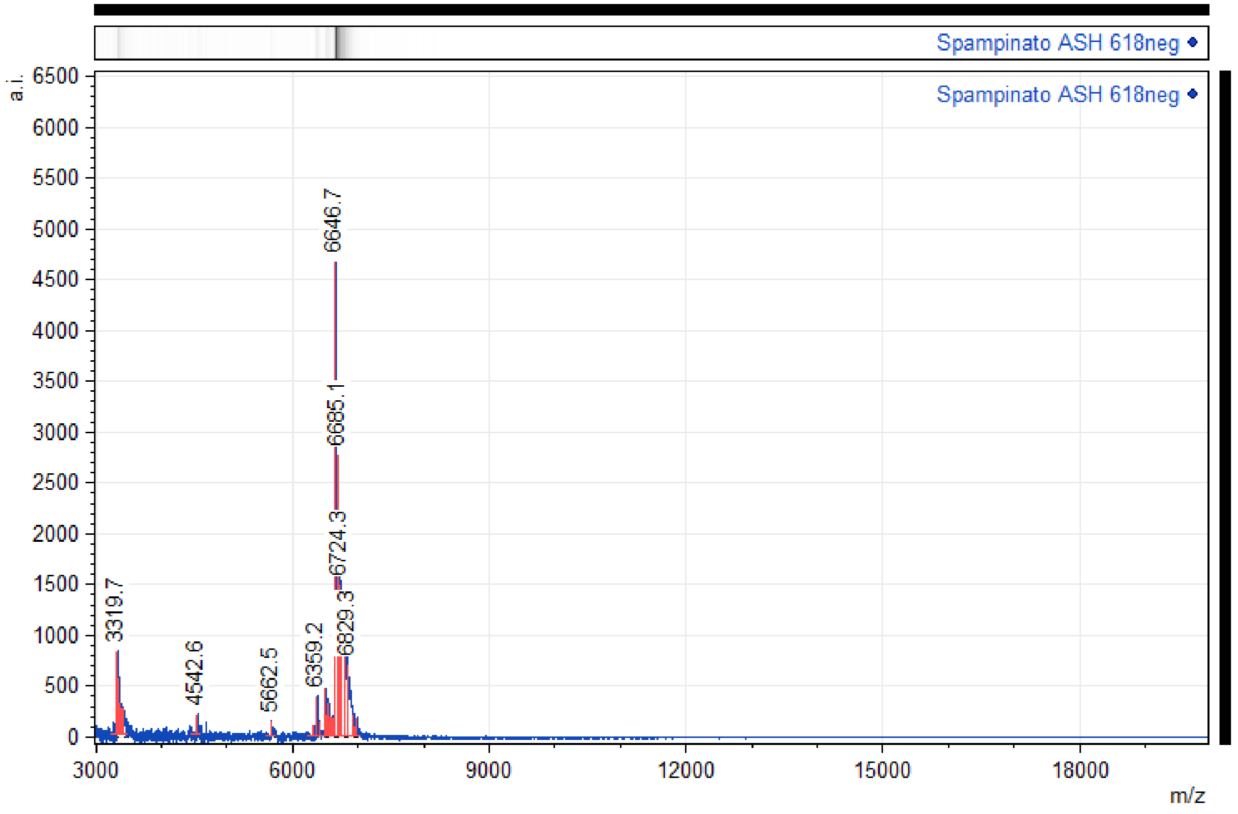

Figure S68. MALDI-TOF MS spectrum of **19ON_C$^{\mathbf{p4TCOT3}}$**: calculated [M] 6647.28 Da; found 6646.7 Da; Δ=0.6 Da.

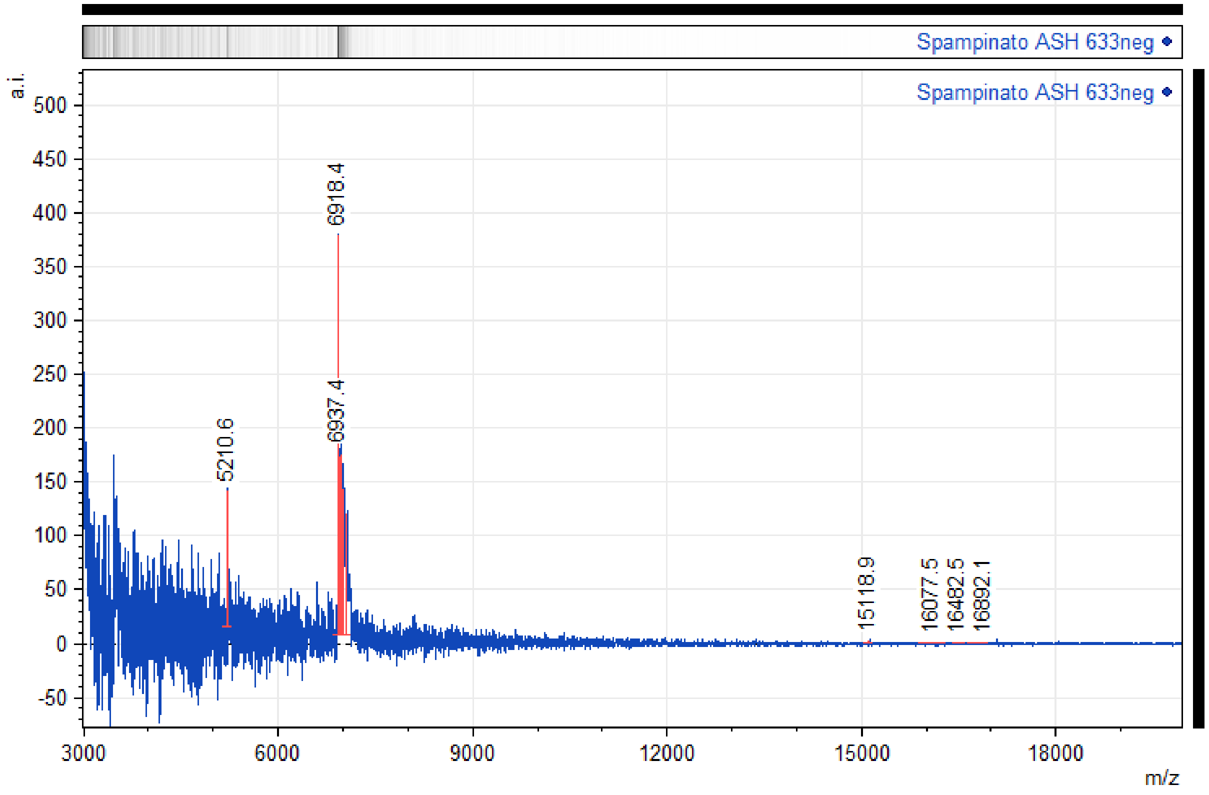

Figure S69. MALDI-TOF MS spectrum of **19ON_C^{pBCN}_FAM**: calculated [M] 6920.1 Da; found 6918.4 Da; Δ=1.7 Da.

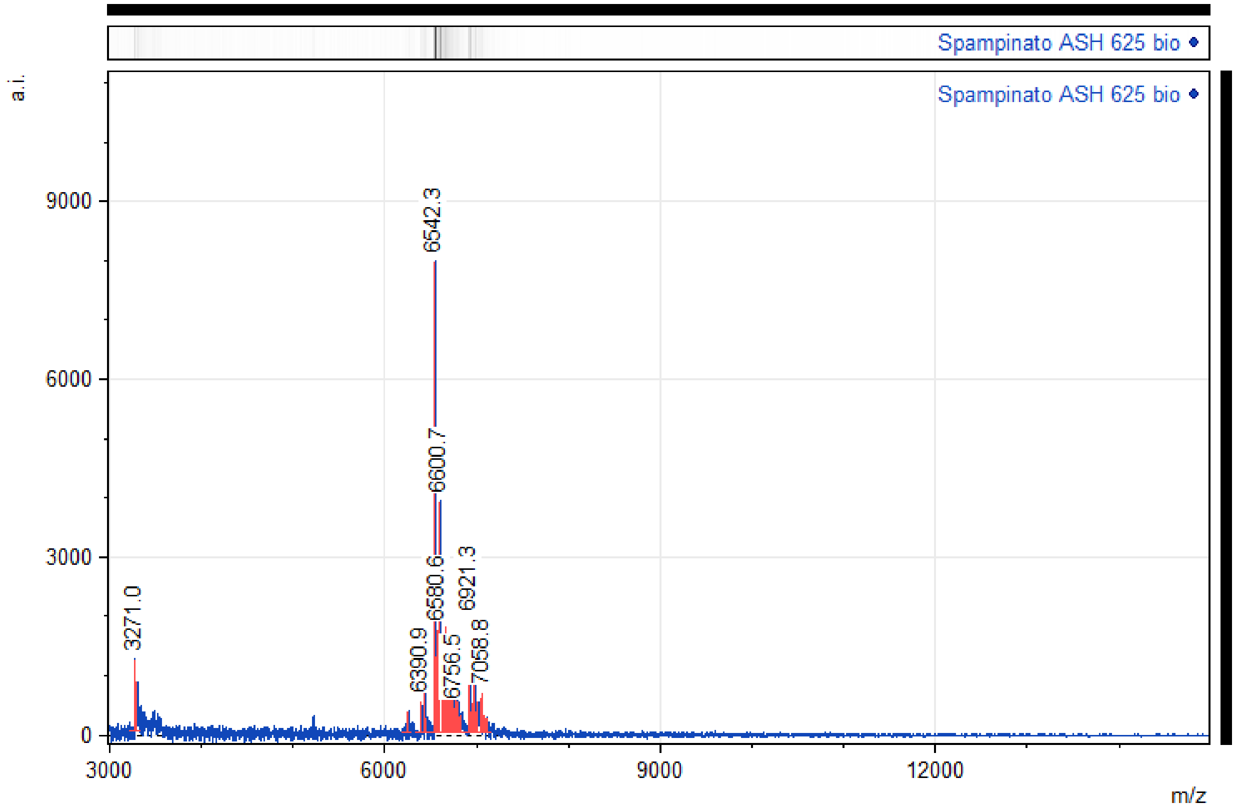

Figure S70. MALDI-TOF MS spectrum of **19DNA_C^{pBCN}_FAM**: calculated [M] 6920.1 Da; found 6921.3 Da; Δ=1.2 Da. The peak at m/z=6542.3 Da is assigned to **19Oligo1C 2xbio** template.

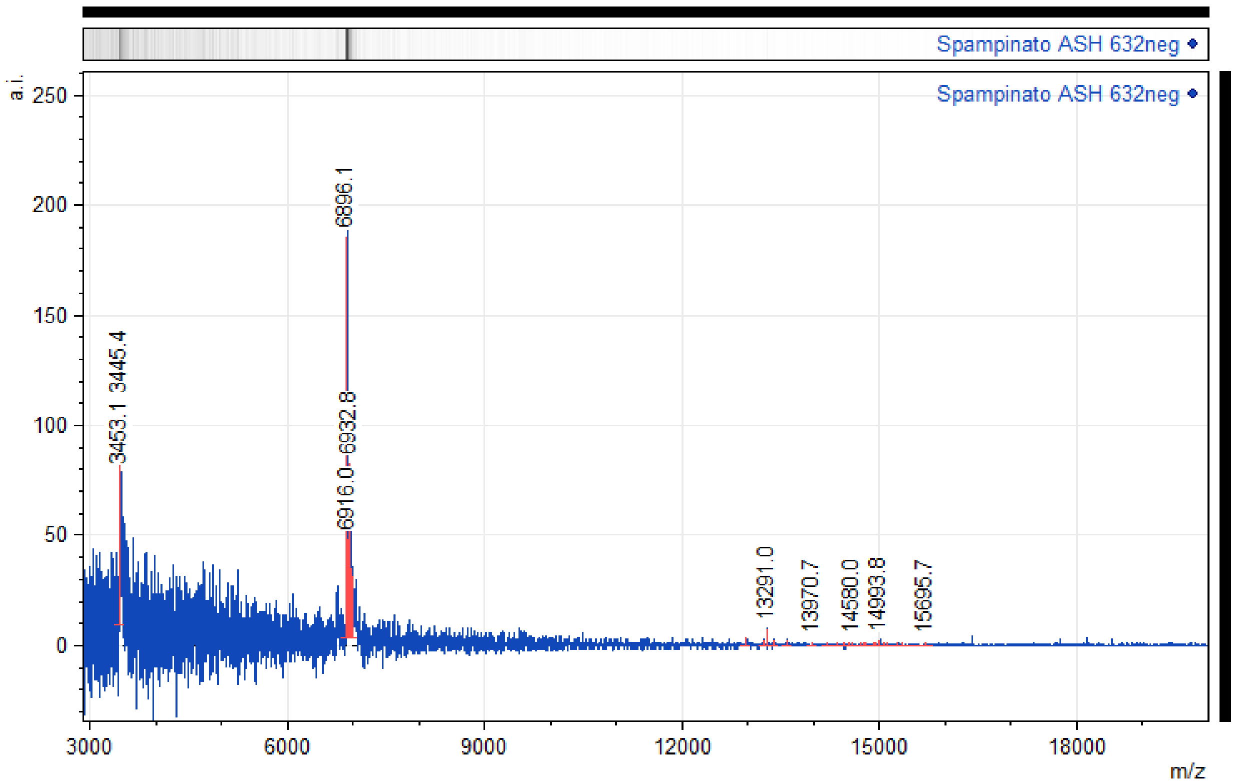

Figure S71. MALDI-TOF MS spectrum of **19ON_C^{p4TCO}_FAM**: calculated [M] 6896.1 Da; found 6896.1 Da; Δ=0 Da.

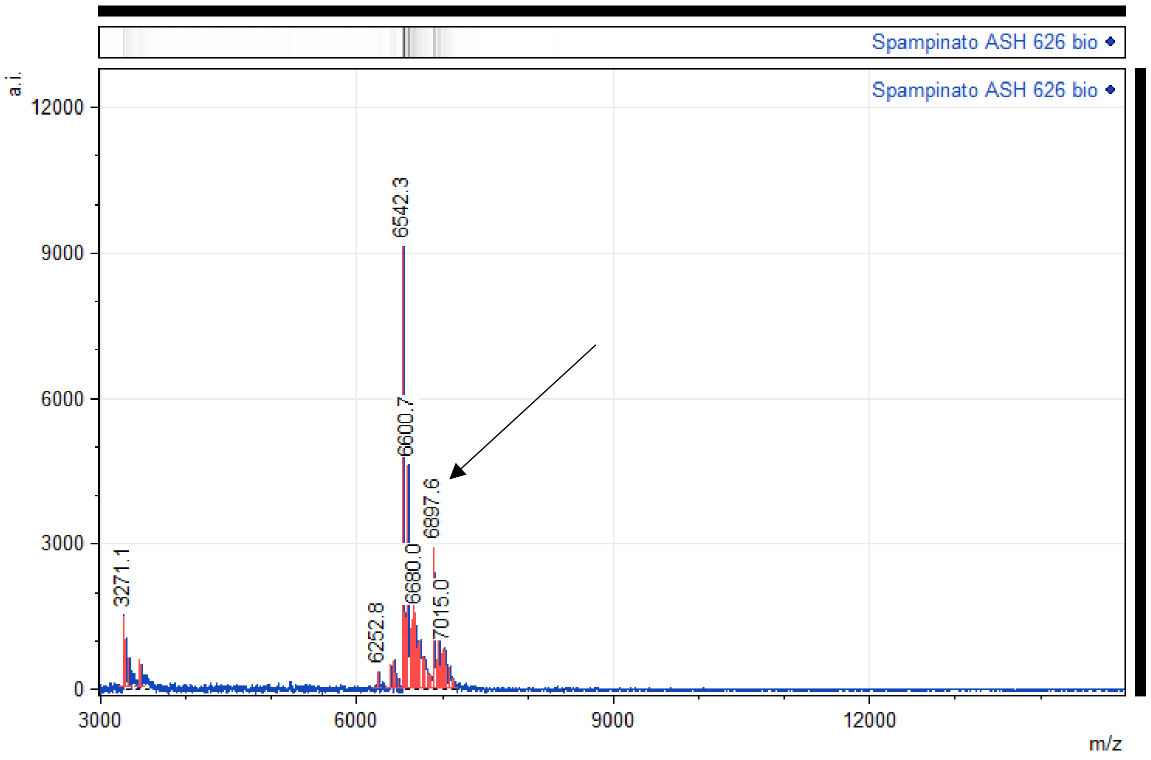

Figure S72. MALDI-TOF MS spectrum of **19DNA_C^{p4TCO}_FAM**: calculated [M] 6896.1 Da; found 6897.6 Da; Δ=1.5 Da. The peak at m/z=6542.3 Da is assigned to **19Oligo1C 2xbio** template.

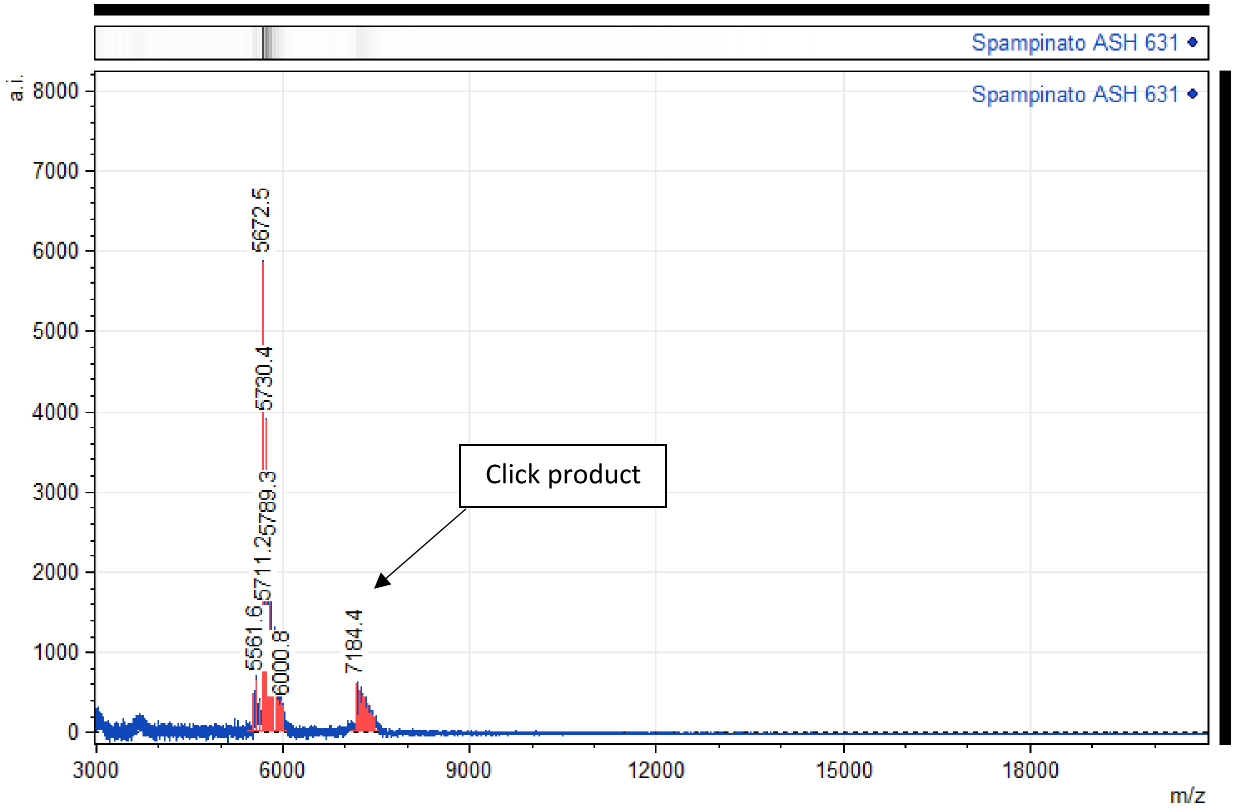

Figure S73. MALDI-TOF MS spectrum of **19DNA_C^{P4TCOT3}_FAM**: calculated [M] 7184.2 Da; found 7184.4 Da; Δ=0.2 Da. The peak at m/z=5672.5 Da is assigned to **19Oligo1C** template.

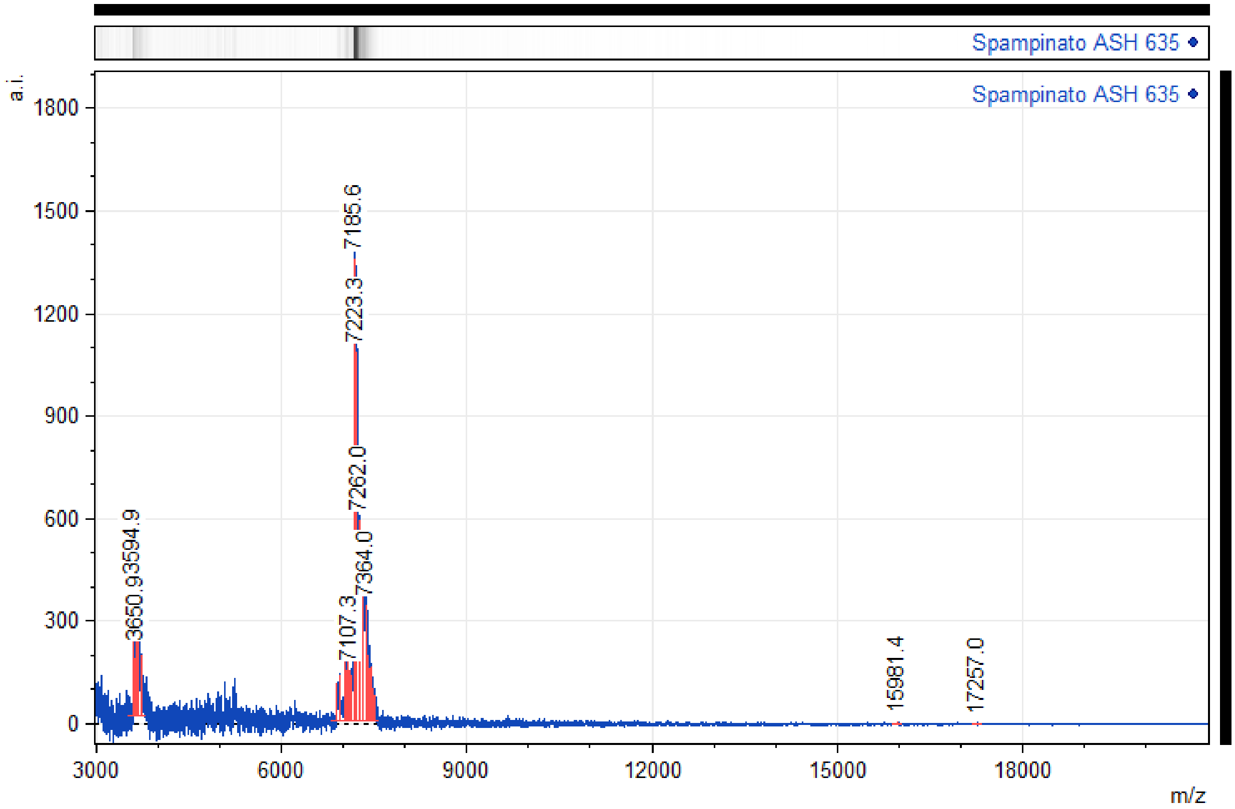

Figure S74. MALDI-TOF MS spectrum of **19ON_C^{p4TCOT3}_FAM**: calculated [M] 7184.4 Da; found 7185.6 Da; Δ=1.2 Da.

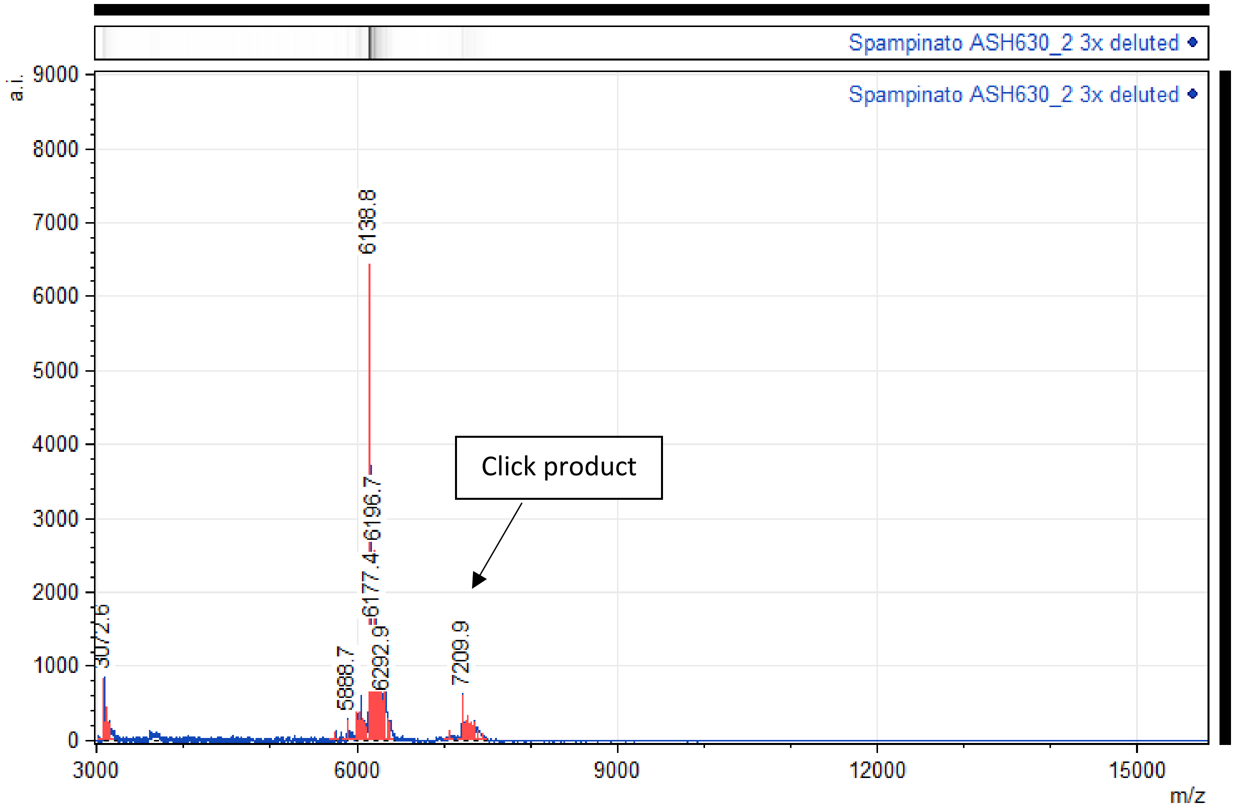

Figure S75. MALDI-TOF MS spectrum of **19DNA_C^{pBCNT3}_FAM**: calculated [M] 7208.2 Da; found 7209.9 Da; Δ=1.7 Da. The peak at m/z=6138.8 Da is assigned to **19Oligo1C_TINA** template.

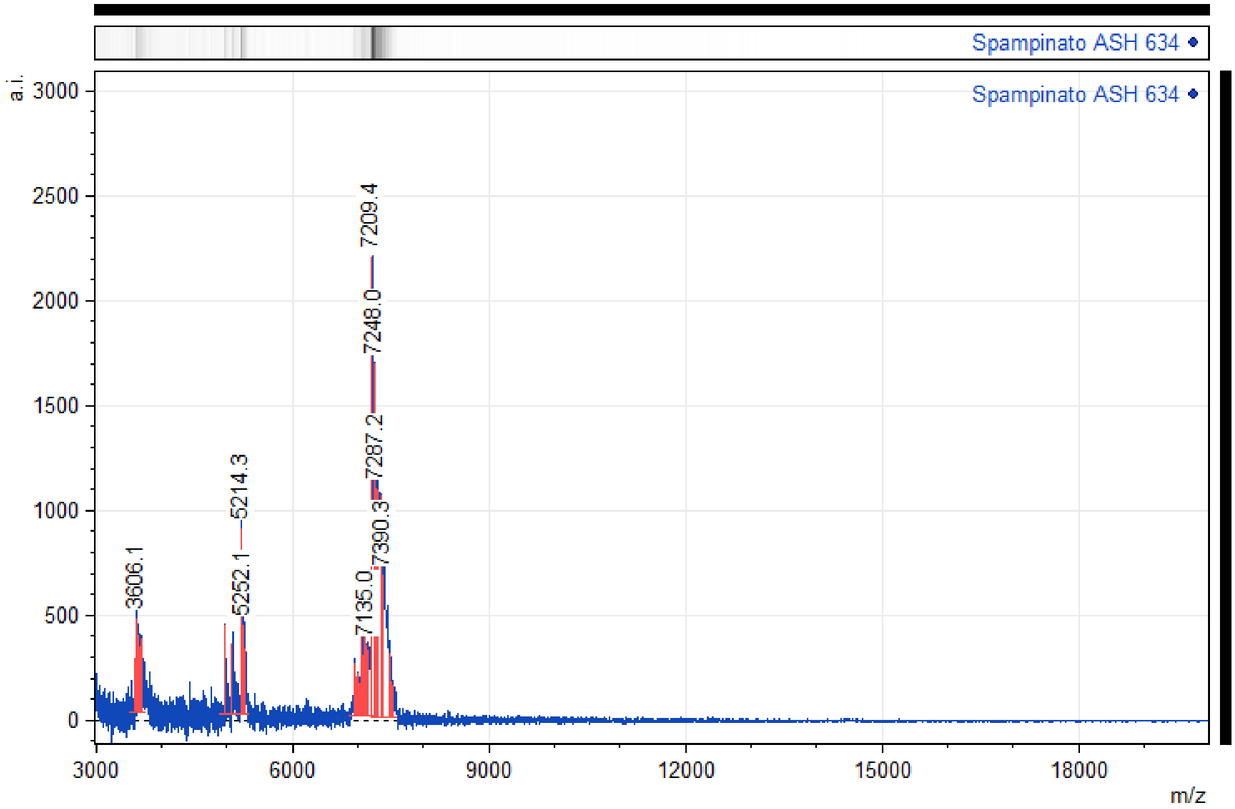

Figure S76. MALDI-TOF MS spectrum of **19ON_C^{pBCNT3}_FAM**: calculated [M] 7208.2 Da; found 7209.4 Da; Δ=1.2 Da.

9. References

1. Hashmi, A. S. K.; Häffner, T.; Yang, W.; Pankajakshan, S.; Schäfer, S.; Schultes, L.; Rominger, F.; Frey, W. Gold Catalysis: Non-Spirocyclic Intermediates in the Conversion of Furanynes by the Formal Insertion of an Alkyne into an Aryl-Alkyl C-C Single Bond. *Chem. Eur. J.* **2012**, *18*, 10480-10486.

2. a) Garg, N. K.; Woodroofe, C. C.; Lacenere, C. J.; Quake, S. R.; Stoltz, B. M. (2005) A ligand-free solid-supported system for Sonogashira couplings: applications in nucleoside chemistry. *Chem. Commun.* **2005**, 4551-4553; b) Jäger, S.; Rasched, G.; Kornreich-Leshem, H.; Engeser, M.; Thum, O.; Famulok, M. A versatile toolbox for Variable DNA Functionalization at High Density. *J. Am. Chem. Soc.* **2005**, *127*, 15071-15082.

3. Galeta, J.; Dzijak, R.; Obořil, J.; Dračínský, M.; Vrabel, M. A Systematic Study of Coumarin–Tetrazine Light-Up Probes for Bioorthogonal Fluorescence Imaging. *Chem. Eur. J.* **2020**, *26*, 9945-9953.

4. Vázquez, A.; Dzijak, R.; Dračínský, M.; Rampmaier, R.; Siegl, S. J.; Vrabel, M. Mechanism-Based Fluorogenic trans-Cyclooctene–Tetrazine Cycloaddition. *Angew. Chem. Int. Ed.* **2016**, 1334-1337.

5. Kužmová, E.; Zawada, Z.; Navrátil, M.; Günterová, J.; Kraus, T. Flow cytometric determination of cell cycle progression via direct labeling of replicated DNA. *Anal. Biochem.* **2021**, *614*, 114002.

6. Loehr, M. O.; Luedtke, N. W. A Kinetic and Fluorogenic Enhancement Strategy for Labeling of Nucleic Acids. *Angew. Chem. Int. Ed.* **2022**, *61*, e202112931.

7. Marty, M. T.; Baldwin, A. J.; Marklund, E. G.; Hochberg, G. K. A.; Benesch, J. L. P.; Robinson, C. V. Bayesian Deconvolution of Mass and Ion Mobility Spectra: From Binary Interactions to Polydisperse Ensembles. *Anal. Chem.* **2015**, *87*, 4370-4376.
